# A comparison of key aspects of gene regulation in *Streptomyces coelicolor* and *Escherichia coli* using nucleotide-resolution transcription maps produced in parallel by global and differential RNA sequencing

**DOI:** 10.1111/mmi.12810

**Published:** 2014-10-27

**Authors:** David A Romero, Ayad H Hasan, Yu-fei Lin, Louise Kime, Olatz Ruiz-Larrabeiti, Mia Urem, Giselda Bucca, Lira Mamanova, Emma E Laing, Gilles P van Wezel, Colin P Smith, Vladimir R Kaberdin, Kenneth J McDowall

**Affiliations:** 1 Astbury Centre for Structural Molecular Biology, School of Molecular and Cellular Biology, Faculty of Biological Sciences, University of LeedsLeeds, LS2 9JT, UK; 2 Department of Immunology, Microbiology and Parasitology, University of the Basque Country UPV/EHULeioa, Spain; 3 Institute of Biology, Sylvius Laboratories, Leiden UniversityLeiden, NL-2300 RA, The Netherlands; 4 Department of Microbial & Cellular Sciences, Faculty of Health & Medical Sciences, University of SurreyGuildford, GU2 7XH, UK; 5 The Wellcome Trust Sanger Institute, Wellcome Trust Genome CampusHinxton, Cambridge, CB10 1SA, UK; 6 IKERBASQUE, Basque Foundation for Science48011, Bilbao, Spain

## Abstract

*Streptomyces coelicolor* is a model for studying bacteria renowned as the foremost source of natural products used clinically. Post-genomic studies have revealed complex patterns of gene expression and links to growth, morphological development and individual genes. However, the underlying regulation remains largely obscure, but undoubtedly involves steps after transcription initiation. Here we identify sites involved in RNA processing and degradation as well as transcription within a nucleotide-resolution map of the transcriptional landscape. This was achieved by combining RNA-sequencing approaches suited to the analysis of GC-rich organisms. *Escherichia coli* was analysed in parallel to validate the methodology and allow comparison. Previously, sites of RNA processing and degradation had not been mapped on a transcriptome-wide scale for *E. coli*. Through examples, we show the value of our approach and data sets. This includes the identification of new layers of transcriptional complexity associated with several key regulators of secondary metabolism and morphological development in *S. coelicolor* and the identification of host-encoded leaderless mRNA and rRNA processing associated with the generation of specialized ribosomes in *E. coli*. New regulatory small RNAs were identified for both organisms. Overall the results illustrate the diversity in mechanisms used by different bacterial groups to facilitate and regulate gene expression.

## Introduction

*Streptomyces coelicolor* serves as an important model for studying the biology of streptomycetes, which are the source of numerous antibacterial, anticancer, immunosuppressive, anthelmintic and antifungal agents (Shiomi and Omura, 2004; Baltz, 2008; Caffrey *et al*., 2008; Graziani, 2009; Olano *et al*., 2009). The production of these clinically important secondary metabolites is in most cases stimulated by nutrient starvation and accompanies a complex developmental programme, whereby spores are produced by aerial hyphae that grow out from a vegetative mycelial mass (Flardh and Buttner, 2009; Claessen *et al*., 2014). Numerous genes and growth conditions that influence secondary metabolite production and morphological development have been identified for *S. coelicolor* and for some the underlying changes in gene expression have been determined using microarrays and to a lesser extent proteomic approaches (for reviews, see van Wezel and McDowall, 2011; Liu *et al*., 2013). Despite this considerable effort, there is no coherent understanding of the pathways that control the flow of genetic information at the level of the whole cell. A limiting factor is the scarcity of experimental information on the organization and regulation of the transcriptional units that encode proteins, and RNA components of the translation and regulatory machinery. Elements such as promoters, transcription start sites and untranslated regions have not been identified experimentally on a genome-wide scale. Other key aspects of gene expression for which virtually no information was available for *S. coelicolor*, and indeed any other bacterial species, were the sites of RNA cleavage that facilitate rapid mRNA turnover (Carpousis *et al*., 2009), a key process that ensures translation follows programmes of transcription, and generate the RNA components of the translation machinery (Deutscher, 2009; Hartmann *et al*., 2009). Currently, genome-wide knowledge of operons and *cis*-regulatory elements is largely inferred from a promoter-centred analysis of modules of coexpressed genes identified from a large compendium of transcriptome data (Castro-Melchor *et al*., 2010).

Here we provide new insight into the steps that control the flow of genetic information in *S. coelicolor* on a genome-wide scale. This was achieved by combining two RNA sequencing approaches, one that identifies and ‘differentiates’ sites of transcription initiation and endonucleolytic cleavage (dRNA-seq) and another that provides a ‘global’ view of transcript abundance and the boundaries of transcription (gRNA-seq) (Lin *et al*., 2013). We also conducted a parallel analysis of *Escherichia coli*, which has been used as a reference for studying *Streptomyces* gene regulation (Taguchi *et al*., 1990; Messer and Zakrzewska-Czerwinska, 2002; Bralley *et al*., 2006; Laing *et al*., 2006; Gatewood and Jones, 2010), as well as being an important model, most recently in the development of systems-level understanding (Schwille and Diez, 2009; De Smet and Marchal, 2010; Hyduke and Palsson, 2010; Zhang *et al*., 2010; Porter *et al*., 2011). The inclusion of *E. coli* provided insight over and above that obtained by many groups via extensive study using traditional gene-specific methods such as northern blotting and primer extension (Kime *et al*., 2008). For example, previously undetected small RNAs, leaderless mRNAs and endonucleolytic cleavage associated with the production of specialized ribosomes were identified. The analysis of *E. coli* also validated our approach and revealed the limitations of previous global analyses, for example, in the mapping of transcription start sites (Cho *et al*., 2009). Moreover, it also allowed comparison with *S. coelicolor*, which revealed substantially differences at the level of RNA processing and degradation as well as translation. These findings reinforce the need to study multiple ‘model organisms’ in order to gain a representative view of the mechanisms used by bacteria to regulate gene expression.

## Results

### Overview of RNA-seq approaches

Sites of transcription initiation and RNA cleavage were mapped for *S. coelicolor* M145 and *E. coli* BW25113 grown in liquid cultures (see *Experimental procedures*). M145 was grown until the mycelia became visibly pigmented. This point was chosen as it is known to coincide with exit from exponential growth and the onset of secondary metabolism (Bibb, 2005; van Wezel and McDowall, 2011). BW25113 was grown to the midpoint of exponential growth, a phase routinely used to study *E. coli*. Both M145 and BW25113 were chosen for this study as they have been, and continue to be, used extensively worldwide for biochemical and genetic studies (Chater *et al*., 2002; Baba *et al*., 2006). At the points of growth described above, total RNA was isolated and enriched for mRNA by depleting 16S and 23S rRNA. To differentiate 5′-triphosphorylated ends generated by transcription from 5′-monophosphorylated ends produced by RNA processing or degradation, an aliquot was incubated with tobacco acid pyrophosphatase (TAP) (Breter and Rhoads, 1979), which leaves a monophosphate on 5′ ends that were originally triphosphorylated. A second aliquot was incubated under the same conditions, but without TAP. 5′ end fragments were then cloned and sequenced via a strategy that required the ligation of an adapter to 5′-monophosphorylated ends (Lin *et al*., 2013). A significant increase in the number of sequencing reads at a specific position following TAP treatment provided an identifier of a transcription start site (TSS). It should be noted that after the addition of the 5′ adaptor the RNA was fragmented to allow good coverage of the 5′ ends of large transcripts and intermediates. A third aliquot of each RNA sample was analysed using an amplification-free form of strand-specific global RNA-seq (Mamanova *et al*., 2010; Lin *et al*., 2013). This allowed transcripts to be mapped along their entire length. Importantly, the RNA-seq approaches used here should not be affected unduly by high GC content (Mamanova *et al*., 2010; Lin *et al*., 2013), a feature of streptomycete genomes. In brief, the gRNA-seq approach avoided the use of PCR, which introduces bias with GC-rich templates (McDowell *et al*., 1998), while the dRNA-seq approach did not use Terminator™ 5′ monophosphate-dependent exonuclease (TEX), which in our hands does not degrade *Streptomyces* RNA efficiently (data not shown), presumably because of the high prevalence of stable secondary structures. In the original and most widely used dRNA-seq approach to date, TSSs are identified by their resistance to treatment with TEX (Sharma *et al*., 2010).

### Identification of transcription start sites

**Fig. 1:**
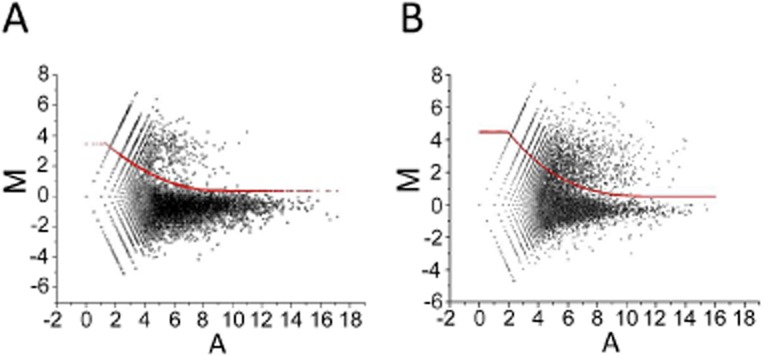
M-A scatterplots of values from the differential RNA-seq analysis. (A) and (B) show data for *S. coelicolor* M145 and *E. coli* BW25113 (seq). The M values correspond to Log_2_ (plus/minus) and A values to (Log_2_ plus + Log_2_ minus)/2, where minus and plus refer to the number of reads before and after treatment with TAP. The points correspond to individual genome positions, not genes. For further details, see *Experimental procedures*. In each panel, the red line represents the upper boundary of the population of values corresponding to sites of processing and degradation (see main text). The upper boundaries were placed manually to enclose the majority of the lower population, while taking into consideration the spread of M values scattered around 0. The boundaries were then described by polynomial equations. These were M = 0.054A^2^ − 0.96A + 4.68 and M = −0.003A^3^ + 0.13A^2^ − 1.57A + 7.08 for *S. coelicolor* and *E. coli* respectively.

To classify the 5′ ends identified by dRNA-seq, we first analysed M-A (ratio-intensity) scatterplots of the reads obtained before and after TAP treatment (Fig. 1). For both *S. coelicolor* and *E. coli*, we found two populations of values, as described previously for *Propionibacterium acnes* (Lin *et al*., 2013). The largest population corresponds to sites of processing and degradation and centres on a value of M close to 0, while the smaller population associated with higher M values corresponds to TSSs. The TSSs for *S. coelicolor* were on average associated with higher M values and lower A values (cf. Fig. 1A and B), which may reflect a lower proportion of the 5′ ends of primary transcripts being monophosphorylated *in vivo*. *E. coli* has an RNA pyrophosphohydrolase (Celesnik *et al*., 2007; Deana *et al*., 2008). The situation for *S. coelicolor* is not yet known, at least to our knowledge. For both organisms, nucleotide positions with M values above what was judged to be the upper boundary of the central cone of the population corresponding to processing and degradation sites (red line, Fig. 1A and B) were designated positions of possible TSSs. The positioning of the upper boundary was based on knowledge of the shape of cones produced by plotting two biological replicates of samples (before TAP treatment) against each other (data not shown). Positions within 8 nt of each other were assigned to the same TSS, as it is known that many promoters initiate transcription from a cluster of nucleotide positions (Salgado *et al*., 2006). These sites were then mapped against leading edges of transcription, which were determined independently via manual inspection of the global RNA-seq data, as described previously (Lin *et al*., 2013).

For *S. coelicolor* and *E. coli*, we identified 1147 and 728 sites, respectively, that were associated with leading edges of transcription as well as being enriched following TAP treatment (Class I). The higher number associated with *S. coelicolor* probably reflects its larger gene content (7.8 versus 4.3k genes). TSSs were identified for all types of functional RNA: mono- and poly-cistronic mRNAs, ribosomal RNAs, transfer RNAs and small RNAs (for examples, see below). For both organisms, we also identified, as described previously for *P. acnes* (Lin *et al*., 2013), sites that were enriched following TAP treatment, but not associated with leading edges of transcription (Class II), and others associated with leading edges, but not enriched (Class III). Although, Class I sites were assigned with the most confidence, being based on two criteria, all three classes contained TSSs that have been identified previously by others and recorded in RegTransBase for *S. coelicolor* (Cipriano *et al*., 2013) or RegulonDB for *E. coli* (Salgado *et al*., 2006; Mendoza-Vargas *et al*., 2009). This information has been included in our annotation of both *S. coelicolor* and *E. coli* sites (Tables S1 and S2 respectively). The probable origin of the different classes of TSS, which has been described previously by us (Lin *et al*., 2013), is outlined in the Discussion section.

### Leaderless mRNAs

A striking difference between *S. coelicolor* and *E. coli* that has been reported previously (Vockenhuber *et al*., 2011) is the prevalence of mRNAs that cannot be translated via the canonical Shine–Dalgarno (SD) interaction (Shine and Dalgarno, 1974; 1975[Bibr b140],[Bibr b141]) because they either lack or have a short 5′ leader. By mapping TSSs onto the annotated genomes, we identified 264 mRNAs with leaders shorter than 10 nt (classified here as leaderless, lmRNA) for *S. coelicolor*, but only five for *E. coli* (Table S3). While analysis of the gene ontology of our extended list of lmRNAs for *S. coelicolor* failed to identify a group with linked function, three of the five leaderless *E. coli* mRNAs were found to encode transcription regulators within prophage (Qin, Rac and e14). This extends the association between lmRNA and the regulators of mobile genetic elements in *E. coli*, which has been a major bacterial model for the study of ‘leaderless’ translation (Moll *et al*., 2002; Malys and McCarthy, 2011). The two best-studied lmRNAs in *E. coli* encode the cI repressor of bacteriophage lambda (Walz *et al*., 1976) and the TetR repressor of transposon Tn1721 (Baumeister *et al*., 1991). However, neither lambda nor Tn1721 are present in MG1655 (seq), which was used as the reference genome for this study. The translation of lmRNAs from several *Streptomyces* species has also been studied (Janssen, 1993).

**Fig. 2:**
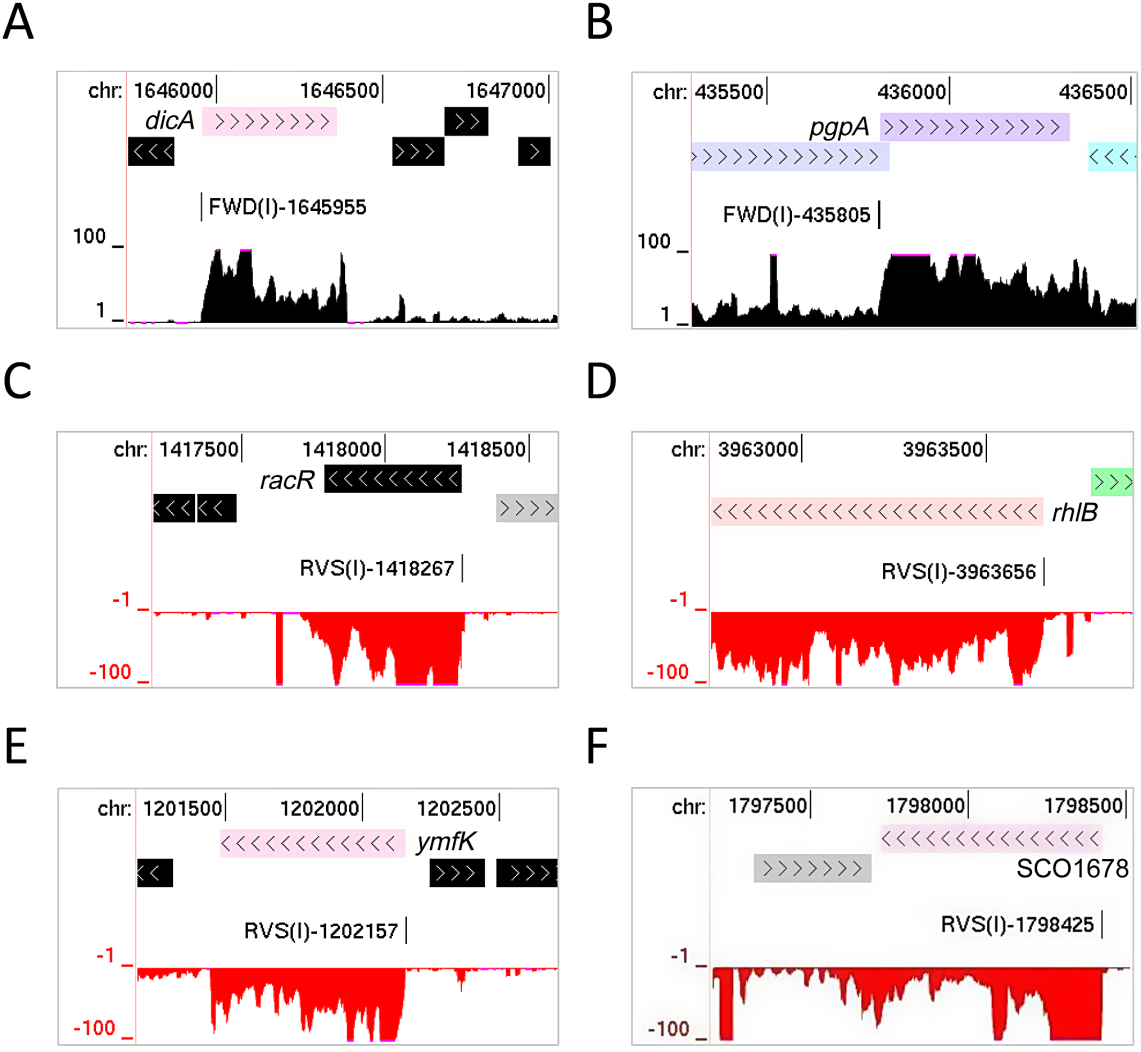
Examples of leaderless mRNAs. (A), (B), (C), (D) and (E) correspond to *E. coli* genes *dicA* (Qin prophage; b1570), *pgpA* (b0418), *racR* (Rac prophage; b1356), *rhlB* (b3780) and *ymfK* (e14 prophage; b1145) respectively. (F) corresponds to an unnamed paralogue (SCO1678) of the *S. coelicolor* gene *whiH*. The panels are modified screenshots from the UCSC Microbial Genome Browser (Chan *et al*., 2012). In each panel the tracks depict from top to bottom, the genome position, location of annotated genes, the positions of TSSs identified by the analysis of M-A scatterplots (Fig. 1), and the number of times each position on the corresponding strand was sequenced following fragmentation of the transcriptome (gRNA-seq). The numbers at the left of the RNA-seq tracks indicate the scale of the sequencing reads.

The two other *E. coli* mRNAs identified as being leaderless encode housekeeping proteins, phosphatidylglycerophosphatase A (PgpA) and the RhlB helicase. The former is involved in phospholipid biosynthesis (Lu *et al*., 2011), while the latter is a core component of the RNA processing and degradation machinery (Carpousis *et al*., 2009). Given the lack of an obvious connection of *pgpA* and *rhlB* to mobile genetic elements, we confirmed that lmRNAs were associated with these two genes using RNA ligase-mediated (RLM), reverse transcription (RT) PCR (Fig. S1). Moreover, sequence matches (uppercase text) to the consensus for the −10 box of *E. coli* vegetative promoters (Mulligan *et al*., 1984; Harley and Reynolds, 1987; Lisser and Margalit, 1993) were found just upstream of the start codons of *pgpA* and *rhlB* (TAgAcT and TATtcT respectively). The start of the RhlB protein has been confirmed by N-terminal sequencing (Py *et al*., 1996). The global RNA-seq data for the five *E. coli* lmRNAs and one of the many *S. coelicolor* lmRNAs are shown in Fig. 2. The *S. coelicolor* example is a paralogue of WhiH, which also has an lmRNA (Ryding *et al*., 1998). WhiH and other members of the GntR family have roles in controlling morphological development and secondary metabolism (Hillerich and Westpheling, 2006; Hoskisson *et al*., 2006; Persson *et al*., 2013). The possibility that the function of WhiH and other regulators is dependent on the leaderless status of the mRNA has not been investigated. It should be noted that here and elsewhere in this report the range of the global RNA-seq reads was restricted in genome-browser views to make it easier to determine the boundaries of transcription units. This can result in a block-like appearance. For the reverse strand, the RNA-seq data were given negative values and plotted in red instead of black. TSSs are represented by vertical lines and labelled according to strand, class and genome position.

### Processing within the 3′ end of 16S rRNA

**Fig. 3:**
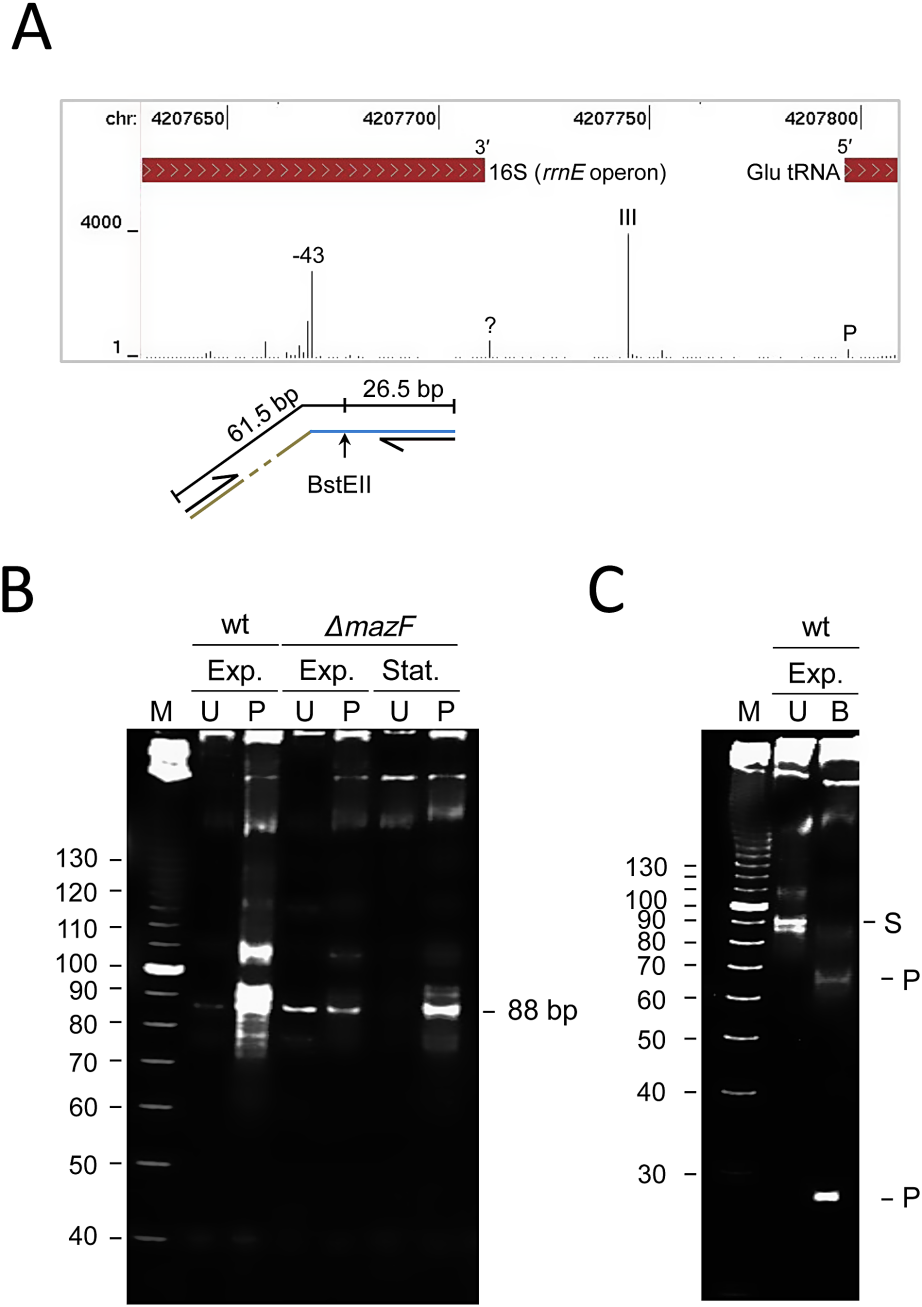
Processing at the −43 site of 16S rRNA.A. Differential RNA-seq data for the 3′ end of 16S rRNA. The screenshot is for the *rrnE* operon, which is representative of all seven rRNA operons in *E. coli*. The tracks from top to bottom show the genome position, location of the 3′ end of 16S rRNA and positions of processing sites as identified by differential RNA-seq in the absence of TAP treatment. The positions of the −43 site, sites of known cleavage by RNase III and P and a site of cleavage by an unknown RNase (labelled ‘?’) are indicated. The numbers at the left of the RNA-seq track indicates the scale of the sequencing reads. The schematic at the bottom of panel indicates the position of a BstEII site that was used to confirm the identity of an 88 bp amplicon produced by RLM-RT-PCR analysis of the −43 site (see B and C). The numbers indicate the sizes (bp) of the predicted products of cleavage at the BstEII site. It should be noted that the products have the equivalent of half a base-pair as BstEII generates 5 nt overhangs. Arrows indicate the position of primers used for PCR. Segments of the amplicon corresponding to the 5′ adaptor are drawn at an angle, while those corresponding to the 3′ end of 16S rRNA are drawn horizontally.B. RLM-RT-PCR analysis of RNA isolated from strain BW25113 (labelled wt) growing exponentially (labelled Exp.) and a congenic Δ*mazF* strain growing exponentially or in stationary phase (labelled Stat.). Prior to RLM-RT-PCR analysis, an aliquot of each sample was treated with T4 polynucleotide kinase (labelled P). Aliquots of untreated samples (labelled U) were also analysed. Labelling on the left indicates the sizes of molecular markers from Invitrogen (labelled M). The amplicon corresponding to the −43 cleavage site is indicated (labelled 88 bp) on the right. Products were analysed using a 10% polyacrylamide gel and stained with ethidium bromide. No amplicons were produced in the absence of reverse transcription (data not shown).C. Restriction enzyme analysis of amplicons produced from BW25113 RNA not treated with PNK. The substrate (labelled U) was incubated with BstEII and along with the resulting products (labelled B) analysed using gel electrophoresis as described in (B). Labelling on the right indicates the positions of resolvable substrate (labelled S) and products (labelled P).

Recently, it has been shown that *E. coli* lmRNAs, or at least those generated by 5′ processing under conditions of stress, are translated by specialized ribosomes from which the last 43 nt of the 3′ end of 16S rRNA have been removed endonucleolytically by MazF, the toxic component of a toxin-antitoxin (TA) system (Vesper *et al*., 2011). This region of 16S rRNA contains the anti-SD sequence and the binding site of S1 (Shine and Dalgarno, 1974; Lauber *et al*., 2012), a protein that augments the SD interaction (Sorensen *et al*., 1998). Interestingly, cleavage at the −43 site (numbered relative to the 3′ end of mature 16S rRNA) was identified by dRNA-seq (Fig. 3A) even though it is specific for 5′-monophosphorylated ends, not the 5′-hydroxylated ends generated by MazF and other mRNases associated with TA systems (Gerdes and Maisonneuve, 2012). Cleavage was also detected at a cluster of positions immediately upstream of the −43 site. The basis of cleavage at the −43 site and others in its vicinity was studied further using a modified RLM-RT-PCR approach in which efficient reverse transcription of short fragments was facilitated by adding a 3′ poly(A) tail and then using 5′-d(T)_10_(V) as the RT primer. A nested primer was used for the PCR step (Fig. 3B). The analysis included RNA isolated from BW25113 (wild-type) during exponential growth and a congenic Δ*mazF* strain during stationary phase as well as exponential growth. The RNA was also treated with polynucleotide kinase (PNK) to allow detection of 5′-hydroxylated ends.

For the sample isolated from the wild-type strain during exponential growth, an amplicon of 88 bp, the size expected for cleavage at the −43 site was detected, albeit of low abundance, without treating with PNK. This confirmed the generation of 5′-monophosphorylated ends at the −43 site. The possibility of cleavage by RNase E or RNase G is currently being explored. Following PNK treatment, amplicons corresponding to the −43 site and positions immediately upstream were readily detectable indicating that much of the cleavage within the 3′ end of 16S rRNA generates a downstream product with a 5′-hydroxyl group. Analysis of the RNA isolated from the Δ*mazF* strain during exponential growth produced a pattern similar to that of the wild-type strain in the absence of PNK treatment. However, in contrast, no substantial increase in the abundance or number of amplicons was detected following treatment with PNK. This suggested that in cultures in which the bulk of the cells are growing exponentially much of the cleavage that occurs at the −43 site and others in its vicinity are dependent on MazF. For RNA isolated from the Δ*mazF* strain during stationary phase, the detection of the 88 bp amplicon was dependent on PNK treatment. This suggests that during stationary phase the RNase that generates downstream products with a 5′-monophosphate group is not as active and an RNase other than MazF can cleave at the −43 site to produce downstream products with a 5′-hydroxyl group. In other words, processing of the 3′ end of 16S rRNA may represent a hub for the integration of signals from multiple regulatory paths. The identity of the amplicons corresponding to the −43 site and positions immediately upstream was confirmed by cutting the products of RLM-RT-PCR with BstEII (Fig. 3C). Fragments with mobility consistent with the expected sizes of 26.5 and ≥ 61.5 bp were produced; moreover, the abundance of the 26.5 bp fragment was significantly higher consistent with longer amplicons (see top of panel B) also being derived from the 3′ end of 16S rRNA. Processing within the equivalent region in *S. coelicolor* 16S rRNA was not detected; however, this was not unexpected as it has been shown for streptomycetes that specialized ribosomes capable of translating lmRNA can be produced by modification of the 16S rRNA (Kaberdina *et al*., 2009).

### Maturation of stable RNAs

**Fig. 4:**
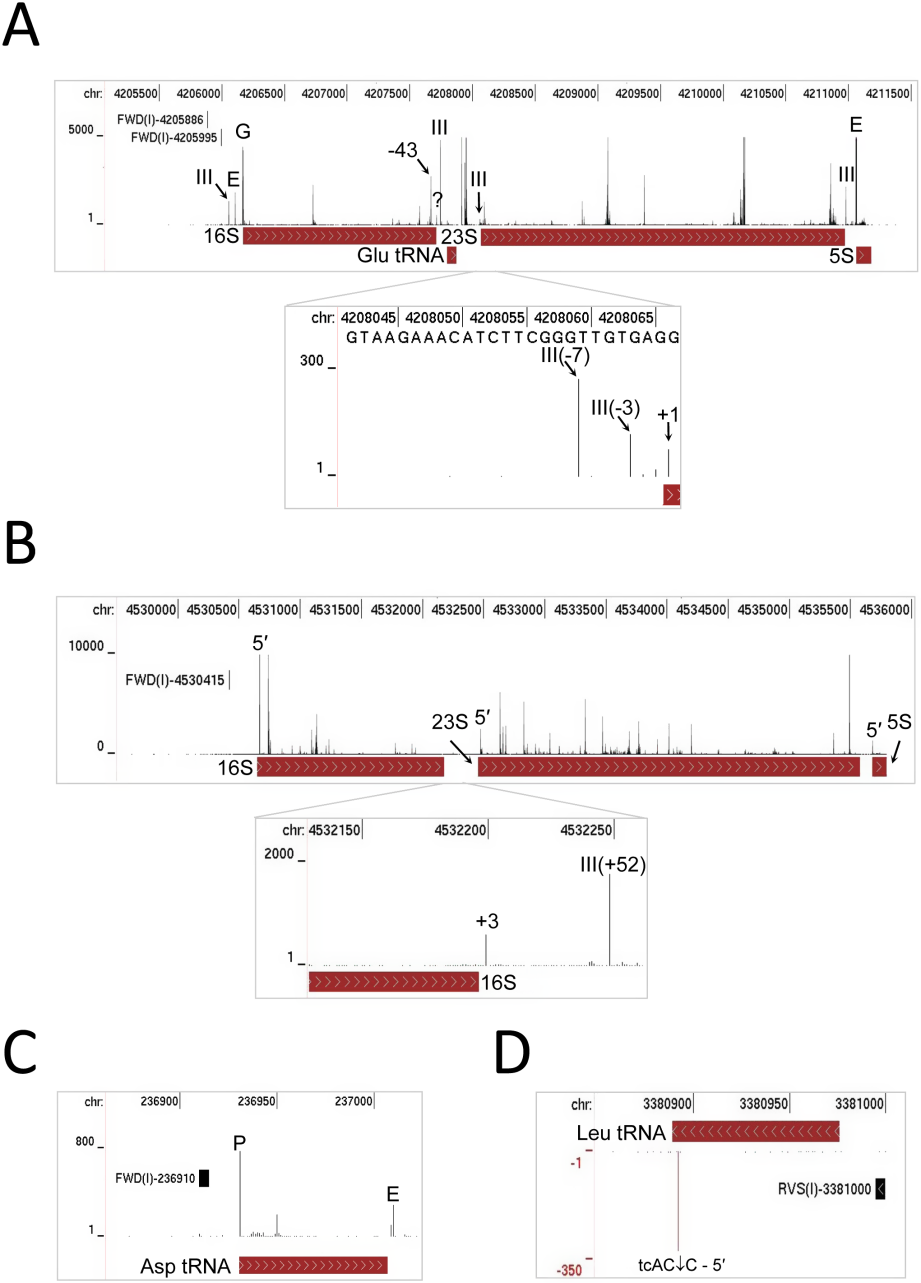
Maturation of stable RNAs.A. Annotated view of the *rrnE* operon of *E. coli*. The tracks from top to bottom show the genome position, differential RNA-seq reads in the absence of TAP treatment and the location of genes within the operon. The track containing the differential RNA-seq reads has been labelled to show the position of known cleavage sites and Class I TSSs. As Fig. 2, the labelling of the latter also indicates the stand and nucleotide position. Sites of cleavage by RNase III, E and G are labelled III, E and G respectively. The remainder of the labelling and numbering is as in Fig. 4. Inset shows positions at the 5′ end of 23S rRNA using a lower read scale.B. Annotated view of the *rrnA* operon of *S. coelicolor*. Tracks and numbering are as (A). Sites referred to in the text are labelled. Inset shows positions at the 3′ end of 16S rRNA using a lower read scale The 5′ and 3′ ends of the mature rRNAs are as determined using our global RNA-seq data.C. Processing at 5′ and 3′ end of *E. coli aspV* tRNA. Labelling as (B).D. Processing within the CCA at 3′ end of *S. coelicolor bldA* (Leu) tRNA. An arrow indicates the position of cleavage within the CCA motif.

Despite the central role of ribosomes in translation, little is known about the processing and degradation of its RNA in *S. coelicolor*. In contrast, the study of *E. coli* and *Bacillus subtilis* has revealed that mature ribosomal RNAs are produced via a series of nucleolytic steps involving several ribonucleases and that rRNA can be degraded in response to aberrant assembly of the ribosome or cellular stress (Deutscher, 2009). Remarkably, we were able to detect for *E. coli* most of the known endonucleolytic processing sites (Fig. 4A). It was initially considered that many of the processing sites might not be detected given the transitory nature of the corresponding intermediates and the fact that we had enriched the mRNA. However, we were able to detect all three of the known endonucleolytic steps involved in the maturation of the 5′ end of 16S rRNA, which are mediated by the combined action of RNases III, E and G (Young and Steitz, 1978; Li *et al*., 1999; Wachi *et al*., 1999).

RNase III cleavages were also detected that generate a 16S rRNA precursor with 33 extra residues at the 3′ end (Young and Steitz, 1978) and 23S rRNA precursors with three or seven extra residues at the 5′ end and 9 extra residues at the 3′ end (Bram *et al*., 1980). We also detected cleavage by an as yet unidentified ribonuclease that generates the 5′ end of mature 23S rRNA (Deutscher, 2009); a tight cluster of cleavages that produce the 5′ end of 5S rRNA, at least one of which is produced by RNase E (Misra and Apirion, 1979); and several sites internal to the rRNA, most of which are probably involved in degradation (Deutscher, 2009). With regard to the last point, while it is well established that the mature rRNAs within functional ribosomes are relatively stable during exponential growth (Deutscher, 2006), there is evidence that a proportion of the transcription of the rRNA operons terminates prematurely (Klumpp and Hwa, 2009). The corresponding transcripts being incomplete will undoubtedly need to be degraded rapidly to minimize the futile and perhaps detrimental association of r-proteins. Consistent with this notion is an earlier finding that rRNA fragments are a major component of the RNA associated with the *E. coli* RNA degradosome complex (Bessarab *et al*., 1998), which assembles around RNase E (Carpousis *et al*., 2009). Sites internal to the rRNA may also be involved in controlling the quality of rRNA (and ribosomes) (Jacob *et al*., 2013) or preventing the accumulation of rRNA beyond the level of r-proteins available for ribosome assembly (Norris and Koch, 1972; Gausing, 1977).

Sites at the precise 3′ ends of 23S and 5S RNA were not detected as these are generated primarily via 3′ exonucleolytic trimming (Li *et al*., 1998). However, we were able to detect processing at the precise 3′ end of 16S rRNA (most obvious in Fig. 3A). Thus, although much of the maturation at the 3′ end of this RNA appears to be via 3′ exonucleolytic trimming from the downstream RNase III site (Sulthana and Deutscher, 2013), an endonuclease, possibly the newly discovered YbeY RNase (Jacob *et al*., 2013), does appear to make a contribution (Li *et al*., 1999). The data shown is for the *rrnE* operon, but is representative of all seven *E. coli* operons. A high number reads were detected for cleavage sites downstream of the Glu tRNA (TTC). The origins of these cleavage sites are not known, as far as we are aware. We note that the reads associated with cleavages at the 5′ end of 23S rRNA (see inset, panel A) were substantially lower than those of cleavages at the 5′ end of 16S and 5S rRNA precursors. This could be due to differences in the efficiency of their cloning (as discussed later) or the actual abundance of the corresponding fragments being skewed as a result of mRNA enrichment or both. Nevertheless, the above analysis indicates that our dRNA-seq approach provides good coverage of the multiple steps involved in rRNA processing.

Analysis of the rRNA transcripts of *S. coelicolor* (Fig. 4B) revealed as many differences as similarities with *E. coli*. This was expected, as these organisms differ in their complement of ribonucleases. For example, *S. coelicolor* lacks RNase G, but has RNase J (SCO5745), an endonuclease with dual 5′ to 3′ exonucleolytic activity (Mathy *et al*., 2007; Bralley *et al*., 2014) that is absent in *E. coli*, but present in *B. subtilis* where one of its functions is to generate the 5′ end of 16S mature rRNA (Britton *et al*., 2007). Processing was detected at the 5′ ends of mature 16S, 23S and 5S rRNA, and at positions +3 and +52 relative to the mature 3′ end of 16S rRNA. The +52 site is within a segment complementary to another at the 5′ end of 16S rRNA. Thus, this site may correspond to cleavage by RNase III (AbsB, SCO5569), which is specific for double-stranded regions and has been shown to process rRNA in many bacteria (Nicholson, 2003) including *S. coelicolor* (Price *et al*., 1999). However, the product of the staggered cut within the complementary region at the 5′ end of 16S rRNA was not detected. This differs from what was found for *E. coli* and might reflect the closer co-ordination of subsequent 5′ processing steps, which could include 5′ to 3′ exonucleolytic processing by RNase J. No obvious processing sites were detected at the 3′ end of mature 23S or 5S rRNA suggesting that, as found for *E. coli*, the 3′ ends of these RNAs in *S. coelicolor* are generated primarily by 3′ exonucleolytic activity. Also similar to what was described above for *E. coli*, we detected a large number of sites internal to the functional regions of the mature rRNAs of *S. coelicolor*. The ribonucleases responsible for cleavage at these internal sites and those involved in processing the 5′ and 3′ ends can now be determined by analysing knockout mutants using fragment-specific approaches, such as those used to analyse *B. subtilis* rRNA processing (Redko and Condon, 2010). However, we would advocate the incorporation of dRNA-seq as it will provide a genome-wide view on the roles of *S. coelicolor* ribonucleases beyond rRNA processing.

Like rRNAs, tRNAs are produced with 5′ and 3′ segments that have to be removed in order for the molecule to become functional. All of the 86 tRNA genes in *E. coli* encode the 3′ CCA motif to which amino acids are attached, while 53 of the 65 tRNAs in *S. coelicolor* have this motif added post-transcriptionally because it is not encoded in the corresponding genes. Studies of tRNA processing in *E. coli* have led to a model in which the mature 5′ end is generated by the ubiquitous endonuclease RNase P, and the mature 3′ end via endonucleolytic cleavage a few nucleotides downstream followed by 3′ exonucleolytic trimming to the CCA motif. The maturation of the 3′ end can be mediated by tRNase Z (RNase BN), which has dual endo/3′ to 5′ exonucleolytic activity (Dutta and Deutscher, 2010; Dutta *et al*., 2012), or by the combined action of RNase E and 3′ to 5′ exonucleases, mainly RNases PH and T (Hartmann *et al*., 2009). Consistent with this model, processing sites were identified at the precise 5′ end and within a few nucleotides downstream of the 3′ end of *E. coli* tRNAs (Fig. 4C). The positions of the latter have been recorded (Table S4). However, it should be noted that cleavage at the 3′ end was not always detected, which is consistent with there being close coupling between steps, e.g. RNase P cleavage of a tRNA immediately downstream. Short fragments between processing sites would not have been cloned and sequenced by our approach (Lin *et al*., 2013).

In contrast to the situation in *E. coli*, we found that most of the 12 CCA-encoding tRNAs in *S. coelicolor* are cut *within* this motif between the Cs. This includes the *bldA* (Leu) tRNA (Fig. 4D), which is required for morphological development and accompanying secondary metabolism (Lawlor *et al*., 1987). The positions of cleavages within the CCA motif have been recorded (Table S4). It is possible that the *S. coelicolor* homologue of tRNA nucleotidyltransferase (SCO3896), which presumably adds CCA to tRNAs that are not transcribed with this motif (Cudny and Deutscher, 1986), may also be capable of recognizing partial CCA ends and adding only the residues that are missing. There is evidence that at least some tRNA nucleotidyltransferases, including the *E. coli* enzyme (Reuven *et al*., 1997), have the capability of repairing CCA (Betat *et al*., 2010). Such an activity in *S. coelicolor* would mean that cleavages within 3′ CCA triplets would not result in terminal inactivation of the tRNA. For several of the *S. coelicolor* tRNAs encoded without the CCA motif, we identified processing within a few nucleotides downstream of the 3′ end (Table S4). *S. coelicolor* has homologues of both RNase E (SCO2599) and tRNase Z (SCO2547), which could cut on the 3′ side of tRNAs. These cleavages presumably allow 3′ to 5′ exonucleolytic trimming of the tail prior to addition of the CCA by tRNA nucleotidyltransferase.

### The degradation and processing of mRNA

**Fig. 5:**
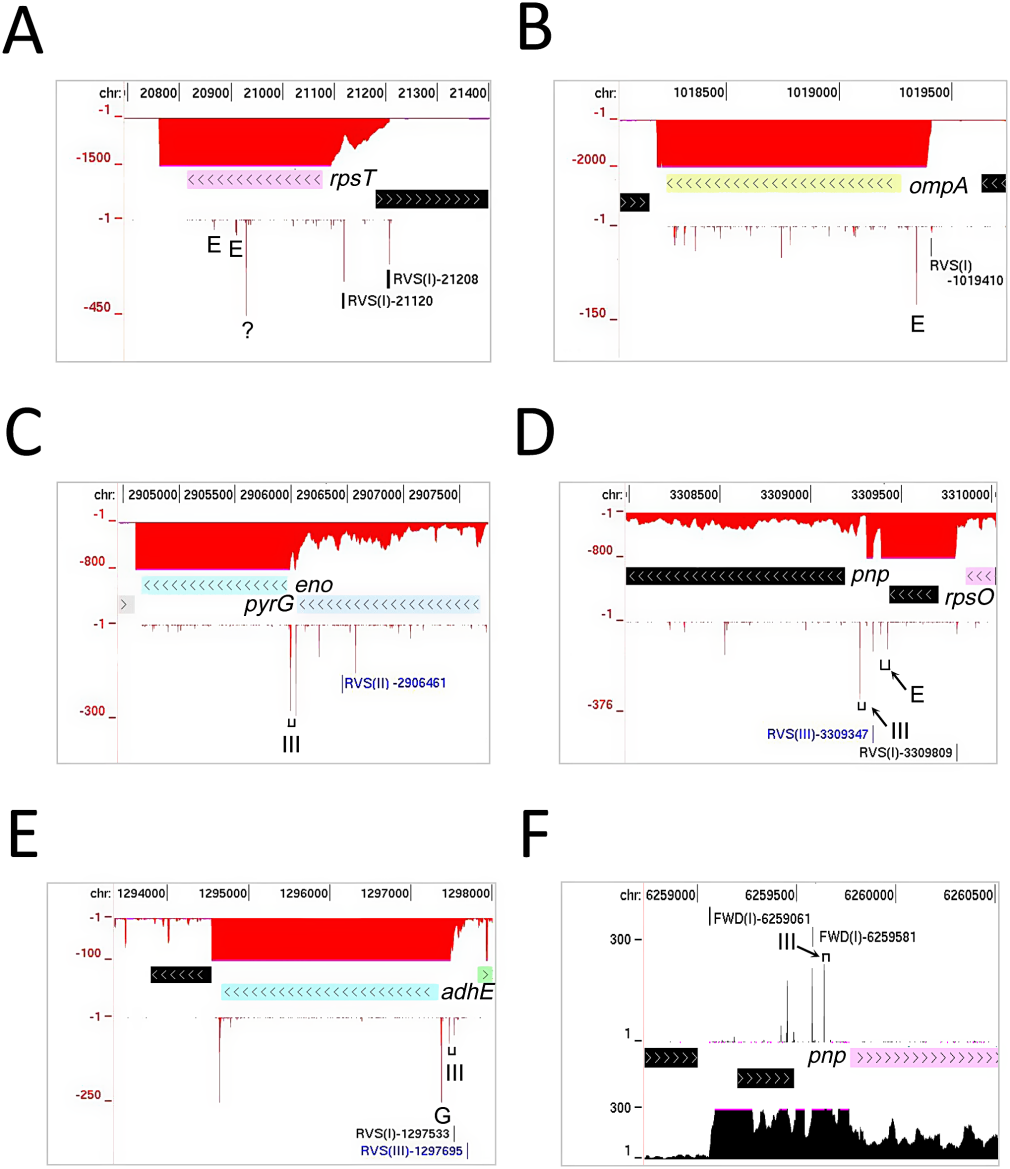
Cleavage sites within mRNAs. (A), (B), (C), (D) and (E) show annotated views of the *E. coli* *rpsT* (b0023), *ompA* (b0957), *eno* (b2779), *pnp* (b3165) and *adhE* (b1241) respectively. The tracks from top to bottom show the genome positions, gRNA-seq reads, gene locations and differential RNA-seq reads (in the absence of TAP treatment). The labelled RNase E sites in *rpsT* mRNA produce the previously described downstream products of 147 and 106 nt (Coburn and Mackie, 1998). An additional site referred to in the text is labelled with a question mark. The labelled RNase E site in the 5′ UTR of *ompA* mRNA is located just downstream of the 5′ stem-loop (Rasmussen *et al*., 2005). The labelled RNase E sites at the 3′ end of *rpsO* were identified previously as M2 and M (Regnier and Portier, 1986; Regnier and Hajnsdorf, 1991). (F) shows an annotated view of the *S. coelicolor* *pnp* (SCO5737) gene. Tracks, as the other panels, except that the order is reversed. TSSs and cleavage sites referred to specifically in the text are labelled. The RNase III site on the downstream side was detected using a lower range of reads. As Fig. 2, the gRNA-seq data for the forward strand are colour black and have positive values, while the reverse strand is coloured red and has negative values. TSSs are labelled according to the strand, class and nucleotide position.

With regard to mRNA, we were able to detect endonucleolytic sites known to be involved in both the degradation and processing of mRNA (Fig. 5). This included, but was not restricted to, RNase E sites that initiate the turnover of the mRNA of *rpsT* (Coburn and Mackie, 1998) (panel A) and *ompA* (Rasmussen *et al*., 2005) (panel B), and pairs of RNase III sites between *pyrG* and *eno* (Ow *et al*., 2003) (panel C), *rpsO* and *pnp* (Portier *et al*., 1987) (panel D) and in the 5′ leader of *adhE* (Aristarkhov *et al*., 1996) (panel E) that prime the downstream transcript for inactivation, in the case of *eno* and *adhE* by RNase G (Jourdan and McDowall, 2008). Two RNase E sites that span the Rho-independent terminator between *rpsO* and *pnp* were also detected (Regnier and Hajnsdorf, 1991) (panel D). The removal of the terminator, which has a relatively stable secondary structure, facilitates 3′ to 5′ exonucleolytic attack of the *rpsO* segment (Hajnsdorf *et al*., 1994). In addition, we detected previously uncharacterized sites within mRNAs that serve as models for understanding mRNA degradation, e.g. one internal to the coding region of *rpsT* mRNA (panel A) and another downstream of the RNase III sites in *adhE* that has been mapped previously, but not analysed (Aristarkhov *et al*., 1996) (panel E). A high number of reads were also associated with a previously undescribed site at the 3′ end of *adhE* (panel E), which might facilitate 3′ to 5′ exonucleolytic attack. Thus, our RNA-seq approach not only confirms, but extends knowledge of events controlling the activity and longevity of mRNA in *E. coli*. We also detected numerous processing sites within *S. coelicolor* mRNA. This included sites upstream of the coding segment of *pnp* (panel F), which are cut by RNase III to initiate a mechanism that autoregulates the cellular level of PNPase activity (Gatewood *et al*., 2011). Thus, although *S. coelicolor*, unlike *E. coli*, contains RNase J (SCO5745), an endoribonuclease with dual 5′ to 3′ exonuclease activity (Condon, 2010), the precise 5′ ends of the downstream products of endonucleolytic events can be detected. Moreover, the density of 5′ ends (number per transcribed kbp) is not significantly higher in *S. coelicolor* than in *E. coli* (data not shown) suggesting that 5′ to 3′ exonucleolytic decay does not dominate bulk mRNA degradation in the former, even though it contains a ribonuclease with 5′ to 3′ exonucleolytic activity.

### Identification of potential sRNAs

The mapping of the transcription data against the annotated genomes of *S. coelicolor* and *E. coli* revealed a number of short transcripts of high abundance relative to the background. A proportion of these transcripts mapped to palindromic sequences, which are the signatures of intrinsic transcriptional terminators (Peters *et al*., 2011) and other relatively stable stem-loop structures, e.g. those of repetitive extragenic palindromic (REP) elements in *E. coli* (Gilson *et al*., 1986; Higgins *et al*., 1988). Most of this group, many of which are located in 3′ UTRs (for examples, see Fig. S2A and B), probably only correspond to metastable decay intermediates and are not listed here as potential regulatory RNAs. There are over 650 REP elements in *E. coli* K-12 (Tobes and Ramos, 2005). It should be noted however that a number of reports indicate that some stable secondary structures in 3′ UTRs do correspond to functional sRNAs (Gossringer and Hartmann, 2012). We also identified cr-RNA associated with the CRISPR loci of *E. coli* (Fig. S2C). For both *S. coelicolor* and *E. coli*, the remaining group was found to contain all of the ubiquitous bacterial sRNAs (Fig. S3): 6S RNA, tmRNA, and the RNA components of RNase P and the Signal Recognition Particle (Storz *et al*., 2011). Moreover, of the 107 small RNAs in the remaining group for *E. coli* (Table S5), which included RNAs ranging from antisense regulator to riboswitches, at least 85% had been identified previously by other studies (e.g. Rivas *et al*., 2001; Wassarman *et al*., 2001; Vogel *et al*., 2003; Shinhara *et al*., 2011; Raghavan *et al*., 2012; Conway *et al*., 2014). This indicated that many of the 83 small RNAs identified for *S. coelicolor* should be easily verifiable by independent studies (Table S6). Indeed, subsequent analysis revealed that 51 had been identified by prior RNA-seq studies focussed on the small RNA component of *S. coelicolor* (Vockenhuber *et al*., 2011; Moody *et al*., 2013) or predicted by bio-computational approaches (Panek *et al*., 2008; Swiercz *et al*., 2008). This leaves 32 sRNAs that were previously undetected to our knowledge. Interestingly, 12 transcripts annotated previously as sRNAs (Vockenhuber *et al*., 2011) were found to extend into protein-coding sequences. The differences in annotation may simply reflect an increase in the sensitivity of detection and transcript coverage provided by the gRNA-seq approach adopted here. The comparison with the prior RNA-seq study of *S. coelicolor* has been summarized (Table S7).

**Fig. 6:**
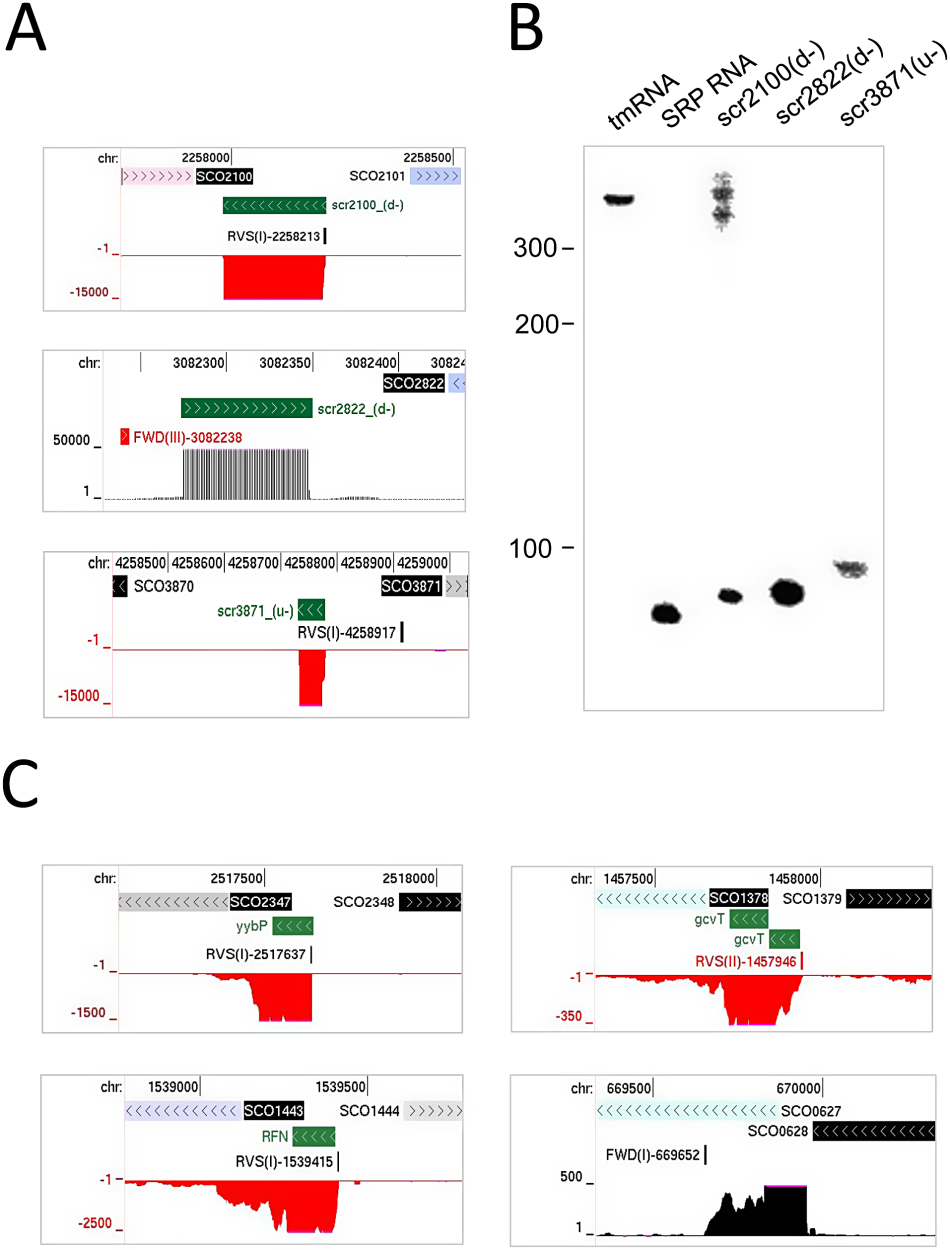
Northern blot analysis of *S. coelicolor* sRNAs. Labelling of sRNAs in parentheses indicates whether the sRNA is upstream (u) or downstream (d) of the nearest protein-coding gene and whether on the same (+) or opposite (−) strand.A. Annotated views of sRNAs downstream of SCO2100 on the opposite strand, downstream of SCO2822 on the opposite strand and upstream of SCO3871 on the opposite strand. The tracks from top to bottom show the genome positions, gene locations, TSSs and gRNA-seq reads.B. Northern blot analysis of sRNAs depicted in (A). The tmRNA and RNA component of the signal recognition particle were probed to provide controls. The expected sizes of the most abundant species of these controls as judged from gRNA-seq data were ∼ 400 and 80 nt respectively.C. Examples of active ribo-switching (attenuation) and a possible *cis*-encoded antisense RNA. The yybP element is reported to be pH responsive (Nechooshtan *et al*., 2009) and is found in a large number of bacteria (Barrick *et al*., 2004) including *E. coli* (Argaman *et al*., 2001), SCO2347 encodes an integral membrane protein. The gcvT element binds the amino acid glycine (Mandal *et al*., 2004), SCO1378 encodes glycine dehydrogenase. The RFN element (or FMN riboswitch) binds flavin mononucleotide (Serganov *et al*., 2009), SCO1443 encodes riboflavin synthase. SCO0627, the target of the putative *cis-*encoded asRNA, encodes a putative ATP-utilizing protein.

To verify the ability of our approach to detect small RNAs for *S. coelicolor*, we selected nine randomly (indicated in Table S6) from a list of sRNAs that at the time had not been identified experimentally. These were then analysed using northern blotting under stringent conditions that detect tmRNA and the RNA component of SRP, both of which are relatively abundant species. For three, scr2100(d−), scr2822(d+) and scr3871(u−), signals were detected readily (Fig. 6A and B). The sRNAs are labelled according to the nearest annotated gene, while the symbols in parenthesis indicate whether the RNA is downstream (d) or upstream (u) and transcribed from the same (+) or opposite strand (−). Moreover, the estimated sizes of the largest of the bands in each case corresponded reasonably well with the segment of highest abundance. For scr2822(d+) and scr3871(u−), these segments did not coincide with the predicted transcription start sites indicating a role for processing in their maturation. The presence of scr2100(d−) and scr2822d(+) were confirmed by Moody *et al*. (2013). For the other six, weaker signals could be detected, but against a background of hybridization after much longer exposure (data not shown). Despite this limitation, which is an inherent feature of northern blotting, we are confident that most of the small RNAs identified here for *S. coelicolor* by global RNA-seq are authentic. The list of 32 small RNAs that are listed here for *S. coelicolor* as being potentially novel include examples of riboswitches that appear to be actively reducing the levels of downstream transcripts and potential *cis*-encoded antisense RNAs (Fig. 6C).

**Fig. 7:**
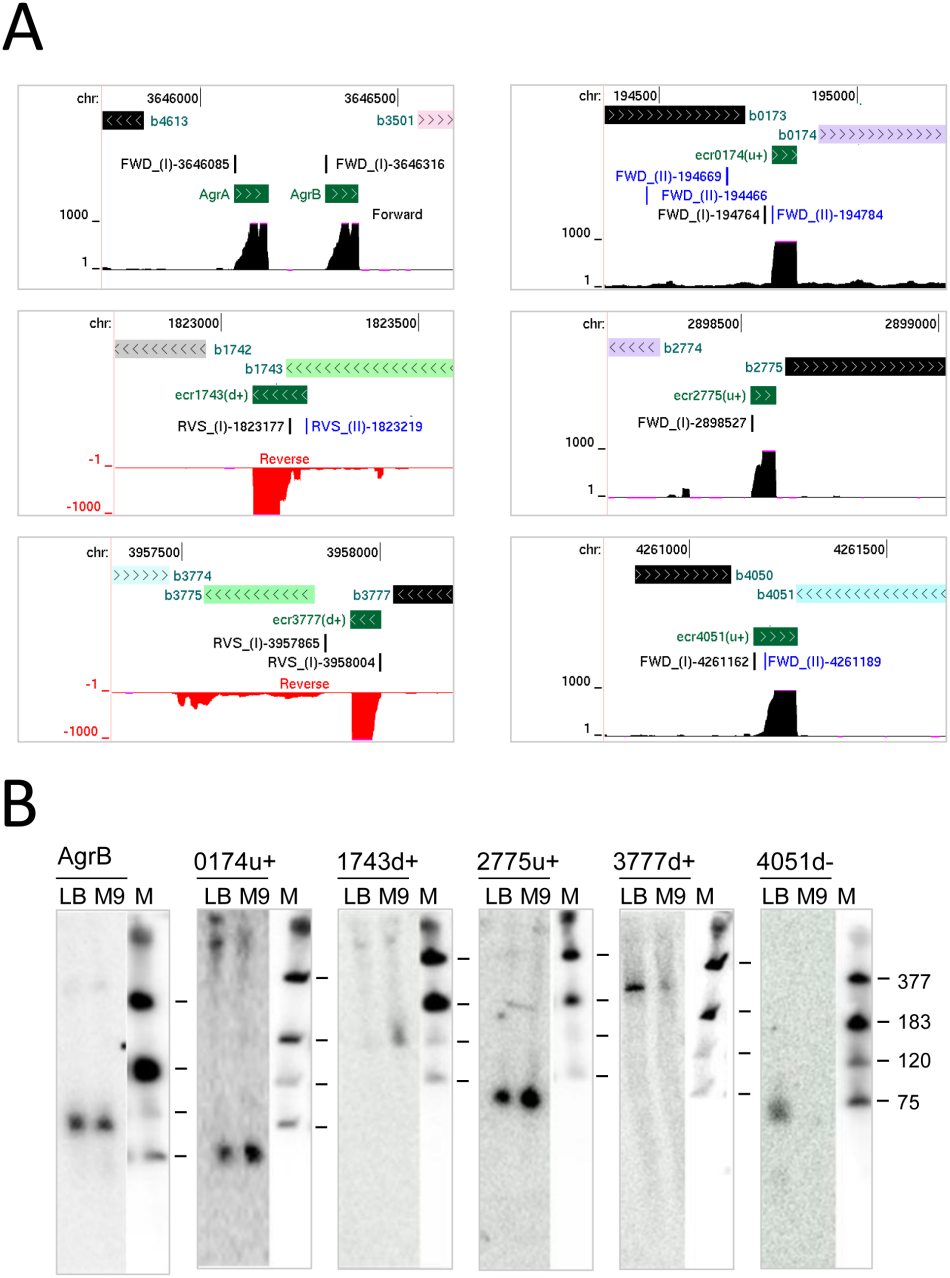
Examples of previously undetected RNAs in *E. coli*.A. Annotated views of the AgrA and AgrB small RNAs, the latter served as a positive control, and sRNAs encoded upstream of *ispU* (b0174) on the same strand, downstream of *spy* (b1743) on the same strand, upstream of *yqcE* (b2775) on the same strand, downstream of *yifN* (b3775) on the same strand, and downstream of *qor* (b4051) on the opposite strand. Labelling of sRNAs as Fig. 7. Tracks are as described in Fig. 2.B. Northern blot analysis of sRNAs depicted in (A). AgrB was probed to provide a positive control. For details of probes, see *Experimental procedures*.

We also undertook northern blot analysis of six sRNAs that at the time had not been described previously for *E. coli*, at least to our knowledge (indicated in Table S5). RNA, separate from that used for RNA-seq analysis, was used. As the expression of several sRNAs has been shown to be regulated by growth (Vogel *et al*., 2003), we isolated RNA from cells growing exponentially in M9 Glucose as well as LB. As a control, we included the analysis of AgrB sRNA, which is an RNA regulator of the SOS-related DinQ protein (Weel-Sneve *et al*., 2013). Positive results were obtained for five of the sRNAs and the AgrB control (Fig. 7). The actual abundance of all of the detected sRNAs, apart from the AgrB control, was dependent on the growth media. Two of the five *E. coli* sRNAs (see Table S5) have now been identified by an independent RNA-seq study that provides an unprecedented high-resolution view of bacterial operon architecture (Conway *et al*., 2014). The higher success with *E. coli* probably reflected the higher abundance of the transcripts that were selected and their lower GC-content. Thus, even in an organism that has been extensively studied (Storz *et al*., 2011), improved RNA-seq approaches can extend knowledge of sRNAs. A compendium of genome browser views of 10 *E. coli* sRNAs that are potentially novel and are in addition to the five that were identified by northern blotting is provided (Fig. S4).

### Transcription regulation and organization

The previous RNA-seq analysis of *S. coelicolor* (Vockenhuber *et al*., 2011) identified 193 TSSs for mRNA, 98 were associated in our analysis with readily detectable transcription downstream, and of these 79 were identified as Class I sites (see Table S1). The finding that not all TSSs identified previously by RNA-seq (Vockenhuber *et al*., 2011) or indeed conventional mapping approaches, such as S1 mapping and primer extension (recorded in RegTransBase) (Cipriano *et al*., 2013), were identified here is not surprising given the physiological and regulatory complexity of streptomycetes (Strohl, 1992; Chater, 2001; van Wezel and McDowall, 2011). There are also technical reasons that are discussed below. Nevertheless, the identification of 1147 Class I TSSs within the overall transcriptional landscape for *S. coelicolor* provides a much-improved platform for studying gene expression. For example, we identified several obvious transcription units that started within the 5′ portion of regions annotated as being coding sequences (Table S8) suggesting that the corresponding genes are actually shorter than previously thought. In support of this, we found using the BlastP track of the UCSC browser, which displays the results from running BLASTP for all predicted proteins in the genome against those from other prokaryotic species, that many homologues were predicted to be shorter, with regard to their N-terminal ends, than their *S. coelicolor* counterpart (data not shown). Thus, as others have indicated, RNA-seq data can aid the accurate prediction of the 5′ ends of protein-coding regions (Sallet *et al*., 2013).

**Fig. 8:**
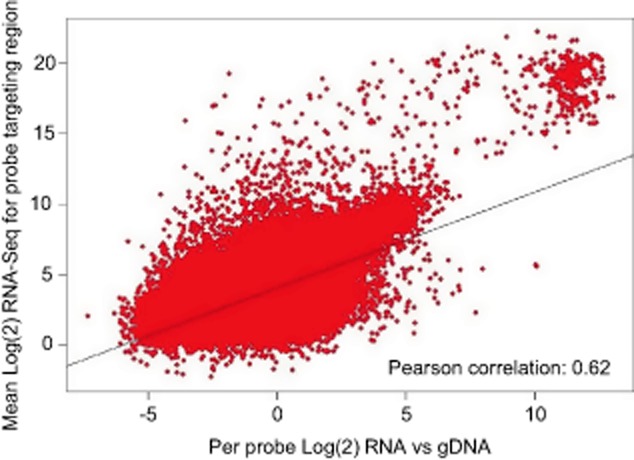
Comparison of global RNA-seq and microarray data for *S. coelicolor*. The mean RNA-seq reads for each base within the 60 bp region targeted by a microarray probe is directly compared with the microarray signal for the same probe target. Trendline calculated by the linear model fit within R.

In addition, our global RNA-seq data revealed many examples of operon structures that differ significantly from ‘Arkin Lab’ predictions (Price *et al*., 2005). Fortunately, automated processes are being developed that allow transcriptional units identified by RNA-seq to be mapped onto genome sequences (Sallet *et al*., 2013). Clearly accurate annotation is required for gene expression and regulation to be modelled closely at the level of the whole cell (Karr *et al*., 2012). In the context of gene expression, we also analysed transcript levels in our *S. coelicolor* RNA using high-density oligonucleotide-based microarrays and compared the level of hybridization (as a log2 ratio of signal obtained for the RNA sample to signal obtained for chromosomal DNA) against the number of global RNA-seq reads obtained over the regions covered by the oligonucleotide probes. This revealed reasonable congruence (Fig. 8) with a Pearson correlation coefficient of 0.63, supporting the view that the global RNA-seq approach adopted here, which does not require PCR, is suited to the study of bacteria with GC-rich genomes (Lin *et al*., 2013). The disparity that exists appears to be due at least in part to some regions that were not sequenced with high frequency producing significant hybridization signals. The latter may have resulted from a limited amount of cross-hybridization. Although the probes were experimentally validated and selected on the basis of sensitivity and selectivity (Bucca *et al*., 2009) and the microarrays have been used successfully (Lewis *et al*., 2010; Allenby *et al*., 2012; Swiatek *et al*., 2013; Rico *et al*., 2014), it is not possible to completely eliminate the effects of cross-hybridization (Wernersson *et al*., 2007; Mulle *et al*., 2010).

**Fig. 9:**
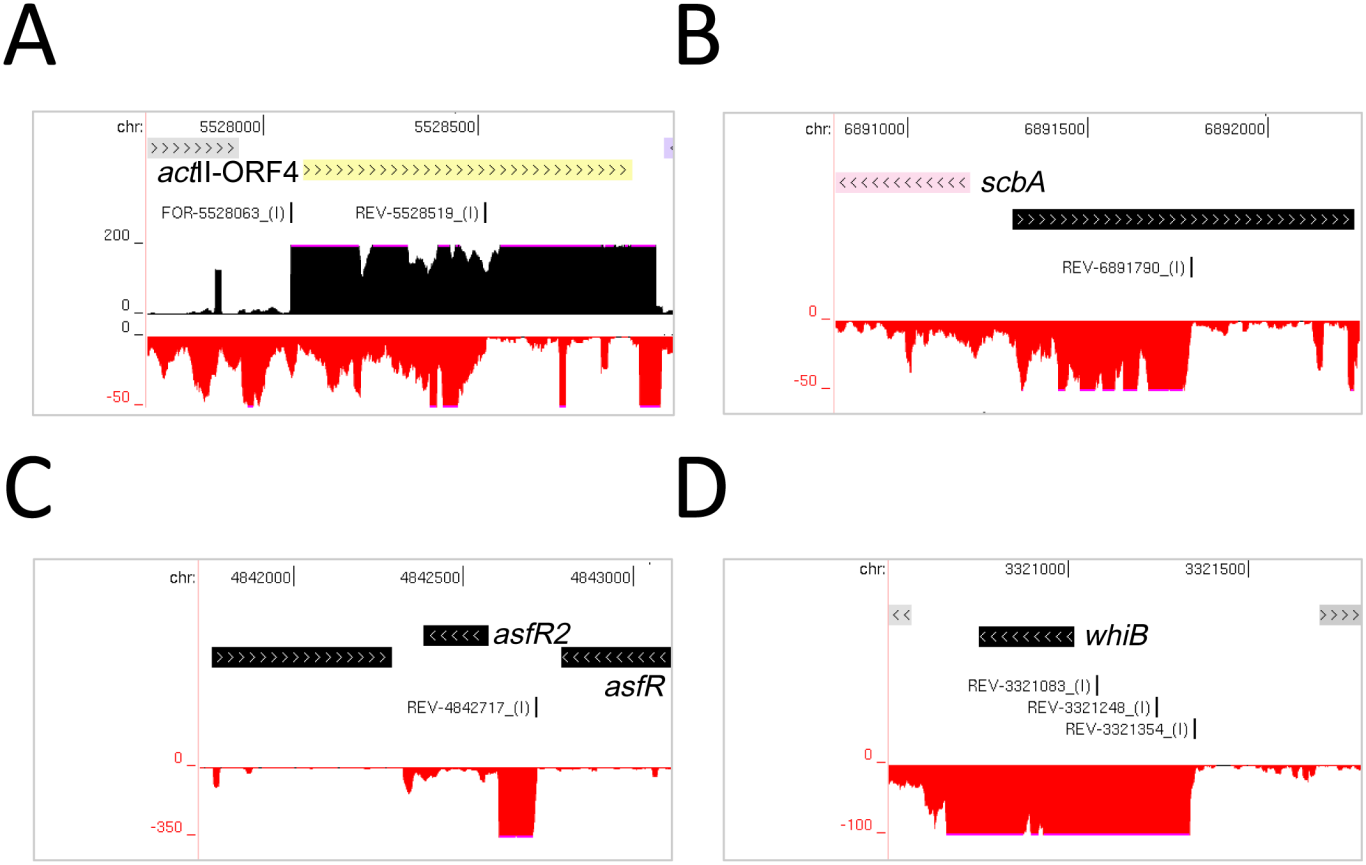
Examples of transcriptional complexity associated with key regulators of *S. coelicolor* metabolism and development. (A), (B), (C) and (D) shows annotated views of *act*II-ORFA, *scbA*, *afsR2*, and *whiB* respectively. Labelling is as in Fig. 2.

Consistent with the *S. coelicolor* RNA being isolated during exit from exponential growth, viewing of the transcriptome map revealed that the production of several secondary metabolites was primed. We readily detected transcription of *act*II-ORF4, *redZ* and *redD*, and *cdaR*, the cluster-situated regulators of actinorhodin (Act), undecylprodigiosin (Red) and the calcium-dependent antibiotic (CDA) respectively (Narva and Feitelson, 1990; Gramajo *et al*., 1993; Guthrie *et al*., 1998; Hojati *et al*., 2002), and *bldA*, the leucyl tRNA for the rare TTA codon (Lawlor *et al*., 1987), which is required for the effective translation of *act*II-ORF4 and *redD*, two of the aforementioned regulators. The level of transcription of *bldA* was similar to other tRNAs such as Val (CAC), Leu (TAG) and Arg (CCG) (data not shown). Transcription of the biosynthetic genes for Act, Red and CDA was barely detectable suggesting secondary metabolite production may have been awaiting the triggering of the stringent response (Strauch *et al*., 1991). Interestingly, however, in the case of *act*II-ORF4 we detected a long asRNA (> 500 nt) that conceivably has a regulatory role (Fig. 9A). Moreover, a long asRNA was also detected for *scbA* (Fig. 9B), which encodes the synthase of γ-butyrolactones (Hsiao *et al*., 2007; Kato *et al*., 2007), small signalling molecules that regulate secondary metabolism and morphological differentiation (Willey and Gaskell, 2011). A transcript antisense to *scbA* was also reported by Moody *et al*. (2013). Further unexpected features were identified for other key regulators. For example, we detected a previously unknown transcript in the intergenic region between *afsR2* (Vogtli *et al*., 1994) and *afsR* (Horinouchi *et al*., 1990), both of which are regulators of secondary metabolism. This transcript could have a discrete function or be the result of active riboswitching upstream of *afsR2* (Fig. 9C). We also detected additional transcriptional complexity for a number of transcription factor genes including *whiB*, a redox-sensitive transcription factor required for sporulation (Davis and Chater, 1992); in addition to the two promoters previously identified for *whiB* (Soliveri *et al*., 1992), we identified a strong promoter farther upstream (Fig. 9D).

**Fig. 10:**
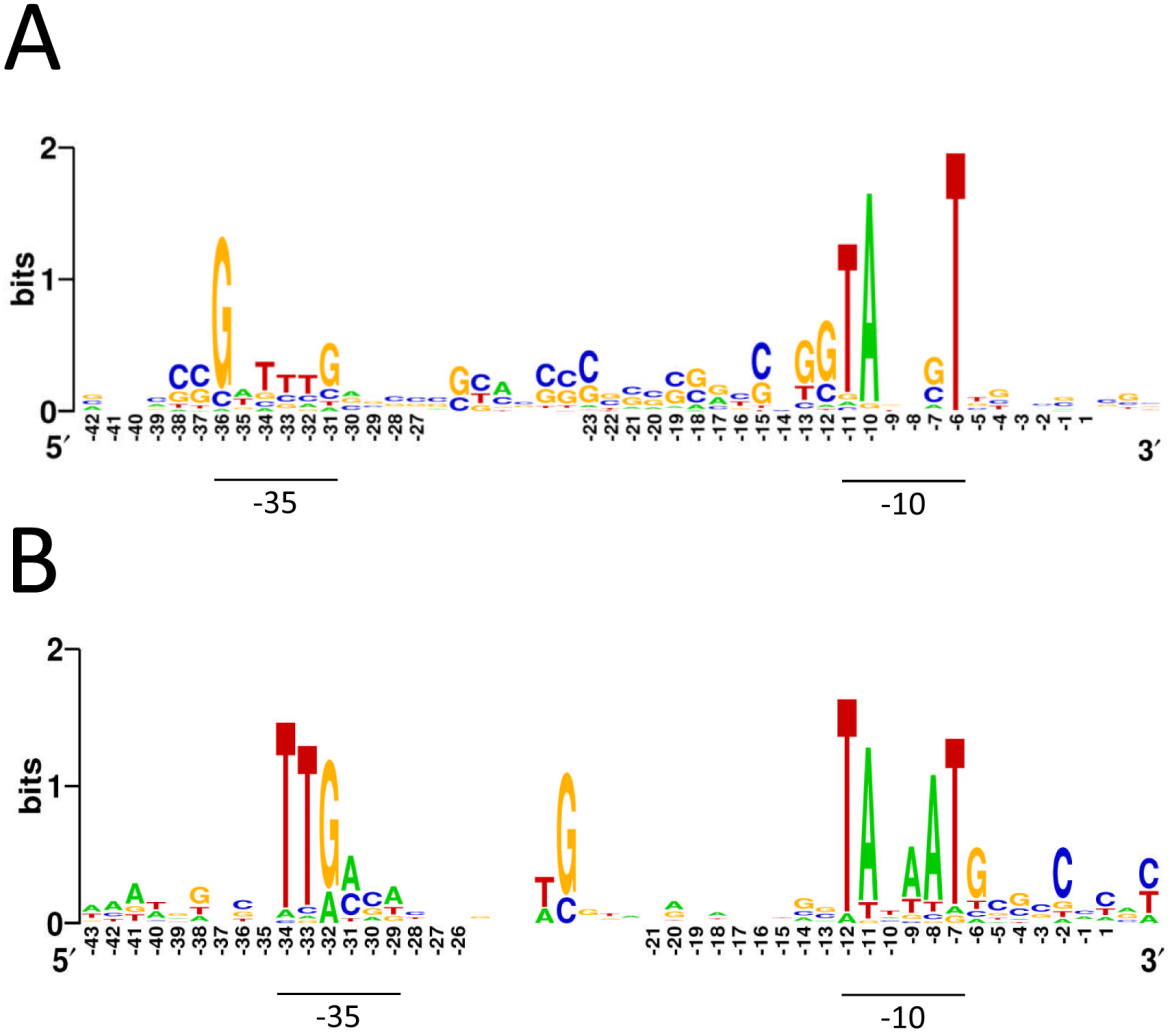
Conserved sequences in promoters associated with genes encoding the translational machinery. (A) and (B) are Weblogo representations of the promoters of *S. coelicolor* and *E. coli* respectively. The length of the space upstream of the −10 box was adjusted manually to maximize alignment of the −35 box as described previously (Lin *et al*., 2013). The combined height of nucleotide symbols shows the level of sequence conservation at a particular position, while the height of individual symbols within a stack of nucleotides indicates the relative frequency at that position. The nucleotide positions are numbered relative to the average position of TSSs to the point at which gaps were introduced to maximize the alignment. The numbering of position over the region of the −35 box is based on the average length of the spacer region.

Finally, knowing the nucleotide position of a control step narrows and simplifies the search for *cis*-regulatory elements (Lin *et al*., 2013). By way of illustration, we searched sequences immediately upstream of TSSs associated with genes of the translational machinery using MEME (Bailey *et al*., 2009). This was sufficient to reveal conserved hexanucleotide regions similar to the consensus sequences reported previously for the ‘vegetative’ promoters of streptomycetes (Strohl, 1992) and *E. coli* (Harley and Reynolds, 1987; Lisser and Margalit, 1993). Moreover, as has been reported recently for *P. acnes* (Lin *et al*., 2013), another actinomycete, the ‘−35′ box in *S. coelicolor* appears to be on average 2 to 3 bp further upstream from the TSS than its *E. coli* counterpart (Fig. 10). This means that a shared TTG motif, located in the 5′ half of the *E. coli* box and in the 3′ half of the *S. coelicolor* box, is on average in the same position relative to TSSs in both organisms. This may explain at least in part why many *Streptomyces* promoters function effectively in *E. coli* (Strohl, 1992). Alignment of the *E. coli* promoters also revealed the GC-rich discriminator region (Travers, 1980), which is located immediately downstream of the ‘−10’ box and is now known to facilitate regulation by the RNA polymerase-binding factors DksA and (p)ppGpp (Haugen *et al*., 2006). A similar sequence was not enriched in the *S. coelicolor* promoters.

## Discussion

By combining global and differential RNA-seq methodologies that should not be unduly affected by high GC content (Lin *et al*., 2013), we have obtained a genome-wide view of many factors that control *S. coelicolor* gene expression at the level of transcription initiation and beyond. For example, we identified lmRNAs (Fig. 2), key steps in the processing and degradation of rRNA, tRNA and mRNA (Figs 4 and 5), and small RNAs, including those that may be involved in attenuation-like switching mechanisms (Fig. 6). Many of the small RNAs identified in this study are novel (Table S6). Moreover, it is likely that more exists because the limited number of growth conditions studied to date (Vockenhuber *et al*., 2011; Moody *et al*., 2013) are unlikely to have captured the full physiological depth of *S. coelicolor* (Bentley *et al*., 2002). Our approach also identified over one thousand TSSs (Table S1) and transcription units, encompassing all classes of RNA. The global RNA-seq data can now be mapped onto the genome sequence (Sallet *et al*., 2013) to provide experiment-based annotation that should aid whole-cell modelling of, for example, regulatory modules (Castro-Melchor *et al*., 2010). The examination of individual regulators that are central to the control of secondary metabolism and morphological development has already revealed new layers of transcriptional complexity (for examples, see Fig. 9). Moreover, as was illustrated using promoters associated with the translational machinery (Fig. 10), knowing the nucleotide positions of key events in gene control can aid the identification of *cis*-regulatory sequences. This type of analysis can now be extended to other steps in the control of gene expression such as RNA processing and degradation and transcriptional termination. The RNA-seq data analysed here has been deposited in the GEO archive (Barrett *et al*., 2013).

The parallel analysis of *E. coli*, in addition to validating the approach, provided new insights into its gene regulation, despite it being one of the best-studied model organisms (Neidhardt, 1996). For example, it extended the known association between lmRNAs and repressors of mobile genetic elements in *E. coli*, while identifying lmRNAs associated with two housekeeping genes *pgpA* and *rhlB*. The genes encode the phospholipid biosynthesis enzyme, phosphatidylglycerophosphatase A (Lu *et al*., 2011), and the RhlB helicase, a core component of the RNA processing and degradation machinery (Carpousis *et al*., 2009) respectively (Fig. 2 and Fig. S1). We speculate that by being leaderless these mRNAs may be transcriptionally primed to be translated effectively during stress when translation is redirected by the activation of MazF, an RNase that removes the 5′-UTR regions from a selection of mRNAs, while initiating the degradation of others and producing specialized ribosomes that selectively translate lmRNAs (Vesper *et al*., 2011). Continued translation of the repressors of mobile genetic elements would ensure that additional demands are not placed on the cell when it is already stressed. Why uninterrupted translation of *pgpA* and *rhlB* mRNA would be required during stress is much less certain. With regard to RhlB, it is possible that its continued production ensures the rapid removal of inactivated mRNAs thereby reinforcing the selectivity of the translational machinery for the lmRNAs that ultimately determine the fate of the cell (Amitai *et al*., 2009). Alternatively, it could have a function beyond RNA processing and degradation in, for example, the remodelling of the specialized ribosomes or lmRNAs to ensure effective translation during cell stress. Roles for RNA helicases in ribosome remodelling are well established in at least eukaryotes (Martin *et al*., 2013). In both of these models, we imply that RhlB can function independent of continued production of the other degradosome components because the degradosome is either not required or the other components do not need to be replenished.

Related to the translation of lmRNAs, we identified endonucleolytic cleavage at the same site in 16S rRNA (Fig. 3) that is targeted by MazF to produce specialized ribosomes, even though the differential RNA-seq approach was specific for 5′-monophosphorylated ends (Lin *et al*., 2013), not the 5′-hydroxylated ends generated by MazF and other RNases associated with TA systems (Gerdes and Maisonneuve, 2012). As mentioned earlier, the possibility of cleavage by RNase E or RNase G is being explored. Further study of cleavage at this −43 site also revealed cleavage that produces downstream products with a 5′-hydroxyl group. Much of this additional cleavage could be attributed to MazF, even though the RNA sample was from cells growing exponentially (Fig. 3). Whether this reflects a low level of activity in all cells or a high level of activity in a subpopulation is not known. We also identified cleavage at the −43 site that produces downstream fragments with a 5′ hydroxyl independent of MazF during stationary phase (Fig. 3). Thus, processing of the 3′ end of 16S rRNA may represent a regulatory point at which the effects of several ribonucleases are integrated to control the specificity of the ribosome.

Processing at the equivalent of the −43 site in *S. coelicolor* 16S rRNA was not detected (Fig. 4), even though a significant proportion of its mRNAs are leaderless; however, this was not unexpected as it has been shown for streptomycetes that specialized ribosomes capable of translating lmRNA can be produced by modification of the 16S rRNA (Kaberdina *et al*., 2009). It would be interesting to determine whether *S. coelicolor* produces a molecule analogous to kasugamycin, a ribosome-interacting aminoglycoside originally isolated from *Streptomyces kasugaensis* that promotes 16S rRNA modification and thereby the translation of lmRNA (Schluenzen *et al*., 2006; Schuwirth *et al*., 2006; Kaberdina *et al*., 2009), and what role, if any, links the functions encoded by lmRNAs in *Streptomyces* spp. The substantial difference in the prevalence of lmRNAs between *S. coelicolor* and *E. coli* may also reflect the fact that the r-protein S1, which strongly promotes SD interactions in *E. coli* (Sorensen *et al*., 1998), is truncated at its C-terminus in *S. coelicolor* (SCO1998). Regardless of the underlying molecular biology, the results of this study add to the growing body of evidence that bacteria differ substantially in the extent to which they use lmRNAs (Nakagawa *et al*., 2010).

The parallel analysis of *E. coli* also identified 107 sRNA of which 13 were not described previously for *E. coli* to our knowledge (Table S5). We do not believe that this group of 13 sRNAs represents metastable decay intermediates as most are associated with TSSs in Class I. Nor do we believe that they represent artefacts of the RNA-seq as the majority of a selection probed by northern blotting were detected readily (Fig. 7). However, simply because a region is transcribed does not mean it has a function (Graur *et al*., 2013). Evidence for background (or pervasive) transcription on a genome scale has been obtained for several bacterial species (e.g. Lin *et al*., 2013) including *E. coli* (Raghavan *et al*., 2012). Therefore, an assessment of the impact on cell physiology of the plethora of small RNAs being discovered will require careful genetic analysis. Background transcription could explain at least a proportion of the TSSs in Class II, which are associated with TAP enrichment, but not an obvious step increase in transcription. However, verification of background transcription initiation will require a number of biological replicates and statistical analysis, as applied recently to *P. acnes* (Lin *et al*., 2013). TSSs associated with alternative promoters nested downstream of ones that produce a substantial increase in transcription would also have been assigned here to Class II. For *E. coli*, this represents about 15–20% of the promoters recorded in RegulonDB (Salgado *et al*., 2006) that have been verified by transcript-specific mapping (e.g. nuclease protection or primer extension assays) and associated in this study with obvious transcription extending downstream (data not shown).

The ability to detect sites involved in the initiation or mediation of rapid mRNA degradation using the differential RNA-seq approach described here (Fig. 5) offers a much-improved platform to further understanding of this key aspect of gene regulation. By extending the analysis to strains defective in key ribonucleases and their regulators, it should be possible to determine the impact of individual factors on a genome-wide scale, and to identify model transcripts, whose subsequent characterization should reveal the underlying molecular and structural biology. For example, by combining in *E. coli* mutations in RNase E (Mackie, 2013), the RppH pyrophosphohydrolase (Belasco, 2010) and Hfq, the chaperone of *trans*-acting antisense RNAs (De Lay *et al*., 2013), it should be possible to identify the targets and precise sites of RNase E cleavage that are mediated by antisense RNAs as a result of direct entry (Kime *et al*., 2010) or loading mediated via their de-pyrophosphorylated (i.e. monophosphorylated) 5′ ends (Bandyra *et al*., 2012). Moreover, by adapting our differential RNA-seq approach to include the ligation of a 3′ adaptor prior to fragmentation of the RNA and the subsequent addition of a 5′ adaptor, it should be possible to investigate degradation by 3′–5′ exonucleases.

While we advocate the use of TAP to differentiate nascent 5′ ends, we note that treatment with TEX, the 5′ to 3′ exonuclease specific for 5′-monophosphorylated transcripts, may offer increased discrimination of TSSs that cannot be identified by TAP enrichment *in vitro* as a consequence of efficient de-pyrophosphorylation by RppH (or similar) *in vivo*. TEX treatment would remove the majority of the de-pyrophosphorylated species thereby allowing those with nascent 5′ triphosphorylated ends to be detected. The mapping of TSSs could also be improved in *E. coli* at least by analysing strains deficient in RppH; however, this might not always be desirable given the resulting changes in gene expression and presumably cell physiology (Deana *et al*., 2008). Another improvement would be to remove the PCR step from the differential RNA-seq approach. As illustrated by the global RNA-seq approach, amplification is not required during the preparation of cDNA libraries (Mamanova *et al*., 2010). The removal of PCR would remove amplification bias. Related to PCR bias, we noted that most of the TSSs in Class III were associated with extremely low A (intensity) values (data not shown). Furthermore, analysis of the corresponding 5′ ends did not identify terminal stem-loops, which are known to block RNA pyrophosphohydrolases (Celesnik *et al*., 2007). Considered together, these results suggest that 5′ ends associated with leading edges of transcription, but not identified by dRNA-seq could not compete with others during the PCR step. This possibility is currently being investigated. About 25% of the *E. coli* promoters in RegulonDB (Salgado *et al*., 2006) that have been verified by transcript-specific mapping (e.g. nuclease protection or primer extension assays), and were associated in this study with obvious transcription extending downstream (data not shown), were assigned to Class III.

Combining Class I and III TSSs, we obtain numbers of 1598 and 1040 for *S. coelicolor* and *E. coli* respectively. These numbers are slightly lower than the number of proteins that have been detected for single conditions by proteomic approaches (Manteca *et al*., 2006; Rodriguez-Garcia *et al*., 2007); however, it should be remembered that many transcription units in both organisms contain multiple genes. In addition, the numbers of transcripts detected for both organisms will likely increase as more conditions are analysed. The inclusion of differential RNA-seq, regardless of its form, is crucial for accurate TSS assignment. Sites of processing, including many which are well characterized and documented (e.g. RNase P maturation of the 5′ end of tRNA), have been identified erroneously as transcriptional start sites by a previous RNA-seq analysis (Cho *et al*., 2009). Finally, the addition of a phosphorylation step to our differential RNA-seq approach would allow the identification of the cleavage sites of RNases that produce downstream products with 5′-hydroxyl group. This is likely to be particularly relevant to studies of suboptimal growth conditions under which such RNases, e.g. MazF (Vesper *et al*., 2011), are highly activated. The addition of a phosphorylation step would also allow the identification of positions with transcriptional start sites that are primed by nanoRNAs (Goldman *et al*., 2011; Nickels and Dove, 2011), which a recent study indicates tend to have a 5′-hydroxyl group (Vvedenskaya *et al*., 2012). The latter study also suggests that while nanoRNAs can alter gene expression, this class of sRNA are not absolutely required for transcription from individual promoters. Thus, the omission of a phosphorylation step in this study should not have prevented TSSs being identified.

## Experimental procedures

### Bacterial strains and their cultivation

*Streptomyces coelicolor* A3(2) strain M145 (Kieser *et al*., 2000) was obtained from the John Innes Centre (Norwich, UK), *E. coli* K-12 strain BW25113 (Baba *et al*., 2006) and its derivatives (Δ*mazF* and Δ*rppH*) from the Keio Collection (Yamagata, Japan), and *E. coli* K-12 strain MG1655 (seq) from the *E. coli* Genetic Stock Center (Yale). Both *S. coelicolor* and *E. coli* were cultivated with shaking (100–200 r.p.m.) in 250 ml Erlenmeyer flasks containing 50 ml of media. The flask for growing the former was fitted with a spring baffle to aid dispersed growth of the mycelia (Kieser *et al*., 2000). At the required stage of growth (see *Results*), a 1/8th volume of stop solution [95% (v/v) ethanol; 5% (v/v) phenol] was added to inhibit cell metabolism (Kime *et al*., 2008) and the cells were harvested by centrifugation. When necessary, cell pellets were stored frozen at −80°C.

### Isolation of bacterial RNA and transcriptome analysis

RNA for RNA-seq analysis was isolated from *S. coelicolor* grown in YEME broth (Kieser *et al*., 2000), as described previously for *P. acnes* (Lin *et al*., 2013), and from *E. coli* BW25113 grown in Luria–Bertani broth (Sigma), as described previously for this organism (Kime *et al*., 2008). To remove contaminating DNA, samples were treated with DNase I using conditions described by the vendor (Ambion) and extracted with phenol: chloroform as described previously (Kime *et al*., 2008). The concentration and integrity of RNA samples were determined using a NanoDrop™ 1000 spectrophotometer (Thermo Fisher Scientific) and agarose gel electrophoresis (Kime *et al*., 2008) respectively. Samples were then enriched for mRNA using *MICROBE*xpress™-Bacteria beads, as described by the manufacturer (Ambion).

Differential RNA-seq data were generated by Vertis Biotechnologie AG (Germany) as a service that included the construction of cDNA libraries before and after treatment with TAP, and the alignment of RNA sequence reads to the corresponding genome positions (Lin *et al*., 2013), which were retrieved from the NCBI database (Pruitt *et al*., 2007). For each genome position, the number of times it was the first nucleotide in sequence reads, i.e. associated with a 5′ end *in vivo*, was counted. This was done separately for each of the two libraries and the counts compared. It should be noted that as described previously (Lin *et al*., 2013) the 5′-sequencing adaptor was ligated to transcripts prior to fragmentation, thereby allowing the 5′ ends of both long and short transcripts to be detected. Global RNA-seq was performed at the Wellcome Trust Sanger Centre (Cambridge, UK) using a published methodology (Mamanova *et al*., 2010) and the sequences processed as described previously (Lin *et al*., 2013). After aligning the gRNA-seq reads to the genome, the number of times each genome position was present in a read irrespective of its position was counted. For both types of RNA-seq, the reference genomes for *S. coelicolor* A3(2) strain M145 and *E. coli* K-12 strain BW25113 were AL645882 and U00096.2 respectively. The RNA-seq data have been deposited in the GEO archive (Barrett *et al*., 2013) under accession numbers GSM1126846 and GSM1126845 respectively. The same RNA from *S. coelicolor* was also analysed using custom 105 000 × 60 mer whole-genome arrays manufactured by Agilent Technologies (Lewis *et al*., 2010). cDNA preparation, labelling, and hybridization was performed as described previously (Bucca *et al*., 1997; 2009[Bibr b22],[Bibr b23]). Two technical repeats of co-hybridizing the RNA with labelled genomic DNA were performed. A single Log_2_ RNA/gDNA value for each probe that passed the Agilent probe-quality criteria on at least one array was generated by averaging the global median normalized microarray signals. Corresponding Log_2_ ‘probe’ signals from the RNA-seq data were generated by averaging the signals of each base within the 60 bp sequence that a probe targets. RNA-seq coverage vectors for the forward and reverse strands were used to generate the data for forward-gene-targeting and reverse-gene-targeting probes respectively.

### RLM-RT-PCR analysis and northern blotting

The mapping of the 5′ ends of specific transcripts was done using RNA ligase-mediated RT-PCR as described previously (Kime *et al*., 2008). Unless otherwise stated, cDNA was synthesized using SuperScript® RT III (Invitrogen) with random hexamers (100 nM) and 200 ng of RNA template according to the instruction of the vendor of the reverse transcriptase. The PCR reaction was carried out using GoTaq® DNA polymerase (Promega) according to the vendor's instruction using cDNA diluted with RNase-free water as the template. The sequences of transcript-specific primers are provided in Table S9. Primers were designed with the assistance of Primer 3 software (Rozen and Skaletsky, 2000) and purchased from Eurofins MWG or Sigma. Two primers, RLM1 and RLM2, were used to bind the 5′ segment of cDNAs encoded by the 5′ adaptor. The sequences of these primers along with that of the 5′ adaptor are also provided in Table S9. The cDNA of the *pgpA* transcript was amplified using RLM1, while the cDNA of the remainder of the transcripts studied here were amplified using RLM2.

RNA for northern blotting was isolated as described for the RNA-seq analysis, with the exception that the mRNA was not enriched, from a second batch of cultures. *E. coli* K-12 MG1655 (seq) was grown in Luria–Bertani (Amresco) as well as M9 minimal media (Sigma) supplemented with glucose (0.4%, w/v) at 37°C with shaking (100 r.p.m.) until an OD_600_ of ∼ 0.5 at which point RNA was isolated as described previously (Kime *et al*., 2008). *S. coelicolor* was grown in YEME as described above. For each sample, an aliquot of 5–10 μg was mixed with an equal volume of 2× RNA-loading dye (New England BioLabs), denatured by incubation at 90°C for 90 s, chilled on ice, and analysed along with other samples by denaturing electrophoreses using a 6% sequencing-type gel [acrylamide : *bis*-acrylamide (29:1), 1× TBE, 7 M urea]. Fractionated RNA was electro-transferred to a Hybond-N^+^ membrane (Amersham) using 20× saline-sodium citrate (SSC) buffer at 11 V for 1 h, and subsequently fixed to the membrane by UV cross-linking.

Specific *E. coli* transcripts were probed using complementary oligonucleotides (see Table S9) labelled at their 5′ ends with ^32^P using T4 polynucleotide kinase (Thermo Scientific) and γ-^32^P-ATP (3000 Ci mmol^−1^, 10 mCi ml^−1^, 250 μCi, Perkin Elmer). The labelling reaction was carried out at 37°C for 30 min and stopped by the addition of EDTA, both as described by the vendor of the enzyme (Thermo Scientific). The radioactively labelled probes were precipitated by ethanol as described above, and resuspended in 20 μl of RNase-free water. The membrane was pre-hybridized with 3 ml of ULTRAhyb-Oligo Hybridization Buffer (Ambion) at 42^o^C for 30 min. Radiolabelled probe, which had been denatured by incubation at 90°C for 90 s and chilled on ice, was added to the hybridization tube. Hybridization was done at 42°C overnight. The membrane was washed twice with 20 ml of preheated washing buffer [5× SSC containing 0.5% (w/v) SDS] at 49°C for 30 min and exposed to Imaging Screen-K (Bio-Rad). The image was captured by Molecular Imager FX (Bio-Rad), and further processed using Quantity One (Bio-Rad) and GeneSys (Syngene) software.

Specific *S. coelicolor* transcripts were probed using riboprobes generated by *in vitro* transcription. The primers used to construct the templates for the riboprobes are listed in Table S9. Reactions of 20 μl contained 100 nM template (produce by PCR), 100 U T7 RNA polymerase (Invitrogen), 8 pmoles α-^32^P UTP (3000 Ci mmol^−1^, 10 mCi ml^−1^, 250 μCi; Perkin-Elmer), 5 μM UTP, 0.5 mM rATP, rGTP and rCTP, 1 U yeast inorganic pyrophosphatase (Sigma), 80 U RNaseOUT™ (Invitrogen) in 1× T7 RNA polymerase buffer: 40 mM Tris-HCl (pH 8.0), 8 mM MgCl_2_, 2 mM spermidine-(HCl)_3_, 25 mM NaCl, and 5 mM DTT. Reactions were incubated at 37°C for 3 h. Unincorporated nucleotides were removed by adding molecular biology-grade water (Sigma) to 50 μl and passing reaction products through G-25 spin columns, as per manufacturer's instructions (GE healthcare). Hybridizations were carried out overnight in the PerfectHyb™ Plus Hybridization buffer (Sigma-Aldrich) at 68°C followed by 2 × 20 min washes in high stringency buffer (0.5× SSC, 0.1% SDS) at 68°C. The hybridized blots were then exposed to a phosphoimager plate (Fuji) and read with a FLA 5000 scanner (Fuji).

### Data access

For both types of RNA-seq, the reference genomes for *S. coelicolor* A3(2) strain M145 and *E. coli* K-12 strain BW25113 were AL645882 and U00096.2 respectively. The RNA-seq data have been deposited in the GEO archive (Barrett *et al*., 2013) under accession numbers GSM1126846 and GSM1126845 respectively.

## Competing interests

The authors declare that they have no competing interests of a financial or non-financial nature.

## Authors’ contributions

KJM, Y-fL and DRA designed the transcriptome study. KJM, DRA and AHH performed the bulk of the RNA-seq analysis, with the assistance of MU. GB and EEL performed the *S. coelicolor* microarray analysis, supervised by CPS. DRA, AHH and OR-L analysed individual transcripts, supervised by LK and VRK. LM performed the global RNA sequencing. GPvW provided valuable input. KJM, DRA and AHH wrote the article. All authors read and approved the final manuscript.
