# Supplementary material for: A comparison of key aspects of gene regulation in Streptomyces coelicolor and Escherichia coli using nucleotide-resolution transcription maps produced in parallel by global and differential RNA sequencing
Source: Mol Microbiol. 2014 Oct 27;94(5):963–87. doi: 10.1111/mmi.12810 (PMC4681348; doi:10.1111/mmi.12810)
Supplement: Supplementary file 1 — Supporting Information [file mmi0094-0963-sd1.pdf]

A comparison of key aspects of gene regulation in *Streptomyces coelicolor* and *Escherichia coli* using nucleotide-resolution transcription maps produced in parallel by global and differential RNA-sequencing

David Romero A.[1], Ayad H. Hasan[1], Yu-fei Lin[1], Louise Kime[1], Olatz Ruiz-Larrabeiti[2], Mia Urem[5], Giselda Bucca[4], Lira Mamanova[6], Emma E. Laing[4], Gilles P. van Wezel[5], Colin P. Smith[4], Vladimir R. Kaberdin[2,3] and Kenneth J. McDowall[1*]

[1]*Astbury Centre for Structural Molecular Biology, School of Molecular and Cellular Biology, Faculty of Biological Sciences, University of Leeds, Leeds, LS2 9JT, UK*

[2]*Department of Immunology, Microbiology and Parasitology, University of the Basque Country UPV/EHU, Leioa, Spain*

[3]*IKERBASQUE, Basque Foundation for Science, 48011 Bilbao, Spain*

[4]*Department of Microbial & Cellular Sciences, Faculty of Health & Medical Sciences, University of Surrey, Guildford GU2 7XH, UK*

[5]*Institute of Biology, Sylvius Laboratories, Leiden University, Leiden, NL-2300 RA, Netherlands*

[6]*The Wellcome Trust Sanger Institute, Wellcome Trust Genome Campus, Hinxton, Cambridge CB10 1SA, UK*

Supplementary material:

Name	Strand	Left	Right	RegTransBase *	RNA sequencing **
FWD_(III)-6319	fwd	6319	6324		
RVS_(III)-28865	rvs	28860	28865		
FWD_(II)-29918	fwd	29918	29923		
RVS_(I)-31296	rvs	31291	31296		SCO0038-31296
RVS_(I)-39326	rvs	39321	39326		
FWD_(I)-44252	fwd	44252	44257		
RVS_(I)-45219	rvs	45214	45219		
FWD_(I)-66543	fwd	66543	66548		
FWD_(III)-77207	fwd	77207	77212		
RVS_(III)-77505	rvs	77500	77505		
FWD_(III)-82464	fwd	82464	82469		
FWD_(I)-122729	fwd	122729	122734		
FWD_(I)-122748	fwd	122748	122753		
FWD_(I)-145499	fwd	145499	145504		
FWD_(I)-150435	fwd	150435	150440		
FWD_(I)-156413	fwd	156413	156418		
RVS_(I)-156588	rvs	156583	156588		
RVS_(I)-158115	rvs	158110	158115		
FWD_(I)-158309	fwd	158309	158314		
FWD_(I)-158499	fwd	158499	158504		
FWD_(I)-158768	fwd	158768	158773		
RVS_(III)-161095	rvs	161090	161095		
FWD_(III)-161202	fwd	161202	161207		
RVS_(I)-164962	rvs	164957	164962		
FWD_(I)-166013	fwd	166013	166018		
RVS_(I)-168616	rvs	168611	168616		
RVS_(III)-170518	rvs	170513	170518		
FWD_(III)-173768	fwd	173768	173773	crtEIBVf-173765(fwd)	
RVS_(I)-182248	rvs	182243	182248	crtYTUr-182247(rvs)	
RVS_(I)-184798	rvs	184793	184798	litRr-184797(rvs)	
FWD_(I)-184865	fwd	184865	184870	litSABf-184865(fwd)	
RVS_(I)-190859	rvs	190854	190859		SCO0200-190858
RVS_(III)-191654	rvs	191649	191654		
RVS_(I)-195491	rvs	195486	195491		SCO0204-195490
FWD_(I)-197191	fwd	197191	197196		
FWD_(I)-199994	fwd	199994	199999		
RVS_(III)-200662	rvs	200657	200662		
FWD_(I)-204387	fwd	204387	204392		
RVS_(III)-205943	rvs	205938	205943		
FWD_(I)-206480	fwd	206480	206485		
RVS_(I)-214153	rvs	214148	214153		
FWD_(I)-223812	fwd	223812	223817		
FWD_(I)-230957	fwd	230957	230962		
FWD_(I)-256218	fwd	256218	256223		
RVS_(I)-293520	rvs	293515	293520		SCO0298-293519
FWD_(I)-323210	fwd	323210	323215		
RVS_(I)-324069	rvs	324064	324069		
RVS_(I)-327643	rvs	327638	327643		
FWD_(III)-393471	fwd	393471	393476		
FWD_(I)-395702	fwd	395702	395707		SCO0381-395702
RVS_(III)-424508	rvs	424503	424508		
FWD_(I)-427612	fwd	427612	427617		
RVS_(I)-430019	rvs	430014	430019	sapAr-430019(rvs)	
RVS_(III)-432613	rvs	432608	432613		
RVS_(III)-433680	rvs	433675	433680		
RVS_(I)-471265	rvs	471260	471265		
FWD_(I)-484318	fwd	484318	484323		
RVS_(I)-492072	rvs	492067	492072		

FWD_(I)-492858	fwd	492858	492863		
FWD_(III)-507547	fwd	507547	507552		
RVS_(III)-533800	rvs	533795	533800		
FWD_(I)-537545	fwd	537545	537550		SCO0504-537545
FWD_(I)-547809	fwd	547809	547814		
RVS_(I)-559145	rvs	559140	559145		SCO0527-559144
FWD_(III)-598727	fwd	598727	598732		
RVS_(I)-603841	rvs	603836	603841	furA-catCr-603840(rvs)	
FWD_(III)-638393	fwd	638393	638398		
FWD_(III)-647368	fwd	647368	647373		
FWD_(I)-651435	fwd	651435	651440		
FWD_(III)-653398	fwd	653398	653403		
FWD_(I)-669652	fwd	669652	669657		
RVS_(I)-673581	rvs	673576	673581		
FWD_(I)-677033	fwd	677033	677038		
FWD_(III)-681905	fwd	681905	681910		SCO0641-681905
RVS_(III)-711661	rvs	711656	711661		
FWD_(III)-711786	fwd	711786	711791		
RVS_(III)-712693	rvs	712688	712693		
FWD_(III)-717150	fwd	717150	717155		
RVS_(III)-721195	rvs	721190	721195		
RVS_(I)-726847	rvs	726842	726847		SCO0685-726846
RVS_(III)-743449	rvs	743444	743449		
FWD_(III)-743465	fwd	743465	743470		
FWD_(I)-743901	fwd	743901	743906		
RVS_(I)-745809	rvs	745804	745809		
RVS_(III)-764153	rvs	764148	764153		
FWD_(III)-767383	fwd	767383	767388		
FWD_(I)-768417	fwd	768417	768422		
FWD_(III)-769518	fwd	769518	769523		
FWD_(III)-772457	fwd	772457	772462		
RVS_(I)-773384	rvs	773379	773384		
RVS_(I)-776377	rvs	776372	776377		
RVS_(III)-779891	rvs	779886	779891		
FWD_(I)-786526	fwd	786526	786531		
FWD_(II)-786824	fwd	786824	786829		
FWD_(I)-789174	fwd	789174	789179		
RVS_(III)-789838	rvs	789833	789838		
RVS_(III)-806672	rvs	806667	806672		
FWD_(I)-806741	fwd	806741	806746		
RVS_(I)-809988	rvs	809983	809988		
FWD_(I)-815887	fwd	815887	815892		
FWD_(II)-827422	fwd	827422	827427		
RVS_(I)-835475	rvs	835470	835475		
FWD_(I)-838746	fwd	838746	838751		
RVS_(I)-841713	rvs	841708	841713		
FWD_(III)-843749	fwd	843749	843754		
RVS_(I)-850043	rvs	850038	850043		
FWD_(II)-862026	fwd	862026	862031		
FWD_(I)-866076	fwd	866076	866081		
RVS_(I)-870364	rvs	870359	870364		
RVS_(III)-883049	rvs	883044	883049		
RVS_(II)-892998	rvs	892993	892998		
FWD_(III)-899420	fwd	899420	899425		
FWD_(I)-904019	fwd	904019	904024		
RVS_(II)-915716	rvs	915711	915716		
RVS_(I)-919723	rvs	919718	919723		
RVS_(I)-945873	rvs	945868	945873		
RVS_(III)-950367	rvs	950362	950367		

FWD_(I)-960504	fwd	960504	960509	
RVS_(II)-962569	rvs	962564	962569	
FWD_(I)-973359	fwd	973359	973364	
RVS_(II)-976777	rvs	976772	976777	
FWD_(I)-988511	fwd	988511	988516	
FWD_(I)-988755	fwd	988755	988760	
RVS_(I)-991113	rvs	991108	991113	
RVS_(I)-993244	rvs	993239	993244	
RVS_(I)-1004998	rvs	1004993	1004998	
FWD_(III)-1005154	fwd	1005154	1005159	
RVS_(III)-1008697	rvs	1008692	1008697	
FWD_(I)-1008746	fwd	1008746	1008751	
RVS_(III)-1020577	rvs	1020572	1020577	
FWD_(III)-1023032	fwd	1023032	1023037	
FWD_(I)-1024446	fwd	1024446	1024451	SCO0973-1024446
FWD_(I)-1037861	fwd	1037861	1037866	
RVS_(I)-1043334	rvs	1043329	1043334	
FWD_(III)-1043351	fwd	1043351	1043356	
RVS_(III)-1044374	rvs	1044369	1044374	
RVS_(I)-1044601	rvs	1044596	1044601	
RVS_(III)-1045867	rvs	1045862	1045867	
FWD_(I)-1045890	fwd	1045890	1045895	
FWD_(I)-1051245	fwd	1051245	1051250	
RVS_(III)-1055438	rvs	1055433	1055438	
RVS_(I)-1057184	rvs	1057179	1057184	
RVS_(I)-1057259	rvs	1057254	1057259	
FWD_(I)-1058541	fwd	1058541	1058546	
FWD_(III)-1059936	fwd	1059936	1059941	
FWD_(I)-1062550	fwd	1062550	1062555	
FWD_(II)-1063164	fwd	1063164	1063169	
RVS_(III)-1069633	rvs	1069628	1069633	
RVS_(I)-1073220	rvs	1073215	1073220	
RVS_(II)-1074085	rvs	1074080	1074085	
FWD_(II)-1074142	fwd	1074142	1074147	
FWD_(I)-1078484	fwd	1078484	1078489	
FWD_(I)-1087637	fwd	1087637	1087642	
FWD_(I)-1092334	fwd	1092334	1092339	
FWD_(III)-1097279	fwd	1097279	1097284	
RVS_(III)-1119115	rvs	1119110	1119115	
RVS_(I)-1134122	rvs	1134117	1134122	
RVS_(I)-1142972	rvs	1142967	1142972	
RVS_(III)-1143607	rvs	1143602	1143607	
RVS_(III)-1144865	rvs	1144860	1144865	
FWD_(III)-1144997	fwd	1144997	1145002	
RVS_(I)-1147713	rvs	1147708	1147713	
RVS_(III)-1160062	rvs	1160057	1160062	
RVS_(III)-1161701	rvs	1161696	1161701	
FWD_(I)-1170662	fwd	1170662	1170667	
RVS_(I)-1180791	rvs	1180786	1180791	
FWD_(I)-1180851	fwd	1180851	1180856	
FWD_(I)-1193304	fwd	1193304	1193309	
RVS_(I)-1205670	rvs	1205665	1205670	
FWD_(III)-1209660	fwd	1209660	1209665	
RVS_(III)-1224160	rvs	1224155	1224160	
RVS_(I)-1225868	rvs	1225863	1225868	
RVS_(II)-1235080	rvs	1235075	1235080	
FWD_(I)-1258193	fwd	1258193	1258198	
RVS_(III)-1272567	rvs	1272562	1272567	
FWD_(I)-1277241	fwd	1277241	1277246	

FWD_(I)-1283231	fwd	1283231	1283236	SCO1212-1283231
FWD_(I)-1285400	fwd	1285400	1285405	
FWD_(I)-1286627	fwd	1286627	1286632	SCO1215-1286626
FWD_(I)-1287646	fwd	1287646	1287651	
FWD_(III)-1292643	fwd	1292643	1292648	
RVS_(I)-1305845	rvs	1305840	1305845	
FWD_(I)-1308749	fwd	1308749	1308754	
RVS_(I)-1311105	rvs	1311100	1311105	
RVS_(III)-1313720	rvs	1313715	1313720	
FWD_(I)-1319859	fwd	1319859	1319864	
RVS_(I)-1321208	rvs	1321203	1321208	
RVS_(I)-1327925	rvs	1327920	1327925	SCO1256-1327924
FWD_(I)-1328895	fwd	1328895	1328900	
RVS_(I)-1333704	rvs	1333699	1333704	
FWD_(III)-1361518	fwd	1361518	1361523	
FWD_(I)-1367543	fwd	1367543	1367548	
RVS_(I)-1379753	rvs	1379748	1379753	
RVS_(III)-1385350	rvs	1385345	1385350	SCO1307-1385349
FWD_(I)-1394271	fwd	1394271	1394276	
RVS_(I)-1402744	rvs	1402739	1402744	
FWD_(II)-1404380	fwd	1404380	1404385	
FWD_(III)-1411647	fwd	1411647	1411652	
RVS_(I)-1441016	rvs	1441011	1441016	
RVS_(III)-1442749	rvs	1442744	1442749	
FWD_(I)-1442944	fwd	1442944	1442949	
RVS_(I)-1443945	rvs	1443940	1443945	
FWD_(I)-1445963	fwd	1445963	1445968	
FWD_(III)-1451347	fwd	1451347	1451352	
FWD_(I)-1451590	fwd	1451590	1451595	
FWD_(I)-1452495	fwd	1452495	1452500	
FWD_(II)-1452555	fwd	1452555	1452560	
RVS_(III)-1457946	rvs	1457941	1457946	
RVS_(II)-1459823	rvs	1459818	1459823	
FWD_(I)-1460671	fwd	1460671	1460676	
FWD_(II)-1460713	fwd	1460713	1460718	
RVS_(I)-1461210	rvs	1461205	1461210	
RVS_(I)-1462960	rvs	1462955	1462960	
RVS_(I)-1463580	rvs	1463575	1463580	
RVS_(I)-1467808	rvs	1467803	1467808	
RVS_(I)-1473972	rvs	1473967	1473972	SCOr03-1473971
RVS_(I)-1474115	rvs	1474110	1474115	
FWD_(I)-1479786	fwd	1479786	1479791	
FWD_(III)-1483722	fwd	1483722	1483727	
FWD_(I)-1490293	fwd	1490293	1490298	
RVS_(I)-1497018	rvs	1497013	1497018	
RVS_(II)-1501731	rvs	1501726	1501731	
RVS_(I)-1510110	rvs	1510105	1510110	
FWD_(I)-1517331	fwd	1517331	1517336	
RVS_(I)-1518446	rvs	1518441	1518446	
RVS_(I)-1519427	rvs	1519422	1519427	
RVS_(III)-1519788	rvs	1519783	1519788	
RVS_(I)-1527054	rvs	1527049	1527054	
RVS_(I)-1536058	rvs	1536053	1536058	SCO1439-1536057
RVS_(I)-1539415	rvs	1539410	1539415	
RVS_(III)-1549276	rvs	1549271	1549276	
FWD_(I)-1549709	fwd	1549709	1549714	
RVS_(I)-1555615	rvs	1555610	1555615	SCO1457-1555614
RVS_(I)-1559888	rvs	1559883	1559888	
RVS_(I)-1561405	rvs	1561400	1561405	

RVS_(I)-1573431	rvs	1573426	1573431		
RVS_(I)-1580892	rvs	1580887	1580892		
RVS_(II)-1581475	rvs	1581470	1581475		SCO1479-1581474
RVS_(III)-1581940	rvs	1581935	1581940		
RVS_(I)-1592142	rvs	1592137	1592142		SCO1488-1592141
FWD_(II)-1592249	fwd	1592249	1592254		
RVS_(III)-1594295	rvs	1594290	1594295		
RVS_(I)-1596415	rvs	1596410	1596415		
RVS_(III)-1599369	rvs	1599364	1599369		
RVS_(I)-1606617	rvs	1606612	1606617		SCO1503-1606616
RVS_(I)-1609865	rvs	1609860	1609865		
RVS_(I)-1614202	rvs	1614197	1614202		
FWD_(III)-1615234	fwd	1615234	1615239		
RVS_(III)-1623790	rvs	1623785	1623790		
FWD_(I)-1632020	fwd	1632020	1632025		
FWD_(III)-1634498	fwd	1634498	1634503		
FWD_(I)-1636660	fwd	1636660	1636665		
RVS_(I)-1636877	rvs	1636872	1636877		
RVS_(III)-1638996	rvs	1638991	1638996		
RVS_(I)-1641034	rvs	1641029	1641034		
RVS_(I)-1642024	rvs	1642019	1642024		
RVS_(I)-1650186	rvs	1650181	1650186		
FWD_(I)-1650327	fwd	1650327	1650332	ssgBf-1650325(fwd)	
RVS_(I)-1652580	rvs	1652575	1652580		
RVS_(II)-1655153	rvs	1655148	1655153		
FWD_(III)-1656316	fwd	1656316	1656321		
RVS_(III)-1658669	rvs	1658664	1658669		SCO1550-1658666
FWD_(III)-1659151	fwd	1659151	1659156		
RVS_(I)-1666667	rvs	1666662	1666667		
RVS_(I)-1666843	rvs	1666838	1666843		
RVS_(I)-1672073	rvs	1672068	1672073		
FWD_(I)-1676193	fwd	1676193	1676198		
FWD_(III)-1681977	fwd	1681977	1681982		
FWD_(I)-1699196	fwd	1699196	1699201		
FWD_(I)-1703984	fwd	1703984	1703989		
RVS_(III)-1708379	rvs	1708374	1708379		
RVS_(III)-1709286	rvs	1709281	1709286		
RVS_(I)-1711835	rvs	1711830	1711835		
RVS_(I)-1712074	rvs	1712069	1712074		
FWD_(III)-1713303	fwd	1713303	1713308		
RVS_(III)-1720147	rvs	1720142	1720147		
FWD_(I)-1739096	fwd	1739096	1739101		
RVS_(I)-1744803	rvs	1744798	1744803		
RVS_(I)-1747894	rvs	1747889	1747894		
RVS_(I)-1749367	rvs	1749362	1749367		SCO1633-1749366
RVS_(I)-1751297	rvs	1751292	1751297		
RVS_(I)-1752160	rvs	1752155	1752160		
RVS_(I)-1755252	rvs	1755247	1755252		SCO1640-1755251
RVS_(II)-1756568	rvs	1756563	1756568		
RVS_(I)-1760200	rvs	1760195	1760200		
FWD_(II)-1761014	fwd	1761014	1761019		
RVS_(III)-1763988	rvs	1763983	1763988		
FWD_(I)-1763989	fwd	1763989	1763994		
FWD_(I)-1765246	fwd	1765246	1765251		
RVS_(I)-1766160	rvs	1766155	1766160		SCO1651-1766159
RVS_(III)-1768017	rvs	1768012	1768017		
RVS_(I)-1776418	rvs	1776413	1776418		SCO1657-1776417
FWD_(I)-1777741	fwd	1777741	1777746		
FWD_(I)-1782009	fwd	1782009	1782014		

RVS_(I)-1784337	rvs	1784332	1784337	
FWD_(I)-1789861	fwd	1789861	1789866	
FWD_(I)-1794076	fwd	1794076	1794081	
FWD_(III)-1797129	fwd	1797129	1797134	
RVS_(I)-1798425	rvs	1798420	1798425	
FWD_(I)-1805345	fwd	1805345	1805350	
FWD_(I)-1816391	fwd	1816391	1816396	
FWD_(II)-1816581	fwd	1816581	1816586	
RVS_(I)-1820484	rvs	1820479	1820484	
RVS_(I)-1835574	rvs	1835569	1835574	
RVS_(I)-1842204	rvs	1842199	1842204	
FWD_(I)-1843759	fwd	1843759	1843764	
FWD_(I)-1847564	fwd	1847564	1847569	
FWD_(I)-1850983	fwd	1850983	1850988	
FWD_(I)-1869233	fwd	1869233	1869238	
RVS_(III)-1873037	rvs	1873032	1873037	
FWD_(I)-1874625	fwd	1874625	1874630	
RVS_(I)-1877199	rvs	1877194	1877199	
RVS_(I)-1881959	rvs	1881954	1881959	
RVS_(I)-1883525	rvs	1883520	1883525	
RVS_(I)-1888602	rvs	1888597	1888602	
RVS_(I)-1892214	rvs	1892209	1892214	
RVS_(III)-1898451	rvs	1898446	1898451	
FWD_(I)-1898618	fwd	1898618	1898623	
RVS_(I)-1898703	rvs	1898698	1898703	
RVS_(III)-1904230	rvs	1904225	1904230	
RVS_(III)-1908396	rvs	1908391	1908396	
FWD_(I)-1908437	fwd	1908437	1908442	
RVS_(II)-1920591	rvs	1920586	1920591	
RVS_(I)-1922096	rvs	1922091	1922096	SCOr06-1922095
RVS_(I)-1922772	rvs	1922767	1922772	
FWD_(III)-1922882	fwd	1922882	1922887	
FWD_(I)-1924363	fwd	1924363	1924368	
RVS_(I)-1926167	rvs	1926162	1926167	
RVS_(I)-1926663	rvs	1926658	1926663	
RVS_(III)-1927605	rvs	1927600	1927605	
RVS_(I)-1928885	rvs	1928880	1928885	SCO1800-1928884
FWD_(III)-1933883	fwd	1933883	1933888	
RVS_(I)-1934196	rvs	1934191	1934196	
FWD_(I)-1936829	fwd	1936829	1936834	
FWD_(I)-1939007	fwd	1939007	1939012	
RVS_(I)-1940833	rvs	1940828	1940833	
RVS_(I)-1941432	rvs	1941427	1941432	
RVS_(III)-1942890	rvs	1942885	1942890	
FWD_(I)-1948418	fwd	1948418	1948423	
FWD_(III)-1950274	fwd	1950274	1950279	
FWD_(I)-1950867	fwd	1950867	1950872	
RVS_(III)-1952260	rvs	1952255	1952260	
RVS_(I)-1958194	rvs	1958189	1958194	
RVS_(III)-1965408	rvs	1965403	1965408	
RVS_(I)-1967490	rvs	1967485	1967490	
RVS_(I)-1969476	rvs	1969471	1969476	SCO1840-1969475
FWD_(III)-1969476	fwd	1969476	1969481	
FWD_(I)-1975651	fwd	1975651	1975656	
RVS_(I)-1995067	rvs	1995062	1995067	
FWD_(III)-1996959	fwd	1996959	1996964	
FWD_(I)-1998468	fwd	1998468	1998473	
RVS_(I)-1999398	rvs	1999393	1999398	
RVS_(III)-2006201	rvs	2006196	2006201	

FWD_(I)-2007027	fwd	2007027	2007032		
FWD_(III)-2007926	fwd	2007926	2007931		
RVS_(I)-2040933	rvs	2040928	2040933		
RVS_(I)-2044227	rvs	2044222	2044227		
RVS_(II)-2058457	rvs	2058452	2058457		
RVS_(I)-2059133	rvs	2059128	2059133		SCO1926-2059132
FWD_(I)-2060169	fwd	2060169	2060174		
RVS_(I)-2061290	rvs	2061285	2061290		
RVS_(I)-2066404	rvs	2066399	2066404		
FWD_(III)-2066590	fwd	2066590	2066595		
RVS_(III)-2081221	rvs	2081216	2081221		SCO1947-2081220
RVS_(I)-2081353	rvs	2081348	2081353		
RVS_(I)-2089857	rvs	2089852	2089857		
RVS_(III)-2097456	rvs	2097451	2097456		
FWD_(I)-2098289	fwd	2098289	2098294		
RVS_(I)-2104969	rvs	2104964	2104969		
RVS_(I)-2107200	rvs	2107195	2107200		
FWD_(I)-2108379	fwd	2108379	2108384		
FWD_(I)-2115934	fwd	2115934	2115939		
RVS_(I)-2120423	rvs	2120418	2120423		
RVS_(III)-2124706	rvs	2124701	2124706		
RVS_(I)-2128542	rvs	2128537	2128542		
FWD_(III)-2128658	fwd	2128658	2128663		
RVS_(I)-2130537	rvs	2130532	2130537		
FWD_(III)-2130680	fwd	2130680	2130685		
RVS_(I)-2132990	rvs	2132985	2132990		
RVS_(I)-2134822	rvs	2134817	2134822		
RVS_(I)-2134974	rvs	2134969	2134974		
RVS_(III)-2140897	rvs	2140892	2140897		
RVS_(III)-2144384	rvs	2144379	2144384		
FWD_(III)-2144607	fwd	2144607	2144612		
RVS_(II)-2145449	rvs	2145444	2145449		
RVS_(III)-2147688	rvs	2147683	2147688		
RVS_(I)-2150797	rvs	2150792	2150797		
RVS_(I)-2157774	rvs	2157769	2157774		
FWD_(III)-2165817	fwd	2165817	2165822		
RVS_(I)-2177604	rvs	2177599	2177604		
RVS_(I)-2186263	rvs	2186258	2186263		
RVS_(III)-2189613	rvs	2189608	2189613		
RVS_(I)-2190480	rvs	2190475	2190480		SCO2039-2190479
FWD_(III)-2194212	fwd	2194212	2194217		
RVS_(III)-2202561	rvs	2202556	2202561	hisDr-2202560(rvs)	
RVS_(I)-2207577	rvs	2207572	2207577		
RVS_(I)-2209845	rvs	2209840	2209845		
RVS_(II)-2215009	rvs	2215004	2215009		
RVS_(III)-2215231	rvs	2215226	2215231		
FWD_(III)-2215327	fwd	2215327	2215332		
FWD_(I)-2216800	fwd	2216800	2216805		
FWD_(I)-2220881	fwd	2220881	2220886		
RVS_(I)-2225156	rvs	2225151	2225156		
FWD_(I)-2226957	fwd	2226957	2226962		
RVS_(II)-2231963	rvs	2231958	2231963		
RVS_(I)-2232254	rvs	2232249	2232254		
RVS_(II)-2232420	rvs	2232415	2232420		
RVS_(I)-2233069	rvs	2233064	2233069		
FWD_(II)-2233577	fwd	2233577	2233582		
RVS_(I)-2235877	rvs	2235872	2235877		
RVS_(I)-2238195	rvs	2238190	2238195		
RVS_(I)-2248859	rvs	2248854	2248859		

FWD_(I)-2249741	fwd	2249741	2249746		
RVS_(I)-2255301	rvs	2255296	2255301		
RVS_(II)-2255358	rvs	2255353	2255358		
RVS_(II)-2255393	rvs	2255388	2255393		
RVS_(I)-2255440	rvs	2255435	2255440		SCO2097-2255438
RVS_(I)-2258213	rvs	2258208	2258213		
RVS_(I)-2261133	rvs	2261128	2261133		
RVS_(I)-2263247	rvs	2263242	2263247		
FWD_(I)-2264717	fwd	2264717	2264722		
FWD_(I)-2267216	fwd	2267216	2267221		
RVS_(I)-2270953	rvs	2270948	2270953		
RVS_(I)-2273426	rvs	2273421	2273426		SCO2115-2273425
FWD_(I)-2276540	fwd	2276540	2276545		
FWD_(II)-2278690	fwd	2278690	2278695		
FWD_(I)-2283605	fwd	2283605	2283610		
RVS_(I)-2286965	rvs	2286960	2286965	glkr-2286964(rvs)	
RVS_(I)-2287591	rvs	2287586	2287591	orf2-glkr-2287593(rvs)	
FWD_(I)-2298952	fwd	2298952	2298957		
RVS_(III)-2300687	rvs	2300682	2300687		
RVS_(I)-2308772	rvs	2308767	2308772		SCO2146-2308771
RVS_(III)-2309955	rvs	2309950	2309955		
FWD_(III)-2314405	fwd	2314405	2314410		
FWD_(II)-2318480	fwd	2318480	2318485		
FWD_(I)-2322389	fwd	2322389	2322394		
RVS_(I)-2324828	rvs	2324823	2324828		SCO2161-2324826
FWD_(III)-2325018	fwd	2325018	2325023		
RVS_(I)-2332889	rvs	2332884	2332889		
RVS_(I)-2332911	rvs	2332906	2332911		
RVS_(I)-2333070	rvs	2333065	2333070		
RVS_(III)-2333089	rvs	2333084	2333089		
FWD_(I)-2333148	fwd	2333148	2333153		
FWD_(III)-2342349	fwd	2342349	2342354		
FWD_(I)-2344238	fwd	2344238	2344243		
RVS_(I)-2344488	rvs	2344483	2344488		
FWD_(III)-2347731	fwd	2347731	2347736		
FWD_(I)-2354572	fwd	2354572	2354577		
FWD_(III)-2360873	fwd	2360873	2360878		
FWD_(I)-2361877	fwd	2361877	2361882		
FWD_(I)-2363038	fwd	2363038	2363043		
FWD_(I)-2363402	fwd	2363402	2363407		
RVS_(I)-2364685	rvs	2364680	2364685		
FWD_(III)-2364762	fwd	2364762	2364767		
FWD_(I)-2364831	fwd	2364831	2364836		
FWD_(III)-2366433	fwd	2366433	2366438		SCO2199-2366432
FWD_(I)-2367728	fwd	2367728	2367733		
FWD_(I)-2370829	fwd	2370829	2370834		
RVS_(I)-2371385	rvs	2371380	2371385		
FWD_(I)-2376423	fwd	2376423	2376428		
RVS_(II)-2380826	rvs	2380821	2380826		
FWD_(I)-2387313	fwd	2387313	2387318		
RVS_(II)-2392655	rvs	2392650	2392655		
FWD_(I)-2401747	fwd	2401747	2401752		
FWD_(I)-2401778	fwd	2401778	2401783		
FWD_(III)-2412697	fwd	2412697	2412702		
RVS_(III)-2419916	rvs	2419911	2419916		
RVS_(I)-2423077	rvs	2423072	2423077		
FWD_(I)-2423246	fwd	2423246	2423251		
FWD_(III)-2430315	fwd	2430315	2430320		
RVS_(II)-2431423	rvs	2431418	2431423		

RVS_(I)-2449562	rvs	2449557	2449562	
RVS_(I)-2449574	rvs	2449569	2449574	
RVS_(II)-2450090	rvs	2450085	2450090	
RVS_(I)-2463314	rvs	2463309	2463314	
FWD_(III)-2474063	fwd	2474063	2474068	
FWD_(I)-2485358	fwd	2485358	2485363	
FWD_(I)-2491687	fwd	2491687	2491692	
RVS_(III)-2506738	rvs	2506733	2506738	
FWD_(I)-2508321	fwd	2508321	2508326	
FWD_(I)-2510090	fwd	2510090	2510095	
RVS_(I)-2517637	rvs	2517632	2517637	
RVS_(III)-2523283	rvs	2523278	2523283	
FWD_(III)-2524296	fwd	2524296	2524301	
RVS_(I)-2533548	rvs	2533543	2533548	
RVS_(I)-2537964	rvs	2537959	2537964	
RVS_(I)-2538226	rvs	2538221	2538226	
RVS_(I)-2539214	rvs	2539209	2539214	
FWD_(III)-2539397	fwd	2539397	2539402	
RVS_(III)-2539432	rvs	2539427	2539432	
RVS_(III)-2544618	rvs	2544613	2544618	
RVS_(III)-2545701	rvs	2545696	2545701	
FWD_(II)-2546758	fwd	2546758	2546763	
FWD_(I)-2551643	fwd	2551643	2551648	
FWD_(III)-2556410	fwd	2556410	2556415	
RVS_(I)-2563831	rvs	2563826	2563831	
RVS_(III)-2565976	rvs	2565971	2565976	
FWD_(I)-2600215	fwd	2600215	2600220	
FWD_(II)-2602848	fwd	2602848	2602853	
FWD_(II)-2611531	fwd	2611531	2611536	
FWD_(I)-2613285	fwd	2613285	2613290	
RVS_(II)-2619474	rvs	2619469	2619474	
FWD_(III)-2625429	fwd	2625429	2625434	
FWD_(II)-2645607	fwd	2645607	2645612	
RVS_(I)-2647034	rvs	2647029	2647034	
FWD_(III)-2647273	fwd	2647273	2647278	
RVS_(III)-2647346	rvs	2647341	2647346	
FWD_(II)-2647362	fwd	2647362	2647367	
FWD_(I)-2647782	fwd	2647782	2647787	
FWD_(I)-2649323	fwd	2649323	2649328	
RVS_(III)-2659471	rvs	2659466	2659471	
FWD_(III)-2666878	fwd	2666878	2666883	
FWD_(I)-2690336	fwd	2690336	2690341	
RVS_(I)-2699585	rvs	2699580	2699585	
RVS_(II)-2703150	rvs	2703145	2703150	
RVS_(I)-2703897	rvs	2703892	2703897	
FWD_(III)-2704003	fwd	2704003	2704008	
FWD_(I)-2704834	fwd	2704834	2704839	
FWD_(I)-2706771	fwd	2706771	2706776	
RVS_(III)-2708924	rvs	2708919	2708924	
FWD_(III)-2709440	fwd	2709440	2709445	
RVS_(II)-2712843	rvs	2712838	2712843	
RVS_(I)-2723824	rvs	2723819	2723824	
RVS_(I)-2723947	rvs	2723942	2723947	
RVS_(I)-2727365	rvs	2727360	2727365	SCO2528-2727364
RVS_(I)-2727404	rvs	2727399	2727404	
FWD_(I)-2730367	fwd	2730367	2730372	
FWD_(I)-2736606	fwd	2736606	2736611	
RVS_(I)-2751451	rvs	2751446	2751451	
RVS_(II)-2754880	rvs	2754875	2754880	

RVS_(III)-2756142	rvs	2756137	2756142		
FWD_(III)-2756985	fwd	2756985	2756990		
RVS_(I)-2759715	rvs	2759710	2759715		
RVS_(I)-2762221	rvs	2762216	2762221		
RVS_(I)-2766525	rvs	2766520	2766525		
FWD_(III)-2766597	fwd	2766597	2766602		
RVS_(I)-2768569	rvs	2768564	2768569		
RVS_(I)-2775438	rvs	2775433	2775438		
RVS_(I)-2778625	rvs	2778620	2778625		SCO2571-2778624
RVS_(III)-2782028	rvs	2782023	2782028		
RVS_(I)-2784088	rvs	2784083	2784088		
FWD_(I)-2787005	fwd	2787005	2787010		
RVS_(I)-2787174	rvs	2787169	2787174		
FWD_(II)-2787572	fwd	2787572	2787577		
RVS_(II)-2789583	rvs	2789578	2789583		
RVS_(I)-2790239	rvs	2790234	2790239		
RVS_(III)-2792315	rvs	2792310	2792315		SCO2586-2792304
FWD_(III)-2796995	fwd	2796995	2797000		
FWD_(II)-2797071	fwd	2797071	2797076		
RVS_(I)-2797087	rvs	2797082	2797087		
RVS_(I)-2797141	rvs	2797136	2797141		
FWD_(II)-2797907	fwd	2797907	2797912		
RVS_(I)-2810672	rvs	2810667	2810672		SCO2594-2810671
RVS_(I)-2813095	rvs	2813090	2813095		
RVS_(I)-2819512	rvs	2819507	2819512		
RVS_(I)-2829378	rvs	2829373	2829378		
RVS_(I)-2836015	rvs	2836010	2836015	mreBCDr-2836016(rvs)	
RVS_(I)-2836090	rvs	2836085	2836090	mreBCDr-2836091(rvs)	
RVS_(I)-2838672	rvs	2838667	2838672		
RVS_(III)-2841582	rvs	2841577	2841582		
RVS_(I)-2844163	rvs	2844158	2844163		
FWD_(I)-2844654	fwd	2844654	2844659		
RVS_(I)-2845747	rvs	2845742	2845747		
RVS_(I)-2847515	rvs	2847510	2847515		
FWD_(I)-2847782	fwd	2847782	2847787		
RVS_(I)-2847824	rvs	2847819	2847824		
RVS_(I)-2848242	rvs	2848237	2848242		
RVS_(I)-2848650	rvs	2848645	2848650		
RVS_(I)-2851770	rvs	2851765	2851770		
RVS_(I)-2854598	rvs	2854593	2854598		
FWD_(III)-2858682	fwd	2858682	2858687		
RVS_(III)-2859171	rvs	2859166	2859171		
FWD_(I)-2860051	fwd	2860051	2860056		SCO2633-2860051
RVS_(I)-2870590	rvs	2870585	2870590		
FWD_(III)-2870778	fwd	2870778	2870783		
FWD_(I)-2873663	fwd	2873663	2873668		
RVS_(I)-2880370	rvs	2880365	2880370		
FWD_(III)-2885454	fwd	2885454	2885459		
RVS_(I)-2886691	rvs	2886686	2886691		
FWD_(I)-2895077	fwd	2895077	2895082		
FWD_(III)-2904722	fwd	2904722	2904727		
RVS_(I)-2907652	rvs	2907647	2907652		
FWD_(I)-2907747	fwd	2907747	2907752		
RVS_(I)-2918392	rvs	2918387	2918392		
RVS_(I)-2918452	rvs	2918447	2918452		
FWD_(II)-2919162	fwd	2919162	2919167		
FWD_(I)-2929547	fwd	2929547	2929552		
RVS_(II)-2947506	rvs	2947501	2947506		
RVS_(II)-2962637	rvs	2962632	2962637		

RVS_(I)-2970238	rvs	2970233	2970238		
RVS_(I)-2974784	rvs	2974779	2974784		
RVS_(III)-2975176	rvs	2975171	2975176		
FWD_(I)-2982224	fwd	2982224	2982229		
FWD_(I)-2982254	fwd	2982254	2982259		
RVS_(I)-2984105	rvs	2984100	2984105		SCO2736-2984105
RVS_(I)-2984674	rvs	2984669	2984674		
RVS_(I)-2996440	rvs	2996435	2996440		
RVS_(III)-3008278	rvs	3008273	3008278		
RVS_(I)-3015807	rvs	3015802	3015807		
RVS_(III)-3019925	rvs	3019920	3019925		
FWD_(I)-3022836	fwd	3022836	3022841		
RVS_(III)-3027417	rvs	3027412	3027417		
RVS_(I)-3046218	rvs	3046213	3046218		
RVS_(I)-3046229	rvs	3046224	3046229		
FWD_(III)-3046939	fwd	3046939	3046944		
FWD_(II)-3048673	fwd	3048673	3048678		
FWD_(I)-3049351	fwd	3049351	3049356		
FWD_(I)-3056376	fwd	3056376	3056381		
RVS_(III)-3062013	rvs	3062008	3062013		
FWD_(I)-3064665	fwd	3064665	3064670		
RVS_(III)-3079208	rvs	3079203	3079208		
RVS_(III)-3080357	rvs	3080352	3080357		
FWD_(III)-3082238	fwd	3082238	3082243		
RVS_(III)-3084120	rvs	3084115	3084120		
FWD_(I)-3090945	fwd	3090945	3090950		
FWD_(I)-3091792	fwd	3091792	3091797		
FWD_(I)-3094995	fwd	3094995	3095000		
FWD_(I)-3095131	fwd	3095131	3095136		
FWD_(I)-3095311	fwd	3095311	3095316		
FWD_(I)-3095388	fwd	3095388	3095393		
RVS_(III)-3100556	rvs	3100551	3100556		
RVS_(I)-3116932	rvs	3116927	3116932		
FWD_(II)-3117827	fwd	3117827	3117832		
RVS_(I)-3123433	rvs	3123428	3123433		
FWD_(III)-3131326	fwd	3131326	3131331		
FWD_(I)-3133775	fwd	3133775	3133780		
FWD_(III)-3141751	fwd	3141751	3141756		
FWD_(III)-3145612	fwd	3145612	3145617		
FWD_(II)-3145677	fwd	3145677	3145682		
FWD_(I)-3150872	fwd	3150872	3150877		
RVS_(I)-3153789	rvs	3153784	3153789		
RVS_(I)-3154576	rvs	3154571	3154576		
FWD_(I)-3154677	fwd	3154677	3154682		
FWD_(I)-3155174	fwd	3155174	3155179		
RVS_(I)-3161376	rvs	3161371	3161376		
FWD_(III)-3161531	fwd	3161531	3161536		
RVS_(I)-3163178	rvs	3163173	3163178		
RVS_(III)-3166599	rvs	3166594	3166599		
RVS_(I)-3179766	rvs	3179761	3179766	hppDr-3179764(rvs)	
RVS_(I)-3192927	rvs	3192922	3192927		
FWD_(I)-3199114	fwd	3199114	3199119		
RVS_(I)-3204036	rvs	3204031	3204036		
FWD_(II)-3204516	fwd	3204516	3204521		
RVS_(I)-3204639	rvs	3204634	3204639		
FWD_(I)-3204837	fwd	3204837	3204842		
RVS_(I)-3206922	rvs	3206917	3206922		
FWD_(I)-3208717	fwd	3208717	3208722		
RVS_(III)-3212259	rvs	3212254	3212259		

RVS_(I)-3224041	rvs	3224036	3224041		
RVS_(I)-3226999	rvs	3226994	3226999		
RVS_(I)-3227533	rvs	3227528	3227533		SCO2966-3227532
FWD_(I)-3230634	fwd	3230634	3230639		SCO2970-3230633
FWD_(II)-3230670	fwd	3230670	3230675		
RVS_(I)-3233272	rvs	3233267	3233272	prfBr-3233273(rvs)	
RVS_(I)-3234626	rvs	3234621	3234626		
RVS_(III)-3236406	rvs	3236401	3236406		
RVS_(I)-3236442	rvs	3236437	3236442		
FWD_(I)-3236626	fwd	3236626	3236631		
RVS_(II)-3246513	rvs	3246508	3246513		
FWD_(III)-3255126	fwd	3255126	3255131		
FWD_(III)-3257115	fwd	3257115	3257120		
RVS_(I)-3260950	rvs	3260945	3260950		
FWD_(I)-3262343	fwd	3262343	3262348		
FWD_(II)-3264379	fwd	3264379	3264384		
RVS_(I)-3273669	rvs	3273664	3273669		
RVS_(I)-3273913	rvs	3273908	3273913		
RVS_(II)-3277726	rvs	3277721	3277726		
RVS_(II)-3279141	rvs	3279136	3279141		
RVS_(II)-3279781	rvs	3279776	3279781		
RVS_(I)-3279874	rvs	3279869	3279874		
RVS_(I)-3283787	rvs	3283782	3283787		
RVS_(I)-3283984	rvs	3283979	3283984		
RVS_(I)-3289568	rvs	3289563	3289568		
RVS_(I)-3294216	rvs	3294211	3294216		
RVS_(II)-3298149	rvs	3298144	3298149		
RVS_(II)-3299882	rvs	3299877	3299882		
RVS_(II)-3300175	rvs	3300170	3300175		
RVS_(III)-3302230	rvs	3302225	3302230	16S_rRNAr-3302230(rvs)	
RVS_(I)-3302281	rvs	3302276	3302281	rRNAr-3302281(rvs)	
RVS_(I)-3306798	rvs	3306793	3306798		
FWD_(I)-3307621	fwd	3307621	3307626		
RVS_(I)-3314417	rvs	3314412	3314417		
FWD_(I)-3314758	fwd	3314758	3314763		
RVS_(I)-3321083	rvs	3321078	3321083	whiBr-3321084(rvs)	
RVS_(I)-3321248	rvs	3321243	3321248	whiBr-3321248(rvs)	
RVS_(I)-3321354	rvs	3321349	3321354		
FWD_(I)-3321484	fwd	3321484	3321489		
FWD_(I)-3322266	fwd	3322266	3322271		
FWD_(I)-3330747	fwd	3330747	3330752		
FWD_(I)-3332628	fwd	3332628	3332633		
FWD_(I)-3332714	fwd	3332714	3332719		
FWD_(I)-3334376	fwd	3334376	3334381		
RVS_(I)-3341166	rvs	3341161	3341166		
RVS_(I)-3342978	rvs	3342973	3342978		
RVS_(I)-3344282	rvs	3344277	3344282		
RVS_(I)-3353919	rvs	3353914	3353919		
FWD_(I)-3354047	fwd	3354047	3354052		SCO3061-3354048
RVS_(I)-3356668	rvs	3356663	3356668		
FWD_(II)-3356779	fwd	3356779	3356784		
FWD_(I)-3356874	fwd	3356874	3356879		
RVS_(I)-3360482	rvs	3360477	3360482		SCO3067-3360481
FWD_(I)-3360598	fwd	3360598	3360603		
FWD_(I)-3363078	fwd	3363078	3363083		
FWD_(III)-3374639	fwd	3374639	3374644		
FWD_(I)-3375912	fwd	3375912	3375917		
RVS_(I)-3377208	rvs	3377203	3377208		
RVS_(I)-3377277	rvs	3377272	3377277		

FWD_(III)-3379356	fwd	3379356	3379361		
RVS_(I)-3381000	rvs	3380995	3381000		
RVS_(I)-3386288	rvs	3386283	3386288		
RVS_(I)-3387938	rvs	3387933	3387938		SCO3092-3387937
RVS_(I)-3391914	rvs	3391909	3391914		
RVS_(III)-3393055	rvs	3393050	3393055		
FWD_(I)-3404418	fwd	3404418	3404423		
RVS_(III)-3408378	rvs	3408373	3408378		
RVS_(III)-3418676	rvs	3418671	3418676		
RVS_(I)-3421341	rvs	3421336	3421341		
RVS_(I)-3421387	rvs	3421382	3421387		
FWD_(I)-3422152	fwd	3422152	3422157		
FWD_(I)-3423898	fwd	3423898	3423903		
RVS_(I)-3424268	rvs	3424263	3424268		
FWD_(II)-3425050	fwd	3425050	3425055		
FWD_(I)-3434528	fwd	3434528	3434533		
RVS_(I)-3435936	rvs	3435931	3435936		
FWD_(III)-3441592	fwd	3441592	3441597		
RVS_(I)-3449017	rvs	3449012	3449017		
RVS_(I)-3453712	rvs	3453707	3453712		
FWD_(I)-3456606	fwd	3456606	3456611		
FWD_(I)-3457305	fwd	3457305	3457310		
FWD_(III)-3459030	fwd	3459030	3459035		
RVS_(III)-3461232	rvs	3461227	3461232		
FWD_(III)-3461365	fwd	3461365	3461370		
RVS_(III)-3465615	rvs	3465610	3465615		
RVS_(I)-3470268	rvs	3470263	3470268		
RVS_(I)-3474234	rvs	3474229	3474234		
RVS_(I)-3481920	rvs	3481915	3481920		
RVS_(I)-3483237	rvs	3483232	3483237		
RVS_(I)-3492670	rvs	3492665	3492670		
FWD_(I)-3492992	fwd	3492992	3492997		
FWD_(I)-3494473	fwd	3494473	3494478		
RVS_(I)-3497358	rvs	3497353	3497358		
FWD_(I)-3500032	fwd	3500032	3500037		
RVS_(III)-3506219	rvs	3506214	3506219		
RVS_(III)-3509290	rvs	3509285	3509290		
FWD_(I)-3511171	fwd	3511171	3511176		
RVS_(III)-3511750	rvs	3511745	3511750	hrdDr-3511749(rvs)	
RVS_(III)-3516055	rvs	3516050	3516055		
RVS_(II)-3522694	rvs	3522689	3522694		
FWD_(III)-3528912	fwd	3528912	3528917		
FWD_(I)-3533028	fwd	3533028	3533033		
FWD_(II)-3602352	fwd	3602352	3602357		
FWD_(III)-3602600	fwd	3602600	3602605		
FWD_(III)-3605514	fwd	3605514	3605519		
RVS_(I)-3609722	rvs	3609717	3609722		
FWD_(I)-3618213	fwd	3618213	3618218		
FWD_(I)-3619120	fwd	3619120	3619125		
RVS_(I)-3636568	rvs	3636563	3636568		
RVS_(III)-3654066	rvs	3654061	3654066		
FWD_(III)-3654135	fwd	3654135	3654140		
RVS_(I)-3663043	rvs	3663038	3663043		
RVS_(III)-3665115	rvs	3665110	3665115		
FWD_(I)-3669433	fwd	3669433	3669438		
RVS_(II)-3671713	rvs	3671708	3671713		
FWD_(I)-3672967	fwd	3672967	3672972		
RVS_(I)-3673439	rvs	3673434	3673439		
FWD_(I)-3675098	fwd	3675098	3675103		

FWD_(I)-3676100	fwd	3676100	3676105		
RVS_(I)-3678838	rvs	3678833	3678838		
RVS_(I)-3680010	rvs	3680005	3680010		
RVS_(I)-3680341	rvs	3680336	3680341	bdtAr-3680340(rvs)	
RVS_(I)-3691140	rvs	3691135	3691140		
FWD_(I)-3691217	fwd	3691217	3691222		
RVS_(I)-3699957	rvs	3699952	3699957		
FWD_(I)-3700535	fwd	3700535	3700540		
RVS_(I)-3704643	rvs	3704638	3704643		
RVS_(I)-3707328	rvs	3707323	3707328		
FWD_(I)-3707399	fwd	3707399	3707404		
RVS_(I)-3710076	rvs	3710071	3710076		
RVS_(I)-3714180	rvs	3714175	3714180		
FWD_(I)-3715513	fwd	3715513	3715518		
RVS_(I)-3723318	rvs	3723313	3723318		
FWD_(I)-3725595	fwd	3725595	3725600		
FWD_(I)-3727304	fwd	3727304	3727309		
RVS_(I)-3739011	rvs	3739006	3739011		
RVS_(II)-3739103	rvs	3739098	3739103		
RVS_(I)-3739353	rvs	3739348	3739353		
RVS_(III)-3740525	rvs	3740520	3740525		
RVS_(I)-3741123	rvs	3741118	3741123		
FWD_(I)-3741146	fwd	3741146	3741151		
RVS_(I)-3741641	rvs	3741636	3741641		
RVS_(I)-3741943	rvs	3741938	3741943		
RVS_(I)-3754485	rvs	3754480	3754485		
RVS_(I)-3756808	rvs	3756803	3756808		
FWD_(I)-3761781	fwd	3761781	3761786		SCO3397-3761781
RVS_(I)-3761851	rvs	3761846	3761851		
FWD_(II)-3764143	fwd	3764143	3764148		
FWD_(I)-3765749	fwd	3765749	3765754		
FWD_(III)-3766796	fwd	3766796	3766801		
RVS_(II)-3770190	rvs	3770185	3770190		
RVS_(I)-3770250	rvs	3770245	3770250		
RVS_(I)-3772107	rvs	3772102	3772107		
RVS_(I)-3773343	rvs	3773338	3773343		
FWD_(III)-3774814	fwd	3774814	3774819		
FWD_(I)-3775110	fwd	3775110	3775115		
FWD_(I)-3775703	fwd	3775703	3775708		
FWD_(II)-3778314	fwd	3778314	3778319		
RVS_(I)-3787875	rvs	3787870	3787875		
FWD_(I)-3794733	fwd	3794733	3794738		
RVS_(III)-3799372	rvs	3799367	3799372		
FWD_(I)-3801445	fwd	3801445	3801450		
FWD_(I)-3805287	fwd	3805287	3805292		
RVS_(I)-3808328	rvs	3808323	3808328		
RVS_(I)-3811239	rvs	3811234	3811239		
RVS_(III)-3825350	rvs	3825345	3825350		
RVS_(III)-3826467	rvs	3826462	3826467		
FWD_(III)-3831524	fwd	3831524	3831529		
RVS_(II)-3832020	rvs	3832015	3832020		
RVS_(I)-3832248	rvs	3832243	3832248		
RVS_(II)-3835468	rvs	3835463	3835468		
RVS_(I)-3835814	rvs	3835809	3835814		
RVS_(I)-3836989	rvs	3836984	3836989		
RVS_(I)-3839956	rvs	3839951	3839956		
FWD_(II)-3842723	fwd	3842723	3842728		
RVS_(I)-3853626	rvs	3853621	3853626		
RVS_(I)-3854443	rvs	3854438	3854443		

FWD_(I)-3854745	fwd	3854745	3854750		
RVS_(III)-3858499	rvs	3858494	3858499		
FWD_(I)-3858610	fwd	3858610	3858615		
FWD_(I)-3861775	fwd	3861775	3861780		
FWD_(I)-3870336	fwd	3870336	3870341		
RVS_(I)-3905142	rvs	3905137	3905142		
RVS_(III)-3907267	rvs	3907262	3907267		
RVS_(I)-3908442	rvs	3908437	3908442		
RVS_(III)-3914880	rvs	3914875	3914880		
RVS_(I)-3917967	rvs	3917962	3917967		
FWD_(I)-3923475	fwd	3923475	3923480		
RVS_(I)-3923607	rvs	3923602	3923607		
RVS_(I)-3924716	rvs	3924711	3924716		
RVS_(I)-3924873	rvs	3924868	3924873		
RVS_(I)-3933630	rvs	3933625	3933630		
RVS_(I)-3933671	rvs	3933666	3933671		
FWD_(I)-3934692	fwd	3934692	3934697		
RVS_(III)-3935803	rvs	3935798	3935803		
FWD_(I)-3935912	fwd	3935912	3935917		
FWD_(I)-3936282	fwd	3936282	3936287		
RVS_(III)-3936449	rvs	3936444	3936449		
FWD_(I)-3939552	fwd	3939552	3939557		
FWD_(I)-3943299	fwd	3943299	3943304		
FWD_(I)-3946569	fwd	3946569	3946574		
RVS_(I)-3950119	rvs	3950114	3950119		
FWD_(I)-3950227	fwd	3950227	3950232		
FWD_(I)-3950265	fwd	3950265	3950270		
RVS_(III)-3954845	rvs	3954840	3954845		
FWD_(I)-3954997	fwd	3954997	3955002		
FWD_(II)-3957037	fwd	3957037	3957042		
FWD_(III)-3958160	fwd	3958160	3958165		
RVS_(I)-3958182	rvs	3958177	3958182		
FWD_(I)-3958243	fwd	3958243	3958248		
RVS_(III)-3961105	rvs	3961100	3961105		SCO3581-3961105
FWD_(I)-3961226	fwd	3961226	3961231		
FWD_(III)-3963084	fwd	3963084	3963089		
RVS_(III)-3968618	rvs	3968613	3968618	vanRSr-3968617(rvs)	
RVS_(I)-3985789	rvs	3985784	3985789		SCO3608-3985789
FWD_(I)-3985924	fwd	3985924	3985929		
RVS_(I)-3989857	rvs	3989852	3989857		
FWD_(III)-3989989	fwd	3989989	3989994		
RVS_(I)-3992945	rvs	3992940	3992945		
RVS_(I)-3993156	rvs	3993151	3993156		
RVS_(I)-3995751	rvs	3995746	3995751		
RVS_(I)-3995824	rvs	3995819	3995824		
RVS_(I)-3997557	rvs	3997552	3997557		
RVS_(III)-3998016	rvs	3998011	3998016		SCO3619-3998015
RVS_(I)-4001470	rvs	4001465	4001470		
FWD_(III)-4001665	fwd	4001665	4001670		
RVS_(I)-4004956	rvs	4004951	4004956		
RVS_(III)-4005887	rvs	4005882	4005887		
RVS_(I)-4008341	rvs	4008336	4008341		
FWD_(I)-4013595	fwd	4013595	4013600		
RVS_(I)-4031249	rvs	4031244	4031249		
FWD_(I)-4033355	fwd	4033355	4033360		
RVS_(I)-4035926	rvs	4035921	4035926		
RVS_(I)-4038567	rvs	4038562	4038567		
FWD_(II)-4039721	fwd	4039721	4039726		
RVS_(I)-4043902	rvs	4043897	4043902		

FWD_(III)-4043962	fwd	4043962	4043967		
RVS_(I)-4048318	rvs	4048313	4048318		
FWD_(I)-4051472	fwd	4051472	4051477		
RVS_(I)-4052726	rvs	4052721	4052726	dnaKr-4052725(rvs)	
FWD_(I)-4059496	fwd	4059496	4059501		
RVS_(I)-4062019	rvs	4062014	4062019		
FWD_(I)-4062131	fwd	4062131	4062136		SCOt30-4062131
RVS_(I)-4062172	rvs	4062167	4062172		
FWD_(I)-4064602	fwd	4064602	4064607		
RVS_(I)-4067494	rvs	4067489	4067494		
FWD_(I)-4067669	fwd	4067669	4067674		
RVS_(III)-4068714	rvs	4068709	4068714		
FWD_(I)-4071088	fwd	4071088	4071093		
FWD_(III)-4081161	fwd	4081161	4081166		
FWD_(III)-4088150	fwd	4088150	4088155		
RVS_(I)-4090509	rvs	4090504	4090509		SCO3715-4090508
RVS_(I)-4103851	rvs	4103846	4103851		
FWD_(I)-4105234	fwd	4105234	4105239		
RVS_(I)-4109238	rvs	4109233	4109238		
RVS_(I)-4119138	rvs	4119133	4119138		
RVS_(I)-4119272	rvs	4119267	4119272	cspAr-4119269(rvs)	SCO3748-4119271
RVS_(I)-4120171	rvs	4120166	4120171		
RVS_(III)-4135037	rvs	4135032	4135037		
FWD_(I)-4151472	fwd	4151472	4151477		
FWD_(I)-4153041	fwd	4153041	4153046		SCO3778-4153041
RVS_(I)-4167934	rvs	4167929	4167934		
FWD_(III)-4169611	fwd	4169611	4169616		
FWD_(I)-4171299	fwd	4171299	4171304		
RVS_(III)-4174567	rvs	4174562	4174567		
RVS_(III)-4179536	rvs	4179531	4179536		
FWD_(I)-4179615	fwd	4179615	4179620		
RVS_(III)-4189716	rvs	4189711	4189716		
RVS_(I)-4196092	rvs	4196087	4196092		
RVS_(I)-4197433	rvs	4197428	4197433		
FWD_(I)-4197520	fwd	4197520	4197525		
RVS_(III)-4200543	rvs	4200538	4200543		
FWD_(I)-4200566	fwd	4200566	4200571		
FWD_(I)-4200695	fwd	4200695	4200700		
RVS_(I)-4203093	rvs	4203088	4203093		
FWD_(III)-4203210	fwd	4203210	4203215		
RVS_(III)-4205082	rvs	4205077	4205082		
RVS_(III)-4214500	rvs	4214495	4214500	bkdA2B2C2r-4214496(rvs)	
RVS_(I)-4222871	rvs	4222866	4222871		
RVS_(I)-4223719	rvs	4223714	4223719		
RVS_(III)-4226896	rvs	4226891	4226896		
FWD_(I)-4227007	fwd	4227007	4227012		
FWD_(I)-4233527	fwd	4233527	4233532		
RVS_(I)-4238361	rvs	4238356	4238361		
RVS_(I)-4239207	rvs	4239202	4239207		
RVS_(II)-4241411	rvs	4241406	4241411		
RVS_(I)-4243385	rvs	4243380	4243385		
RVS_(III)-4243801	rvs	4243796	4243801		
RVS_(I)-4247967	rvs	4247962	4247967		
FWD_(III)-4252821	fwd	4252821	4252826		
RVS_(I)-4258462	rvs	4258457	4258462		
RVS_(I)-4258917	rvs	4258912	4258917		
RVS_(I)-4259903	rvs	4259898	4259903		SCOt33-4259901
RVS_(I)-4260907	rvs	4260902	4260907		
RVS_(I)-4265575	rvs	4265570	4265575		SCO3874-4265574

RVS_(I)-4270496	rvs	4270491	4270496		
RVS_(I)-4272844	rvs	4272839	4272844		
FWD_(I)-4272883	fwd	4272883	4272888		
FWD_(I)-4272963	fwd	4272963	4272968		
FWD_(I)-4273559	fwd	4273559	4273564		
FWD_(II)-4274036	fwd	4274036	4274041		
FWD_(I)-4277021	fwd	4277021	4277026	parABf-4277024(fwd)	
FWD_(I)-4279316	fwd	4279316	4279321		
RVS_(I)-4281615	rvs	4281610	4281615	trxBAr-4281616(rvs)	
RVS_(I)-4282644	rvs	4282639	4282644		
RVS_(I)-4285071	rvs	4285066	4285071		
RVS_(III)-4295599	rvs	4295594	4295599		
RVS_(I)-4303014	rvs	4303009	4303014		
FWD_(I)-4303079	fwd	4303079	4303084		SCO3906-4303080
FWD_(II)-4303167	fwd	4303167	4303172		
FWD_(III)-4306710	fwd	4306710	4306715		
FWD_(I)-4315358	fwd	4315358	4315363		
FWD_(I)-4315418	fwd	4315418	4315423		
FWD_(I)-4318070	fwd	4318070	4318075		
FWD_(I)-4321572	fwd	4321572	4321577		
FWD_(I)-4322963	fwd	4322963	4322968		
FWD_(III)-4323842	fwd	4323842	4323847		
RVS_(II)-4325797	rvs	4325792	4325797		
RVS_(I)-4325868	rvs	4325863	4325868		
RVS_(I)-4326773	rvs	4326768	4326773		
FWD_(II)-4327942	fwd	4327942	4327947		
RVS_(II)-4332700	rvs	4332695	4332700		
RVS_(I)-4336434	rvs	4336429	4336434		
RVS_(I)-4337698	rvs	4337693	4337698		
RVS_(III)-4339150	rvs	4339145	4339150		
FWD_(I)-4339530	fwd	4339530	4339535		
FWD_(I)-4340821	fwd	4340821	4340826	cydABCDf-4340819(fwd)	
FWD_(I)-4355984	fwd	4355984	4355989		
RVS_(I)-4361826	rvs	4361821	4361826		
RVS_(III)-4369813	rvs	4369808	4369813		
FWD_(II)-4370022	fwd	4370022	4370027		
FWD_(I)-4372114	fwd	4372114	4372119		
RVS_(I)-4373706	rvs	4373701	4373706		
FWD_(I)-4373868	fwd	4373868	4373873		
FWD_(I)-4373975	fwd	4373975	4373980		
RVS_(III)-4373984	rvs	4373979	4373984		
RVS_(I)-4376917	rvs	4376912	4376917		SCO3974-4376916
RVS_(I)-4378083	rvs	4378078	4378083		
FWD_(I)-4378924	fwd	4378924	4378929		
FWD_(I)-4379250	fwd	4379250	4379255		
FWD_(I)-4382030	fwd	4382030	4382035		
FWD_(I)-4382657	fwd	4382657	4382662		
RVS_(III)-4386361	rvs	4386356	4386361		
RVS_(I)-4387540	rvs	4387535	4387540		
RVS_(III)-4389068	rvs	4389063	4389068		
FWD_(II)-4393893	fwd	4393893	4393898		
FWD_(III)-4397767	fwd	4397767	4397772		
FWD_(III)-4400929	fwd	4400929	4400934		
FWD_(I)-4401679	fwd	4401679	4401684		
RVS_(I)-4406762	rvs	4406757	4406762		
FWD_(III)-4407434	fwd	4407434	4407439		
RVS_(I)-4407796	rvs	4407791	4407796		
FWD_(I)-4409011	fwd	4409011	4409016		
FWD_(I)-4415204	fwd	4415204	4415209		

RVS_(I)-4424020	rvs	4424015	4424020		
RVS_(III)-4424465	rvs	4424460	4424465		
FWD_(I)-4424575	fwd	4424575	4424580		
RVS_(I)-4430186	rvs	4430181	4430186		
RVS_(III)-4431741	rvs	4431736	4431741	sigNr-4431741(rvs)	SCO4034-4431740
RVS_(III)-4433638	rvs	4433633	4433638		
FWD_(I)-4433648	fwd	4433648	4433653		
RVS_(III)-4437187	rvs	4437182	4437187		SCO4043-4437184
RVS_(III)-4449139	rvs	4449134	4449139		
FWD_(III)-4449193	fwd	4449193	4449198		
RVS_(I)-4452207	rvs	4452202	4452207		
RVS_(I)-4456953	rvs	4456948	4456953		
FWD_(III)-4456987	fwd	4456987	4456992		
FWD_(I)-4457029	fwd	4457029	4457034		
FWD_(I)-4461056	fwd	4461056	4461061		
FWD_(III)-4463290	fwd	4463290	4463295		
RVS_(I)-4466056	rvs	4466051	4466056		
RVS_(I)-4469990	rvs	4469985	4469990		
RVS_(I)-4470150	rvs	4470145	4470150		
RVS_(I)-4470725	rvs	4470720	4470725		
FWD_(I)-4470727	fwd	4470727	4470732		
FWD_(II)-4470898	fwd	4470898	4470903		
FWD_(I)-4479327	fwd	4479327	4479332		
RVS_(I)-4482431	rvs	4482426	4482431		SCO4088-4482428
RVS_(I)-4482453	rvs	4482448	4482453		
RVS_(III)-4483812	rvs	4483807	4483812	vdhr-4483811(rvs)	
FWD_(I)-4483901	fwd	4483901	4483906		
FWD_(I)-4484899	fwd	4484899	4484904		
FWD_(III)-4485007	fwd	4485007	4485012		
RVS_(I)-4485057	rvs	4485052	4485057		
FWD_(II)-4485240	fwd	4485240	4485245	bldCf-4485238(fwd)	
FWD_(II)-4485322	fwd	4485322	4485327		
RVS_(I)-4486579	rvs	4486574	4486579		
RVS_(I)-4490914	rvs	4490909	4490914		
RVS_(III)-4491050	rvs	4491045	4491050		
RVS_(I)-4492814	rvs	4492809	4492814		
FWD_(III)-4492828	fwd	4492828	4492833		
FWD_(I)-4494009	fwd	4494009	4494014		SCO4096-4494009
FWD_(I)-4498114	fwd	4498114	4498119		
RVS_(I)-4502810	rvs	4502805	4502810		
FWD_(I)-4505450	fwd	4505450	4505455		
FWD_(I)-4507126	fwd	4507126	4507131		
FWD_(I)-4508513	fwd	4508513	4508518		
RVS_(III)-4511704	rvs	4511699	4511704		
RVS_(I)-4515139	rvs	4515134	4515139		SCO4114-4515138
FWD_(I)-4515751	fwd	4515751	4515756		
RVS_(III)-4524527	rvs	4524522	4524527		
FWD_(I)-4524781	fwd	4524781	4524786		
FWD_(I)-4530415	fwd	4530415	4530420		SCOr13-4530415
FWD_(II)-4530908	fwd	4530908	4530913		
FWD_(II)-4531829	fwd	4531829	4531834		
FWD_(II)-4532199	fwd	4532199	4532204		
FWD_(II)-4532253	fwd	4532253	4532258		
FWD_(II)-4534905	fwd	4534905	4534910		
RVS_(I)-4538796	rvs	4538791	4538796		
RVS_(I)-4545841	rvs	4545836	4545841		
FWD_(III)-4546047	fwd	4546047	4546052		
FWD_(I)-4547180	fwd	4547180	4547185		
RVS_(III)-4550071	rvs	4550066	4550071		

FWD_(I)-4551104	fwd	4551104	4551109		
RVS_(III)-4551717	rvs	4551712	4551717		
RVS_(I)-4557893	rvs	4557888	4557893	pstSr-4557895(rvs)	
RVS_(I)-4562290	rvs	4562285	4562290		
RVS_(I)-4574986	rvs	4574981	4574986		
RVS_(I)-4577180	rvs	4577175	4577180	glnRr-4577180(rvs)	
FWD_(I)-4578069	fwd	4578069	4578074		
FWD_(I)-4580774	fwd	4580774	4580779		
FWD_(I)-4582262	fwd	4582262	4582267		
FWD_(I)-4588574	fwd	4588574	4588579		
RVS_(III)-4588581	rvs	4588576	4588581		
FWD_(II)-4590561	fwd	4590561	4590566		
FWD_(II)-4591019	fwd	4591019	4591024		
FWD_(I)-4597385	fwd	4597385	4597390		
FWD_(III)-4598635	fwd	4598635	4598640	devABf-4598635(fwd)	
FWD_(III)-4606581	fwd	4606581	4606586		
RVS_(III)-4607264	rvs	4607259	4607264		
FWD_(I)-4607362	fwd	4607362	4607367		
FWD_(I)-4608516	fwd	4608516	4608521		
FWD_(II)-4608527	fwd	4608527	4608532		
FWD_(I)-4614619	fwd	4614619	4614624		
RVS_(I)-4615970	rvs	4615965	4615970		
FWD_(I)-4618020	fwd	4618020	4618025		
FWD_(I)-4625942	fwd	4625942	4625947		
FWD_(II)-4626220	fwd	4626220	4626225		
RVS_(II)-4631877	rvs	4631872	4631877		
RVS_(III)-4633056	rvs	4633051	4633056	phoUr-4633055(rvs)	
FWD_(I)-4633199	fwd	4633199	4633204	phoRPf-4633201(fwd)	
FWD_(I)-4636721	fwd	4636721	4636726		
RVS_(III)-4637493	rvs	4637488	4637493		
FWD_(I)-4639322	fwd	4639322	4639327		
RVS_(II)-4652565	rvs	4652560	4652565		
RVS_(III)-4662953	rvs	4662948	4662953		
RVS_(III)-4666470	rvs	4666465	4666470		
FWD_(I)-4667076	fwd	4667076	4667081		
FWD_(I)-4668591	fwd	4668591	4668596		
FWD_(II)-4670132	fwd	4670132	4670137		
FWD_(I)-4674581	fwd	4674581	4674586		
RVS_(III)-4675330	rvs	4675325	4675330		
RVS_(I)-4693489	rvs	4693484	4693489		
RVS_(I)-4693507	rvs	4693502	4693507		
FWD_(I)-4695458	fwd	4695458	4695463		SCO4281-4695457
FWD_(I)-4697268	fwd	4697268	4697273		
FWD_(I)-4704546	fwd	4704546	4704551		
FWD_(I)-4708375	fwd	4708375	4708380		
FWD_(I)-4710964	fwd	4710964	4710969	groEL2f-4710964(fwd)	SCO4296-4710964
RVS_(I)-4717144	rvs	4717139	4717144		
RVS_(I)-4718223	rvs	4718218	4718223		
FWD_(III)-4722391	fwd	4722391	4722396		
FWD_(I)-4729584	fwd	4729584	4729589		
RVS_(III)-4731460	rvs	4731455	4731460		
RVS_(I)-4734674	rvs	4734669	4734674		
RVS_(III)-4741368	rvs	4741363	4741368		
FWD_(I)-4741580	fwd	4741580	4741585		
RVS_(I)-4748437	rvs	4748432	4748437		
RVS_(III)-4748797	rvs	4748792	4748797		SCO4333-4748796
RVS_(I)-4750672	rvs	4750667	4750672		
FWD_(I)-4755170	fwd	4755170	4755175		
FWD_(II)-4757053	fwd	4757053	4757058		

FWD_(II)-4758456	fwd	4758456	4758461		
RVS_(III)-4766981	rvs	4766976	4766981		
FWD_(III)-4767683	fwd	4767683	4767688		
RVS_(I)-4769659	rvs	4769654	4769659		
RVS_(I)-4779348	rvs	4779343	4779348		SCO4366-4779347
RVS_(III)-4785385	rvs	4785380	4785385		
RVS_(III)-4786455	rvs	4786450	4786455		
RVS_(III)-4805790	rvs	4805785	4805790		
FWD_(III)-4806880	fwd	4806880	4806885		
FWD_(III)-4810778	fwd	4810778	4810783		
FWD_(I)-4811627	fwd	4811627	4811632		
RVS_(I)-4811828	rvs	4811823	4811828		
RVS_(III)-4821085	rvs	4821080	4821085		
RVS_(I)-4828004	rvs	4827999	4828004		
RVS_(I)-4828046	rvs	4828041	4828046		
RVS_(I)-4828093	rvs	4828088	4828093		
FWD_(III)-4830456	fwd	4830456	4830461		
RVS_(I)-4842717	rvs	4842712	4842717	afsSr-4842717(rvs)	
FWD_(III)-4847949	fwd	4847949	4847954		
RVS_(I)-4858236	rvs	4858231	4858236		
RVS_(I)-4859677	rvs	4859672	4859677		
FWD_(I)-4862394	fwd	4862394	4862399		
FWD_(I)-4862621	fwd	4862621	4862626		
FWD_(III)-4863597	fwd	4863597	4863602		SCO4441-4863597
FWD_(I)-4864623	fwd	4864623	4864628		
FWD_(I)-4869075	fwd	4869075	4869080		
RVS_(I)-4886753	rvs	4886748	4886753		SCO4468-4886752
RVS_(I)-4886857	rvs	4886852	4886857		
FWD_(I)-4886912	fwd	4886912	4886917		
FWD_(I)-4889130	fwd	4889130	4889135		
RVS_(I)-4893835	rvs	4893830	4893835		
RVS_(I)-4909579	rvs	4909574	4909579		
FWD_(III)-4909614	fwd	4909614	4909619		
FWD_(I)-4912005	fwd	4912005	4912010		
FWD_(I)-4915902	fwd	4915902	4915907		
FWD_(I)-4916377	fwd	4916377	4916382		
FWD_(I)-4917934	fwd	4917934	4917939		
FWD_(I)-4922615	fwd	4922615	4922620		
RVS_(I)-4926108	rvs	4926103	4926108		SCO4505-4926107
RVS_(I)-4926246	rvs	4926241	4926246		
RVS_(I)-4933623	rvs	4933618	4933623		
FWD_(I)-4933735	fwd	4933735	4933740		
RVS_(II)-4936689	rvs	4936684	4936689		
RVS_(I)-4943457	rvs	4943452	4943457		
FWD_(I)-4943532	fwd	4943532	4943537		
RVS_(I)-4945437	rvs	4945432	4945437		
FWD_(III)-4949084	fwd	4949084	4949089		
FWD_(I)-4960034	fwd	4960034	4960039		
RVS_(III)-4960485	rvs	4960480	4960485		
FWD_(I)-4960539	fwd	4960539	4960544		
FWD_(I)-4961638	fwd	4961638	4961643		
FWD_(III)-4967883	fwd	4967883	4967888		
FWD_(I)-4980142	fwd	4980142	4980147		
RVS_(I)-4984794	rvs	4984789	4984794		
RVS_(II)-4992161	rvs	4992156	4992161		
RVS_(III)-4997196	rvs	4997191	4997196		
FWD_(III)-4997331	fwd	4997331	4997336		
FWD_(I)-5004849	fwd	5004849	5004854		
FWD_(I)-5006077	fwd	5006077	5006082		

FWD_(I)-5006125	fwd	5006125	5006130		
FWD_(I)-5010216	fwd	5010216	5010221		
RVS_(III)-5015369	rvs	5015364	5015369		
RVS_(I)-5019530	rvs	5019525	5019530		
RVS_(III)-5020447	rvs	5020442	5020447		
RVS_(III)-5031250	rvs	5031245	5031250		
FWD_(III)-5033866	fwd	5033866	5033871		
RVS_(I)-5037275	rvs	5037270	5037275		
FWD_(I)-5037333	fwd	5037333	5037338		
FWD_(I)-5038463	fwd	5038463	5038468		
FWD_(II)-5038552	fwd	5038552	5038557		
FWD_(I)-5043145	fwd	5043145	5043150		
FWD_(I)-5054804	fwd	5054804	5054809		
RVS_(I)-5055174	rvs	5055169	5055174		
FWD_(II)-5055790	fwd	5055790	5055795		
FWD_(I)-5057877	fwd	5057877	5057882		
RVS_(I)-5059682	rvs	5059677	5059682		
FWD_(I)-5061312	fwd	5061312	5061317		
FWD_(II)-5061363	fwd	5061363	5061368		
FWD_(II)-5061560	fwd	5061560	5061565		
RVS_(I)-5070976	rvs	5070971	5070976		
FWD_(I)-5071138	fwd	5071138	5071143		SCO4646-5071138
FWD_(I)-5071207	fwd	5071207	5071212		
FWD_(I)-5072786	fwd	5072786	5072791	rplKf-5072787(fwd)	
FWD_(I)-5075190	fwd	5075190	5075195		
FWD_(I)-5076199	fwd	5076199	5076204	rplJLf-5076201(fwd)	SCO4652-5076200
FWD_(I)-5077716	fwd	5077716	5077721		
FWD_(I)-5077768	fwd	5077768	5077773		
FWD_(I)-5088759	fwd	5088759	5088764		
RVS_(I)-5089832	rvs	5089827	5089832		
FWD_(I)-5092006	fwd	5092006	5092011	tuf1f-5092005(fwd)	
FWD_(III)-5099208	fwd	5099208	5099213		
RVS_(III)-5106711	rvs	5106706	5106711		
FWD_(I)-5108124	fwd	5108124	5108129		
FWD_(I)-5113588	fwd	5113588	5113593		
RVS_(I)-5117024	rvs	5117019	5117024		
RVS_(I)-5117056	rvs	5117051	5117056		
RVS_(III)-5123093	rvs	5123088	5123093		
RVS_(III)-5123876	rvs	5123871	5123876		
FWD_(I)-5127282	fwd	5127282	5127287		
FWD_(II)-5138714	fwd	5138714	5138719		
FWD_(II)-5141213	fwd	5141213	5141218		
FWD_(I)-5142279	fwd	5142279	5142284		
FWD_(I)-5148552	fwd	5148552	5148557		
FWD_(I)-5153334	fwd	5153334	5153339		
FWD_(I)-5158505	fwd	5158505	5158510		
FWD_(I)-5161069	fwd	5161069	5161074		
FWD_(I)-5168215	fwd	5168215	5168220		
FWD_(III)-5172614	fwd	5172614	5172619	groESL1f-5172612(fwd)	
RVS_(I)-5172749	rvs	5172744	5172749		
FWD_(III)-5179389	fwd	5179389	5179394		
RVS_(I)-5179429	rvs	5179424	5179429	whiDr-5179430(rvs)	
FWD_(I)-5180479	fwd	5180479	5180484		
FWD_(II)-5180619	fwd	5180619	5180624		
FWD_(I)-5181314	fwd	5181314	5181319		
FWD_(I)-5182963	fwd	5182963	5182968		
FWD_(I)-5184400	fwd	5184400	5184405		SCO4772-5184400
FWD_(I)-5189066	fwd	5189066	5189071		
FWD_(III)-5191354	fwd	5191354	5191359		

FWD_(I)-5194479	fwd	5194479	5194484		
FWD_(I)-5197629	fwd	5197629	5197634		
RVS_(I)-5204414	rvs	5204409	5204414		
RVS_(I)-5204983	rvs	5204978	5204983		SCO4784-5204982
FWD_(I)-5205131	fwd	5205131	5205136		
RVS_(I)-5211923	rvs	5211918	5211923		
FWD_(I)-5212113	fwd	5212113	5212118		
FWD_(III)-5218621	fwd	5218621	5218626		
RVS_(I)-5222949	rvs	5222944	5222949		
FWD_(I)-5224380	fwd	5224380	5224385		
FWD_(I)-5224391	fwd	5224391	5224396		
FWD_(III)-5234881	fwd	5234881	5234886		
RVS_(I)-5249681	rvs	5249676	5249681		
RVS_(III)-5253001	rvs	5252996	5253001		
RVS_(III)-5255433	rvs	5255428	5255433		
FWD_(III)-5255564	fwd	5255564	5255569		
FWD_(I)-5257257	fwd	5257257	5257262		
RVS_(I)-5261304	rvs	5261299	5261304		
FWD_(I)-5271525	fwd	5271525	5271530		
FWD_(I)-5272674	fwd	5272674	5272679		
FWD_(I)-5279853	fwd	5279853	5279858		
RVS_(III)-5280028	rvs	5280023	5280028		
RVS_(I)-5288697	rvs	5288692	5288697		
FWD_(III)-5288818	fwd	5288818	5288823		
RVS_(III)-5303473	rvs	5303468	5303473		
FWD_(I)-5303491	fwd	5303491	5303496		
FWD_(I)-5303704	fwd	5303704	5303709		
FWD_(I)-5310555	fwd	5310555	5310560		
FWD_(II)-5315450	fwd	5315450	5315455		
FWD_(I)-5315640	fwd	5315640	5315645		
FWD_(III)-5319336	fwd	5319336	5319341		
RVS_(I)-5330841	rvs	5330836	5330841		
RVS_(I)-5336592	rvs	5336587	5336592		
RVS_(I)-5337184	rvs	5337179	5337184		
RVS_(III)-5340203	rvs	5340198	5340203	afsQr-5340202(rvs)	
FWD_(I)-5340374	fwd	5340374	5340379		
FWD_(I)-5346245	fwd	5346245	5346250		
FWD_(I)-5352573	fwd	5352573	5352578		
FWD_(II)-5354161	fwd	5354161	5354166		
FWD_(I)-5354193	fwd	5354193	5354198		SCO4920-5354193
RVS_(I)-5357079	rvs	5357074	5357079		SCO4921-5357078
RVS_(III)-5357210	rvs	5357205	5357210		
RVS_(I)-5358663	rvs	5358658	5358663		
FWD_(III)-5364676	fwd	5364676	5364681		
RVS_(III)-5368023	rvs	5368018	5368023		
FWD_(I)-5368406	fwd	5368406	5368411		
FWD_(I)-5368512	fwd	5368512	5368517		
FWD_(I)-5368621	fwd	5368621	5368626		
RVS_(III)-5378731	rvs	5378726	5378731		
FWD_(I)-5381058	fwd	5381058	5381063		SCO4947-5381058
FWD_(III)-5391976	fwd	5391976	5391981		
RVS_(III)-5393751	rvs	5393746	5393751		
FWD_(I)-5393846	fwd	5393846	5393851		
FWD_(III)-5396674	fwd	5396674	5396679		
FWD_(I)-5397988	fwd	5397988	5397993		
RVS_(III)-5400600	rvs	5400595	5400600		
RVS_(I)-5401166	rvs	5401161	5401166		
FWD_(III)-5401181	fwd	5401181	5401186		
FWD_(I)-5405872	fwd	5405872	5405877		

RVS_(I)-5416002	rvs	5415997	5416002		SCO4978-5416001
RVS_(I)-5416064	rvs	5416059	5416064		
FWD_(I)-5431032	fwd	5431032	5431037		
RVS_(I)-5436351	rvs	5436346	5436351		
RVS_(III)-5436449	rvs	5436444	5436449		
FWD_(I)-5441939	fwd	5441939	5441944		
RVS_(I)-5455883	rvs	5455878	5455883		
FWD_(III)-5462581	fwd	5462581	5462586		
RVS_(III)-5463125	rvs	5463120	5463125		
FWD_(I)-5464197	fwd	5464197	5464202		
FWD_(I)-5464551	fwd	5464551	5464556		SCO5029-5464551
FWD_(III)-5465595	fwd	5465595	5465600		
RVS_(I)-5468491	rvs	5468486	5468491	ahpCDr-5468492(rvs)	
FWD_(III)-5468546	fwd	5468546	5468551		
RVS_(I)-5474641	rvs	5474636	5474641		
RVS_(I)-5479784	rvs	5479779	5479784		
RVS_(III)-5479814	rvs	5479809	5479814		
RVS_(I)-5482010	rvs	5482005	5482010		
RVS_(I)-5485482	rvs	5485477	5485482		
RVS_(I)-5485635	rvs	5485630	5485635		
FWD_(III)-5485654	fwd	5485654	5485659		
FWD_(III)-5488235	fwd	5488235	5488240		
RVS_(III)-5495898	rvs	5495893	5495898		
FWD_(I)-5497994	fwd	5497994	5497999		
FWD_(II)-5499055	fwd	5499055	5499060		
RVS_(III)-5500409	rvs	5500404	5500409		
FWD_(III)-5500543	fwd	5500543	5500548		
FWD_(I)-5519054	fwd	5519054	5519059		
RVS_(I)-5524014	rvs	5524009	5524014	actII-1r-5524014(rvs)	
FWD_(I)-5528063	fwd	5528063	5528068	actII-orf4f-5528063(fwd)	
RVS_(I)-5528519	rvs	5528514	5528519		
FWD_(III)-5540983	fwd	5540983	5540988		
FWD_(I)-5542131	fwd	5542131	5542136		SCO5101-5542131
FWD_(III)-5554455	fwd	5554455	5554460		
FWD_(I)-5556919	fwd	5556919	5556924		
RVS_(III)-5572343	rvs	5572338	5572343		
FWD_(I)-5574581	fwd	5574581	5574586		
FWD_(III)-5582817	fwd	5582817	5582822		
FWD_(III)-5587645	fwd	5587645	5587650		
RVS_(II)-5590240	rvs	5590235	5590240		
FWD_(I)-5592362	fwd	5592362	5592367		SCO5145-5592361
FWD_(I)-5593465	fwd	5593465	5593470		SCO5147-5593465
FWD_(I)-5593587	fwd	5593587	5593592		
RVS_(I)-5600210	rvs	5600205	5600210		
RVS_(III)-5605296	rvs	5605291	5605296		
FWD_(I)-5607428	fwd	5607428	5607433		
FWD_(III)-5609278	fwd	5609278	5609283		
RVS_(III)-5610122	rvs	5610117	5610122		
RVS_(I)-5615316	rvs	5615311	5615316		
RVS_(I)-5617120	rvs	5617115	5617120		
RVS_(III)-5618321	rvs	5618316	5618321		
FWD_(I)-5619282	fwd	5619282	5619287		
FWD_(III)-5621407	fwd	5621407	5621412		
FWD_(III)-5626385	fwd	5626385	5626390		SCO5178-5626387
RVS_(III)-5634748	rvs	5634743	5634748		
FWD_(I)-5641824	fwd	5641824	5641829		
RVS_(III)-5644726	rvs	5644721	5644726		
FWD_(I)-5647134	fwd	5647134	5647139		SCO5189-5647134
FWD_(I)-5647184	fwd	5647184	5647189		

RVS_(I)-5650047	rvs	5650042	5650047		
FWD_(I)-5652116	fwd	5652116	5652121		
FWD_(III)-5657544	fwd	5657544	5657549		SCO5200-5657544
RVS_(I)-5657574	rvs	5657569	5657574		
RVS_(III)-5657635	rvs	5657630	5657635		
FWD_(III)-5659353	fwd	5659353	5659358		
FWD_(II)-5659650	fwd	5659650	5659655		
FWD_(I)-5664553	fwd	5664553	5664558		
FWD_(I)-5664843	fwd	5664843	5664848		
FWD_(I)-5664876	fwd	5664876	5664881		
RVS_(I)-5666899	rvs	5666894	5666899		
RVS_(I)-5667361	rvs	5667356	5667361	catRr-5667360(rvs)	
RVS_(I)-5667433	rvs	5667428	5667433	catRr-5667430(rvs)	
FWD_(I)-5673488	fwd	5673488	5673493		
FWD_(I)-5675004	fwd	5675004	5675009	sigRf-5675003(fwd)	
FWD_(III)-5675179	fwd	5675179	5675184	sigRf-5675178(fwd)	
FWD_(II)-5675764	fwd	5675764	5675769		
FWD_(I)-5676317	fwd	5676317	5676322		SCO5218-5676317
RVS_(I)-5688405	rvs	5688400	5688405	nrdABSr-5688405(rvs)	
RVS_(III)-5692295	rvs	5692290	5692295		
FWD_(III)-5699726	fwd	5699726	5699731		
RVS_(I)-5702285	rvs	5702280	5702285		
RVS_(III)-5703402	rvs	5703397	5703402		
FWD_(III)-5703529	fwd	5703529	5703534		
RVS_(I)-5706015 *	rvs	5706010	5706015	ushY-ushX-sigHr-5706016(rvs)	
RVS_(III)-5709379	rvs	5709374	5709379		
FWD_(III)-5709572	fwd	5709572	5709577		
FWD_(I)-5713933	fwd	5713933	5713938		
RVS_(I)-5714688	rvs	5714683	5714688		
RVS_(I)-5725710	rvs	5725705	5725710		
FWD_(III)-5727136	fwd	5727136	5727141		
RVS_(III)-5727184	rvs	5727179	5727184		
RVS_(III)-5753715	rvs	5753710	5753715		
RVS_(III)-5769625	rvs	5769620	5769625		
RVS_(I)-5779338	rvs	5779333	5779338		
FWD_(III)-5788465	fwd	5788465	5788470		
FWD_(III)-5790803	fwd	5790803	5790808		
FWD_(I)-5796340	fwd	5796340	5796345		
RVS_(III)-5797661	rvs	5797656	5797661		
FWD_(III)-5798773	fwd	5798773	5798778		
RVS_(I)-5798855	rvs	5798850	5798855		
FWD_(III)-5805822	fwd	5805822	5805827		
FWD_(I)-5807011	fwd	5807011	5807016		
RVS_(I)-5809893	rvs	5809888	5809893		
RVS_(I)-5814864	rvs	5814859	5814864		
FWD_(I)-5818165	fwd	5818165	5818170		
RVS_(III)-5818829	rvs	5818824	5818829		
FWD_(I)-5818975	fwd	5818975	5818980		
FWD_(I)-5819600	fwd	5819600	5819605		
FWD_(II)-5822182	fwd	5822182	5822187		
FWD_(I)-5824728	fwd	5824728	5824733		
RVS_(I)-5825840	rvs	5825835	5825840		
FWD_(I)-5825846	fwd	5825846	5825851		
FWD_(I)-5828646	fwd	5828646	5828651		
FWD_(I)-5829950	fwd	5829950	5829955		
FWD_(I)-5833774	fwd	5833774	5833779		
FWD_(I)-5835114	fwd	5835114	5835119		
FWD_(II)-5835134	fwd	5835134	5835139		
FWD_(I)-5835174	fwd	5835174	5835179		

FWD_(I)-5836644	fwd	5836644	5836649	
FWD_(III)-5836731	fwd	5836731	5836736	
FWD_(I)-5844225	fwd	5844225	5844230	SCO5374-5844225
RVS_(III)-5850687	rvs	5850682	5850687	
FWD_(I)-5854579	fwd	5854579	5854584	
FWD_(III)-5860119	fwd	5860119	5860124	
RVS_(I)-5862681	rvs	5862676	5862681	
RVS_(I)-5864546	rvs	5864541	5864546	
RVS_(I)-5865636	rvs	5865631	5865636	
RVS_(III)-5869991	rvs	5869986	5869991	
FWD_(III)-5870326	fwd	5870326	5870331	
RVS_(III)-5870466	rvs	5870461	5870466	
FWD_(I)-5874456	fwd	5874456	5874461	
RVS_(I)-5877969	rvs	5877964	5877969	
RVS_(II)-5882924	rvs	5882919	5882924	
FWD_(I)-5883321	fwd	5883321	5883326	
FWD_(I)-5883756	fwd	5883756	5883761	
FWD_(I)-5888395	fwd	5888395	5888400	
RVS_(I)-5892590	rvs	5892585	5892590	
RVS_(I)-5894894	rvs	5894889	5894894	
RVS_(III)-5897827	rvs	5897822	5897827	
FWD_(III)-5897905	fwd	5897905	5897910	
RVS_(III)-5904394	rvs	5904389	5904394	
RVS_(I)-5909692	rvs	5909687	5909692	
RVS_(I)-5912259	rvs	5912254	5912259	
FWD_(I)-5922470	fwd	5922470	5922475	
FWD_(I)-5940590	fwd	5940590	5940595	
RVS_(III)-5952014	rvs	5952009	5952014	
RVS_(I)-5959117	rvs	5959112	5959117	
FWD_(III)-5959223	fwd	5959223	5959228	
FWD_(I)-5960950	fwd	5960950	5960955	
FWD_(III)-5961978	fwd	5961978	5961983	
FWD_(I)-5964956	fwd	5964956	5964961	
RVS_(I)-5965135	rvs	5965130	5965135	
FWD_(I)-5971925	fwd	5971925	5971930	
RVS_(I)-5973863	rvs	5973858	5973863	
FWD_(I)-5973921	fwd	5973921	5973926	
FWD_(I)-5976351	fwd	5976351	5976356	
FWD_(I)-5981029	fwd	5981029	5981034	
FWD_(I)-5984729	fwd	5984729	5984734	
FWD_(III)-5988476	fwd	5988476	5988481	
FWD_(III)-5991461	fwd	5991461	5991466	
FWD_(I)-5995757	fwd	5995757	5995762	
FWD_(III)-5999456	fwd	5999456	5999461	
FWD_(I)-6002908	fwd	6002908	6002913	
FWD_(I)-6005616	fwd	6005616	6005621	
FWD_(I)-6006754	fwd	6006754	6006759	
FWD_(I)-6010146	fwd	6010146	6010151	
FWD_(I)-6012688	fwd	6012688	6012693	
FWD_(I)-6014903	fwd	6014903	6014908	
FWD_(I)-6016406	fwd	6016406	6016411	
FWD_(I)-6016462	fwd	6016462	6016467	
FWD_(I)-6023430	fwd	6023430	6023435	
FWD_(I)-6025338	fwd	6025338	6025343	SCO5530-6025338
FWD_(I)-6031665	fwd	6031665	6031670	SCO5535-6031665
RVS_(III)-6035868	rvs	6035863	6035868	
RVS_(I)-6038799	rvs	6038794	6038799	
FWD_(I)-6040177	fwd	6040177	6040182	
FWD_(I)-6045079	fwd	6045079	6045084	

FWD_(I)-6045445	fwd	6045445	6045450	
FWD_(I)-6048321	fwd	6048321	6048326	
FWD_(III)-6049064	fwd	6049064	6049069	
FWD_(II)-6049171	fwd	6049171	6049176	
FWD_(II)-6049510	fwd	6049510	6049515	
FWD_(II)-6049542	fwd	6049542	6049547	
FWD_(III)-6051887	fwd	6051887	6051892	
FWD_(III)-6054327	fwd	6054327	6054332	
FWD_(I)-6054328	fwd	6054328	6054333	
RVS_(II)-6056372	rvs	6056367	6056372	
FWD_(I)-6056430	fwd	6056430	6056435	
RVS_(III)-6056664	rvs	6056659	6056664	
RVS_(II)-6059941	rvs	6059936	6059941	
RVS_(III)-6060122	rvs	6060117	6060122	
RVS_(I)-6062375	rvs	6062370	6062375	
RVS_(I)-6062403	rvs	6062398	6062403	
FWD_(III)-6069055	fwd	6069055	6069060	
FWD_(II)-6069394	fwd	6069394	6069399	
RVS_(I)-6072169	rvs	6072164	6072169	
FWD_(I)-6074179	fwd	6074179	6074184	
FWD_(I)-6081765	fwd	6081765	6081770	
RVS_(I)-6083948	rvs	6083943	6083948	
FWD_(I)-6084061	fwd	6084061	6084066	
FWD_(III)-6090346	fwd	6090346	6090351	
RVS_(III)-6095750	rvs	6095745	6095750	
FWD_(I)-6096063	fwd	6096063	6096068	
FWD_(I)-6096339	fwd	6096339	6096344	
FWD_(I)-6097062	fwd	6097062	6097067	
FWD_(I)-6099368	fwd	6099368	6099373	
FWD_(I)-6101525	fwd	6101525	6101530	
FWD_(I)-6104083	fwd	6104083	6104088	
FWD_(I)-6108716	fwd	6108716	6108721	
RVS_(I)-6109957	rvs	6109952	6109957	
RVS_(I)-6110690	rvs	6110685	6110690	
FWD_(I)-6111063	fwd	6111063	6111068	
RVS_(I)-6111563	rvs	6111558	6111563	
RVS_(I)-6117503	rvs	6117498	6117503	
RVS_(I)-6118370	rvs	6118365	6118370	
FWD_(I)-6121990	fwd	6121990	6121995	
FWD_(III)-6123333	fwd	6123333	6123338	rpsB-tsff-6123332(fwd)
FWD_(II)-6124590	fwd	6124590	6124595	
FWD_(I)-6125400	fwd	6125400	6125405	
FWD_(II)-6127169	fwd	6127169	6127174	
FWD_(I)-6131201	fwd	6131201	6131206	
RVS_(III)-6132956	rvs	6132951	6132956	
RVS_(III)-6137095	rvs	6137090	6137095	
FWD_(II)-6137987	fwd	6137987	6137992	
FWD_(III)-6139816	fwd	6139816	6139821	
FWD_(II)-6139890	fwd	6139890	6139895	
FWD_(III)-6140654	fwd	6140654	6140659	
FWD_(I)-6140844	fwd	6140844	6140849	
FWD_(I)-6143016	fwd	6143016	6143021	
RVS_(III)-6151577	rvs	6151572	6151577	
FWD_(III)-6156094	fwd	6156094	6156099	
FWD_(I)-6162748	fwd	6162748	6162753	
RVS_(I)-6170573	rvs	6170568	6170573	
RVS_(I)-6176414	rvs	6176409	6176414	
FWD_(I)-6186588	fwd	6186588	6186593	
RVS_(I)-6201763	rvs	6201758	6201763	

FWD_(III)-6201820	fwd	6201820	6201825		
FWD_(I)-6203922	fwd	6203922	6203927		
FWD_(III)-6206617	fwd	6206617	6206622		
FWD_(I)-6214288	fwd	6214288	6214293		
FWD_(I)-6216001	fwd	6216001	6216006		
FWD_(I)-6216281	fwd	6216281	6216286		
RVS_(I)-6221570	rvs	6221565	6221570		
RVS_(III)-6236874	rvs	6236869	6236874		
FWD_(I)-6238638	fwd	6238638	6238643		
RVS_(III)-6244271	rvs	6244266	6244271	bldBr-6244273(rvs)	
FWD_(III)-6244412	fwd	6244412	6244417		
FWD_(I)-6257499	fwd	6257499	6257504		
FWD_(I)-6259061	fwd	6259061	6259066		
FWD_(I)-6259581	fwd	6259581	6259586		
RVS_(I)-6265644	rvs	6265639	6265644		SCO5742-6265643
FWD_(I)-6265755	fwd	6265755	6265760		
FWD_(III)-6269574	fwd	6269574	6269579		
FWD_(II)-6269747	fwd	6269747	6269752		
FWD_(II)-6270231	fwd	6270231	6270236		
FWD_(II)-6271522	fwd	6271522	6271527		
FWD_(II)-6271576	fwd	6271576	6271581		
FWD_(II)-6273145	fwd	6273145	6273150		
FWD_(II)-6273154	fwd	6273154	6273159		
FWD_(II)-6274229	fwd	6274229	6274234		
FWD_(I)-6275223	fwd	6275223	6275228		
FWD_(I)-6284967	fwd	6284967	6284972		
FWD_(I)-6285148	fwd	6285148	6285153		
FWD_(I)-6288885	fwd	6288885	6288890		
FWD_(I)-6290145	fwd	6290145	6290150		
FWD_(I)-6292172	fwd	6292172	6292177		
FWD_(I)-6293026	fwd	6293026	6293031		
FWD_(I)-6293618	fwd	6293618	6293623		
FWD_(I)-6304454	fwd	6304454	6304459		
FWD_(I)-6306450	fwd	6306450	6306455		
RVS_(I)-6310033	rvs	6310028	6310033		
RVS_(III)-6315599	rvs	6315594	6315599		
FWD_(I)-6321291	fwd	6321291	6321296		
FWD_(I)-6324453	fwd	6324453	6324458		
RVS_(I)-6327478	rvs	6327473	6327478		
FWD_(III)-6327656	fwd	6327656	6327661		
FWD_(I)-6329272	fwd	6329272	6329277		
RVS_(II)-6334530	rvs	6334525	6334530		
RVS_(III)-6338707	rvs	6338702	6338707		
FWD_(I)-6347731	fwd	6347731	6347736		
FWD_(I)-6353795	fwd	6353795	6353800		
FWD_(II)-6355153	fwd	6355153	6355158		
RVS_(I)-6355219	rvs	6355214	6355219		
FWD_(II)-6355467	fwd	6355467	6355472		
FWD_(I)-6356446	fwd	6356446	6356451		SCO5812-6356446
RVS_(I)-6358968	rvs	6358963	6358968		
FWD_(III)-6367510	fwd	6367510	6367515	hrdBf-6367510(fwd)	
RVS_(I)-6370603	rvs	6370598	6370603		
RVS_(I)-6370779	rvs	6370774	6370779		
FWD_(III)-6375779	fwd	6375779	6375784		SCO5826-6375779
RVS_(II)-6378311	rvs	6378306	6378311		
FWD_(II)-6384749	fwd	6384749	6384754		
RVS_(II)-6388921	rvs	6388916	6388921		
FWD_(II)-6389127	fwd	6389127	6389132		
FWD_(I)-6394529	fwd	6394529	6394534		

RVS_(I)-6394796	rvs	6394791	6394796		
FWD_(II)-6397754	fwd	6397754	6397759		
RVS_(II)-6412315	rvs	6412310	6412315		
RVS_(I)-6412514	rvs	6412509	6412514		
FWD_(I)-6412684	fwd	6412684	6412689		
RVS_(I)-6419080	rvs	6419075	6419080		
RVS_(III)-6419109	rvs	6419104	6419109		
FWD_(I)-6419234	fwd	6419234	6419239		
FWD_(II)-6421412	fwd	6421412	6421417		
FWD_(I)-6424274	fwd	6424274	6424279		
FWD_(III)-6426285	fwd	6426285	6426290		
FWD_(I)-6429608	fwd	6429608	6429613		
RVS_(I)-6432311	rvs	6432306	6432311	trkAr-6432313(rvs)	
FWD_(III)-6432601	fwd	6432601	6432606		
RVS_(I)-6438973	rvs	6438968	6438973		
FWD_(II)-6439043	fwd	6439043	6439048		
RVS_(I)-6482627	rvs	6482622	6482627		SCO5914-6482626
FWD_(I)-6484345	fwd	6484345	6484350		
FWD_(II)-6484699	fwd	6484699	6484704		
FWD_(I)-6486266	fwd	6486266	6486271		
RVS_(I)-6489798	rvs	6489793	6489798		
RVS_(I)-6489984	rvs	6489979	6489984		
RVS_(I)-6491282	rvs	6491277	6491282		
FWD_(II)-6500269	fwd	6500269	6500274		
FWD_(I)-6502819	fwd	6502819	6502824		
RVS_(I)-6514555	rvs	6514550	6514555		
FWD_(II)-6517292	fwd	6517292	6517297		
FWD_(I)-6520509	fwd	6520509	6520514		
FWD_(III)-6539241	fwd	6539241	6539246		
FWD_(I)-6541450	fwd	6541450	6541455		
RVS_(I)-6563142	rvs	6563137	6563142		
FWD_(I)-6564778	fwd	6564778	6564783		
FWD_(I)-6575152	fwd	6575152	6575157	acoAf-6575153(fwd)	
FWD_(I)-6578132	fwd	6578132	6578137		
FWD_(II)-6578244	fwd	6578244	6578249		
FWD_(I)-6588177	fwd	6588177	6588182		
FWD_(II)-6589532	fwd	6589532	6589537		
RVS_(I)-6617995	rvs	6617990	6617995		
FWD_(I)-6620001	fwd	6620001	6620006		
FWD_(I)-6632494	fwd	6632494	6632499		
RVS_(I)-6646227	rvs	6646222	6646227		
FWD_(III)-6674122	fwd	6674122	6674127		
FWD_(I)-6674823	fwd	6674823	6674828		
FWD_(III)-6692588	fwd	6692588	6692593		
RVS_(I)-6702820	rvs	6702815	6702820		
RVS_(III)-6705005	rvs	6705000	6705005		
FWD_(I)-6706584	fwd	6706584	6706589		
FWD_(I)-6724307	fwd	6724307	6724312		
RVS_(II)-6725274	rvs	6725269	6725274		
RVS_(I)-6729121	rvs	6729116	6729121		
FWD_(II)-6740466	fwd	6740466	6740471		
RVS_(I)-6744800	rvs	6744795	6744800		
FWD_(I)-6748526	fwd	6748526	6748531		
FWD_(II)-6749509	fwd	6749509	6749514		
RVS_(I)-6753740	rvs	6753735	6753740		
FWD_(I)-6755074	fwd	6755074	6755079		
RVS_(III)-6760356	rvs	6760351	6760356		
RVS_(II)-6767401	rvs	6767396	6767401		
RVS_(I)-6771630	rvs	6771625	6771630		

FWD_(I)-6773663	fwd	6773663	6773668		
FWD_(I)-6783843	fwd	6783843	6783848	cwgf-6783842(fwd)	
RVS_(III)-6804137	rvs	6804132	6804137		
FWD_(II)-6822451	fwd	6822451	6822456		
RVS_(I)-6831956	rvs	6831951	6831956		
RVS_(I)-6860345	rvs	6860340	6860345		
FWD_(I)-6860882	fwd	6860882	6860887		
FWD_(II)-6863826	fwd	6863826	6863831		
FWD_(II)-6868293	fwd	6868293	6868298		
FWD_(III)-6869666	fwd	6869666	6869671		
FWD_(I)-6889567	fwd	6889567	6889572		
RVS_(I)-6891790	rvs	6891785	6891790		
RVS_(II)-6907331	rvs	6907326	6907331		
RVS_(III)-6942568	rvs	6942563	6942568		SCO6282-6942568
RVS_(III)-6972731	rvs	6972726	6972731		
FWD_(II)-6983615	fwd	6983615	6983620		
RVS_(I)-6987789	rvs	6987784	6987789		
RVS_(III)-6988322	rvs	6988317	6988322		
RVS_(II)-6990799	rvs	6990794	6990799		
FWD_(II)-6991231	fwd	6991231	6991236		
RVS_(I)-6994725	rvs	6994720	6994725		
FWD_(I)-7000969	fwd	7000969	7000974		
FWD_(II)-7018971	fwd	7018971	7018976		
FWD_(II)-7030354	fwd	7030354	7030359		
RVS_(I)-7034299	rvs	7034294	7034299		
RVS_(II)-7034396	rvs	7034391	7034396		
RVS_(II)-7036402	rvs	7036397	7036402		
FWD_(I)-7037644	fwd	7037644	7037649		
FWD_(II)-7040304	fwd	7040304	7040309		
RVS_(II)-7045562	rvs	7045557	7045562		
RVS_(II)-7046449	rvs	7046444	7046449		
FWD_(I)-7046588	fwd	7046588	7046593		
FWD_(II)-7047196	fwd	7047196	7047201		
FWD_(I)-7051508	fwd	7051508	7051513		
FWD_(III)-7060389	fwd	7060389	7060394		
RVS_(I)-7060982	rvs	7060977	7060982		
FWD_(III)-7063561	fwd	7063561	7063566		
FWD_(II)-7063621	fwd	7063621	7063626		
RVS_(I)-7065700	rvs	7065695	7065700		
FWD_(III)-7065757	fwd	7065757	7065762		
RVS_(I)-7069584	rvs	7069579	7069584		
RVS_(II)-7122905	rvs	7122900	7122905		
RVS_(I)-7141924	rvs	7141919	7141924		
FWD_(II)-7151586	fwd	7151586	7151591		
RVS_(I)-7157389	rvs	7157384	7157389		
RVS_(II)-7157475	rvs	7157470	7157475		
FWD_(II)-7166743	fwd	7166743	7166748		
FWD_(I)-7174943	fwd	7174943	7174948		
FWD_(I)-7209840	fwd	7209840	7209845		
RVS_(I)-7215990	rvs	7215985	7215990		
RVS_(I)-7266174	rvs	7266169	7266174		
FWD_(III)-7267198	fwd	7267198	7267203		
FWD_(III)-7300564	fwd	7300564	7300569		
FWD_(II)-7310585	fwd	7310585	7310590		
RVS_(I)-7310726	rvs	7310721	7310726		
FWD_(II)-7343041	fwd	7343041	7343046		
RVS_(I)-7347604	rvs	7347599	7347604		
FWD_(II)-7353337	fwd	7353337	7353342		
FWD_(I)-7356693	fwd	7356693	7356698		

FWD_(I)-7358814	fwd	7358814	7358819		SCO6631-7358814
FWD_(I)-7360029	fwd	7360029	7360034		
FWD_(II)-7361954	fwd	7361954	7361959		
RVS_(III)-7402730	rvs	7402725	7402730		
RVS_(I)-7416046	rvs	7416041	7416046		
FWD_(I)-7418587	fwd	7418587	7418592		
FWD_(I)-7446523	fwd	7446523	7446528		
RVS_(I)-7457484	rvs	7457479	7457484		
FWD_(I)-7460584	fwd	7460584	7460589		
RVS_(I)-7467606	rvs	7467601	7467606		
RVS_(I)-7471746	rvs	7471741	7471746		
RVS_(II)-7476905	rvs	7476900	7476905		
RVS_(III)-7478180	rvs	7478175	7478180		
FWD_(II)-7480241	fwd	7480241	7480246		
RVS_(I)-7501881	rvs	7501876	7501881		
RVS_(I)-7506927	rvs	7506922	7506927		
FWD_(I)-7515818	fwd	7515818	7515823		
RVS_(III)-7518822	rvs	7518817	7518822		
RVS_(III)-7530640	rvs	7530635	7530640		
FWD_(I)-7530963	fwd	7530963	7530968		
FWD_(I)-7530975	fwd	7530975	7530980		
FWD_(I)-7532326	fwd	7532326	7532331		
RVS_(II)-7536256	rvs	7536251	7536256		
RVS_(III)-7537174	rvs	7537169	7537174		
FWD_(I)-7555316	fwd	7555316	7555321		
FWD_(I)-7565919	fwd	7565919	7565924		
FWD_(II)-7568116	fwd	7568116	7568121		
RVS_(I)-7570990	rvs	7570985	7570990		
FWD_(II)-7588011	fwd	7588011	7588016		
FWD_(II)-7598968	fwd	7598968	7598973		
RVS_(I)-7671187	rvs	7671182	7671187		
FWD_(II)-7672451	fwd	7672451	7672456		
FWD_(III)-7688336	fwd	7688336	7688341		
FWD_(III)-7713327	fwd	7713327	7713332		
RVS_(I)-7713338	rvs	7713333	7713338		
RVS_(II)-7716410	rvs	7716405	7716410		
FWD_(II)-7717031	fwd	7717031	7717036		
FWD_(II)-7733154	fwd	7733154	7733159		
FWD_(III)-7740458	fwd	7740458	7740463		
RVS_(II)-7760905	rvs	7760900	7760905		SCO6992-7760905
FWD_(II)-7790639	fwd	7790639	7790644		
RVS_(III)-7821798	rvs	7821793	7821798		
FWD_(II)-7832331	fwd	7832331	7832336		
RVS_(I)-7835299	rvs	7835294	7835299		
FWD_(I)-7835391	fwd	7835391	7835396		
RVS_(I)-7842839	rvs	7842834	7842839		
RVS_(I)-7846476	rvs	7846471	7846476		
FWD_(I)-7881832	fwd	7881832	7881837		
FWD_(I)-7888420	fwd	7888420	7888425		
FWD_(II)-7893950	fwd	7893950	7893955		
FWD_(II)-7932022	fwd	7932022	7932027		
RVS_(I)-7932266	rvs	7932261	7932266		
FWD_(II)-7936071	fwd	7936071	7936076		
FWD_(I)-7964759	fwd	7964759	7964764	agl3Rf-7964760(fwd)	
FWD_(II)-7995561	fwd	7995561	7995566		
FWD_(II)-8004770	fwd	8004770	8004775		
RVS_(I)-8006184	rvs	8006179	8006184		
FWD_(II)-8006448	fwd	8006448	8006453		
RVS_(I)-8027679	rvs	8027674	8027679		

FWD_(II)-8031996	fwd	8031996	8032001
RVS_(I)-8062009	rvs	8062004	8062009
FWD_(I)-8062112	fwd	8062112	8062117
RVS_(I)-8079048	rvs	8079043	8079048
FWD_(I)-8084879	fwd	8084879	8084884
RVS_(I)-8086328	rvs	8086323	8086328
RVS_(III)-8086375	rvs	8086370	8086375
FWD_(II)-8092023	fwd	8092023	8092028
RVS_(III)-8107134	rvs	8107129	8107134
RVS_(I)-8117883	rvs	8117878	8117883
RVS_(I)-8139529	rvs	8139524	8139529
FWD_(II)-8150968	fwd	8150968	8150973
FWD_(I)-8165657	fwd	8165657	8165662
FWD_(I)-8175486	fwd	8175486	8175491
FWD_(I)-8194923	fwd	8194923	8194928
FWD_(I)-8197326	fwd	8197326	8197331
FWD_(II)-8224977	fwd	8224977	8224982
FWD_(I)-8284266	fwd	8284266	8284271
RVS_(III)-8294618	rvs	8294613	8294618
FWD_(II)-8308924	fwd	8308924	8308929
RVS_(II)-8315805	rvs	8315800	8315805
RVS_(I)-8326393	rvs	8326388	8326393
FWD_(II)-8329928	fwd	8329928	8329933
FWD_(III)-8352452	fwd	8352452	8352457
FWD_(I)-8355459	fwd	8355459	8355464
RVS_(I)-8361805	rvs	8361800	8361805
RVS_(I)-8363976	rvs	8363971	8363976
RVS_(III)-8372623	rvs	8372618	8372623
FWD_(III)-8398294	fwd	8398294	8398299
FWD_(I)-8402836	fwd	8402836	8402841
RVS_(I)-8442161	rvs	8442156	8442161
FWD_(II)-8444856	fwd	8444856	8444861
RVS_(I)-8471504	rvs	8471499	8471504
FWD_(I)-8488336	fwd	8488336	8488341
RVS_(I)-8490964	rvs	8490959	8490964
FWD_(I)-8531016	fwd	8531016	8531021
FWD_(II)-8541365	fwd	8541365	8541370
FWD_(II)-8552486	fwd	8552486	8552491
FWD_(I)-8557437	fwd	8557437	8557442
FWD_(II)-8578376	fwd	8578376	8578381
FWD_(III)-8594978	fwd	8594978	8594983
RVS_(I)-8597411	rvs	8597406	8597411
FWD_(III)-8611606	fwd	8611606	8611611
FWD_(II)-8648065	fwd	8648065	8648070
RVS_(I)-8655263	rvs	8655258	8655263
FWD_(II)-8662483	fwd	8662483	8662488

Table S1. Transcription start sites identified for *S. coelicolor* . * data extracted from (Cipriano et al., 2013). ** data extracted from (Vockenhuber *et al.* , 2011).

TSS	Left	Right	Overlap with RegulonDB*
FWD_(I)-148	148	148	YES
FWD_(I)-2581	2581	2581	
RVS_(I)-8054	8053	8054	
FWD_(III)-8191	8191	8191	YES
FWD_(I)-9277	9277	9277	
RVS_(II)-10477	10477	10477	
RVS_(II)-10990	10990	10990	
FWD_(I)-12048	12048	12048	YES
FWD_(II)-12121	12121	12121	YES
RVS_(II)-12136	12136	12136	
FWD_(III)-15387	15387	15387	
RVS_(II)-16950	16950	16950	
FWD_(I)-16952	16952	16952	
RVS_(I)-17152	17152	17152	
FWD_(II)-17981	17981	17981	
FWD_(II)-18023	18023	18025	
FWD_(II)-18553	18553	18553	
FWD_(II)-19187	19187	19187	
FWD_(II)-19486	19486	19486	
RVS_(II)-19512	19512	19512	
FWD_(II)-19831	19831	19831	
RVS_(II)-20681	20681	20681	
RVS_(I)-21120	21119	21120	YES
RVS_(I)-21208	21208	21208	YES
FWD_(I)-21383	21383	21383	YES
FWD_(II)-21834	21834	21835	YES
FWD_(II)-25014	25014	25014	YES
FWD_(II)-27114	27114	27114	
FWD_(II)-27263	27263	27263	
FWD_(III)-28287	28287	28287	YES
FWD_(II)-29129	29129	29129	
FWD_(I)-34218	34218	34218	YES
FWD_(II)-34492	34492	34492	
RVS_(II)-34858	34858	34858	
RVS_(II)-39940	39940	39940	
RVS_(II)-46142	46142	46142	
RVS_(II)-51951	51950	51951	
RVS_(II)-52035	52035	52035	
RVS_(II)-52588	52588	52588	YES
RVS_(II)-53777	53777	53777	
RVS_(I)-57242	57242	57242	YES
RVS_(I)-57254	57254	57254	
FWD_(III)-57261	57261	57264	
RVS_(II)-58727	58727	58727	
RVS_(II)-59151	59150	59151	
RVS_(II)-63358	63358	63358	YES
RVS_(II)-65804	65804	65804	
RVS_(II)-65947	65947	65947	
FWD_(III)-66580	66580	66580	
RVS_(III)-66815	66812	66815	
FWD_(I)-70221	70221	70221	
FWD_(III)-72132	72132	72135	
RVS_(I)-75609	75609	75609	YES
FWD_(I)-77367	77367	77367	YES
FWD_(II)-78332	78332	78332	
RVS_(I)-83736	83736	83736	YES
RVS_(II)-83896	83895	83896	
FWD_(II)-84808	84808	84808	

RVS_(II)-84904	84904	84904	
FWD_(II)-85767	85767	85767	
FWD_(II)-87847	87847	87847	
FWD_(I)-87969	87969	87969	
FWD_(I)-89590	89590	89590	YES
FWD_(III)-92485	92485	92485	
FWD_(II)-92626	92626	92626	
FWD_(II)-97673	97673	97673	
FWD_(II)-102454	102454	102454	
FWD_(II)-102744	102744	102745	YES
FWD_(II)-102872	102872	102872	YES
FWD_(II)-104636	104636	104636	YES
FWD_(II)-106502	106502	106508	
FWD_(II)-106746	106746	106746	
FWD_(I)-107668	107668	107668	
FWD_(II)-107697	107697	107697	
RVS_(II)-111829	111829	111829	
RVS_(II)-113200	113200	113200	
RVS_(II)-113213	113213	113213	
FWD_(I)-113394	113394	113394	
FWD_(II)-116380	116380	116380	
RVS_(II)-117052	117052	117052	
RVS_(II)-118680	118680	118680	
FWD_(I)-118703	118703	118703	
FWD_(II)-122034	122034	122034	YES
FWD_(III)-122852	122852	122852	
FWD_(II)-126733	126733	126733	
FWD_(II)-127637	127637	127637	
FWD_(II)-127718	127718	127718	YES
FWD_(II)-130200	130200	130202	
FWD_(II)-130935	130935	130935	
FWD_(III)-131519	131519	131520	YES
FWD_(II)-132455	132455	132455	
FWD_(I)-134339	134339	134339	YES
RVS_(II)-135547	135547	135547	
RVS_(II)-136378	136378	136378	
RVS_(I)-136942	136940	136942	YES
FWD_(I)-141360	141360	141361	YES
FWD_(II)-141650	141650	141650	
FWD_(II)-142292	142292	142292	
RVS_(I)-142704	142704	142704	YES
FWD_(I)-144560	144560	144560	
FWD_(II)-145610	145610	145610	
RVS_(I)-146755	146755	146755	
FWD_(II)-146793	146793	146793	
FWD_(I)-147583	147583	147584	
FWD_(II)-147880	147880	147880	
RVS_(II)-148855	148854	148855	
RVS_(III)-149632	149628	149632	
FWD_(II)-150548	150548	150548	
RVS_(II)-154860	154860	154860	
RVS_(II)-155277	155277	155277	
RVS_(II)-156425	156425	156425	
RVS_(II)-159173	159173	159173	YES
RVS_(II)-160468	160468	160468	
RVS_(II)-160658	160658	160658	YES
RVS_(II)-160695	160695	160695	
RVS_(II)-161059	161059	161059	
RVS_(II)-161143	161143	161143	

RVS_(I)-161380	161378	161380	
FWD_(II)-164235	164235	164235	
FWD_(I)-169124	169124	169124	
FWD_(II)-169215	169215	169215	
FWD_(II)-169506	169506	169506	
FWD_(II)-169546	169546	169546	
RVS_(II)-169816	169816	169816	
RVS_(II)-174920	174920	174920	YES
FWD_(I)-174933	174933	174933	
RVS_(III)-174995	174990	174995	
FWD_(II)-175012	175012	175012	
FWD_(II)-176558	176558	176558	YES
RVS_(II)-179182	179182	179182	YES
RVS_(II)-181049	181049	181049	
FWD_(I)-182447	182447	182447	YES
FWD_(II)-182508	182508	182508	
RVS_(II)-184001	184001	184001	
RVS_(I)-184125	184125	184125	
RVS_(I)-184194	184194	184194	
RVS_(I)-185979	185977	185979	YES
RVS_(I)-189554	189554	189554	
FWD_(III)-189712	189712	189713	YES
FWD_(I)-192615	192615	192617	
FWD_(II)-192814	192814	192814	
FWD_(II)-194201	194201	194201	
FWD_(II)-194466	194466	194466	
FWD_(II)-194669	194669	194669	
FWD_(I)-194764	194764	194764	
FWD_(I)-194784	194784	194784	YES
FWD_(I)-196159	196159	196159	
FWD_(II)-198185	198185	198185	
FWD_(II)-199299	199299	199299	
FWD_(II)-199671	199671	199671	
FWD_(II)-199980	199980	199980	
FWD_(II)-200399	200399	200399	
FWD_(II)-201484	201484	201484	
FWD_(II)-201715	201715	201715	
FWD_(II)-202110	202110	202110	
FWD_(II)-202518	202518	202518	
FWD_(II)-208276	208276	208276	
FWD_(II)-208326	208326	208326	
FWD_(II)-208411	208411	208411	YES
FWD_(II)-211967	211967	211967	
FWD_(II)-213611	213611	213611	
RVS_(I)-214198	214198	214198	
FWD_(I)-214269	214269	214269	
FWD_(II)-214988	214988	214989	
FWD_(III)-216051	216051	216053	
RVS_(I)-216167	216166	216167	
RVS_(I)-216327	216327	216327	
RVS_(III)-218838	218838	218838	YES
FWD_(II)-220023	220023	220023	
RVS_(II)-221630	221630	221630	
FWD_(I)-222806	222806	222806	YES
FWD_(I)-223485	223485	223485	
FWD_(II)-223594	223594	223595	
FWD_(II)-225018	225018	225018	
FWD_(II)-226160	226160	226160	
FWD_(II)-227393	227393	227393	

FWD_(II)-227865	227865	227865	
FWD_(II)-228372	228372	228372	
FWD_(I)-231063	231063	231063	YES
FWD_(I)-232367	232367	232367	
FWD_(II)-232414	232414	232414	
FWD_(II)-234288	234288	234288	
FWD_(II)-234785	234785	234785	
FWD_(I)-236910	236910	236910	
RVS_(II)-240217	240217	240217	YES
FWD_(I)-243512	243512	243512	
RVS_(I)-246534	246533	246534	
FWD_(III)-248144	248144	248144	
FWD_(III)-253340	253340	253340	
RVS_(I)-255810	255808	255810	YES
FWD_(II)-257810	257810	257810	YES
FWD_(II)-257905	257905	257905	
FWD_(II)-259461	259461	259461	
FWD_(I)-259574	259574	259574	
FWD_(I)-262063	262063	262063	
RVS_(II)-262243	262243	262243	
RVS_(II)-262303	262303	262303	
FWD_(II)-268228	268228	268228	
FWD_(II)-268350	268350	268350	
FWD_(III)-269812	269812	269812	
FWD_(II)-270874	270874	270874	
RVS_(III)-274374	274372	274374	
RVS_(II)-277022	277022	277022	
FWD_(II)-279224	279224	279224	
FWD_(I)-291112	291112	291112	
FWD_(II)-292365	292365	292365	
FWD_(II)-293211	293211	293211	
RVS_(III)-294845	294845	294845	
RVS_(II)-295194	295194	295194	
FWD_(III)-296425	296425	296425	
FWD_(II)-302198	302198	302198	
RVS_(II)-302911	302911	302911	
FWD_(II)-302922	302922	302922	
FWD_(II)-303970	303970	303970	
FWD_(II)-306913	306913	306913	
RVS_(II)-320200	320200	320200	
FWD_(I)-320708	320708	320708	
RVS_(I)-333696	333696	333696	
FWD_(III)-338972	338972	338975	
RVS_(III)-339343	339340	339343	
FWD_(I)-344476	344476	344478	
FWD_(I)-345656	345656	345656	YES
FWD_(III)-348851	348851	348851	
FWD_(III)-353860	353860	353862	
FWD_(III)-356701	356701	356709	
FWD_(III)-356900	356900	356905	
FWD_(III)-358954	358954	358954	
FWD_(II)-361116	361116	361116	
FWD_(II)-371000	371000	371000	
FWD_(III)-374140	374140	374145	
RVS_(III)-374548	374545	374548	
FWD_(III)-375954	375954	375954	
FWD_(II)-376766	376766	376766	
RVS_(II)-376816	376816	376816	
RVS_(II)-379926	379926	379926	

FWD_(III)-380546	380546	380547	
FWD_(II)-380863	380863	380863	
RVS_(I)-388979	388979	388979	
RVS_(II)-390222	390222	390222	
FWD_(II)-390603	390603	390603	
FWD_(II)-390637	390637	390637	
RVS_(II)-392204	392204	392204	
FWD_(I)-393639	393639	393639	
FWD_(II)-397063	397063	397063	
FWD_(II)-398668	398668	398668	
RVS_(III)-400179	400175	400179	
FWD_(III)-400542	400542	400542	YES
FWD_(I)-400587	400587	400587	YES
FWD_(II)-400746	400746	400746	
RVS_(II)-401913	401913	401913	
RVS_(II)-402921	402921	402921	
RVS_(II)-402929	402929	402929	
FWD_(II)-404965	404965	404965	
FWD_(II)-406178	406178	406178	
FWD_(II)-406972	406972	406972	
FWD_(I)-407372	407372	407372	YES
FWD_(II)-407798	407798	407798	
FWD_(III)-410300	410300	410302	
RVS_(III)-410495	410495	410495	
FWD_(II)-413091	413091	413091	
FWD_(II)-415586	415586	415586	
FWD_(II)-416325	416325	416325	
FWD_(II)-418643	418643	418643	
RVS_(II)-419991	419991	419991	
FWD_(II)-423644	423644	423644	
FWD_(II)-425279	425279	425279	YES
FWD_(III)-425998	425998	426003	
FWD_(II)-426339	426339	426339	
FWD_(II)-426428	426428	426428	YES
RVS_(I)-431316	431316	431316	YES
FWD_(III)-432198	432198	432201	
FWD_(I)-435805	435805	435805	
FWD_(I)-437386	437386	437386	
RVS_(I)-437510	437509	437510	
RVS_(II)-440598	440598	440598	
RVS_(II)-443785	443785	443785	
FWD_(II)-443827	443827	443827	
FWD_(I)-443882	443882	443882	YES
RVS_(II)-445912	445912	445912	YES
RVS_(II)-449847	449847	449847	
RVS_(I)-450878	450878	450878	YES
FWD_(I)-453575	453575	453575	YES
FWD_(II)-453658	453658	453658	YES
FWD_(I)-454217	454217	454219	YES
FWD_(I)-455778	455778	455778	
FWD_(II)-455801	455801	455801	YES
FWD_(I)-458039	458039	458039	
FWD_(I)-460556	460556	460556	YES
FWD_(I)-460619	460619	460621	YES
FWD_(II)-460768	460768	460768	
FWD_(II)-460950	460950	460953	
FWD_(I)-461077	461077	461077	
RVS_(II)-463620	463620	463620	
FWD_(I)-475646	475646	475646	

RVS_(II)-477837	477837	477837	
RVS_(II)-477867	477867	477867	
RVS_(II)-478513	478513	478513	YES
RVS_(II)-479648	479648	479648	
RVS_(II)-479726	479726	479726	
RVS_(I)-480020	480020	480020	YES
RVS_(III)-485205	485205	485205	
FWD_(I)-493101	493101	493101	
FWD_(II)-494328	494328	494328	
FWD_(III)-496357	496357	496357	
FWD_(II)-497255	497255	497255	YES
FWD_(III)-500636	500636	500637	
FWD_(III)-504106	504106	504112	
FWD_(III)-506426	506426	506426	YES
RVS_(II)-507322	507322	507322	
FWD_(III)-507804	507804	507804	
RVS_(III)-508044	508041	508044	
RVS_(II)-515050	515050	515050	
FWD_(II)-518613	518613	518613	
FWD_(II)-528252	528252	528254	
FWD_(III)-532188	532188	532189	
FWD_(I)-532207	532207	532207	
FWD_(I)-532855	532855	532855	
FWD_(III)-542360	542360	542360	
FWD_(III)-542376	542376	542376	
FWD_(III)-550574	550574	550574	
RVS_(III)-550737	550733	550737	
RVS_(II)-553247	553247	553247	
RVS_(I)-553716	553714	553716	
RVS_(II)-556248	556248	556248	
RVS_(III)-557005	557005	557005	YES
FWD_(I)-563921	563921	563922	
RVS_(I)-565237	565237	565237	
FWD_(II)-576081	576081	576081	
RVS_(I)-576094	576094	576094	YES
FWD_(III)-576438	576438	576438	
FWD_(I)-576452	576452	576453	
RVS_(II)-577166	577166	577166	
FWD_(II)-579199	579199	579199	
FWD_(III)-582520	582520	582525	
RVS_(III)-584889	584886	584889	YES
FWD_(III)-585275	585275	585281	
RVS_(II)-586241	586241	586241	
RVS_(I)-596318	596317	596318	
RVS(III)-596332	596332	596332	
RVS_(II)-596838	596838	596838	
RVS_(II)-603795	603795	603795	
RVS_(II)-603907	603907	603907	
RVS_(I)-607019	607016	607019	
FWD_(III)-607228	607228	607233	
FWD_(III)-609317	609317	609317	
RVS_(III)-609446	609441	609446	
FWD_(II)-631232	631232	631232	
FWD_(III)-631333	631333	631336	
FWD_(II)-636512	636512	636512	
FWD_(I)-637852	637852	637852	
FWD_(I)-637916	637916	637916	YES
FWD_(II)-638054	638054	638058	
FWD_(II)-638144	638144	638144	YES

FWD_(II)-638483	638483	638483	
RVS_(I)-641127	641127	641127	YES
RVS_(II)-644258	644258	644258	YES
FWD_(II)-651822	651822	651822	
RVS_(I)-655247	655247	655247	
FWD_(I)-656473	656473	656473	YES
FWD_(II)-657187	657187	657189	
RVS_(II)-657191	657191	657191	
FWD_(III)-658097	658097	658100	
RVS_(III)-659526	659522	659526	YES
RVS_(II)-661534	661534	661534	
RVS_(II)-664556	664556	664556	
RVS_(II)-666860	666860	666860	
RVS_(II)-668296	668296	668296	YES
RVS_(II)-670126	670126	670126	
RVS_(II)-670303	670303	670303	
RVS_(I)-674146	674146	674146	
FWD_(I)-674216	674216	674216	YES
RVS_(II)-682793	682793	682793	
RVS_(I)-683663	683663	683663	
RVS_(II)-685858	685858	685858	
FWD_(II)-685875	685875	685875	
RVS_(II)-685887	685887	685887	
RVS_(III)-688275	688272	688275	
RVS_(III)-692655	692653	692655	
RVS_(III)-694215	694215	694215	
RVS_(II)-695038	695038	695038	
RVS_(II)-696311	696311	696311	
RVS_(I)-696391	696390	696391	
RVS_(II)-699579	699579	699579	YES
RVS_(I)-701089	701089	701089	YES
RVS_(I)-702934	702930	702934	YES
FWD_(I)-703063	703063	703063	YES
RVS_(II)-705216	705216	705216	
FWD_(I)-705286	705286	705286	YES
FWD_(I)-707459	707459	707459	YES
RVS_(II)-709947	709940	709947	YES
RVS_(II)-710305	710305	710305	
FWD_(II)-710650	710650	710650	
RVS_(III)-710746	710745	710746	YES
FWD_(I)-712073	712073	712074	
FWD_(II)-712169	712169	712169	
RVS_(II)-720996	720996	720996	
FWD_(III)-728295	728295	728305	
FWD_(II)-732683	732683	732683	
FWD_(II)-734924	734924	734924	
FWD_(II)-738203	738203	738203	YES
FWD_(II)-739655	739655	739655	
FWD_(I)-742021	742021	742021	YES
RVS_(II)-745110	745110	745110	
RVS_(I)-747027	747027	747027	
RVS_(II)-753907	753907	753907	
RVS_(I)-753945	753945	753945	
FWD_(III)-754174	754174	754182	YES
RVS_(III)-757752	757748	757752	
FWD_(III)-757806	757806	757810	YES
FWD_(I)-764295	764295	764295	
FWD_(II)-767999	767999	767999	
RVS_(II)-770069	770069	770069	

FWD_(I)-770393	770393	770393	YES
FWD_(II)-770427	770427	770427	
FWD_(II)-770436	770436	770436	YES
FWD_(I)-770507	770507	770509	YES
FWD_(II)-770592	770592	770592	YES
FWD_(II)-773934	773934	773934	
FWD_(II)-774023	774023	774023	
FWD_(II)-776924	776924	776924	
FWD_(II)-777352	777352	777352	
FWD_(III)-778044	778044	778049	
FWD_(I)-779735	779735	779735	
FWD_(II)-784815	784815	784815	YES
RVS_(II)-786220	786220	786220	
RVS_(II)-786856	786856	786856	YES
RVS_(II)-786894	786894	786894	YES
FWD_(II)-788960	788960	788960	
RVS_(I)-791305	791305	791305	YES
FWD_(II)-793803	793803	793803	
FWD_(I)-793974	793974	793976	
FWD_(I)-794219	794219	794219	
FWD_(II)-794815	794815	794815	
FWD_(I)-797506	797506	797506	
RVS_(I)-802660	802660	802660	
FWD_(III)-805167	805167	805177	
RVS_(II)-805320	805320	805320	
RVS_(III)-806547	806547	806547	YES
RVS_(I)-807153	807153	807153	
FWD_(II)-812375	812375	812375	YES
FWD_(I)-816050	816050	816052	YES
FWD_(II)-816137	816137	816137	YES
FWD_(II)-818867	818867	818869	
FWD_(II)-818868	818868	818875	
FWD_(I)-819081	819081	819081	
FWD_(II)-819904	819904	819904	
FWD_(II)-820538	820538	820538	
FWD_(I)-823824	823824	823824	YES
RVS_(II)-829894	829894	829894	
FWD_(III)-831498	831498	831500	
RVS_(I)-831647	831647	831647	
RVS_(II)-832166	832166	832166	
RVS_(II)-835530	835530	835530	
FWD_(III)-836802	836802	836806	
RVS_(II)-837439	837439	837439	
RVS_(II)-837706	837706	837706	
RVS_(II)-847271	847271	847271	YES
RVS_(III)-847407	847403	847407	
RVS_(I)-848174	848174	848174	YES
FWD_(I)-849436	849436	849436	YES
FWD_(I)-849640	849640	849640	YES
RVS_(I)-852271	852271	852271	
FWD_(II)-852360	852360	852360	
FWD_(II)-853968	853968	853968	
FWD_(II)-853983	853983	853983	
RVS_(III)-856941	856937	856941	
RVS_(I)-865618	865618	865618	YES
FWD_(II)-865720	865720	865720	
RVS_(II)-869394	869393	869394	
FWD_(II)-872122	872122	872122	
RVS_(II)-874892	874890	874892	

FWD_(I)-877440	877440	877440	
RVS_(II)-879743	879743	879743	YES
FWD_(I)-879841	879841	879841	
RVS_(II)-881241	881241	881241	
RVS_(I)-887281	887281	887281	YES
FWD_(I)-889110	889110	889111	
RVS_(III)-890000	890000	890000	YES
FWD_(I)-890115	890115	890115	YES
FWD_(II)-891158	891158	891158	
FWD_(II)-897171	897171	897171	
RVS_(II)-897702	897702	897702	
RVS_(II)-902994	902994	902994	YES
RVS_(II)-903746	903746	903746	YES
FWD_(I)-903754	903754	903754	
RVS_(II)-908523	908523	908523	
RVS_(II)-911632	911631	911632	
FWD_(II)-914241	914241	914241	
RVS_(I)-914525	914525	914525	
FWD_(II)-915663	915663	915663	
FWD_(II)-920708	920708	920708	
RVS_(I)-921902	921900	921902	YES
FWD_(III)-922052	922052	922052	
FWD_(I)-922301	922301	922301	
FWD_(II)-923449	923449	923449	
RVS_(I)-925233	925230	925233	
RVS_(II)-925702	925702	925702	YES
RVS_(II)-925908	925908	925908	YES
RVS_(III)-931302	931302	931302	YES
RVS_(II)-931481	931481	931481	
FWD_(III)-931550	931550	931552	YES
FWD_(II)-932358	932358	932359	YES
FWD_(I)-938588	938588	938588	YES
FWD_(I)-940046	940046	940046	YES
RVS_(II)-944840	944840	944840	
RVS_(II)-946316	946315	946316	
RVS_(II)-948822	948822	948822	
FWD_(II)-949501	949501	949501	
RVS_(II)-950450	950450	950450	
RVS_(II)-951138	951138	951138	
RVS_(II)-952121	952121	952121	
RVS_(I)-953716	953716	953716	YES
RVS_(II)-955414	955414	955414	
FWD_(II)-956792	956792	956792	
FWD_(II)-956802	956802	956802	
FWD_(I)-960941	960941	960941	YES
FWD_(I)-961085	961085	961085	YES
RVS_(II)-961674	961674	961674	
FWD_(II)-962586	962586	962586	
FWD_(II)-962698	962698	962698	
FWD_(I)-969867	969867	969867	YES
FWD_(I)-970950	970950	970950	
FWD_(III)-972721	972721	972721	
RVS_(II)-978033	978033	978033	
FWD_(II)-979499	979499	979499	
RVS_(II)-980063	980063	980063	
FWD_(II)-980111	980111	980111	
FWD_(II)-980154	980154	980154	YES
FWD_(II)-980263	980263	980263	
FWD_(II)-982246	982246	982246	YES

FWD_(II)-982798	982798	982798	
RVS_(III)-984969	984966	984969	YES
RVS_(III)-986318	986314	986318	YES
RVS_(I)-988268	988268	988268	YES
RVS_(II)-988493	988493	988493	
RVS_(II)-989638	989638	989638	YES
FWD_(I)-989738	989738	989738	
FWD_(I)-1005076	1005076	1005076	
FWD_(II)-1007053	1007053	1007053	
FWD_(II)-1010147	1010147	1010147	
FWD_(II)-1012777	1012777	1012777	
RVS_(II)-1014804	1014802	1014804	
FWD_(III)-1014873	1014873	1014873	
RVS_(I)-1015721	1015721	1015721	
RVS_(II)-1016573	1016573	1016573	
FWD_(II)-1017067	1017067	1017067	
FWD_(III)-1017686	1017686	1017686	YES
RVS_(I)-1019410	1019410	1019410	YES
FWD_(I)-1019524	1019524	1019524	
RVS_(II)-1020173	1020173	1020173	YES
FWD_(II)-1020306	1020306	1020306	
FWD_(II)-1020880	1020880	1020880	
RVS_(II)-1021609	1021609	1021609	
RVS_(I)-1026263	1026262	1026263	YES
FWD_(II)-1027150	1027150	1027150	
RVS_(I)-1028091	1028091	1028091	
FWD_(II)-1028976	1028976	1028976	
FWD_(II)-1029185	1029185	1029185	
RVS_(I)-1030674	1030670	1030674	YES
RVS_(I)-1030942	1030941	1030942	
RVS_(II)-1041270	1041265	1041270	
RVS_(I)-1041279	1041279	1041279	
FWD_(II)-1043279	1043279	1043279	
RVS_(I)-1049927	1049923	1049927	
RVS_(I)-1049936	1049936	1049936	
FWD_(II)-1050528	1050528	1050528	
FWD_(I)-1051218	1051218	1051218	
FWD_(II)-1053001	1053001	1053001	
FWD_(II)-1056117	1056117	1056117	
RVS_(I)-1063057	1063057	1063057	YES
RVS_(II)-1063261	1063261	1063261	
FWD_(I)-1064782	1064782	1064782	YES
RVS_(I)-1066982	1066981	1066982	YES
FWD_(I)-1067285	1067285	1067285	
FWD_(II)-1067564	1067564	1067564	
FWD_(III)-1067589	1067589	1067594	
FWD_(III)-1067637	1067637	1067637	
FWD_(II)-1073443	1073443	1073443	
FWD_(II)-1092063	1092063	1092064	YES
FWD_(II)-1092762	1092762	1092762	
RVS_(I)-1096903	1096903	1096903	
FWD_(II)-1098493	1098493	1098493	
FWD_(II)-1099519	1099519	1099519	
RVS_(II)-1100216	1100216	1100216	
FWD_(I)-1104426	1104426	1104426	
RVS_(II)-1105346	1105346	1105346	
FWD_(II)-1105515	1105515	1105515	YES
FWD_(I)-1108501	1108501	1108501	YES
RVS_(II)-1115839	1115839	1115839	

RVS_(II)-1119833	1119833	1119833	YES
RVS_(I)-1120244	1120244	1120244	YES
RVS_(I)-1120253	1120253	1120253	YES
FWD_(II)-1120574	1120574	1120574	
RVS_(II)-1120731	1120731	1120731	
RVS_(II)-1124578	1124578	1124578	
FWD_(III)-1124689	1124689	1124690	
FWD_(I)-1124745	1124745	1124745	
FWD_(II)-1128457	1128457	1128457	
RVS_(II)-1129390	1129388	1129390	
RVS_(I)-1130109	1130109	1130109	
FWD_(I)-1130215	1130215	1130216	
FWD_(II)-1137576	1137576	1137576	
RVS_(I)-1143952	1143952	1143952	YES
FWD_(I)-1145872	1145872	1145873	YES
FWD_(II)-1146015	1146015	1146015	YES
FWD_(II)-1146291	1146291	1146291	
FWD_(II)-1147613	1147613	1147613	
FWD_(II)-1150157	1150157	1150157	
FWD_(II)-1150180	1150180	1150180	
FWD_(II)-1150734	1150734	1150734	
FWD_(II)-1150799	1150799	1150799	YES
FWD_(II)-1152504	1152504	1152504	
FWD_(II)-1154111	1154111	1154111	
FWD_(III)-1156988	1156988	1156991	YES
FWD_(III)-1161050	1161050	1161050	
FWD_(I)-1164239	1164239	1164239	
RVS_(II)-1168086	1168086	1168086	
FWD_(II)-1174624	1174624	1174624	
FWD_(II)-1182082	1182082	1182082	
RVS_(III)-1182136	1182133	1182136	
RVS_(II)-1184884	1184884	1184884	
FWD_(III)-1184961	1184961	1184961	
FWD_(I)-1185046	1185046	1185046	YES
RVS_(II)-1189197	1189192	1189197	
RVS_(I)-1189698	1189694	1189698	YES
RVS_(I)-1189707	1189707	1189707	YES
FWD_(I)-1194184	1194184	1194184	YES
FWD_(III)-1194231	1194231	1194231	YES
RVS_(II)-1196078	1196071	1196078	
FWD_(II)-1200691	1200691	1200691	
RVS_(I)-1202157	1202157	1202157	
RVS_(III)-1203046	1203043	1203046	
RVS_(II)-1203132	1203132	1203132	
FWD_(II)-1207102	1207102	1207102	
RVS_(II)-1212360	1212360	1212360	
RVS_(II)-1212504	1212504	1212504	
RVS_(I)-1213336	1213336	1213336	YES
RVS_(II)-1217962	1217962	1217962	
RVS_(II)-1223595	1223595	1223595	
RVS_(I)-1225365	1225362	1225365	
FWD_(II)-1225387	1225387	1225387	
FWD_(II)-1225704	1225704	1225704	
FWD_(II)-1228020	1228020	1228020	
RVS_(II)-1232282	1232279	1232282	
RVS_(II)-1233990	1233989	1233990	
FWD_(I)-1234128	1234128	1234128	
RVS_(II)-1236509	1236509	1236509	YES
FWD_(II)-1241244	1241244	1241244	

RVS_(II)-1243779	1243779	1243779	
FWD_(II)-1243901	1243901	1243901	
RVS_(III)-1250132	1250128	1250132	
FWD_(II)-1250212	1250212	1250212	
FWD_(II)-1252179	1252179	1252179	
RVS_(II)-1257105	1257105	1257105	YES
FWD_(I)-1257961	1257961	1257963	YES
RVS_(I)-1261401	1261400	1261401	YES
FWD_(II)-1262806	1262806	1262806	YES
FWD_(II)-1267079	1267079	1267079	YES
FWD_(III)-1267250	1267250	1267260	
FWD_(II)-1268173	1268173	1268173	
RVS_(II)-1268510	1268510	1268510	
FWD_(I)-1268546	1268546	1268546	
FWD_(III)-1268861	1268861	1268868	
FWD_(I)-1269081	1269081	1269081	
FWD_(III)-1269385	1269385	1269390	
RVS_(II)-1269580	1269580	1269580	
FWD_(I)-1269616	1269616	1269616	
RVS_(II)-1272547	1272547	1272547	
RVS_(I)-1272852	1272852	1272852	
RVS_(I)-1276909	1276909	1276909	
FWD_(I)-1277155	1277155	1277155	YES
RVS_(III)-1278688	1278684	1278688	
FWD_(I)-1279029	1279029	1279030	YES
RVS_(I)-1286890	1286889	1286890	YES
FWD_(I)-1288317	1288317	1288317	
FWD_(I)-1288329	1288329	1288329	YES
RVS_(I)-1288401	1288401	1288401	YES
FWD_(I)-1289400	1289400	1289400	
FWD_(III)-1290565	1290565	1290570	
FWD_(II)-1291105	1291105	1291105	
RVS_(III)-1292184	1292180	1292184	YES
FWD_(II)-1292716	1292716	1292716	
RVS_(I)-1297533	1297533	1297533	YES
RVS_(II)-1297695	1297695	1297695	
FWD_(I)-1298694	1298694	1298695	
FWD_(II)-1298939	1298939	1298939	
FWD_(I)-1306787	1306787	1306788	
RVS_(II)-1308374	1308374	1308374	
FWD_(II)-1308499	1308499	1308499	
RVS_(I)-1308917	1308917	1308917	YES
FWD_(II)-1310323	1310323	1310323	
RVS_(II)-1311379	1311379	1311379	
FWD_(III)-1312015	1312015	1312015	YES
FWD_(I)-1321789	1321789	1321789	
FWD_(I)-1324724	1324724	1324725	
RVS_(II)-1324826	1324826	1324826	
FWD_(I)-1327326	1327326	1327326	
RVS_(II)-1328712	1328711	1328712	
RVS_(I)-1328739	1328739	1328739	
FWD_(II)-1328906	1328906	1328906	YES
FWD_(III)-1331782	1331782	1331788	
FWD_(II)-1332812	1332812	1332812	
RVS_(II)-1332972	1332972	1332972	
FWD_(I)-1333114	1333114	1333114	
FWD_(II)-1336679	1336679	1336679	
RVS_(I)-1337215	1337213	1337215	YES
FWD_(II)-1337333	1337333	1337333	

RVS_(II)-1337714	1337713	1337714	
FWD_(II)-1338211	1338211	1338212	
FWD_(II)-1342751	1342751	1342751	
RVS_(II)-1349146	1349145	1349146	YES
FWD_(II)-1349701	1349701	1349701	
FWD_(II)-1349814	1349814	1349814	
RVS_(II)-1352574	1352574	1352574	
RVS_(II)-1355288	1355288	1355288	
RVS_(II)-1355850	1355850	1355850	
RVS_(II)-1355950	1355950	1355950	
RVS_(II)-1357065	1357065	1357065	
FWD_(III)-1366055	1366055	1366057	YES
FWD_(I)-1367686	1367686	1367686	
FWD_(I)-1384715	1384715	1384715	YES
RVS_(I)-1386869	1386869	1386869	YES
FWD_(II)-1389565	1389565	1389565	
FWD_(II)-1391141	1391141	1391141	
FWD_(I)-1391220	1391220	1391220	
FWD_(I)-1394073	1394073	1394073	
FWD_(II)-1394109	1394109	1394109	
FWD_(I)-1395352	1395352	1395352	
RVS_(I)-1396686	1396684	1396686	YES
RVS_(I)-1397577	1397577	1397577	
RVS_(II)-1398310	1398310	1398310	YES
RVS_(I)-1403777	1403776	1403777	
FWD_(II)-1405985	1405985	1405985	
FWD_(II)-1406048	1406048	1406048	YES
FWD_(I)-1407153	1407153	1407153	YES
RVS_(II)-1409463	1409463	1409463	
FWD_(II)-1414535	1414535	1414535	
FWD_(II)-1415269	1415269	1415269	
FWD_(I)-1415447	1415447	1415447	
FWD_(II)-1416670	1416670	1416670	
RVS_(I)-1418267	1418266	1418267	
RVS_(II)-1423415	1423415	1423415	
RVS_(II)-1424998	1424998	1424998	
FWD_(II)-1431042	1431042	1431042	
RVS_(III)-1433666	1433665	1433666	YES
RVS_(II)-1437029	1437029	1437029	
RVS_(I)-1438870	1438870	1438870	
FWD_(III)-1439047	1439047	1439047	
RVS_(II)-1461537	1461537	1461537	
RVS_(III)-1467202	1467200	1467202	
FWD_(III)-1467367	1467367	1467367	
FWD_(II)-1468427	1468427	1468427	
RVS_(II)-1468786	1468786	1468786	
FWD_(II)-1468803	1468803	1468803	
FWD_(II)-1471283	1471283	1471283	
FWD_(II)-1472213	1472213	1472213	
FWD_(III)-1486217	1486217	1486217	YES
RVS_(III)-1488779	1488776	1488779	
FWD_(II)-1489298	1489298	1489298	
RVS_(II)-1489530	1489528	1489530	YES
RVS_(I)-1489551	1489545	1489551	
RVS_(II)-1489626	1489626	1489626	
FWD_(I)-1490143	1490143	1490143	
RVS_(III)-1490242	1490240	1490242	
RVS_(II)-1490328	1490328	1490328	
FWD_(II)-1493289	1493289	1493289	

FWD_(II)-1493413	1493413	1493413	
FWD_(II)-1495767	1495767	1495767	
FWD_(II)-1495856	1495856	1495856	
FWD_(I)-1496631	1496631	1496631	
FWD_(II)-1496853	1496853	1496853	
FWD_(II)-1501681	1501681	1501681	
RVS_(II)-1508186	1508186	1508186	
FWD_(II)-1509620	1509620	1509620	
RVS_(II)-1515147	1515147	1515147	
RVS_(II)-1515174	1515174	1515174	
RVS_(II)-1515242	1515241	1515242	
RVS_(III)-1515274	1515271	1515274	
FWD_(II)-1515287	1515287	1515287	
FWD_(III)-1515636	1515636	1515636	
FWD_(II)-1517023	1517023	1517023	
RVS_(II)-1517891	1517891	1517891	
RVS_(II)-1520315	1520315	1520315	
FWD_(II)-1521251	1521251	1521251	YES
FWD_(II)-1524763	1524763	1524763	
FWD_(III)-1545394	1545394	1545397	YES
RVS_(I)-1553764	1553764	1553764	
RVS_(II)-1554072	1554072	1554072	YES
FWD_(I)-1554621	1554621	1554621	
RVS_(II)-1566875	1566875	1566875	
FWD_(II)-1568523	1568523	1568523	
RVS_(II)-1582456	1582456	1582456	
FWD_(II)-1582537	1582537	1582537	
RVS_(II)-1590505	1590505	1590505	YES
FWD_(II)-1591680	1591680	1591681	
RVS_(II)-1593555	1593555	1593555	
RVS_(II)-1599273	1599273	1599273	
RVS_(II)-1604133	1604133	1604133	
RVS_(II)-1606501	1606501	1606501	
RVS_(II)-1606821	1606821	1606821	
RVS_(II)-1609886	1609886	1609886	
FWD_(I)-1620611	1620611	1620611	YES
RVS_(III)-1620939	1620939	1620939	YES
RVS_(II)-1621902	1621902	1621902	YES
RVS_(II)-1623390	1623390	1623390	
FWD_(I)-1625515	1625515	1625515	YES
FWD_(II)-1626350	1626350	1626350	YES
FWD_(II)-1627192	1627192	1627192	
RVS_(II)-1627573	1627573	1627573	
RVS_(II)-1635849	1635849	1635849	
RVS_(I)-1635871	1635871	1635871	YES
FWD_(II)-1636948	1636948	1636948	
RVS_(I)-1639740	1639740	1639740	
FWD_(II)-1640243	1640243	1640243	
RVS_(I)-1640447	1640444	1640447	
RVS_(II)-1642655	1642655	1642655	
RVS_(II)-1642891	1642891	1642891	
RVS_(III)-1643926	1643926	1643926	YES
FWD_(III)-1644404	1644404	1644404	
RVS_(II)-1644847	1644847	1644847	
RVS_(II)-1644905	1644905	1644905	
RVS_(I)-1645145	1645145	1645145	
FWD_(I)-1645955	1645955	1645955	
FWD_(III)-1647400	1647400	1647405	
FWD_(III)-1647475	1647475	1647480	

FWD_(I)-1653733	1653733	1653733	YES
FWD_(I)-1653749	1653749	1653749	
RVS_(II)-1654770	1654770	1654770	
RVS_(II)-1658507	1658507	1658507	
FWD_(II)-1663154	1663154	1663154	
RVS_(I)-1665295	1665295	1665295	
RVS_(III)-1666629	1666625	1666629	YES
RVS_(II)-1669044	1669044	1669044	
RVS_(I)-1669158	1669158	1669158	
FWD_(II)-1669940	1669940	1669940	
RVS_(II)-1671803	1671803	1671803	
RVS_(II)-1675999	1675999	1675999	
RVS_(I)-1676039	1676039	1676039	
FWD_(I)-1676392	1676392	1676392	
RVS_(II)-1680085	1680085	1680085	
FWD_(III)-1680162	1680162	1680165	YES
RVS_(II)-1686338	1686338	1686338	
RVS_(I)-1686472	1686472	1686472	YES
FWD_(I)-1686569	1686569	1686569	YES
RVS_(II)-1695099	1695099	1695099	
RVS_(II)-1695786	1695786	1695786	
RVS_(I)-1696112	1696112	1696112	
RVS_(II)-1697255	1697255	1697255	YES
FWD_(II)-1698864	1698864	1698864	
RVS_(II)-1702356	1702356	1702356	
FWD_(I)-1702536	1702536	1702538	YES
FWD_(II)-1702788	1702788	1702788	
FWD_(II)-1703230	1703230	1703230	YES
FWD_(II)-1703768	1703768	1703768	
RVS_(II)-1706509	1706509	1706509	
FWD_(II)-1712378	1712378	1712378	
RVS_(I)-1715312	1715312	1715312	YES
RVS_(II)-1716059	1716059	1716059	YES
FWD_(III)-1717775	1717775	1717776	YES
FWD_(I)-1717801	1717801	1717801	YES
RVS_(II)-1718435	1718435	1718435	
FWD_(II)-1718872	1718872	1718872	
RVS_(I)-1718895	1718895	1718895	
RVS_(I)-1718904	1718904	1718904	
FWD_(II)-1718906	1718906	1718906	
RVS_(II)-1722159	1722159	1722159	
RVS_(I)-1723680	1723678	1723680	
RVS_(II)-1723970	1723970	1723970	
FWD_(I)-1725824	1725824	1725824	
RVS_(I)-1732161	1732160	1732161	
FWD_(III)-1733349	1733349	1733349	YES
RVS_(II)-1733377	1733377	1733377	
RVS_(I)-1735608	1735608	1735608	
FWD_(I)-1735712	1735712	1735712	YES
FWD_(I)-1739225	1739225	1739225	YES
FWD_(II)-1741400	1741400	1741400	
RVS_(I)-1741481	1741481	1741481	
RVS_(II)-1744189	1744189	1744189	
FWD_(I)-1744441	1744441	1744441	YES
FWD_(II)-1744697	1744697	1744697	
RVS_(II)-1746012	1746010	1746012	
RVS_(II)-1752668	1752668	1752668	YES
FWD_(II)-1752898	1752898	1752898	
FWD_(III)-1753493	1753493	1753493	

FWD_(I)-1755407	1755407	1755407	YES
RVS_(I)-1762794	1762793	1762794	
FWD_(II)-1767001	1767001	1767001	
FWD_(II)-1767036	1767036	1767036	
FWD_(I)-1768396	1768396	1768396	YES
FWD_(II)-1776332	1776332	1776332	
RVS_(II)-1776476	1776476	1776476	
RVS_(I)-1785186	1785186	1785186	YES
FWD_(II)-1786408	1786408	1786408	YES
RVS_(I)-1790785	1790785	1790785	
RVS_(II)-1793745	1793745	1793745	YES
RVS_(II)-1793944	1793944	1793944	
RVS_(II)-1798055	1798055	1798055	
RVS_(II)-1798844	1798842	1798844	YES
RVS_(II)-1798954	1798954	1798954	
RVS_(II)-1799371	1799371	1799371	YES
RVS_(II)-1799735	1799735	1799735	
RVS_(II)-1799793	1799793	1799793	
RVS_(I)-1800758	1800757	1800758	YES
RVS_(II)-1800831	1800831	1800831	
RVS_(I)-1804195	1804193	1804195	
FWD_(I)-1805794	1805794	1805794	
RVS_(I)-1807288	1807287	1807288	YES
FWD_(II)-1807348	1807348	1807348	YES
RVS_(II)-1807375	1807375	1807375	
FWD_(II)-1808951	1808951	1808951	
RVS_(II)-1810428	1810428	1810428	
RVS_(II)-1815383	1815383	1815383	
RVS_(II)-1819751	1819751	1819751	YES
RVS_(I)-1820310	1820308	1820310	YES
RVS_(I)-1823177	1823177	1823177	
RVS_(II)-1823219	1823219	1823219	
FWD_(III)-1830423	1830423	1830423	YES
RVS_(II)-1830922	1830922	1830922	
FWD_(II)-1837463	1837463	1837465	YES
RVS_(I)-1846055	1846055	1846055	YES
FWD_(II)-1846816	1846816	1846816	
FWD_(II)-1850204	1850204	1850204	
FWD_(II)-1850427	1850427	1850427	
RVS_(II)-1859834	1859832	1859834	
RVS_(I)-1860489	1860489	1860489	
FWD_(I)-1860550	1860550	1860550	YES
FWD_(I)-1860621	1860621	1860621	YES
FWD_(II)-1860642	1860642	1860642	YES
FWD_(II)-1860759	1860759	1860759	YES
FWD_(II)-1862462	1862462	1862462	
RVS_(II)-1863678	1863678	1863678	
RVS_(I)-1864531	1864529	1864531	YES
RVS_(II)-1868361	1868361	1868361	
FWD_(II)-1869288	1869288	1869288	
RVS_(II)-1870977	1870977	1870977	
FWD_(II)-1874901	1874901	1874901	
FWD_(II)-1874969	1874969	1874969	
RVS_(III)-1877323	1877323	1877323	
RVS_(II)-1886089	1886088	1886089	YES
RVS_(II)-1886626	1886626	1886626	
RVS_(II)-1888008	1888008	1888008	
FWD_(I)-1891360	1891360	1891360	
FWD_(II)-1892413	1892413	1892413	

RVS_(II)-1893274	1893274	1893274	
FWD_(I)-1894167	1894167	1894167	
FWD_(I)-1894178	1894178	1894178	
FWD_(I)-1894787	1894787	1894787	
FWD_(II)-1894907	1894907	1894907	
RVS_(II)-1899858	1899858	1899858	
FWD_(I)-1899959	1899959	1899959	YES
FWD_(II)-1903486	1903486	1903486	
RVS_(II)-1904501	1904499	1904501	
RVS_(I)-1905644	1905642	1905644	YES
RVS_(I)-1905664	1905661	1905664	
RVS_(I)-1905820	1905818	1905820	YES
FWD_(II)-1906174	1906174	1906174	
RVS_(II)-1906594	1906592	1906594	
RVS_(I)-1906817	1906817	1906817	YES
RVS_(III)-1908165	1908161	1908165	
RVS_(II)-1913607	1913607	1913607	
RVS_(III)-1914192	1914189	1914192	
FWD_(II)-1918977	1918977	1918977	
FWD_(II)-1920121	1920121	1920121	
FWD_(I)-1921090	1921090	1921090	YES
RVS_(I)-1921231	1921231	1921231	
FWD_(II)-1923110	1923110	1923112	
RVS_(I)-1923332	1923332	1923332	
RVS_(II)-1927716	1927716	1927716	
RVS_(II)-1927759	1927759	1927759	YES
RVS_(I)-1928801	1928798	1928801	
RVS_(II)-1932739	1932739	1932739	YES
FWD_(I)-1934613	1934613	1934613	
FWD_(II)-1935521	1935521	1935521	YES
RVS_(II)-1940051	1940051	1940051	
RVS_(II)-1940107	1940107	1940107	
FWD_(II)-1940581	1940581	1940581	
FWD_(II)-1940614	1940614	1940614	
RVS_(II)-1944033	1944033	1944033	YES
RVS_(I)-1948727	1948727	1948727	YES
FWD_(I)-1950260	1950260	1950260	
FWD_(III)-1952448	1952448	1952452	
RVS_(II)-1956772	1956772	1956772	
RVS_(II)-1960391	1960391	1960391	
RVS_(I)-1964242	1964242	1964242	
RVS_(I)-1970740	1970740	1970740	YES
RVS_(I)-1975327	1975325	1975327	
RVS_(II)-1976420	1976420	1976420	YES
RVS_(II)-1977621	1977621	1977621	
FWD_(II)-1977744	1977744	1977744	
RVS_(II)-1978760	1978758	1978760	
FWD_(II)-1982461	1982461	1982461	
FWD_(I)-1984821	1984821	1984822	YES
FWD_(III)-1986221	1986221	1986221	
FWD_(III)-1986706	1986706	1986706	
RVS_(III)-1987500	1987500	1987500	
RVS_(III)-1987535	1987533	1987535	
RVS_(III)-1989674	1989673	1989674	
RVS_(I)-1990208	1990208	1990208	
FWD_(II)-1992437	1992437	1992437	
RVS_(II)-1992901	1992901	1992901	YES
RVS_(I)-1993428	1993427	1993428	YES
FWD_(III)-1993797	1993797	1993800	

FWD_(I)-1994904	1994904	1994904	
RVS_(I)-1994918	1994918	1994918	
RVS_(I)-1994935	1994935	1994935	
FWD_(I)-1994942	1994942	1994945	
FWD_(I)-1994951	1994951	1994951	
FWD_(II)-1994973	1994973	1994973	
FWD_(II)-1995003	1995003	1995003	
FWD_(II)-1996502	1996502	1996502	
RVS_(I)-1999832	1999831	1999832	YES
RVS_(I)-1999844	1999843	1999844	YES
FWD_(III)-1999988	1999988	1999990	
RVS_(I)-2001700	2001700	2001700	YES
FWD_(I)-2001839	2001839	2001839	
FWD_(II)-2001860	2001860	2001860	
FWD_(II)-2004211	2004211	2004211	
RVS_(III)-2006140	2006137	2006140	
FWD_(I)-2006262	2006262	2006264	
RVS_(I)-2011064	2011064	2011064	
FWD_(I)-2011167	2011167	2011167	
FWD_(I)-2017609	2017609	2017611	
RVS_(I)-2022869	2022869	2022869	
FWD_(II)-2022951	2022951	2022951	
RVS_(I)-2023337	2023335	2023337	YES
FWD_(II)-2023546	2023546	2023546	
FWD_(II)-2026130	2026130	2026130	
RVS_(II)-2026421	2026421	2026421	
RVS_(II)-2027415	2027415	2027415	
RVS_(II)-2031124	2031124	2031124	
RVS_(II)-2033246	2033246	2033246	
FWD_(II)-2033655	2033655	2033655	YES
RVS_(II)-2036848	2036848	2036848	
FWD_(II)-2037773	2037773	2037773	
FWD_(I)-2041439	2041439	2041439	
RVS_(I)-2041590	2041590	2041590	
FWD_(I)-2042562	2042562	2042562	
RVS_(II)-2042793	2042793	2042793	
FWD_(II)-2050035	2050035	2050035	
RVS_(II)-2051383	2051383	2051383	
FWD_(II)-2051590	2051590	2051590	
FWD_(II)-2053054	2053054	2053054	YES
FWD_(III)-2054821	2054821	2054823	
FWD_(II)-2055587	2055587	2055587	
RVS_(I)-2056138	2056138	2056138	
FWD_(I)-2057866	2057866	2057867	
FWD_(I)-2060273	2060273	2060273	
RVS_(II)-2061378	2061378	2061378	
RVS_(II)-2063872	2063872	2063872	
RVS_(II)-2064021	2064021	2064021	
RVS_(III)-2065380	2065375	2065380	
RVS_(II)-2065943	2065937	2065943	
RVS_(II)-2065945	2065943	2065945	
RVS_(I)-2066508	2066507	2066508	
RVS_(III)-2068232	2068232	2068232	
RVS_(I)-2072769	2072765	2072769	
RVS_(I)-2072774	2072769	2072774	
FWD_(II)-2076002	2076002	2076002	
FWD_(II)-2076053	2076053	2076053	
RVS_(I)-2077494	2077492	2077494	
RVS_(I)-2079311	2079311	2079311	

RVS_(I)-2079351	2079351	2079351	YES
FWD_(II)-2083636	2083636	2083636	
RVS_(II)-2084952	2084952	2084952	
FWD_(II)-2085083	2085083	2085083	
RVS_(I)-2085287	2085284	2085287	
RVS_(II)-2087177	2087177	2087177	
RVS_(II)-2087188	2087188	2087188	
RVS_(III)-2087758	2087756	2087758	YES
FWD_(II)-2087780	2087780	2087780	
FWD_(I)-2087989	2087989	2087989	YES
FWD_(II)-2094649	2094649	2094649	
RVS_(II)-2096227	2096224	2096227	
RVS_(III)-2096370	2096366	2096370	
RVS_(I)-2099349	2099349	2099349	YES
RVS_(III)-2100974	2100974	2100974	
FWD_(II)-2107454	2107454	2107454	
RVS_(II)-2107875	2107875	2107875	
RVS_(I)-2111259	2111256	2111259	
RVS_(II)-2112123	2112123	2112123	
RVS_(I)-2112376	2112376	2112376	YES
RVS_(II)-2112441	2112441	2112441	
FWD_(III)-2116487	2116487	2116490	
RVS_(III)-2116680	2116677	2116680	
FWD_(II)-2124816	2124816	2124816	
FWD_(II)-2135887	2135887	2135887	YES
RVS_(II)-2137643	2137643	2137643	
RVS_(II)-2137695	2137695	2137695	
RVS_(II)-2139529	2139529	2139529	
RVS_(I)-2140304	2140304	2140304	
FWD_(I)-2151335	2151335	2151335	YES
FWD_(I)-2151670	2151670	2151670	YES
RVS_(II)-2151852	2151852	2151852	
FWD_(II)-2152003	2152003	2152003	
FWD_(II)-2163617	2163617	2163619	
FWD_(I)-2165138	2165138	2165138	YES
RVS_(I)-2167783	2167783	2167783	
RVS_(II)-2169717	2169717	2169717	
RVS_(I)-2175257	2175256	2175257	YES
RVS_(II)-2181397	2181397	2181397	
FWD_(II)-2181639	2181639	2181639	
RVS_(II)-2183474	2183474	2183474	YES
FWD_(II)-2184884	2184884	2184884	
RVS_(I)-2192223	2192222	2192223	
FWD_(I)-2192291	2192291	2192291	YES
FWD_(I)-2202424	2202424	2202424	
FWD_(II)-2212942	2212942	2212942	
FWD_(II)-2213659	2213659	2213659	
RVS_(II)-2221884	2221884	2221884	
RVS_(II)-2223088	2223087	2223088	
RVS_(II)-2223201	2223201	2223201	
FWD_(II)-2227180	2227180	2227180	
FWD_(II)-2228625	2228625	2228625	
FWD_(III)-2229839	2229839	2229842	YES
RVS_(II)-2231320	2231320	2231320	
FWD_(III)-2231993	2231993	2231998	
RVS_(III)-2238590	2238589	2238590	
RVS_(II)-2246583	2246583	2246583	YES
FWD_(II)-2253312	2253312	2253312	YES
RVS_(II)-2255340	2255340	2255340	

RVS_(II)-2257413	2257413	2257413	
FWD_(II)-2257511	2257511	2257511	
FWD_(II)-2257579	2257579	2257579	
FWD_(II)-2262845	2262845	2262845	
RVS_(II)-2263354	2263352	2263354	
FWD_(III)-2263431	2263431	2263439	
RVS_(II)-2265816	2265816	2265816	
FWD_(II)-2265818	2265818	2265818	
FWD_(I)-2267832	2267832	2267832	
FWD_(I)-2267942	2267942	2267942	YES
FWD_(II)-2268505	2268505	2268505	
FWD_(II)-2268736	2268736	2268736	
FWD_(II)-2270314	2270314	2270314	
FWD_(II)-2270335	2270335	2270335	
RVS_(II)-2275804	2275799	2275804	
RVS_(I)-2276489	2276487	2276489	
FWD_(II)-2276509	2276509	2276511	
RVS_(I)-2278567	2278567	2278567	
FWD_(I)-2280463	2280463	2280463	
FWD_(I)-2284227	2284227	2284227	
RVS_(II)-2286893	2286893	2286893	
RVS_(III)-2288136	2288133	2288136	
FWD_(III)-2288470	2288470	2288473	
RVS_(III)-2289327	2289325	2289327	
RVS_(II)-2295934	2295934	2295934	
RVS_(I)-2301599	2301597	2301599	YES
FWD_(I)-2301883	2301883	2301883	
RVS_(II)-2303080	2303080	2303080	
RVS_(I)-2303100	2303100	2303100	
RVS_(I)-2310851	2310851	2310851	YES
FWD_(I)-2311106	2311106	2311106	YES
RVS_(II)-2311283	2311283	2311283	
FWD_(III)-2313720	2313720	2313721	YES
FWD_(II)-2313997	2313997	2313997	YES
FWD_(II)-2316346	2316346	2316346	
FWD_(II)-2318037	2318037	2318037	
RVS_(II)-2318152	2318152	2318152	
RVS_(II)-2318490	2318490	2318490	
FWD_(II)-2331924	2331924	2331924	
RVS_(I)-2337482	2337480	2337482	YES
FWD_(III)-2338330	2338330	2338334	
FWD_(III)-2338351	2338351	2338355	
FWD_(II)-2345086	2345086	2345086	
FWD_(III)-2345178	2345178	2345180	
RVS_(III)-2345352	2345348	2345352	
RVS_(I)-2350474	2350473	2350474	
FWD_(I)-2350607	2350607	2350607	
FWD_(I)-2350643	2350643	2350645	
RVS_(II)-2355400	2355400	2355400	
RVS_(II)-2356086	2356086	2356086	
FWD_(II)-2359440	2359440	2359440	
RVS_(II)-2361678	2361678	2361678	YES
RVS_(III)-2362340	2362337	2362340	
RVS_(II)-2371558	2371558	2371558	
RVS_(I)-2371595	2371594	2371595	
RVS_(III)-2375025	2375023	2375025	
RVS_(II)-2377304	2377304	2377304	
RVS_(III)-2379076	2379076	2379076	
RVS_(II)-2379586	2379586	2379586	

FWD_(II)-2383426	2383426	2383426	
FWD_(II)-2384931	2384931	2384931	
RVS_(III)-2403414	2403414	2403414	
RVS_(I)-2403850	2403850	2403850	
RVS_(II)-2404822	2404822	2404822	
RVS_(II)-2404843	2404842	2404843	
RVS_(II)-2405035	2405035	2405035	
RVS_(II)-2406875	2406875	2406875	
FWD_(II)-2408853	2408853	2408853	
RVS_(II)-2409402	2409402	2409402	
RVS_(II)-2410668	2410668	2410668	
FWD_(I)-2411269	2411269	2411269	
RVS_(II)-2411313	2411313	2411313	
FWD_(I)-2411449	2411449	2411449	
FWD_(II)-2412509	2412509	2412509	
RVS_(II)-2417247	2417247	2417247	
RVS_(I)-2417833	2417833	2417833	
FWD_(I)-2419321	2419321	2419323	
RVS_(II)-2420912	2420912	2420912	
FWD_(II)-2423922	2423922	2423922	
RVS_(II)-2426343	2426343	2426343	
RVS_(II)-2426673	2426673	2426673	
RVS_(II)-2427034	2427034	2427034	
RVS_(II)-2428822	2428822	2428822	YES
RVS_(I)-2429154	2429152	2429154	
RVS_(I)-2432037	2432037	2432037	YES
RVS_(II)-2432215	2432215	2432215	
RVS_(I)-2439664	2439664	2439664	
FWD_(III)-2441802	2441802	2441802	
RVS_(III)-2441876	2441870	2441876	
RVS_(II)-2445653	2445651	2445653	
RVS_(I)-2446496	2446495	2446496	
FWD_(III)-2446600	2446600	2446605	
RVS_(II)-2447460	2447460	2447460	
RVS_(II)-2447739	2447739	2447739	
RVS_(II)-2454046	2454046	2454046	
FWD_(II)-2454248	2454248	2454248	
RVS_(II)-2454939	2454939	2454939	
RVS_(II)-2455022	2455022	2455022	YES
RVS_(II)-2455398	2455398	2455398	
RVS_(II)-2455535	2455535	2455535	
RVS_(I)-2459002	2459002	2459002	YES
FWD_(I)-2459272	2459272	2459272	
RVS_(II)-2461373	2461373	2461373	
RVS_(II)-2463107	2463107	2463107	
RVS_(III)-2463238	2463238	2463238	
FWD_(II)-2463467	2463467	2463467	
FWD_(III)-2464326	2464326	2464326	
RVS_(II)-2465281	2465280	2465281	
RVS_(II)-2466650	2466650	2466650	
RVS_(III)-2468688	2468688	2468688	
RVS_(II)-2477125	2477125	2477125	
FWD_(I)-2481652	2481652	2481654	
FWD_(I)-2481663	2481663	2481663	YES
RVS_(II)-2485429	2485429	2485429	
RVS_(II)-2493304	2493304	2493304	
RVS_(II)-2493357	2493357	2493357	
FWD_(II)-2493515	2493515	2493515	
RVS_(II)-2496379	2496379	2496379	

RVS_(II)-2496644	2496644	2496644	
FWD_(II)-2499054	2499054	2499054	
RVS_(II)-2499146	2499146	2499146	
RVS_(II)-2499471	2499471	2499471	
RVS_(II)-2500037	2500037	2500037	
RVS_(I)-2507486	2507486	2507486	YES
RVS_(I)-2508092	2508089	2508092	
FWD_(II)-2509240	2509240	2509240	
FWD_(II)-2510716	2510716	2510716	
FWD_(III)-2511031	2511031	2511034	YES
RVS_(II)-2513869	2513869	2513869	
RVS_(II)-2515890	2515890	2515890	
FWD_(III)-2516059	2516059	2516062	
FWD_(III)-2516173	2516173	2516177	
RVS_(II)-2516280	2516279	2516280	
FWD_(I)-2516429	2516429	2516429	
FWD_(I)-2516443	2516443	2516444	
FWD_(II)-2517138	2517138	2517141	
RVS_(I)-2518753	2518753	2518753	YES
FWD_(I)-2518944	2518944	2518946	
FWD_(III)-2519065	2519065	2519068	
RVS_(II)-2520528	2520528	2520528	YES
RVS_(II)-2521828	2521828	2521828	
FWD_(II)-2523897	2523897	2523897	
FWD_(II)-2524948	2524948	2524948	
RVS_(I)-2529303	2529301	2529303	
RVS_(II)-2529575	2529575	2529575	
RVS_(II)-2529942	2529942	2529942	
RVS_(II)-2530381	2530381	2530381	
RVS_(II)-2530389	2530389	2530389	
FWD_(II)-2531333	2531333	2531333	
RVS_(II)-2531504	2531504	2531504	
FWD_(I)-2531523	2531523	2531523	YES
FWD_(II)-2531628	2531628	2531630	YES
FWD_(II)-2531752	2531752	2531752	
FWD_(II)-2531972	2531972	2531972	
FWD_(II)-2532811	2532811	2532811	
FWD_(II)-2533504	2533504	2533504	YES
FWD_(I)-2533632	2533632	2533632	YES
FWD_(II)-2535779	2535779	2535779	
RVS_(II)-2536402	2536402	2536402	
FWD_(III)-2536552	2536552	2536557	
FWD_(III)-2537625	2537625	2537629	
RVS_(II)-2538213	2538213	2538213	
RVS_(I)-2542688	2542686	2542688	YES
RVS_(II)-2548616	2548613	2548616	
RVS_(II)-2548868	2548868	2548868	
RVS_(II)-2555317	2555317	2555317	
FWD_(I)-2555334	2555334	2555334	
RVS_(II)-2557588	2557588	2557588	
RVS_(I)-2558395	2558395	2558395	
FWD_(II)-2570140	2570140	2570140	
RVS_(III)-2576554	2576554	2576554	
FWD_(II)-2581486	2581486	2581486	
RVS_(II)-2581570	2581570	2581570	
RVS_(II)-2588877	2588877	2588877	
RVS_(II)-2588908	2588907	2588908	
RVS_(I)-2588995	2588991	2588995	
RVS_(II)-2591161	2591161	2591161	

RVS_(II)-2594786	2594786	2594786	
RVS_(I)-2594807	2594807	2594807	
RVS_(II)-2595462	2595462	2595462	
RVS_(II)-2595804	2595804	2595804	YES
RVS_(I)-2597807	2597807	2597807	YES
FWD_(III)-2597898	2597898	2597898	YES
RVS_(II)-2601054	2601054	2601054	
RVS_(II)-2613939	2613938	2613939	
FWD_(II)-2614009	2614009	2614009	
FWD_(II)-2614981	2614981	2614981	
FWD_(II)-2615877	2615877	2615878	
FWD_(II)-2618194	2618194	2618194	
RVS_(III)-2618934	2618930	2618934	YES
FWD_(II)-2619031	2619031	2619031	
FWD_(II)-2619052	2619052	2619052	
FWD_(II)-2620222	2620222	2620223	
FWD_(II)-2620265	2620265	2620265	
FWD_(II)-2620999	2620999	2620999	
FWD_(II)-2626046	2626046	2626046	
RVS_(II)-2627055	2627054	2627055	YES
FWD_(I)-2627274	2627274	2627274	
RVS_(II)-2630738	2630738	2630738	
FWD_(II)-2632730	2632730	2632730	
FWD_(II)-2633987	2633987	2633987	
RVS_(II)-2635501	2635501	2635501	
RVS_(II)-2635667	2635666	2635667	
RVS_(II)-2638664	2638664	2638664	YES
RVS_(II)-2640004	2640004	2640004	
RVS_(II)-2640948	2640948	2640948	
RVS_(I)-2641027	2641025	2641027	
RVS_(III)-2642950	2642945	2642950	
RVS_(II)-2650348	2650348	2650348	YES
RVS_(II)-2650474	2650474	2650474	
FWD_(I)-2650490	2650490	2650491	
RVS_(I)-2651478	2651475	2651478	
RVS_(I)-2651800	2651799	2651800	
FWD_(III)-2651879	2651879	2651879	
FWD_(I)-2654398	2654398	2654399	
RVS_(II)-2658147	2658147	2658147	
FWD_(II)-2660212	2660212	2660212	
FWD_(III)-2660329	2660329	2660332	
RVS_(I)-2661372	2661370	2661372	
FWD_(I)-2663423	2663423	2663423	YES
FWD_(I)-2663433	2663433	2663433	
RVS_(II)-2665056	2665054	2665056	
FWD_(II)-2667517	2667517	2667517	
RVS_(II)-2670679	2670679	2670679	
FWD_(II)-2671343	2671343	2671343	
RVS_(III)-2671827	2671827	2671827	
FWD_(II)-2680800	2680800	2680800	
RVS_(I)-2683594	2683594	2683594	YES
RVS_(I)-2685464	2685464	2685464	YES
RVS_(II)-2685526	2685526	2685526	YES
RVS_(II)-2688077	2688077	2688077	
RVS_(I)-2689363	2689363	2689363	YES
FWD_(II)-2689517	2689517	2689517	
RVS_(III)-2696615	2696615	2696615	
RVS_(I)-2698400	2698399	2698400	
FWD_(I)-2698542	2698542	2698542	YES

RVS_(III)-2699837	2699835	2699837	YES
RVS_(II)-2702031	2702031	2702031	
RVS_(II)-2702248	2702247	2702248	YES
FWD_(II)-2705253	2705253	2705253	
RVS_(II)-2707427	2707427	2707427	
RVS_(II)-2707655	2707655	2707655	YES
RVS_(II)-2708115	2708115	2708115	YES
RVS_(II)-2708178	2708178	2708178	
RVS_(I)-2708252	2708252	2708252	
RVS_(III)-2712411	2712405	2712411	
RVS_(II)-2712548	2712548	2712548	
RVS_(II)-2713343	2713343	2713343	
RVS_(I)-2714544	2714544	2714544	YES
FWD_(II)-2714752	2714752	2714752	YES
RVS_(II)-2716080	2716080	2716080	
RVS_(I)-2716577	2716577	2716577	
FWD_(I)-2717953	2717953	2717953	
FWD_(II)-2720309	2720309	2720309	
FWD_(II)-2720525	2720525	2720525	
RVS_(III)-2723841	2723836	2723841	
FWD_(II)-2724015	2724015	2724015	
RVS_(III)-2724212	2724210	2724212	
RVS_(III)-2727467	2727465	2727467	
RVS_(II)-2727937	2727933	2727937	
RVS_(II)-2729357	2729357	2729357	
RVS_(I)-2729472	2729471	2729472	
RVS_(II)-2729661	2729661	2729661	
RVS_(I)-2732316	2732314	2732316	YES
FWD_(I)-2734094	2734094	2734094	
FWD_(I)-2734107	2734107	2734107	
RVS_(II)-2734295	2734295	2734295	
FWD_(I)-2735146	2735146	2735151	
FWD_(I)-2735602	2735602	2735602	YES
FWD_(I)-2739846	2739846	2739846	
RVS_(I)-2744277	2744277	2744277	YES
RVS_(III)-2745878	2745877	2745878	YES
RVS_(I)-2748769	2748769	2748769	YES
FWD_(II)-2748787	2748787	2748787	
RVS_(II)-2750293	2750293	2750293	
FWD_(I)-2751577	2751577	2751577	
RVS_(I)-2752771	2752771	2752771	
FWD_(I)-2752854	2752854	2752854	
FWD_(I)-2753607	2753607	2753608	
FWD_(I)-2753608	2753608	2753615	
FWD_(II)-2753879	2753879	2753879	
FWD_(II)-2754155	2754155	2754155	
FWD_(II)-2755389	2755389	2755390	
FWD_(II)-2759160	2759160	2759160	
FWD_(II)-2759265	2759265	2759265	
RVS_(II)-2759742	2759742	2759742	
RVS_(II)-2763344	2763344	2763344	
RVS_(II)-2763505	2763505	2763505	
FWD_(I)-2763916	2763916	2763916	YES
FWD_(II)-2764155	2764155	2764156	
FWD_(II)-2764703	2764703	2764703	
FWD_(II)-2764882	2764882	2764882	YES
FWD_(II)-2771308	2771308	2771308	
RVS_(I)-2773541	2773539	2773541	
RVS_(II)-2774382	2774382	2774382	

FWD_(II)-2780903	2780903	2780903	
RVS_(II)-2781594	2781594	2781594	
RVS_(III)-2783859	2783859	2783859	
RVS_(II)-2794437	2794437	2794437	
RVS_(I)-2794836	2794836	2794836	
RVS_(II)-2795099	2795099	2795099	YES
RVS_(II)-2795108	2795108	2795108	YES
RVS_(II)-2796560	2796560	2796560	YES
FWD_(I)-2797108	2797108	2797108	
FWD_(I)-2798143	2798143	2798143	YES
FWD_(II)-2798443	2798443	2798443	
RVS_(II)-2800389	2800389	2800389	
RVS_(II)-2805156	2805156	2805156	
FWD_(II)-2805709	2805709	2805709	
FWD_(I)-2807591	2807591	2807591	
FWD_(I)-2812824	2812824	2812824	YES
RVS_(II)-2813089	2813089	2813089	YES
RVS_(II)-2814776	2814776	2814776	
RVS_(III)-2815551	2815549	2815551	
RVS_(II)-2816165	2816165	2816165	
RVS_(II)-2816304	2816303	2816304	
RVS_(I)-2816724	2816721	2816724	
RVS_(II)-2817212	2817210	2817212	
RVS_(II)-2817221	2817221	2817221	
RVS_(II)-2817296	2817296	2817296	YES
RVS_(II)-2817710	2817709	2817710	
RVS_(II)-2820113	2820113	2820113	YES
RVS_(III)-2821843	2821839	2821843	YES
RVS_(I)-2822396	2822396	2822396	
FWD_(I)-2823813	2823813	2823813	YES
FWD_(I)-2826983	2826983	2826984	
RVS_(II)-2830334	2830333	2830334	YES
FWD_(II)-2830612	2830612	2830612	
RVS_(II)-2832208	2832208	2832208	
RVS_(II)-2835643	2835643	2835643	
FWD_(II)-2840105	2840105	2840105	
FWD_(II)-2840407	2840407	2840407	
RVS_(II)-2841125	2841125	2841125	
FWD_(I)-2848860	2848860	2848861	YES
RVS_(II)-2850764	2850764	2850764	
FWD_(II)-2850896	2850896	2850896	
RVS_(II)-2852282	2852282	2852282	
RVS_(II)-2857758	2857758	2857758	
RVS_(II)-2862750	2862750	2862750	
RVS_(I)-2866140	2866140	2866140	YES
RVS_(II)-2867577	2867577	2867577	
RVS_(I)-2868709	2868709	2868709	YES
RVS_(II)-2870923	2870923	2870923	
RVS_(II)-2870980	2870980	2870980	
RVS_(II)-2870998	2870998	2870998	
RVS_(II)-2871411	2871411	2871411	
RVS_(III)-2876486	2876483	2876486	
RVS_(II)-2878910	2878910	2878910	
FWD_(II)-2885375	2885375	2885375	
FWD_(II)-2890658	2890658	2890658	
RVS_(I)-2898388	2898384	2898388	
FWD_(I)-2898527	2898527	2898528	
FWD_(II)-2902008	2902008	2902008	
RVS_(I)-2902484	2902484	2902484	

RVS_(I)-2902602	2902601	2902602	
RVS_(I)-2903465	2903465	2903465	
RVS_(II)-2906461	2906461	2906461	
RVS_(II)-2906902	2906902	2906902	
RVS_(III)-2907741	2907736	2907741	YES
FWD_(II)-2908687	2908687	2908687	
RVS_(II)-2909391	2909391	2909391	YES
RVS_(II)-2911388	2911388	2911388	
RVS_(I)-2911852	2911852	2911852	YES
RVS_(II)-2912300	2912300	2912300	YES
FWD_(II)-2912782	2912782	2912782	
RVS_(II)-2915565	2915565	2915565	
RVS_(I)-2921036	2921036	2921036	
RVS_(I)-2922547	2922547	2922547	YES
RVS_(II)-2923336	2923336	2923336	
FWD_(II)-2923341	2923341	2923341	YES
FWD_(I)-2924294	2924294	2924294	
RVS_(II)-2924459	2924459	2924459	
FWD_(II)-2925959	2925959	2925959	
FWD_(III)-2926124	2926124	2926125	
RVS_(II)-2927598	2927598	2927598	
FWD_(II)-2929056	2929056	2929056	
FWD_(II)-2930652	2930652	2930652	
FWD_(II)-2932838	2932838	2932838	
FWD_(II)-2936740	2936740	2936740	
FWD_(II)-2937343	2937343	2937343	
RVS_(II)-2938290	2938290	2938290	
FWD_(I)-2940718	2940718	2940720	YES
FWD_(II)-2941341	2941341	2941341	
RVS_(I)-2944086	2944086	2944086	
RVS_(II)-2944296	2944296	2944296	
RVS_(II)-2945231	2945231	2945231	
FWD_(I)-2945404	2945404	2945404	YES
FWD_(II)-2948561	2948561	2948561	
RVS_(II)-2954223	2954222	2954223	
RVS_(I)-2967028	2967028	2967028	
FWD_(II)-2968416	2968416	2968416	
FWD_(I)-2969260	2969260	2969260	
FWD_(I)-2969274	2969274	2969274	
FWD_(II)-2969587	2969587	2969588	
RVS_(II)-2974073	2974073	2974073	
RVS_(I)-2974213	2974210	2974213	YES
RVS_(II)-2974388	2974388	2974388	
RVS_(I)-2974410	2974406	2974410	YES
FWD_(II)-2974591	2974591	2974591	YES
RVS_(II)-2975755	2975755	2975755	
RVS_(II)-2983636	2983636	2983636	
FWD_(II)-2986481	2986481	2986481	
RVS_(III)-2995654	2995650	2995654	
RVS_(II)-2996158	2996158	2996158	
RVS_(I)-2997092	2997088	2997092	
RVS_(I)-2998115	2998115	2998115	
RVS_(II)-3002696	3002696	3002696	
FWD_(II)-3003507	3003507	3003507	
RVS_(I)-3003870	3003865	3003870	YES
RVS_(II)-3005787	3005787	3005787	
FWD_(II)-3007175	3007175	3007176	
FWD_(II)-3013080	3013080	3013080	
RVS_(II)-3013245	3013241	3013245	

RVS_(II)-3013395	3013395	3013395	
RVS_(II)-3013813	3013813	3013813	
RVS_(II)-3014410	3014410	3014410	
RVS_(II)-3023600	3023600	3023600	
RVS_(II)-3037789	3037789	3037789	
RVS_(II)-3037801	3037801	3037801	
FWD_(I)-3037844	3037844	3037844	YES
RVS_(I)-3039122	3039122	3039122	YES
FWD_(III)-3039301	3039301	3039304	YES
RVS_(II)-3041207	3041207	3041207	
FWD_(II)-3041508	3041508	3041508	
FWD_(I)-3041661	3041661	3041661	
RVS_(III)-3041678	3041678	3041678	
FWD_(II)-3043096	3043096	3043096	
RVS_(II)-3045109	3045109	3045109	
RVS_(I)-3048800	3048798	3048800	YES
FWD_(II)-3053471	3053471	3053471	
RVS_(III)-3053497	3053495	3053497	
FWD_(II)-3053576	3053576	3053576	
FWD_(I)-3053781	3053781	3053781	YES
FWD_(I)-3053996	3053996	3053996	YES
FWD_(I)-3054873	3054873	3054873	YES
RVS_(I)-3055032	3055032	3055032	
RVS_(II)-3056571	3056571	3056571	YES
RVS_(III)-3057375	3057375	3057375	YES
RVS_(II)-3061141	3061141	3061141	
FWD_(II)-3065037	3065037	3065037	
RVS_(II)-3065235	3065235	3065235	
RVS_(II)-3065803	3065803	3065803	
RVS_(I)-3066150	3066150	3066150	YES
RVS_(II)-3066859	3066859	3066859	YES
RVS_(II)-3067239	3067239	3067239	
RVS_(I)-3067894	3067894	3067894	YES
RVS_(I)-3070882	3070880	3070882	YES
RVS_(I)-3071846	3071846	3071846	YES
RVS_(I)-3073253	3073248	3073253	
RVS_(I)-3079739	3079738	3079739	
FWD_(I)-3079869	3079869	3079869	
RVS_(II)-3081969	3081969	3081969	YES
RVS_(II)-3083954	3083954	3083954	
RVS_(I)-3084432	3084432	3084432	
FWD_(III)-3084589	3084589	3084589	
FWD_(II)-3085268	3085268	3085268	
FWD_(II)-3086737	3086737	3086737	
FWD_(I)-3089519	3089519	3089519	
FWD_(II)-3093898	3093898	3093898	
RVS_(I)-3098775	3098774	3098775	YES
RVS_(II)-3098805	3098805	3098805	YES
RVS_(II)-3099462	3099462	3099462	
RVS_(II)-3099774	3099774	3099774	YES
RVS_(II)-3100222	3100222	3100222	
RVS_(II)-3101998	3101998	3101998	
FWD_(I)-3102014	3102014	3102014	YES
FWD_(II)-3102580	3102580	3102580	
FWD_(I)-3103673	3103673	3103673	YES
FWD_(I)-3108325	3108325	3108325	
FWD_(I)-3108385	3108385	3108385	
FWD_(II)-3114756	3114756	3114756	
FWD_(II)-3115242	3115242	3115242	

RVS_(II)-3117363	3117363	3117363	
RVS_(II)-3117659	3117659	3117659	
FWD_(II)-3119165	3119165	3119165	
FWD_(I)-3128173	3128173	3128173	
FWD_(II)-3128209	3128209	3128209	
RVS_(II)-3136633	3136633	3136633	YES
RVS_(III)-3137731	3137725	3137731	
RVS_(II)-3137979	3137979	3137979	
RVS_(II)-3141026	3141026	3141026	
RVS_(II)-3144311	3144311	3144311	
RVS_(I)-3144386	3144386	3144386	YES
RVS_(I)-3144431	3144428	3144431	
FWD_(I)-3145884	3145884	3145884	
RVS_(I)-3150088	3150088	3150088	
FWD_(I)-3151551	3151551	3151551	YES
RVS_(II)-3153213	3153213	3153213	
FWD_(III)-3153325	3153325	3153325	YES
FWD_(II)-3154594	3154594	3154594	YES
RVS_(II)-3156212	3156212	3156212	
RVS_(I)-3160940	3160940	3160940	
RVS_(II)-3161588	3161586	3161588	
RVS_(II)-3163011	3163011	3163011	
FWD_(II)-3164061	3164061	3164061	
RVS_(II)-3166421	3166421	3166421	
RVS_(I)-3166585	3166585	3166585	YES
RVS_(II)-3167438	3167438	3167438	
FWD_(II)-3168803	3168803	3168803	
FWD_(I)-3171142	3171142	3171142	
RVS_(II)-3173668	3173668	3173668	
RVS_(I)-3174988	3174987	3174988	
FWD_(II)-3174999	3174999	3174999	
RVS_(II)-3175051	3175051	3175051	
RVS_(II)-3175129	3175129	3175129	
FWD_(III)-3175888	3175888	3175888	
RVS_(II)-3176377	3176377	3176377	
FWD_(I)-3177739	3177739	3177739	
RVS_(II)-3179062	3179062	3179062	
FWD_(II)-3180446	3180446	3180446	
FWD_(II)-3180474	3180474	3180474	
FWD_(II)-3181294	3181294	3181294	
RVS_(I)-3181648	3181644	3181648	
RVS_(II)-3182286	3182286	3182286	
RVS_(I)-3182741	3182741	3182741	YES
FWD_(III)-3182835	3182835	3182835	
RVS_(II)-3184025	3184025	3184025	
FWD_(III)-3184181	3184181	3184181	
RVS_(II)-3189142	3189142	3189142	
RVS_(I)-3190012	3190012	3190012	
FWD_(II)-3192723	3192723	3192723	
RVS_(I)-3192890	3192888	3192890	YES
FWD_(II)-3193100	3193100	3193100	
RVS_(I)-3193265	3193261	3193265	YES
RVS_(II)-3199055	3199055	3199055	
FWD_(II)-3199962	3199962	3199962	
RVS_(III)-3201282	3201280	3201282	
RVS_(II)-3202206	3202206	3202206	
FWD_(II)-3202677	3202677	3202677	
RVS_(II)-3203460	3203460	3203460	
RVS_(I)-3204302	3204302	3204302	

RVS_(II)-3208589	3208589	3208589	
FWD_(I)-3208669	3208669	3208669	YES
FWD_(I)-3208738	3208738	3208740	YES
FWD_(I)-3213618	3213618	3213618	
FWD_(I)-3213626	3213626	3213626	
FWD_(III)-3214665	3214665	3214667	
FWD_(II)-3219459	3219459	3219459	YES
RVS_(II)-3232503	3232503	3232503	
RVS_(I)-3232783	3232783	3232783	
FWD_(II)-3233926	3233926	3233926	
FWD_(III)-3235302	3235302	3235306	YES
FWD_(II)-3235602	3235602	3235602	
FWD_(II)-3236322	3236322	3236322	
FWD_(II)-3236396	3236396	3236396	
FWD_(II)-3237528	3237528	3237528	
FWD_(II)-3237885	3237885	3237885	YES
FWD_(II)-3243026	3243026	3243026	YES
FWD_(III)-3244645	3244645	3244647	
RVS_(II)-3245500	3245500	3245500	
FWD_(II)-3245625	3245625	3245627	
FWD_(I)-3250285	3250285	3250285	
RVS_(II)-3252265	3252265	3252265	
FWD_(III)-3253035	3253035	3253040	
RVS_(II)-3259471	3259471	3259471	
FWD_(II)-3260171	3260171	3260171	
RVS_(I)-3265116	3265116	3265116	YES
RVS_(I)-3268616	3268615	3268616	YES
FWD_(I)-3273229	3273229	3273229	
RVS_(II)-3286103	3286103	3286103	
RVS_(II)-3286130	3286130	3286130	
FWD_(II)-3291391	3291391	3291391	
FWD_(II)-3293731	3293731	3293731	
FWD_(II)-3297963	3297963	3297963	
RVS_(I)-3299325	3299321	3299325	YES
RVS_(I)-3299340	3299340	3299340	
FWD_(II)-3299463	3299463	3299463	
RVS_(II)-3306721	3306717	3306721	
RVS_(II)-3309347	3309347	3309347	YES
RVS_(I)-3309809	3309807	3309809	YES
RVS_(II)-3315237	3315237	3315237	
RVS_(II)-3316153	3316153	3316153	
RVS_(I)-3316199	3316198	3316199	YES
RVS_(I)-3316320	3316320	3316320	YES
RVS_(II)-3316398	3316398	3316398	YES
RVS_(II)-3319601	3319601	3319601	
RVS_(III)-3320181	3320180	3320181	
RVS_(I)-3320614	3320610	3320614	YES
RVS_(I)-3320658	3320658	3320658	
RVS_(II)-3322348	3322348	3322348	
RVS_(II)-3323414	3323414	3323414	
FWD_(II)-3324772	3324772	3324772	
RVS_(II)-3325075	3325075	3325075	
FWD_(III)-3325303	3325303	3325305	
RVS_(II)-3325538	3325538	3325538	
RVS_(II)-3325565	3325564	3325565	
RVS_(II)-3325706	3325706	3325706	
RVS_(I)-3325754	3325754	3325754	YES
FWD_(III)-3325780	3325780	3325783	YES
RVS_(II)-3326110	3326109	3326110	

RVS_(II)-3326163	3326163	3326163	
RVS_(II)-3326221	3326221	3326221	
RVS_(I)-3326885	3326884	3326885	YES
RVS_(II)-3330056	3330056	3330056	
FWD_(II)-3331534	3331534	3331534	
RVS_(I)-3331540	3331538	3331540	
FWD_(III)-3331658	3331658	3331658	
RVS_(II)-3335006	3335006	3335006	
RVS_(II)-3335959	3335959	3335959	
RVS_(II)-3338144	3338144	3338144	
FWD_(II)-3338233	3338233	3338233	YES
RVS_(II)-3338266	3338265	3338266	
FWD_(II)-3339740	3339740	3339740	
FWD_(II)-3340032	3340032	3340032	
FWD_(II)-3340249	3340249	3340249	
FWD_(II)-3340659	3340659	3340659	
FWD_(II)-3341810	3341810	3341810	
RVS_(II)-3342629	3342629	3342629	
FWD_(II)-3342703	3342703	3342704	
RVS_(II)-3344590	3344590	3344590	
RVS_(II)-3346251	3346250	3346251	
FWD_(I)-3346448	3346448	3346448	YES
FWD_(I)-3348599	3348599	3348599	YES
FWD_(III)-3348663	3348663	3348666	
FWD_(II)-3352084	3352084	3352084	
FWD_(II)-3352533	3352533	3352533	YES
FWD_(II)-3352766	3352766	3352766	
FWD_(II)-3358626	3358626	3358626	
FWD_(II)-3359567	3359567	3359567	
FWD_(II)-3361305	3361305	3361305	
RVS_(II)-3365260	3365260	3365260	
RVS_(II)-3365557	3365556	3365557	
FWD_(II)-3365663	3365663	3365663	
FWD_(II)-3366897	3366897	3366897	
RVS_(II)-3370637	3370637	3370637	
RVS_(II)-3371567	3371567	3371567	
RVS_(III)-3371644	3371644	3371644	YES
RVS_(I)-3372541	3372541	3372541	
RVS_(I)-3375589	3375588	3375589	
FWD_(II)-3375624	3375624	3375624	
RVS_(I)-3376832	3376829	3376832	
FWD_(I)-3378145	3378145	3378145	
RVS_(II)-3378679	3378679	3378679	
RVS_(II)-3381398	3381398	3381398	
RVS_(II)-3382337	3382337	3382337	YES
RVS_(I)-3382495	3382495	3382495	YES
FWD_(II)-3382624	3382624	3382624	YES
FWD_(I)-3382699	3382699	3382699	YES
FWD_(II)-3386965	3386965	3386965	
FWD_(II)-3387502	3387502	3387502	
RVS_(II)-3387836	3387836	3387836	
FWD_(III)-3390096	3390096	3390098	
RVS_(I)-3390444	3390444	3390444	
RVS_(II)-3390953	3390953	3390953	
FWD_(II)-3391554	3391554	3391554	
RVS_(II)-3394777	3394777	3394777	
RVS_(II)-3396538	3396538	3396538	
RVS_(II)-3399377	3399377	3399377	YES
FWD_(II)-3402868	3402868	3402868	

FWD_(I)-3403161	3403161	3403161	YES
FWD_(II)-3403194	3403194	3403194	
FWD_(II)-3405256	3405256	3405256	
FWD_(II)-3408270	3408270	3408270	YES
FWD_(II)-3416352	3416352	3416352	
FWD_(II)-3416940	3416940	3416940	
FWD_(II)-3421119	3421119	3421119	
RVS_(III)-3421814	3421809	3421814	
RVS_(II)-3422700	3422700	3422700	
RVS_(II)-3424407	3424407	3424407	
RVS_(II)-3425542	3425538	3425542	
RVS_(II)-3426965	3426962	3426965	YES
RVS_(I)-3427072	3427070	3427072	YES
RVS_(II)-3430014	3430014	3430014	
RVS_(I)-3430626	3430626	3430626	
RVS_(II)-3430688	3430688	3430688	
FWD_(III)-3431671	3431671	3431674	YES
FWD_(II)-3431729	3431729	3431729	
FWD_(II)-3431820	3431820	3431820	
FWD_(I)-3436012	3436012	3436012	
RVS_(II)-3436696	3436696	3436696	
RVS_(II)-3440588	3440587	3440588	YES
RVS_(II)-3441153	3441153	3441153	
RVS_(II)-3441374	3441373	3441374	
RVS_(II)-3442256	3442256	3442256	
RVS_(II)-3445023	3445023	3445023	
RVS_(I)-3446247	3446242	3446247	YES
RVS_(II)-3446475	3446473	3446475	
RVS_(I)-3451464	3451460	3451464	YES
RVS_(II)-3454592	3454592	3454592	
RVS_(II)-3464531	3464531	3464531	
RVS_(I)-3464770	3464770	3464770	
RVS_(II)-3465413	3465413	3465413	
RVS_(II)-3465801	3465801	3465801	
RVS_(II)-3469606	3469605	3469606	
RVS_(II)-3469747	3469747	3469747	
RVS_(I)-3472642	3472641	3472642	YES
RVS_(II)-3472742	3472742	3472742	
RVS_(II)-3474371	3474371	3474371	
RVS_(II)-3474529	3474529	3474529	YES
FWD_(II)-3475148	3475148	3475148	
RVS_(I)-3475491	3475491	3475491	
RVS_(III)-3475548	3475541	3475548	YES
FWD_(II)-3476523	3476523	3476523	
RVS_(I)-3476548	3476548	3476548	
RVS_(I)-3476581	3476581	3476581	
RVS_(II)-3483868	3483868	3483868	
FWD_(I)-3483975	3483975	3483976	YES
RVS_(I)-3483975	3483975	3483975	YES
RVS_(II)-3489671	3489671	3489671	YES
RVS_(II)-3490358	3490358	3490358	YES
FWD_(I)-3492009	3492009	3492009	YES
RVS_(II)-3513876	3513876	3513876	
RVS_(II)-3514274	3514274	3514274	YES
RVS_(II)-3515622	3515622	3515622	YES
RVS_(II)-3516523	3516523	3516523	
RVS_(I)-3517189	3517189	3517189	YES
RVS_(II)-3517307	3517307	3517307	
RVS_(I)-3517403	3517403	3517403	YES

FWD_(II)-3524488	3524488	3524488	
FWD_(II)-3526896	3526896	3526896	
RVS_(II)-3527787	3527786	3527787	
RVS_(II)-3528804	3528804	3528804	
RVS_(II)-3530486	3530486	3530486	
FWD_(I)-3530702	3530702	3530702	
FWD_(I)-3530813	3530813	3530813	
RVS_(II)-3534429	3534429	3534429	
FWD_(II)-3534460	3534460	3534460	
RVS_(II)-3534642	3534642	3534642	
RVS_(II)-3534708	3534708	3534708	YES
RVS_(I)-3534737	3534737	3534737	YES
RVS_(III)-3534798	3534795	3534798	
FWD_(II)-3535290	3535290	3535290	
RVS_(II)-3537887	3537886	3537887	
RVS_(II)-3537948	3537948	3537948	
FWD_(I)-3538080	3538080	3538080	
FWD_(II)-3541839	3541839	3541839	
RVS_(II)-3542898	3542898	3542898	
FWD_(II)-3543480	3543480	3543480	YES
FWD_(I)-3545759	3545759	3545759	
FWD_(III)-3545905	3545905	3545907	
RVS_(I)-3550522	3550522	3550522	
RVS_(I)-3550533	3550533	3550533	
FWD_(II)-3550761	3550761	3550761	
FWD_(I)-3551046	3551046	3551046	YES
RVS_(II)-3558948	3558948	3558948	
RVS_(II)-3559390	3559390	3559390	
FWD_(III)-3559993	3559993	3559993	YES
RVS_(III)-3561639	3561635	3561639	
RVS_(II)-3572961	3572961	3572961	
RVS_(I)-3573068	3573068	3573068	
RVS_(II)-3576250	3576250	3576250	
FWD_(II)-3576858	3576858	3576859	
FWD_(II)-3578988	3578988	3578988	
RVS_(III)-3579042	3579037	3579042	YES
FWD_(II)-3579134	3579134	3579134	
RVS_(II)-3581409	3581408	3581409	
FWD_(II)-3584943	3584943	3584943	
RVS_(II)-3586631	3586631	3586631	
FWD_(II)-3596459	3596459	3596459	
RVS_(I)-3598873	3598872	3598873	YES
RVS_(I)-3598887	3598887	3598887	YES
RVS_(I)-3599028	3599026	3599028	YES
RVS_(II)-3599057	3599057	3599057	
RVS_(I)-3602340	3602339	3602340	
FWD_(II)-3602350	3602350	3602350	
RVS_(II)-3603657	3603657	3603657	
FWD_(II)-3607178	3607178	3607178	
FWD_(I)-3607940	3607940	3607940	
RVS_(II)-3609783	3609783	3609783	
FWD_(II)-3611209	3611209	3611209	
FWD_(II)-3611651	3611651	3611651	
RVS_(II)-3617109	3617109	3617109	
RVS_(II)-3617188	3617188	3617188	
RVS_(I)-3628953	3628953	3628953	
RVS_(II)-3635460	3635460	3635460	
FWD_(II)-3635635	3635635	3635635	YES
FWD_(II)-3637307	3637307	3637307	

RVS_(I)-3637870	3637870	3637870	YES
FWD_(I)-3637960	3637960	3637960	
FWD_(II)-3638008	3638008	3638008	
FWD_(I)-3638788	3638788	3638788	
FWD_(II)-3638861	3638861	3638861	
FWD_(II)-3640274	3640274	3640274	
RVS_(I)-3646006	3646006	3646006	
FWD_(I)-3646085	3646085	3646085	
FWD_(I)-3646316	3646316	3646316	
RVS_(III)-3651260	3651256	3651260	
RVS_(I)-3651292	3651292	3651292	
RVS_(II)-3652626	3652626	3652626	
RVS_(I)-3654815	3654815	3654815	YES
FWD_(I)-3655824	3655824	3655824	YES
RVS_(II)-3656078	3656078	3656078	
RVS_(II)-3658347	3658347	3658347	
RVS_(I)-3662676	3662676	3662676	YES
FWD_(I)-3662887	3662887	3662887	YES
RVS_(I)-3663865	3663865	3663865	YES
RVS_(II)-3665865	3665865	3665865	
RVS_(I)-3667286	3667286	3667286	YES
RVS_(II)-3668863	3668863	3668863	
FWD_(II)-3669249	3669249	3669249	
RVS_(II)-3669319	3669319	3669319	
RVS_(II)-3674175	3674175	3674175	
FWD_(III)-3674204	3674204	3674204	
FWD_(I)-3677415	3677415	3677415	
RVS_(II)-3678613	3678613	3678613	
FWD_(II)-3680044	3680044	3680044	
RVS_(II)-3680124	3680124	3680124	
RVS_(III)-3681522	3681522	3681522	YES
RVS_(II)-3683450	3683450	3683450	
RVS_(II)-3684329	3684329	3684329	
RVS_(I)-3694389	3694388	3694389	
RVS_(II)-3694417	3694417	3694417	
FWD_(I)-3694420	3694420	3694420	
FWD_(II)-3694481	3694481	3694481	
RVS_(II)-3696176	3696176	3696176	
FWD_(III)-3697645	3697645	3697647	
FWD_(I)-3698159	3698159	3698161	YES
RVS_(II)-3698296	3698296	3698296	YES
FWD_(II)-3699836	3699836	3699836	
FWD_(III)-3703983	3703983	3703985	
FWD_(III)-3704002	3704002	3704005	
RVS_(I)-3705895	3705894	3705895	YES
RVS_(I)-3706721	3706721	3706721	
FWD_(II)-3706920	3706920	3706920	
RVS_(II)-3708603	3708603	3708603	YES
RVS_(II)-3711034	3711034	3711034	
FWD_(II)-3714525	3714525	3714525	
FWD_(II)-3715310	3715310	3715310	
FWD_(II)-3716251	3716251	3716251	
RVS_(II)-3716836	3716836	3716836	
RVS_(I)-3717121	3717121	3717121	YES
RVS_(II)-3717866	3717866	3717866	
FWD_(I)-3717912	3717912	3717912	YES
RVS_(II)-3718541	3718541	3718541	
FWD_(II)-3718873	3718873	3718873	
FWD_(II)-3719955	3719955	3719955	

RVS_(II)-3720574	3720574	3720574	
RVS_(I)-3723378	3723378	3723378	
FWD_(II)-3724875	3724875	3724875	
RVS_(II)-3730755	3730755	3730755	
RVS_(II)-3730807	3730807	3730807	
FWD_(II)-3733163	3733163	3733163	
FWD_(II)-3734615	3734615	3734615	
FWD_(II)-3735030	3735030	3735030	
RVS_(I)-3735264	3735264	3735264	
RVS_(I)-3735298	3735298	3735298	
FWD_(III)-3738979	3738979	3738981	
RVS_(I)-3739626	3739626	3739626	
FWD_(II)-3752610	3752610	3752610	
RVS_(II)-3759324	3759324	3759324	YES
RVS_(II)-3759763	3759763	3759763	
FWD_(II)-3764936	3764936	3764936	
FWD_(II)-3765176	3765176	3765176	
RVS_(II)-3769835	3769835	3769835	
FWD_(I)-3770212	3770212	3770212	
RVS_(II)-3772282	3772282	3772282	
RVS_(III)-3774472	3774468	3774472	
FWD_(III)-3774657	3774657	3774660	
FWD_(III)-3775104	3775104	3775106	
RVS_(II)-3780706	3780705	3780706	YES
RVS_(II)-3782228	3782226	3782228	
RVS_(II)-3782991	3782991	3782991	
RVS_(I)-3783079	3783078	3783079	
FWD_(II)-3783209	3783209	3783209	
FWD_(III)-3783250	3783250	3783250	YES
FWD_(II)-3784837	3784837	3784837	
RVS_(I)-3790601	3790601	3790601	
RVS_(II)-3791816	3791816	3791816	
FWD_(I)-3791962	3791962	3791962	YES
RVS_(II)-3803855	3803855	3803855	
RVS_(I)-3806263	3806261	3806263	YES
RVS_(II)-3807083	3807083	3807083	
RVS_(I)-3809838	3809835	3809838	
RVS_(I)-3810020	3810019	3810020	YES
RVS_(II)-3814601	3814601	3814601	YES
FWD_(I)-3814671	3814671	3814671	YES
FWD_(II)-3815724	3815724	3815724	YES
FWD_(I)-3819409	3819409	3819411	
FWD_(II)-3819946	3819946	3819946	
FWD_(II)-3820099	3820099	3820100	
RVS_(II)-3820120	3820120	3820120	
FWD_(II)-3823045	3823045	3823045	
FWD_(II)-3824701	3824701	3824701	
RVS_(II)-3827178	3827178	3827178	
FWD_(I)-3828454	3828454	3828454	YES
RVS_(II)-3830847	3830847	3830847	
FWD_(II)-3834139	3834139	3834139	
FWD_(I)-3834221	3834221	3834221	
FWD_(II)-3835743	3835743	3835743	
FWD_(II)-3836236	3836236	3836236	
RVS_(II)-3840444	3840444	3840444	
RVS_(I)-3851043	3851040	3851043	YES
RVS_(III)-3851159	3851155	3851159	
RVS_(I)-3851216	3851216	3851216	YES
FWD_(III)-3851360	3851360	3851362	YES

RVS_(II)-3851805	3851805	3851805	
FWD_(II)-3851886	3851886	3851886	
FWD_(II)-3851953	3851953	3851953	
RVS_(II)-3853642	3853642	3853642	
RVS_(I)-3853672	3853672	3853672	
FWD_(II)-3854418	3854418	3854418	
RVS_(II)-3854438	3854438	3854438	
FWD_(II)-3858587	3858587	3858587	
RVS_(I)-3864328	3864328	3864328	
RVS_(I)-3864368	3864368	3864368	
FWD_(II)-3864409	3864409	3864410	
FWD_(II)-3865631	3865631	3865631	
FWD_(II)-3865763	3865763	3865763	
FWD_(II)-3866421	3866421	3866421	
RVS_(II)-3873210	3873210	3873210	
FWD_(II)-3875500	3875500	3875500	
FWD_(II)-3875542	3875542	3875542	
FWD_(II)-3876112	3876112	3876112	
RVS_(I)-3878176	3878176	3878176	YES
FWD_(II)-3878243	3878243	3878243	
RVS_(II)-3879865	3879863	3879865	YES
FWD_(II)-3879885	3879885	3879885	
RVS_(II)-3880755	3880755	3880755	YES
RVS_(I)-3881985	3881985	3881985	YES
FWD_(I)-3882137	3882137	3882140	YES
FWD_(I)-3882263	3882263	3882265	YES
FWD_(III)-3882928	3882928	3882932	
FWD_(II)-3883035	3883035	3883035	
FWD_(II)-3884775	3884775	3884775	
RVS_(II)-3884863	3884863	3884863	
RVS_(I)-3886316	3886316	3886316	
FWD_(I)-3886434	3886434	3886436	YES
FWD_(II)-3887209	3887209	3887209	
FWD_(II)-3891875	3891875	3891875	
RVS_(II)-3895648	3895648	3895648	
FWD_(II)-3896522	3896522	3896522	
FWD_(II)-3902441	3902441	3902442	
RVS_(I)-3904722	3904721	3904722	YES
RVS_(II)-3912384	3912384	3912384	
RVS_(II)-3913212	3913212	3913212	
RVS_(II)-3913246	3913244	3913246	YES
RVS_(I)-3913400	3913400	3913400	
RVS_(II)-3917782	3917780	3917782	
RVS_(II)-3920749	3920744	3920749	
RVS_(II)-3920909	3920909	3920909	
RVS_(II)-3922919	3922919	3922919	
RVS_(I)-3924502	3924500	3924502	YES
FWD_(I)-3925155	3925155	3925155	YES
RVS_(I)-3929185	3929182	3929185	
FWD_(II)-3929239	3929239	3929239	
FWD_(II)-3929260	3929260	3929260	
FWD_(II)-3929345	3929345	3929345	
FWD_(I)-3931345	3931345	3931345	YES
FWD_(II)-3931989	3931989	3931989	
FWD_(II)-3935068	3935068	3935068	
FWD_(II)-3937349	3937349	3937349	
RVS_(III)-3939378	3939376	3939378	
FWD_(I)-3939539	3939539	3939539	
FWD_(II)-3939656	3939656	3939659	

FWD_(III)-3939828	3939828	3939830	
FWD_(II)-3941078	3941078	3941078	
FWD_(II)-3941578	3941578	3941578	
FWD_(II)-3942128	3942128	3942128	
FWD_(II)-3942328	3942328	3942328	
FWD_(II)-3943361	3943361	3943361	
RVS_(II)-3945142	3945142	3945142	
RVS_(II)-3946016	3946016	3946016	
FWD_(I)-3946073	3946073	3946073	
FWD_(I)-3948313	3948313	3948313	YES
RVS_(II)-3951492	3951492	3951492	
RVS_(II)-3955888	3955888	3955888	YES
FWD_(II)-3955939	3955939	3955939	YES
RVS_(I)-3957865	3957865	3957865	YES
RVS_(I)-3958004	3958004	3958004	
RVS_(II)-3958496	3958496	3958496	
RVS_(II)-3962364	3962364	3962364	
RVS_(II)-3962523	3962523	3962523	
FWD_(II)-3963396	3963396	3963396	
RVS_(I)-3963656	3963654	3963656	
FWD_(I)-3963673	3963673	3963675	YES
FWD_(II)-3963714	3963714	3963714	YES
FWD_(II)-3963763	3963763	3963763	
FWD_(II)-3964185	3964185	3964185	YES
FWD_(II)-3964218	3964218	3964218	
FWD_(II)-3965769	3965769	3965769	
FWD_(I)-3965913	3965913	3965914	
FWD_(II)-3966937	3966937	3966937	
RVS_(II)-3978855	3978855	3978855	
FWD_(I)-3980188	3980188	3980188	
FWD_(I)-3980385	3980385	3980385	
RVS_(III)-3982352	3982347	3982352	
FWD_(I)-3984455	3984455	3984457	YES
RVS_(II)-3984726	3984726	3984726	
RVS_(II)-3987494	3987494	3987494	
FWD_(III)-3988797	3988797	3988797	YES
RVS_(II)-3988932	3988932	3988932	
RVS_(I)-3988973	3988973	3988973	
FWD_(I)-3989022	3989022	3989022	YES
FWD_(II)-3989094	3989094	3989094	
FWD_(III)-3992438	3992438	3992438	
FWD_(III)-3992512	3992512	3992516	YES
FWD_(II)-3999215	3999215	3999215	
FWD_(II)-3999585	3999585	3999585	
RVS_(II)-4000475	4000475	4000475	
RVS_(II)-4002226	4002226	4002226	
FWD_(II)-4002946	4002946	4002946	
FWD_(II)-4005749	4005749	4005749	
FWD_(I)-4007124	4007124	4007127	
RVS_(II)-4011048	4011046	4011048	
RVS_(II)-4013411	4013411	4013411	
FWD_(II)-4013593	4013593	4013593	
FWD_(III)-4014409	4014409	4014413	YES
FWD_(II)-4015331	4015331	4015331	
FWD_(II)-4016653	4016653	4016653	
FWD_(II)-4016851	4016851	4016851	
FWD_(II)-4019408	4019408	4019408	
FWD_(II)-4019786	4019786	4019786	
FWD_(II)-4019933	4019933	4019933	

FWD_(III)-4022995	4022995	4022995	
RVS_(II)-4023072	4023072	4023072	
FWD_(I)-4029153	4029153	4029153	
FWD_(II)-4030942	4030942	4030942	
FWD_(I)-4033262	4033262	4033262	
FWD_(I)-4033379	4033379	4033382	
FWD_(III)-4033550	4033550	4033554	
FWD_(II)-4034801	4034801	4034801	
FWD_(II)-4035943	4035943	4035943	
FWD_(II)-4036143	4036143	4036144	
FWD_(II)-4037175	4037175	4037175	
FWD_(II)-4038156	4038156	4038156	
FWD_(II)-4038628	4038628	4038628	
RVS_(I)-4038915	4038915	4038915	
FWD_(I)-4040070	4040070	4040071	YES
FWD_(II)-4040414	4040414	4040414	YES
FWD_(II)-4040801	4040801	4040801	
FWD_(II)-4041190	4041190	4041190	
FWD_(III)-4041390	4041390	4041395	
FWD_(II)-4045565	4045565	4045565	
FWD_(I)-4047920	4047920	4047922	
RVS_(I)-4048818	4048818	4048818	
FWD_(I)-4049059	4049059	4049059	YES
FWD_(III)-4049329	4049329	4049331	YES
FWD_(II)-4049775	4049775	4049775	
FWD_(II)-4049966	4049966	4049966	YES
RVS_(I)-4051800	4051800	4051800	
RVS_(II)-4054664	4054664	4054664	
FWD_(I)-4056196	4056196	4056196	
RVS_(I)-4056244	4056244	4056244	YES
FWD_(I)-4056404	4056404	4056404	
FWD_(II)-4056717	4056717	4056717	
FWD_(II)-4057410	4057410	4057410	
FWD_(II)-4058442	4058442	4058442	
FWD_(III)-4061578	4061578	4061583	
FWD_(II)-4069008	4069008	4069008	
FWD_(II)-4071412	4071412	4071412	
FWD_(II)-4072668	4072668	4072668	
FWD_(II)-4073432	4073432	4073432	
FWD_(I)-4073552	4073552	4073552	
FWD_(II)-4074521	4074521	4074521	
FWD_(II)-4074990	4074990	4074990	
RVS_(II)-4079385	4079383	4079385	
RVS_(II)-4079593	4079593	4079593	
FWD_(II)-4083961	4083961	4083961	
RVS_(I)-4083988	4083988	4083988	
RVS_(II)-4085633	4085633	4085633	
RVS_(II)-4086057	4086057	4086057	
FWD_(II)-4091047	4091047	4091049	
FWD_(III)-4092335	4092335	4092339	
RVS_(III)-4092743	4092741	4092743	
FWD_(III)-4098782	4098782	4098782	YES
FWD_(II)-4101276	4101276	4101276	
FWD_(I)-4103808	4103808	4103808	YES
RVS_(II)-4104057	4104057	4104057	
FWD_(III)-4104351	4104351	4104355	
FWD_(II)-4104885	4104885	4104885	
FWD_(II)-4105068	4105068	4105068	
FWD_(I)-4105394	4105394	4105394	

FWD_(I)-4105497	4105497	4105497	YES
FWD_(II)-4106576	4106576	4106576	
FWD_(II)-4107929	4107929	4107929	YES
FWD_(II)-4108694	4108694	4108694	
RVS_(II)-4109384	4109384	4109384	
RVS_(III)-4109598	4109593	4109598	YES
FWD_(II)-4110312	4110312	4110312	
FWD_(II)-4110368	4110368	4110368	
FWD_(I)-4110951	4110951	4110954	
RVS_(II)-4112521	4112521	4112521	
FWD_(II)-4115853	4115853	4115853	
RVS_(I)-4116185	4116185	4116185	YES
FWD_(I)-4116450	4116450	4116450	
FWD_(I)-4116467	4116467	4116467	
RVS_(I)-4117382	4117380	4117382	YES
RVS_(II)-4120372	4120372	4120372	YES
RVS_(II)-4121548	4121548	4121548	
RVS_(II)-4121942	4121942	4121942	
RVS_(I)-4122534	4122534	4122534	YES
FWD_(I)-4124934	4124934	4124936	YES
FWD_(II)-4124975	4124975	4124975	
FWD_(III)-4125972	4125972	4125976	
RVS_(I)-4126490	4126490	4126490	YES
RVS_(I)-4126553	4126553	4126553	YES
RVS_(II)-4126788	4126788	4126788	
FWD_(II)-4130372	4130372	4130372	
FWD_(III)-4131765	4131765	4131768	
RVS_(II)-4131791	4131791	4131791	
FWD_(III)-4131811	4131811	4131813	
RVS_(III)-4137121	4137120	4137121	
FWD_(II)-4148383	4148383	4148383	
RVS_(III)-4151212	4151210	4151212	YES
FWD_(III)-4151367	4151367	4151371	
FWD_(I)-4156374	4156374	4156374	
RVS_(I)-4156419	4156419	4156419	
FWD_(III)-4156480	4156480	4156480	YES
RVS_(III)-4158846	4158846	4158846	
FWD_(III)-4159101	4159101	4159102	
FWD_(II)-4160108	4160108	4160108	
RVS_(II)-4160152	4160152	4160152	
RVS_(II)-4160947	4160947	4160947	
FWD_(II)-4161309	4161309	4161309	
FWD_(I)-4161422	4161422	4161422	YES
FWD_(II)-4162254	4162254	4162254	
FWD_(II)-4163351	4163351	4163351	
FWD_(I)-4163423	4163423	4163423	
RVS_(II)-4163646	4163646	4163646	
FWD_(I)-4164390	4164390	4164390	YES
FWD_(I)-4164507	4164507	4164510	YES
FWD_(II)-4165929	4165929	4165929	
FWD_(II)-4166279	4166279	4166280	
FWD_(II)-4167065	4167065	4167065	
FWD_(II)-4167265	4167265	4167266	
FWD_(II)-4168298	4168298	4168298	
FWD_(II)-4169417	4169417	4169417	
RVS_(II)-4173131	4173131	4173131	YES
FWD_(I)-4173404	4173404	4173404	
FWD_(II)-4173940	4173940	4173940	
FWD_(II)-4174998	4174998	4174998	

FWD_(II)-4175267	4175267	4175267	
FWD_(II)-4175302	4175302	4175302	
FWD_(I)-4176377	4176377	4176378	YES
FWD_(II)-4177646	4177646	4177649	YES
FWD_(II)-4177665	4177665	4177665	
FWD_(II)-4178563	4178563	4178563	
FWD_(II)-4183396	4183396	4183397	
FWD_(II)-4187787	4187787	4187787	
RVS_(I)-4188512	4188510	4188512	YES
RVS_(III)-4188664	4188662	4188664	
RVS_(I)-4194325	4194319	4194325	
RVS_(III)-4194886	4194886	4194886	YES
FWD_(I)-4198199	4198199	4198199	YES
FWD_(II)-4199918	4199918	4199918	
FWD_(II)-4201269	4201269	4201269	
FWD_(I)-4205886	4205886	4205886	
FWD_(I)-4205995	4205995	4205996	
FWD_(II)-4207417	4207417	4207417	
FWD_(II)-4207917	4207917	4207917	
FWD_(II)-4208467	4208467	4208467	
FWD_(II)-4208668	4208668	4208668	
FWD_(II)-4209700	4209700	4209700	
FWD_(II)-4210172	4210172	4210172	
FWD_(II)-4210679	4210679	4210681	
RVS_(II)-4212166	4212166	4212166	
RVS_(II)-4212606	4212606	4212606	
FWD_(II)-4213678	4213678	4213678	
RVS_(II)-4214515	4214515	4214515	
FWD_(III)-4216456	4216456	4216456	
RVS_(I)-4216630	4216629	4216630	
RVS_(I)-4221677	4221676	4221677	YES
FWD_(II)-4225625	4225625	4225625	YES
RVS_(II)-4225629	4225627	4225629	
RVS_(I)-4228192	4228192	4228192	
FWD_(III)-4228352	4228352	4228359	
RVS_(I)-4229680	4229680	4229680	
RVS_(I)-4231564	4231561	4231564	YES
FWD_(III)-4231744	4231744	4231744	YES
RVS_(II)-4231966	4231966	4231966	
RVS_(III)-4233524	4233520	4233524	
RVS_(II)-4234255	4234255	4234255	
RVS_(I)-4244488	4244488	4244488	YES
FWD_(I)-4244759	4244759	4244759	
FWD_(III)-4248358	4248358	4248362	
FWD_(III)-4248465	4248465	4248469	
RVS_(II)-4248584	4248583	4248584	
FWD_(I)-4250505	4250505	4250505	
FWD_(II)-4251884	4251884	4251884	
RVS_(II)-4254351	4254351	4254351	
FWD_(I)-4254376	4254376	4254376	
RVS_(II)-4254526	4254526	4254526	
RVS_(III)-4254660	4254659	4254660	
RVS_(II)-4254968	4254968	4254968	
FWD_(I)-4255110	4255110	4255110	YES
FWD_(III)-4257197	4257197	4257203	
FWD_(II)-4257316	4257316	4257316	
FWD_(II)-4257409	4257409	4257409	
RVS_(I)-4258056	4258055	4258056	
FWD_(I)-4261162	4261162	4261162	

FWD_(II)-4261189	4261189	4261189	
RVS_(III)-4262328	4262328	4262328	YES
FWD_(II)-4263612	4263612	4263612	YES
FWD_(II)-4264728	4264728	4264728	
FWD_(III)-4265105	4265105	4265105	YES
FWD_(III)-4267375	4267375	4267383	
RVS_(II)-4271957	4271957	4271957	YES
FWD_(III)-4272106	4272106	4272108	
FWD_(I)-4272121	4272121	4272121	YES
RVS_(II)-4272691	4272691	4272691	
FWD_(II)-4275389	4275389	4275389	
FWD_(II)-4275469	4275469	4275469	YES
RVS_(I)-4276090	4276090	4276090	YES
RVS_(II)-4276124	4276124	4276124	
FWD_(II)-4276561	4276561	4276561	
FWD_(II)-4279653	4279653	4279653	
RVS_(I)-4279744	4279744	4279744	
FWD_(III)-4283321	4283321	4283325	
RVS_(III)-4283405	4283404	4283405	
FWD_(III)-4285693	4285693	4285698	YES
RVS_(II)-4292437	4292437	4292437	
FWD_(II)-4292672	4292672	4292672	
FWD_(III)-4293856	4293856	4293857	
RVS_(I)-4297475	4297475	4297475	
FWD_(II)-4301435	4301435	4301435	
FWD_(II)-4307507	4307507	4307507	
RVS_(II)-4311043	4311040	4311043	
FWD_(II)-4315479	4315479	4315479	
RVS_(III)-4316023	4316023	4316023	
FWD_(III)-4323825	4323825	4323830	
RVS_(III)-4324858	4324853	4324858	
FWD_(I)-4327165	4327165	4327165	
FWD_(II)-4328797	4328797	4328797	
RVS_(II)-4331808	4331808	4331808	
RVS_(II)-4331992	4331992	4331992	
RVS_(I)-4336019	4336019	4336019	
RVS_(II)-4336045	4336045	4336045	
FWD_(II)-4337944	4337944	4337944	
FWD_(II)-4338878	4338878	4338878	
RVS_(I)-4339674	4339674	4339674	YES
RVS_(II)-4342152	4342152	4342152	
RVS_(II)-4342163	4342163	4342163	
RVS_(I)-4345844	4345844	4345844	
RVS_(II)-4346736	4346736	4346736	
RVS_(II)-4346750	4346750	4346750	
FWD_(II)-4347435	4347435	4347435	
RVS_(II)-4348296	4348296	4348296	
RVS_(I)-4349710	4349710	4349710	YES
FWD_(I)-4349836	4349836	4349836	
FWD_(II)-4350373	4350373	4350373	
FWD_(I)-4350537	4350537	4350537	
RVS_(II)-4352733	4352733	4352733	
RVS_(III)-4352828	4352828	4352828	YES
FWD_(II)-4354411	4354411	4354411	
FWD_(II)-4355736	4355736	4355736	
RVS_(I)-4358131	4358130	4358131	YES
RVS_(I)-4359984	4359984	4359984	YES
RVS_(I)-4360653	4360653	4360653	
RVS_(II)-4364925	4364925	4364925	

RVS_(II)-4364975	4364975	4364975	
RVS_(II)-4365135	4365135	4365135	
RVS_(I)-4366455	4366455	4366455	YES
FWD_(II)-4366626	4366626	4366626	YES
RVS_(II)-4367188	4367188	4367188	
RVS_(II)-4367989	4367989	4367989	
RVS_(II)-4368479	4368479	4368479	
FWD_(III)-4368639	4368639	4368639	
FWD_(II)-4370332	4370332	4370332	
FWD_(I)-4370784	4370784	4370786	YES
FWD_(II)-4371225	4371225	4371225	
RVS_(III)-4371320	4371320	4371320	
RVS_(II)-4372335	4372335	4372335	
FWD_(II)-4372597	4372597	4372597	
FWD_(I)-4373678	4373678	4373678	
FWD_(II)-4373772	4373772	4373772	
RVS_(I)-4374050	4374050	4374050	
RVS_(II)-4374497	4374497	4374497	
FWD_(I)-4374533	4374533	4374533	YES
FWD_(I)-4374822	4374822	4374822	YES
RVS_(II)-4377060	4377060	4377060	
RVS_(I)-4380442	4380438	4380442	YES
FWD_(II)-4381392	4381392	4381392	
FWD_(II)-4381765	4381765	4381765	
FWD_(II)-4381776	4381776	4381776	
FWD_(II)-4383230	4383230	4383230	
RVS_(II)-4388463	4388463	4388463	
FWD_(I)-4389584	4389584	4389584	
RVS_(I)-4389613	4389613	4389613	
FWD_(I)-4390242	4390242	4390242	
FWD_(I)-4390332	4390332	4390335	
FWD_(II)-4390761	4390761	4390761	YES
FWD_(II)-4391699	4391699	4391699	YES
FWD_(II)-4393202	4393202	4393202	
FWD_(II)-4393438	4393438	4393438	
FWD_(I)-4395123	4395123	4395123	YES
FWD_(II)-4397075	4397075	4397075	YES
FWD_(II)-4397422	4397422	4397422	
FWD_(I)-4397825	4397825	4397825	YES
FWD_(II)-4398243	4398243	4398243	YES
FWD_(II)-4401358	4401358	4401358	
FWD_(II)-4402337	4402337	4402338	
FWD_(III)-4402685	4402685	4402686	YES
RVS_(II)-4404166	4404166	4404166	
FWD_(III)-4404174	4404174	4404175	
RVS_(III)-4407260	4407257	4407260	
RVS_(I)-4414788	4414788	4414788	
RVS_(III)-4414893	4414893	4414893	
RVS_(II)-4415102	4415102	4415102	
FWD_(II)-4417950	4417950	4417950	YES
RVS_(II)-4420194	4420194	4420194	
FWD_(I)-4423048	4423048	4423049	YES
RVS_(II)-4424119	4424119	4424119	
RVS_(I)-4426843	4426843	4426843	YES
FWD_(I)-4426931	4426931	4426931	
FWD_(II)-4427803	4427803	4427803	YES
RVS_(II)-4431142	4431142	4431142	
FWD_(II)-4432106	4432106	4432106	
FWD_(I)-4434589	4434589	4434589	

RVS_(I)-4434653	4434653	4434653	YES
FWD_(II)-4434704	4434704	4434704	
FWD_(I)-4434741	4434741	4434742	
RVS_(II)-4435575	4435575	4435575	
RVS_(II)-4435591	4435591	4435591	
FWD_(I)-4437157	4437157	4437157	
FWD_(I)-4437435	4437435	4437435	
FWD_(II)-4437534	4437534	4437534	YES
FWD_(II)-4437732	4437732	4437732	
RVS_(II)-4438447	4438447	4438447	
FWD_(II)-4440274	4440274	4440274	
RVS_(II)-4440943	4440943	4440943	
RVS_(II)-4443026	4443026	4443026	
FWD_(I)-4445685	4445685	4445686	
FWD_(II)-4445839	4445839	4445839	
RVS_(II)-4445932	4445932	4445932	
FWD_(II)-4446427	4446427	4446427	
FWD_(III)-4446731	4446731	4446731	
RVS_(I)-4447710	4447710	4447710	YES
FWD_(II)-4451672	4451672	4451672	
FWD_(II)-4452531	4452531	4452531	
RVS_(III)-4453671	4453667	4453671	
FWD_(III)-4453778	4453778	4453785	
FWD_(I)-4455210	4455210	4455210	
FWD_(III)-4455967	4455967	4455967	YES
RVS_(III)-4455975	4455975	4455975	
RVS_(I)-4455993	4455993	4455993	
RVS_(I)-4464234	4464234	4464234	YES
FWD_(I)-4465385	4465385	4465385	YES
FWD_(II)-4468361	4468361	4468361	
RVS_(I)-4468968	4468967	4468968	YES
RVS_(II)-4470769	4470769	4470769	YES
RVS_(II)-4471002	4471002	4471002	
FWD_(I)-4472852	4472852	4472852	
FWD_(III)-4476466	4476466	4476470	YES
RVS_(II)-4477555	4477555	4477555	
RVS_(II)-4481952	4481949	4481952	YES
FWD_(III)-4482354	4482354	4482357	
FWD_(I)-4484083	4484083	4484083	
RVS_(I)-4484134	4484134	4484134	YES
RVS_(III)-4484165	4484155	4484165	YES
FWD_(II)-4484264	4484264	4484264	
FWD_(II)-4488103	4488103	4488103	
RVS_(II)-4488770	4488770	4488770	
RVS_(II)-4489594	4489594	4489594	
RVS_(II)-4494257	4494257	4494257	
FWD_(III)-4494404	4494404	4494407	
FWD_(II)-4494596	4494596	4494598	
RVS_(II)-4495568	4495567	4495568	
FWD_(II)-4495884	4495884	4495884	
FWD_(II)-4496266	4496266	4496266	
RVS_(II)-4497553	4497553	4497553	
FWD_(II)-4497610	4497610	4497610	
RVS_(II)-4498528	4498528	4498528	
FWD_(I)-4499861	4499861	4499861	
FWD_(II)-4500078	4500078	4500078	
RVS_(II)-4501528	4501528	4501528	
RVS_(I)-4501548	4501547	4501548	
FWD_(II)-4501549	4501549	4501549	

FWD_(II)-4504605	4504605	4504605	
RVS_(II)-4504926	4504926	4504926	
FWD_(II)-4505808	4505808	4505808	
RVS_(III)-4506659	4506655	4506659	
RVS_(II)-4512707	4512707	4512707	
FWD_(I)-4516390	4516390	4516397	
FWD_(I)-4516403	4516403	4516403	
FWD_(II)-4517137	4517137	4517137	
RVS_(I)-4518425	4518425	4518425	
RVS_(I)-4518438	4518438	4518438	
RVS_(II)-4518687	4518687	4518687	
RVS_(II)-4519236	4519236	4519236	
RVS_(II)-4521709	4521709	4521709	
RVS_(II)-4523847	4523847	4523847	
FWD_(II)-4523991	4523991	4523991	
FWD_(II)-4524716	4524716	4524716	
FWD_(I)-4525999	4525999	4526002	YES
FWD_(II)-4528408	4528408	4528408	
FWD_(II)-4531809	4531809	4531809	
RVS_(II)-4532083	4532083	4532083	
RVS_(I)-4532248	4532247	4532248	
FWD_(II)-4534042	4534042	4534042	
RVS_(I)-4534508	4534506	4534508	
FWD_(II)-4535002	4535002	4535002	
RVS_(I)-4537952	4537952	4537952	YES
FWD_(I)-4538709	4538709	4538709	
FWD_(II)-4539935	4539935	4539935	
FWD_(II)-4540245	4540245	4540245	
FWD_(III)-4541006	4541006	4541010	
FWD_(II)-4541672	4541672	4541672	
FWD_(II)-4541690	4541690	4541690	
FWD_(III)-4543138	4543138	4543142	
FWD_(II)-4543343	4543343	4543343	
FWD_(III)-4544004	4544004	4544008	
FWD_(II)-4549544	4549544	4549544	YES
FWD_(I)-4552503	4552503	4552503	
FWD_(II)-4554879	4554879	4554879	YES
RVS_(II)-4556277	4556277	4556277	
FWD_(II)-4559522	4559522	4559522	
RVS_(I)-4566778	4566778	4566778	
RVS_(II)-4567341	4567341	4567341	
RVS_(II)-4568764	4568764	4568764	
FWD_(I)-4570183	4570183	4570183	
FWD_(II)-4572168	4572168	4572168	
FWD_(II)-4574801	4574801	4574801	
FWD_(II)-4576930	4576930	4576930	
RVS_(I)-4577385	4577385	4577385	
FWD_(III)-4577858	4577858	4577858	YES
RVS_(II)-4581254	4581253	4581254	
RVS_(I)-4584834	4584834	4584834	
RVS_(I)-4584923	4584923	4584923	
FWD_(III)-4584937	4584937	4584939	
FWD_(II)-4589656	4589656	4589656	YES
FWD_(II)-4591710	4591710	4591710	
RVS_(II)-4591964	4591963	4591964	
RVS_(I)-4593889	4593889	4593889	
RVS_(I)-4597517	4597517	4597517	
RVS_(II)-4597546	4597544	4597546	
RVS_(II)-4598187	4598187	4598187	

RVS_(I)-4600931	4600928	4600931	
FWD_(II)-4602630	4602630	4602630	
RVS_(I)-4604460	4604458	4604460	
FWD_(II)-4604728	4604728	4604728	
FWD_(II)-4605804	4605804	4605804	YES
FWD_(II)-4606412	4606412	4606412	
FWD_(III)-4609176	4609176	4609176	YES
FWD_(III)-4612276	4612276	4612281	
RVS_(III)-4612672	4612668	4612672	
FWD_(II)-4614702	4614702	4614702	YES
RVS_(I)-4615117	4615117	4615117	
FWD_(III)-4615300	4615300	4615300	YES
FWD_(II)-4617293	4617293	4617293	
FWD_(II)-4618254	4618254	4618254	
FWD_(II)-4618812	4618812	4618812	
FWD_(II)-4618853	4618853	4618853	
RVS_(II)-4620464	4620463	4620464	
RVS_(II)-4622835	4622835	4622835	
FWD_(II)-4622893	4622893	4622894	
FWD_(II)-4625326	4625326	4625326	
RVS_(II)-4626981	4626981	4626981	
RVS_(I)-4628588	4628588	4628588	
FWD_(II)-4628705	4628705	4628705	
FWD_(II)-4629591	4629591	4629591	
FWD_(II)-4630722	4630722	4630722	YES
FWD_(II)-4631693	4631693	4631693	
FWD_(II)-4631779	4631779	4631779	
RVS_(II)-4631796	4631796	4631796	
FWD_(II)-4632308	4632308	4632308	
RVS_(I)-4633377	4633377	4633377	YES
FWD_(II)-4635007	4635007	4635007	
RVS_(II)-4638533	4638533	4638533	YES
RVS_(II)-4638542	4638541	4638542	YES
RVS_(II)-4638554	4638553	4638554	YES
RVS_(II)-4638619	4638619	4638619	YES
RVS_(II)-4638713	4638713	4638713	YES
RVS_(II)-4638777	4638777	4638777	
RVS_(I)-4638828	4638826	4638828	YES
RVS_(I)-4638837	4638837	4638837	

Table S2. Transcription start sites identified for *E. coli.* * data extracted from (Salgado et al., 2006).

Gene	Strand	Left	Right	Nearest TSS	Length of leader (nt)	Function
E. coli						
pgpA	fwd	435812	436331	FWD_(I)-435804	8	Phosphatidylglycerophosphatase A
ymfK	rvs	1202156	1201481	RVS_(I)-1202156	0	e14 prophage; repressor protein phage e14
racR	rvs	1418265	1417788	RVS_(I)-1418265	0	Rac prophage; predicted DNA-binding transcriptional regulator
dicA	fwd	1645957	1646365	FWD_(I)-1645954	3	Qin prophage; predicted regulator for DicB
rhlB	rvs	3963653	3962387	RVS_(I)-3963653	0	ATP-dependent RNA helicase
S. coelicolor						
SCO0058	fwd	44252	44975	FOR-44252_(I)	1	hypothetical protein
SCO0155	fwd	145498	146122	FOR-145499_(I)	0	transcriptional regulator
SCO0185	fwd	173767	174946	FOR-173768_(III)	0	geranylgeranyl pyrophosphate synthase
SCO0193	rvs	183792	184797	REV-184798_(I)	0	DNA-binding regulator
SCO0214	fwd	204386	204800	FOR-204387_(I)	0	hypothetical protein
SCO0216	fwd	206479	210181	FOR-206480_(I)	0	nitrate reductase subunit alpha NarG2
SCO0298	rvs	292655	293519	REV-293520_(I)	0	LysR family transcriptional regulator
SCO0463	fwd	484317	484932	FOR-484318_(I)	0	hypothetical protein
SCO0513	fwd	547808	548900	FOR-547809_(I)	0	hypothetical protein
SCO0561	rvs	603384	603840	REV-603841_(I)	0	Fe regulatory protein
SCO0723 (*freK*)	fwd	767384	768074	FOR-767383_(III)	2	fructose transport system kinase
SCO0724	fwd	768425	769367	FOR-768417_(I)	9	transcriptional regulator
SCO0747	rvs	789336	789837	REV-789838_(III)	0	hypothetical protein
SCO0788	rvs	835237	835474	REV-835475_(I)	0	hypothetical protein
SCO0802	rvs	849193	850042	REV-850043_(I)	0	hypothetical protein
SCO0945	rvs	990248	991112	REV-991113_(I)	0	formamidopyrimidine-DNA glycosylase
SCO0946	rvs	991152	993243	REV-993244_(I)	0	regulatory protein
SCO1017	rvs	1072676	1073219	REV-1073220_(I)	0	lipoprotein
SCO1041	fwd	1097281	1098505	FOR-1097279_(III)	3	hypothetical protein
SCO1060	rvs	1117869	1119111	REV-1119115_(III)	3	transcriptional repressor
SCO1123	fwd	1180850	1181249	FOR-1180851_(I)	0	hypothetical protein
SCO1146	rvs	1204910	1205669	REV-1205670_(I)	0	lipoprotein
SCO1200	rvs	1271990	1272566	REV-1272567_(III)	0	regulatory protein
SCO1237	rvs	1310819	1311104	REV-1311105_(I)	0	hypothetical protein
SCO1249	rvs	1320979	1321207	REV-1321208_(I)	0	hypothetical protein

SCO1256	rvs	1327483	1327924	REV-1327925_(I)	0	hypothetical protein
SCO1302	rvs	1379125	1379752	REV-1379753_(I)	0	hypothetical protein
SCO1319	fwd	1394272	1395295	FOR-1394271_(I)	2	hypothetical protein
SCO1334	fwd	1411646	1411826	FOR-1411647_(III)	0	hypothetical protein
SCO1364	rvs	1442208	1442748	REV-1442749_(III)	0	hypothetical protein
SCO1369	fwd	1445965	1447180	FOR-1445963_(I)	3	two component system histidine kinase
SCO1382	rvs	1460735	1461209	REV-1461210_(I)	0	hypothetical protein
SCO1394	fwd	1479785	1482173	FOR-1479786_(I)	0	glycosyl hydrolase
SCO1422	rvs	1517860	1518445	REV-1518446_(I)	0	hypothetical protein
SCO1439 (*hisE*)	rvs	1535781	1536054	REV-1536058_(I)	3	phosphoribosyl-ATP pyrophosphatase
SCO1530	rvs	1636315	1636876	REV-1636877_(I)	0	hypothetical protein
SCO1540	rvs	1649732	1650185	REV-1650186_(I)	0	hypothetical protein
SCO1571	fwd	1681978	1682566	FOR-1681977_(III)	2	hypothetical protein
SCO1625	fwd	1739096	1740071	FOR-1739096_(I)	1	ribosomal pseudouridine synthase
SCO1640	rvs	1753889	1755251	REV-1755252_(I)	0	hypothetical protein
SCO1651	rvs	1765256	1766159	REV-1766160_(I)	0	hypothetical protein
SCO1663 (*cysS*)	rvs	1783106	1784336	REV-1784337_(I)	0	cysteinyl-tRNA synthetase
SCO1670	fwd	1789861	1790848	FOR-1789861_(I)	1	oxidoreductase
SCO1673	fwd	1794076	1794265	FOR-1794076_(I)	1	hypothetical protein
SCO1678	rvs	1797722	1798424	REV-1798425_(I)	0	transcriptional regulator
SCO1700	rvs	1820168	1820483	REV-1820484_(I)	0	hypothetical protein
SCO1714	rvs	1835003	1835573	REV-1835574_(I)	0	hypothetical protein
SCO1727	fwd	1847563	1848331	FOR-1847564_(I)	0	hypothetical protein
SCO1753	fwd	1874624	1875647	FOR-1874625_(I)	0	hypothetical protein
SCO1783	rvs	1908047	1908395	REV-1908396_(III)	0	hypothetical protein
SCO1784	fwd	1908437	1908785	FOR-1908437_(I)	1	hypothetical protein
SCO1792	rvs	1922127	1922769	REV-1922772_(I)	2	3-methyladenine DNA glycosylase
SCO1797	rvs	1926230	1926662	REV-1926663_(I)	0	hypothetical protein
SCO1808	fwd	1939009	1940242	FOR-1939007_(I)	3	phosphoserine phosphatase
SCO1809	rvs	1940313	1940832	REV-1940833_(I)	0	hypothetical protein
SCO1811	rvs	1941314	1941431	REV-1941432_(I)	0	hypothetical protein
SCO1820	fwd	1950274	1950754	FOR-1950274_(III)	1	hypothetical protein
SCO1860	rvs	1994049	1995057	REV-1995067_(I)	9	hypothetical protein
SCO1864	fwd	1998467	1998980	FOR-1998468_(I)	0	acetyltransferase
SCO1873	fwd	2007026	2007497	FOR-2007027_(I)	0	acetyltransferase

SCO1926	rvs	2058373	2059132	REV-2059133_(I)	0	DNA-binding protein
SCO1928	fwd	2060168	2061092	FOR-2060169_(I)	0	ABC transporter ATP-binding protein
SCO1953 (*uvrC*)	rvs	2087669	2089856	REV-2089857_(I)	0	excinuclease ABC subunit C
SCO1960	fwd	2098288	2098945	FOR-2098289_(I)	0	hydrolase
SCO1966	rvs	2105060	2107199	REV-2107200_(I)	0	excinuclease ABC subunit B
SCO1990	rvs	2127827	2128541	REV-2128542_(I)	0	hypothetical protein
SCO1991	fwd	2128657	2129218	FOR-2128658_(III)	0	hypothetical protein
SCO2003	rvs	2141658	2144382	REV-2144384_(III)	1	DNA polymerase I
SCO2014	rvs	2156336	2157773	REV-2157774_(I)	0	pyruvate kinase
SCO2034	rvs	2185227	2186259	REV-2186263_(I)	3	prolipoprotein diacylglyceryl transferase
SCO2038	rvs	2189420	2189612	REV-2189613_(III)	0	hypothetical protein
SCO2045	fwd	2194211	2194847	FOR-2194212_(III)	0	hypothetical protein
SCO2054 (*hisD*)	rvs	2201234	2202560	REV-2202561_(III)	0	histidinol dehydrogenase
SCO2066	fwd	2216805	2217516	FOR-2216800_(I)	6	hypothetical protein
SCO2070	fwd	2220880	2222029	FOR-2220881_(I)	0	hypothetical protein
SCO2073	rvs	2224210	2225155	REV-2225156_(I)	0	ribosomal large subunit pseudouridine synthase
SCO2105	rvs	2262880	2263246	REV-2263247_(I)	0	transcriptional regulator
SCO2112	rvs	2270304	2270952	REV-2270953_(I)	0	hypothetical protein
SCO2115	rvs	2272072	2273425	REV-2273426_(I)	0	2-dehydro-3-deoxyphosphoheptonate aldolase
SCO2159	fwd	2322388	2322598	FOR-2322389_(I)	0	hypothetical protein
SCO2196	fwd	2363401	2364106	FOR-2363402_(I)	0	hypothetical protein
SCO2197	rvs	2364216	2364684	REV-2364685_(I)	0	hypothetical protein
SCO2201	fwd	2367727	2368099	FOR-2367728_(I)	0	hypothetical protein
SCO2212	fwd	2376422	2376545	FOR-2376423_(I)	0	hypothetical protein
SCO2223	fwd	2387312	2388005	FOR-2387313_(I)	0	TetR family transcriptional regulator
SCO2233	fwd	2401779	2402643	FOR-2401778_(I)	2	hypothetical protein
SCO2305	fwd	2474062	2474989	FOR-2474063_(III)	0	ABC transporter ATP-binding protein
SCO2337	fwd	2508323	2508884	FOR-2508321_(I)	3	acetyltransferase
SCO2340	fwd	2510089	2510497	FOR-2510090_(I)	0	hypothetical protein
SCO2373	rvs	2543006	2544617	REV-2544618_(III)	0	tetracenomycin C efflux protein
SCO2462	fwd	2647781	2649230	FOR-2647782_(I)	0	sugar kinase
SCO2501	rvs	2698678	2699575	REV-2699585_(I)	9	DNA-binding protein
SCO2505	fwd	2704003	2704990	FOR-2704003_(III)	1	ABC-transporter metal-binding lipoprotein
SCO2550	rvs	2750934	2751450	REV-2751451_(I)	0	lipoprotein
SCO2557	fwd	2756985	2757810	FOR-2756985_(III)	1	hypothetical protein

SCO2615 (*valS*)	rvs	2838956	2841581	REV-2841582_(III)	0	valyl-tRNA synthetase
SCO2627	rvs	2854111	2854591	REV-2854598_(I)	6	ribose-5-phosphate isomerase B
SCO2648	rvs	2880081	2880369	REV-2880370_(I)	0	hypothetical protein
SCO2670	rvs	2906676	2907651	REV-2907652_(I)	0	O-methyltransferase
SCO2677	rvs	2916747	2918391	REV-2918392_(I)	0	ABC transporter ATP-binding protein
SCO2771	fwd	3022835	3023624	FOR-3022836_(I)	0	hypothetical protein
SCO2775	rvs	3026789	3027416	REV-3027417_(III)	0	TetR family transcriptional regulator
SCO2860	rvs	3116520	3116931	REV-3116932_(I)	0	rifampin ADP-ribosyl transferase
SCO2901	fwd	3154676	3155144	FOR-3154677_(I)	0	hypothetical protein
SCO2909	fwd	3161530	3161698	FOR-3161531_(III)	0	hypothetical protein
SCO2927	rvs	3178619	3179765	REV-3179766_(I)	0	4-hydroxyphenylpyruvate dioxygenase
SCO2966 (*smpB*)	rvs	3227049	3227529	REV-3227533_(I)	3	SsrA-binding protein
SCO2972 (*prfB*)	rvs	3232164	3233271	REV-3233272_(I)	0	peptide chain release factor 2
SCO2987	fwd	3257115	3257622	FOR-3257115_(III)	1	regulatory protein
SCO3036	fwd	3322265	3323225	FOR-3322266_(I)	0	2-phospho-L-lactate transferase
SCO3063	rvs	3355989	3356667	REV-3356668_(I)	0	two-component system reponse regulator
SCO3119	rvs	3420020	3421340	REV-3421341_(I)	0	hypothetical protein
SCO3133	fwd	3434528	3435026	FOR-3434528_(I)	1	MarR family regulatory protein
SCO3140	fwd	3441594	3441891	FOR-3441592_(III)	3	hypothetical protein
SCO3177	rvs	3481967	3483236	REV-3483237_(I)	0	hypothetical protein
SCO3191	rvs	3497180	3497357	REV-3497358_(I)	0	hypothetical protein
SCO3273	fwd	3618212	3618479	FOR-3618213_(I)	0	hypothetical protein
SCO3311	rvs	3662049	3663042	REV-3663043_(I)	0	delta-aminolevulinic acid dehydratase
SCO3348	rvs	3706397	3707327	REV-3707328_(I)	0	DNA-binding protein
SCO3349	fwd	3707398	3708214	FOR-3707399_(I)	0	hypothetical protein
SCO3350	rvs	3708277	3710074	REV-3710076_(I)	1	hypothetical protein
SCO3367	fwd	3725597	3726185	FOR-3725595_(I)	3	TetR family transcriptional regulator
SCO3369	fwd	3727303	3729892	FOR-3727304_(I)	0	large ATP-binding protein
SCO3378	rvs	3741762	3741942	REV-3741943_(I)	0	small membrane protein
SCO3390	rvs	3753278	3754484	REV-3754485_(I)	0	two component sensor kinase
SCO3392	rvs	3755655	3756807	REV-3756808_(I)	0	NADH dehydrogenase subunit NuoD2
SCO3407	rvs	3772214	3773342	REV-3773343_(I)	0	hypothetical protein
SCO3434	fwd	3794732	3796424	FOR-3794733_(I)	0	DNA polymerase I
SCO3542	rvs	3911609	3914879	REV-3914880_(III)	0	integral membrane protein with kinase activity
SCO3559	rvs	3934977	3935802	REV-3935803_(III)	0	oxidoreductase

SCO3576	rvs	3954682	3954844	REV-3954845_(III)	0	hypothetical protein
SCO3577	fwd	3954996	3955974	FOR-3954997_(I)	0	ion-transporting ATPase
SCO3580	fwd	3958242	3960513	FOR-3958243_(I)	0	transpeptidase
SCO3582	fwd	3961225	3962155	FOR-3961226_(I)	0	hypothetical protein
SCO3590	rvs	3967921	3968617	REV-3968618_(III)	0	two-component system response regulator
SCO3610	rvs	3988035	3989856	REV-3989857_(I)	0	peptide hydrolase
SCO3622	fwd	4001664	4003020	FOR-4001665_(III)	0	hypothetical protein
SCO3714	rvs	4089284	4090508	REV-4090509_(I)	0	transposase
SCO3736	rvs	4108735	4109227	REV-4109238_(I)	10	RNA polymerase ECF sigma factor
SCO3792	fwd	4169610	4171227	FOR-4169611_(III)	0	methionyl-tRNA synthetase
SCO3799	rvs	4179064	4179535	REV-4179536_(III)	0	hypothetical protein
SCO3810	rvs	4189064	4189715	REV-4189716_(III)	0	GntR family transcriptional regulator
SCO3818	fwd	4197519	4198176	FOR-4197520_(I)	0	two-component system response transcriptional regulator
SCO3822	rvs	4202552	4203092	REV-4203093_(I)	0	hypothetical protein
SCO3823	rvs	4204100	4205081	REV-4205082_(III)	0	quinone oxidoreductase
SCO3840	rvs	4222902	4223718	REV-4223719_(I)	0	hypothetical protein
SCO3943	rvs	4338033	4339146	REV-4339150_(III)	3	transcriptional regulator
SCO3956	fwd	4355989	4356952	FOR-4355984_(I)	6	ABC transporter ATP-binding protein
SCO3961	rvs	4360547	4361825	REV-4361826_(I)	0	seryl-tRNA synthetase
SCO3968	rvs	4368954	4369809	REV-4369813_(III)	3	hypothetical protein
SCO3979	fwd	4382029	4382620	FOR-4382030_(I)	0	TetR family transcriptional regulator
SCO4008	fwd	4401678	4402257	FOR-4401679_(I)	0	tetR family regulatory protein
SCO4026	rvs	4423548	4424019	REV-4424020_(I)	0	ATP binding protein
SCO4043	rvs	4436887	4437184	REV-4437187_(III)	2	hypothetical protein
SCO4094	fwd	4492827	4493178	FOR-4492828_(III)	0	hypothetical protein
SCO4109	fwd	4508514	4509459	FOR-4508513_(I)	2	oxidoreductase
SCO4111 (*trmB*)	rvs	4510884	4511700	REV-4511704_(III)	3	tRNA (guanine-N(7)-)-methyltransferase
SCO4145	rvs	4559964	4562289	REV-4562290_(I)	0	polyphosphate kinase
SCO4197	fwd	4606580	4607048	FOR-4606581_(III)	0	MarR family regulatory protein
SCO4198	fwd	4607361	4607787	FOR-4607362_(I)	0	DNA-binding protein
SCO4205	fwd	4614618	4615125	FOR-4614619_(I)	0	hypothetical protein
SCO4206	rvs	4615153	4615969	REV-4615970_(I)	0	AraC family transcription regulator
SCO4209 (*gpmA*)	fwd	4618019	4618781	FOR-4618020_(I)	0	phosphoglyceromutase
SCO4229	fwd	4633198	4634479	FOR-4633199_(I)	0	sensor kinase
SCO4256	fwd	4667075	4668515	FOR-4667076_(I)	0	hydrolytic protein

SCO4257	fwd	4668590	4670033	FOR-4668591_(I)	0	hydrolytic protein
SCO4303	rvs	4717613	4718222	REV-4718223_(I)	0	transcriptional regulator
SCO4309	fwd	4722390	4722771	FOR-4722391_(III)	0	hypothetical protein
SCO4321	rvs	4732069	4734673	REV-4734674_(I)	0	hypothetical protein
SCO4329	rvs	4739972	4741364	REV-4741368_(III)	3	hypothetical protein
SCO4332	rvs	4746027	4748436	REV-4748437_(I)	0	integral membrane ATPase
SCO4333	rvs	4748535	4748796	REV-4748797_(III)	0	hypothetical protein
SCO4352	fwd	4767683	4768676	FOR-4767683_(III)	1	oxidoreductase
SCO4401	rvs	4819766	4821083	REV-4821085_(III)	1	lipoprotein
SCO4490	fwd	4909613	4911071	FOR-4909614_(III)	0	decarboxylase
SCO4499	fwd	4917933	4918656	FOR-4917934_(I)	0	TetR family transcriptional regulator
SCO4522	rvs	4942976	4943453	REV-4943457_(I)	3	hypothetical protein
SCO4612	rvs	5036029	5037274	REV-5037275_(I)	0	amino acid transporter
SCO4633	rvs	5059024	5059681	REV-5059682_(I)	0	hypothetical protein
SCO4645	rvs	5069748	5070975	REV-5070976_(I)	0	aspartate aminotransferase
SCO4740	fwd	5153333	5155181	FOR-5153334_(I)	0	glucosamine--fructose-6-phosphate aminotransferase
SCO4745	fwd	5158507	5159683	FOR-5158505_(I)	3	alanine racemase
SCO4757	fwd	5168215	5169475	FOR-5168215_(I)	1	hypothetical protein
SCO4790	rvs	5210500	5211922	REV-5211923_(I)	0	hypothetical protein
SCO4791	fwd	5212112	5213405	FOR-5212113_(I)	0	two-component system sensor kinase
SCO4797	fwd	5218623	5221119	FOR-5218621_(III)	3	ATP-dependent DNA helicase II
SCO4848	fwd	5279852	5280092	FOR-5279853_(I)	0	hypothetical protein
SCO4879	fwd	5310554	5311886	FOR-5310555_(I)	0	hypothetical protein
SCO4896	rvs	5329529	5330840	REV-5330841_(I)	0	transport integral membrane protein
SCO4904	rvs	5336631	5337183	REV-5337184_(I)	0	hypothetical protein
SCO4907	rvs	5339524	5340202	REV-5340203_(III)	0	transcriptional regulator
SCO4924	rvs	5358533	5358662	REV-5358663_(I)	0	hypothetical protein
SCO4944	rvs	5377854	5378730	REV-5378731_(III)	0	DNA-binding protein
SCO4957	rvs	5392631	5393750	REV-5393751_(III)	0	hypothetical protein
SCO4962	fwd	5396673	5397903	FOR-5396674_(III)	0	threonine dehydratase
SCO4978	rvs	5415296	5416001	REV-5416002_(I)	0	hypothetical protein
SCO4992	fwd	5431031	5431676	FOR-5431032_(I)	0	hypothetical protein
SCO5042 (*fumC*)	rvs	5480623	5482009	REV-5482010_(I)	0	fumarate hydratase
SCO5045	fwd	5485653	5486337	FOR-5485654_(III)	0	hypothetical protein
SCO5048	fwd	5488243	5488768	FOR-5488235_(III)	9	hypothetical protein

SCO5056 (*xseA*)	rvs	5494688	5495897	REV-5495898_(III)	0	exodeoxyribonuclease VII large subunit
SCO5060	rvs	5499805	5500408	REV-5500409_(III)	0	hypothetical protein
SCO5152	rvs	5599075	5600209	REV-5600210_(I)	0	ATP-binding protein
SCO5160	fwd	5607429	5608080	FOR-5607428_(I)	2	hypothetical protein
SCO5185	fwd	5641823	5643236	FOR-5641824_(I)	0	peptidase
SCO5205	fwd	5664875	5666699	FOR-5664876_(I)	0	hypothetical protein
SCO5206	rvs	5666943	5667360	REV-5667361_(I)	0	hydrogen peroxide sensitive repressor
SCO5214	fwd	5673487	5674303	FOR-5673488_(I)	0	hypothetical protein
SCO5241	rvs	5702432	5703401	REV-5703402_(III)	0	hypothetical protein
SCO5242	fwd	5703528	5703987	FOR-5703529_(III)	0	hypothetical protein
SCO5245	rvs	5705752	5706013	REV-5706015_(I)	1	hypothetical protein
SCO5253	fwd	5713932	5714154	FOR-5713933_(I)	0	hypothetical protein
SCO5295	rvs	5769300	5769624	REV-5769625_(III)	0	hypothetical protein
SCO5329	fwd	5798772	5800470	FOR-5798773_(III)	0	hypothetical protein
SCO5332	fwd	5805822	5807334	FOR-5805822_(III)	1	hypothetical protein
SCO5336	rvs	5809421	5809892	REV-5809893_(I)	0	mutT-like protein
SCO5351	fwd	5818974	5819478	FOR-5818975_(I)	0	regulatory protein
SCO5352	fwd	5819599	5820643	FOR-5819600_(I)	0	arginyl tRNA synthetase
SCO5381	rvs	5850113	5850686	REV-5850687_(III)	0	hypothetical protein
SCO5398	rvs	5870022	5870463	REV-5870466_(III)	2	hypothetical protein
SCO5415	fwd	5883755	5885456	FOR-5883756_(I)	0	isobutyryl-CoA mutase A
SCO5435	rvs	5908032	5909691	REV-5909692_(I)	0	two-component sensor kinase
SCO5455	fwd	5940592	5941267	FOR-5940590_(I)	3	two-component system response regulator
SCO5486	fwd	5971927	5973097	FOR-5971925_(I)	3	pyridoxal-phosphate-dependent aminotransferase
SCO5487	rvs	5973133	5973862	REV-5973863_(I)	0	hypothetical protein
SCO5488 (*mnmA*)	fwd	5973923	5975054	FOR-5973921_(I)	3	tRNA-specific 2-thiouridylase MnmA
SCO5504	fwd	5991460	5992087	FOR-5991461_(III)	0	hypothetical protein
SCO5517	fwd	6010145	6010736	FOR-6010146_(I)	0	transcriptional regulator
SCO5530	fwd	6025337	6026690	FOR-6025338_(I)	0	hypothetical protein
SCO5535	fwd	6031664	6033248	FOR-6031665_(I)	0	carboxyl transferase
SCO5549	fwd	6048320	6049055	FOR-6048321_(I)	0	hydrolase
SCO5555	fwd	6054327	6054558	FOR-6054328_(I)	0	hypothetical protein
SCO5590	fwd	6096338	6096935	FOR-6096339_(I)	0	hypothetical protein
SCO5645	fwd	6143018	6144125	FOR-6143016_(I)	3	ribosomal RNA large subunit methyltransferase N
SCO5683	fwd	6186587	6187784	FOR-6186588_(I)	0	two-component system sensor kinase

SCO5693	fwd	6201819	6203796	FOR-6201820_(III)	0	acyl CoA dehydrogenase
SCO5694	fwd	6203921	6205178	FOR-6203922_(I)	0	1-deoxy-D-xylulose 5-phosphate reductoisomerase
SCO5746	fwd	6275222	6275900	FOR-6275223_(I)	0	hypothetical protein
SCO5752	fwd	6290144	6291626	FOR-6290145_(I)	0	hypothetical protein
SCO5766	fwd	6304453	6304648	FOR-6304454_(I)	0	hypothetical protein
SCO5784	fwd	6321290	6322367	FOR-6321291_(I)	0	two-component sensor
SCO5787	fwd	6324452	6325970	FOR-6324453_(I)	0	(dimethylallyl)adenosine tRNA methylthiotransferase
SCO5790	rvs	6327185	6327473	REV-6327478_(I)	4	hypothetical protein
SCO5791 (*miaA*)	fwd	6327655	6328594	FOR-6327656_(III)	0	tRNA delta(2)-isopentenylpyrophosphate transferase
SCO5793 (*dapF*)	fwd	6329271	6330141	FOR-6329272_(I)	0	diaminopimelate epimerase
SCO5812 (*rnhB*)	fwd	6356448	6357150	FOR-6356446_(I)	3	ribonuclease HII
SCO5826	fwd	6375778	6376357	FOR-6375779_(III)	0	hypothetical protein
SCO5842	fwd	6394528	6397648	FOR-6394529_(I)	0	hypothetical protein
SCO6031 (*hemE*)	fwd	6620000	6621068	FOR-6620001_(I)	0	uroporphyrinogen decarboxylase
SCO6041	fwd	6632493	6633978	FOR-6632494_(I)	0	protoporphyrinogen oxidase
SCO6079	fwd	6674121	6674673	FOR-6674122_(III)	0	hypothetical protein
SCO6119	fwd	6724306	6724990	FOR-6724307_(I)	0	AraC family transcription regulator
SCO6152	rvs	6753256	6753739	REV-6753740_(I)	0	small hydrophobic protein membrane protein
SCO6218	rvs	6831345	6831945	REV-6831956_(I)	10	phosphatase
SCO6237	fwd	6860881	6861364	FOR-6860882_(I)	0	regulatory protein
SCO6342	fwd	7000968	7001376	FOR-7000969_(I)	0	hypothetical protein
SCO6456	rvs	7140528	7141923	REV-7141924_(I)	0	hydrolytic protein
SCO6631	fwd	7358813	7359689	FOR-7358814_(I)	0	hypothetical protein
SCO6709	fwd	7460583	7461609	FOR-7460584_(I)	0	hypothetical protein
SCO7042	rvs	7834890	7835298	REV-7835299_(I)	0	MarR family transcriptional regulator
SCO7093	fwd	7881840	7884639	FOR-7881832_(I)	9	transcriptional regulator
SCO7269	rvs	8078531	8079047	REV-8079048_(I)	0	hypothetical protein
SCO7613	rvs	8439703	8442160	REV-8442161_(I)	0	hypothetical protein
SCO7665	rvs	8490753	8490963	REV-8490964_(I)	0	hypothetical protein
SCO7694	fwd	8531015	8531588	FOR-8531016_(I)	0	TetR family transcriptional regulator
SCO7767	rvs	8596582	8597410	REV-8597411_(I)	0	DNA-binding protein

Table S3. Leaderless mRNAs identified for *E. coli* and *S. coelicolor* .

tRNA	Strand	Left	Right	CCA	Sequence context	Position
E. coli						
tRNA4-AspGTC	fwd	236931	237007	yes	CCAc↓t↓a	237008/09/10
tRNA87-GlnCTG	rvs	695765	695839	yes	CCAa↓ttt↓at	695764/63/60
tRNA86-MetCAT	rvs	695887	695963	yes	CCAa↓at	695886/85
tRNA77-TyrGTA	rvs	1286760	1286845	yes	CCAta↓at	1286758/57
tRNA75-CysGCA	rvs	1989938	1990011	yes	CCA↓c↓ttt↓ct	1989938/37/36/33
tRNA74-GlyGCC	rvs	1990066	1990141	yes	CCAg↓tt	1990065/64
tRNA64-ArgACG	rvs	2816495	2816571	yes	CCAt↓at	2816494/93
tRNA63-SerGCT	rvs	2816575	2816667	yes	CCAttt↓gc	2816572/71
tRNA59-ThrGGT	rvs	3421602	3421677	yes	CCActt↓tt	3421599-98
tRNA34-AspGTC	fwd	3944895	3944971	yes	CCAccct↓a↓at	3944975/76/77
tRNA37-HisGTG	fwd	3980533	3980608	yes	CCAtt↓at	3980535/36
tRNA44-TyrGTA	fwd	4173495	4173579	yes	CCA↓a↓tt	4173579/80/81
S. coelicolor						
tRNA65-ValCAC	rvs	1641943	1642017	no	C↓CCgg	1641944/45
tRNA2-CysGCA	fwd	1656463	1656536	yes	C↓CAa	1656534/35
tRNA3-ValGAC	fwd	1656538	1656609	yes	CCAc↓tg	1656610/11
tRNA6-LeuGAG	fwd	1797166	1797253	yes	C↓CAgct	1797151/52
tRNA63-ValTAC	rvs	2523200	2523274	yes	C↓CAgg	2523201/02
tRNA62-MetCAT	rvs	2646860	2646933	yes	CCAc↓tc	2646858/59
tRNA57-ProTGG	rvs	2847576	2847652	yes	C↓CAcc	2847577/78
tRNA13-LeuTAG	fwd	3155187	3155273	yes	C↓CAgta	3155271/72
tRNA55-LeuTAA (BldA)	rvs	3380890	3380976	yes	C↓CAct	3380891/92
tRNA54-AlaCGC	rvs	3481839	3481912	yes	CCAc↓gc	3481837/38
tRNA17-GlyCCC	fwd	4062164	4062237	yes	C↓CAtg	4062235/36
tRNA52-LeuCAG	rvs	4226761	4226847	yes	C↓CAgc	4226762/63
tRNA50-IleGAT	rvs	4259791	4259867	yes	C↓CAgc	4259792/93
tRNA48-SerGGA	rvs	4456865	4456952	yes	C↓CAgt	4456866/67
tRNA47-GlyGCC	rvs	4465950	4466025	yes	C↓CAga	4465951/52
tRNA44-PheGAA	rvs	4490732	4490805	yes	CCAc↓ag	4490730/31
tRNA1-GlyGCC	fwd	1656352	1656424	no	GCTc↓gc	1656425/26
tRNA4-ValGAC	fwd	1656646	1656717	no	GCAc↓gc	1656718/19
tRNA7-LeuCAA	fwd	2155886	2155958	no	GCAc↓t↓ttt	2155959/60/61
tRNA60-AsnGTT	rvs	2647210	2647282	no	GAGc↓tc	2647208/09
tRNA58-AlaGGC	rvs	2782760	2782835	no	C↓AAcc	2782761/62

tRNA9-ArgTCT	fwd	2929681	2929756	no	C↓CGgg	2929754/55
tRNA11-LysCTT	fwd	3091827	3091900	no	GCAc↓ag	3091901/02
tRNA14-PseudoTTG	fwd	3422227	3422299	no	GAGc↓tc	3422300/01
tRNA53-ThrCGT	rvs	3908335	3908411	no	C↓CGaa	3908336/37
tRNA16-ProCGG	fwd	3963111	3963184	no	CGA↓c↓a↓g↓tg	3963184/85/86/87/88
tRNA51-AlaTGC	rvs	4243682	4243757	no	C↓CGtc	4243683/84
tRNA45-AspGTC	rvs	4486471	4486545	no	CCGc↓ag	4486469/70
tRNA43-AspGTC	rvs	4490830	4490904	no	CCGc↓tg	4490828/29
tRNA42-GluTTC	rvs	4490939	4491011	no	GCAc↓gc	4490937/38
tRNA21-LysTTT	fwd	4498149	4498221	no	CTAc↓tc	4498222/23
tRNA22-MetCAT	fwd	4505460	4505533	no	cg↓CTAc	4505530/31
tRNA25-ThrGGT	fwd	5061316	5061388	no	GCTc↓tg	5061389/90
tRNA26-MetCAT	fwd	5061435	5061507	no	CTAc↓tc	5061508/9
tRNA27-TrpCCA	fwd	5071215	5071290	no	C↓TAca	5071288/89
tRNA28-MetCAT	fwd	5664562	5664635	no	CTA↓c↓t	5664635/36/37
tRNA39-ArgCCG	rvs	5818721	5818795	no	C↓CCag	5818722/23
tRNA29-GlnCTG	fwd	6049148	6049219	no	CAGc↓tc	6049220/21
tRNA30-GluCTC	fwd	6049259	6049331	no	GTAc↓ag	6049332/33
tRNA31-GluCTC	fwd	6049394	6049469	no	C↓GAac	6049467/68
tRNA32-GlnCTG	fwd	6049487	6049558	no	CGAc↓tc	6049559/60
tRNA33-GluCTC	fwd	6049603	6049675	no	GTAc↓tc	6049676/77
tRNA35-LeuGAG	fwd	7300604	7300688	no	ACAc↓ac	7300689/90
tRNA36-ProGGG	fwd	7565958	7566031	no	CGAc↓tc	7566032/33

Table S4. Identifiable 3′ processing associated with tRNAs in *S. coelicolor* and *E. coli.*

sRNA	Flanking genes	Orientation of flanking genes and sRNA (sRNA is middle arrow)	Left	Right	Length (nt) °	Elements partially(†) or completely(*) overlapping with the sRNA	Annotation in databases	Previously verified experimentally
**Potential novel sRNAs**								
**Complex (predicted to produce mutliple secondary structures)**								
ecr0114 (u+)	*pdhR-aceE*	→→→	122852	122945	93	*tp2* †(←), *pdhR* †(→)		
ecr0174 (u+) ##	*dxr-ispU*	→→→	194784	194848	64			
ecr0210 (d+)	*yafE-mltD*	→→←	232367	232584	217	*yafE* †(→)		
ecr0375 (u+)	*yaiU-yaiV*	→→→	393639	393720	81	*yaiU* †(→), *yaiV* †(→)		
ecr1891 (d+)	*motA-flhC*	←←←	1975230	1975327	97	*flhC* †(←)		
ecr4051 (u+) ##	*sucD-mngR*	→→←	4261162	4261273	111			
ecr4224 (d+)	*chpS-chpB*	→→→	4446731	4446835	104	*chpB* *(→)		
**Simple (predicted to form a single extended stemloop; perhaps associated with REP-like element)**								
ecr0235 (u+)	*yafP-ykfJ*	→→→	253340	253465	125			
ecr0573 (u-)	*cusC-cusF*	→←→	596198	596332	134			
ecr0770 (d-) #	*ybhI-ybhJ*	→←→	802544	802660	116			
ecr2774 (u+)	*ygcW-yqcE*	←←→	2898315	2898388	73			
ecr2775 (u+) ##	*ygcW-yqcE*	←→→	2898527	2898590	63			
ecr4065 (d-)	*yjcE-yjcF*	→←←	4279660	4279744	84			
**Previously identified sRNAs**								
SokC (sof)	*mokC-nhaA*	←→→	16952	17030	78	*mokC* †(←)	A, B, D, E	Pedersen & Gerdes 1999, Conway *et al*., 2014
SroA (tpe79)	*tbpA-sgrR*	←←←	75470	75609	139	*tbpA* †(←)	A, D, F	Vogel *et al*., 2003
SgrS (RyaA)	*sgrR-setA*	←→→	77367	77595	228	*sgrT* *(→)	A, B, C, D, E, F	Vanderpool *et al*., 2004, Conway *et al*., 2014

I006	*proA-ykfI*	→←←	261977	262243	266	*I006* †(←), *proA* †(→), *thrW* *(→)		Sætrom *et al*., 2005, Conway *et al*., 2014
ecr0419 (u+)	*yajO-dxs*	←←←	437352	437510	158	*yajO* †(←)		Conway *et al*., 2014
ffs (4.5S sRNA component of SRP)	*atl-ybaA*	←→→	475646	475793	147	*ffs* *(→)	A, B, D, E, F	Conway *et al*., 2014
MicM (SroB, RybC, ChiX)	*ybaK -ybaP*	←→←	506426	506515	89	*ybap* †(←)	A, B, C, D, E, F	Vogel *et al*., 2003, Conway *et al*., 2014
IS019	*nmpC-essD*	←→→	576438	576505	67	*IS019* *(→)		Raghavan *et al*., 2011, Conway *et al*., 2014
SokE (ECK4512)	*ybdK-hokE*	←←→	606955	607019	64		B, D, E, F	Kawano *et al*., 2005
ecr0729 (d+)	*sucD-mngR*	→→←	764295	764375	80			Conway *et al*., 2014
rybA	*ybiP-mntR*	←←→	852035	852271	236	*rybA* *(←), *mntS* *(←)		Gerstle *et al*., 2012
RybB (p25)	*rcdA-ybjL*	→←←	887197	887281	84		A, B, C, D, E, F	Wassarman *et al*., 2001, Conway *et al*., 2014
k2	*ybjE-aqpZ*	←←←	914410	914525	115	*k2* *(→)		Raghavan *et al*., 2011
ECS020	*InsB-4-cspH*	→←←	1049560	1049936	376	*insB-4* *(→)		Shinhara *et al*., 2011, Conway *et al*., 2014
RdlA	*ldrA-ldrB*	←→←	1268546	1268658	112		A, B, D, E, F	Kawano *et al*., 2002, Conway *et al*., 2014
RdlB	*ldrB-ldrC*	←→←	1269081	1269193	112		A, B, D, E, F	Kawano *et al*., 2002
RdlC	*ldrC-chaA*	←→←	1269616	1269715	99		A, B, D, E, F	Kawano *et al*., 2002
McaS (IS061, IsrA)	*abgR-smrA*	→←→	1403678	1403777	99		A, C, F	Chen *et al*., 2002 , Conway et al., 2014
t29	*recE-racC*	←→←	1415447	1415590	143	*racC* †(←), *t29* †(→)		Raghavan *et al*., 2011
RydC	*cybB-ydcA*	→←→	1489466	1489551	85	*RydC**	A, B, C, E, D, F	Raghavan *et al*., 2011
SokB	*mokB-trg*	←→→	1490143	1490200	57	*mokB* †(←)	A, B, D, E, F	Kawano *et al*., 2005, Conway *et al*., 2014
IGRSTd	*cspB-cspF*	←←→	1639658	1639740	82	Raghavan IGRST*		Raghavan *et al*., 2011
ECS025	*cspF-ydfT*	→←←	1640375	1640447	72			Shinhara *et al*., 2011
IGRSTe	*ydfU-rem*	←←←	1642532	1642655	123	Raghavan IGRST*		Raghavan *et al*., 2011, Conway *et al*., 2014
IGRSTf	*flxA-ydfW*	→←←	1644765	1644847	82	Raghavan IGRST*		Raghavan *et al*., 2011, Conway *et al*., 2014
IS077, ECS010	*flxA-ydfW*	→←←	1645070	1645145	75			Shinhara *et al*., 2011, Raghavan *et al*., 2011, Conway *et al*., 2014
rydB (tpe7)	*sufA-ydiH*	←←←	1762690	1762794	104	*rydB* †(←)	A, B, C, D, E, F	Rivas *et al*., 2001, Conway *et al*., 2014
rydB	*sufA-ydiH*	←←←	1762730	1762794	64	*rydB**	A, B, C, E, D, F	Raghavan *et al*., 2011

RprA (psrA5, IS083)	*ydik-ydiL*	→→→	1768396	1768505	109		A, B, C, D, E, F	Argaman *et al*., 2001
ecr1743 (d+) ##	*ves-spy*	←←←	1823080	1823177	97	*spy* †(←)		Conway *et al*., 2014
RyeA (SraC, psrA8, tkpe79, IS091)	*pphA-yebY*	←→←	1921090	1921365	275	*ryeB* *(←)	A, B, C, D, E, F	Argaman *et al*., 2001, Conway *et al*., 2014
RyeB (tkpe79, SdsR)	*pphA-yebY*	←←←	1921125	1921231	106	*ryeA* *(→)	A, B, C, D, E, F	Wassarman *et al*., 2001, Conway *et al*., 2014
IS094	*sdiA-yecC*	←→←	1994904	1995095	191	*IS094* †(→), *G0-10699* †(→)		Raghavan *et al*., 2011, Conway *et al*., 2014
DsrA	*yodD-yedP*	→←→	2023243	2023337	94		A, B, C, D, E, F	Sledjeski & Gottesman 1995, Conway *et al*., 2014
ECS007	*insH-yoeA*	←←→	2066405	2066508	103			Shinhara *et al*., 2011
SibA (QUAD1a, tp11, ryeC)	*yegL-* SibB	←→→	2151335	2151555	220	*ibsA* *(←)	A, B, D, E, F	Fozo *et al*., 2008, Conway *et al*., 2014
SibB (QUAD1b, tpe60, RyeD)	SibA-*mdtA*	→→→	2151670	2151815	145	*ibsB* *(←)	A, B, D, E, F	Fozo *et al*., 2008, Conway *et al*., 2014
CyaR (RyeE)	*yegQ-ogrK*	→→←	2165138	2165320	182		A, B, C, D, E, F	Wassarman *et al*., 2001, Conway *et al*., 2014
MicF (stc)	*ompC-rcsD*	←→→	2311106	2311203	97		A, B, C, D, E	Andersen *et al*., 1987, Conway *et al*., 2014
ECS009	*yfdI-tfaS*	→←→	2468483	2468688	205	*ECS009* †(←), *yfdI* †(→)		Shinhara *et al*., 2011, Conway *et al*., 2014
ECS054	*intZ-yffL*	→←→	2558320	2558395	75	*ECS054* †(←), *yffL* †(→)		Shinhara *et al*., 2011
ECS174	*rodZ-rlmN*	←←←	2640965	2641027	62	*ECS174* †(←)		Shinhara *et al*., 2011
C0614	*sseA* -C0614	→←←	2651370	2651478	108	*C0614* †(←)		Tjaden *et al*., 2002, Conway *et al*., 2014
ECS011	IS128-RyfA	→←→	2651690	2651800	110	*ECS011* † (←), *IS128* †(→)		Shinhara *et al*., 2011, Conway *et al*., 2014
RyfA (PAIR3, tp1)	IS128-*sseB*	→→→	2651879	2652180	301		A, C, F	Wassarman et al., 2001, Conway *et al*., 2014
GlmY (tke1, SroF, SraJ, RyiA, k19)	*glrK-purL*	←←←	2689170	2689363	193		A, B, C, D, E, F	Rivas *et al*., 2001, Conway *et al*., 2014
RyfB phantom gene	*yfhL-oshC*	→←→	2698080	2698400	320	*ECS091* †(→), *shoB* *(→)	A, B, D, E, F	Kawano *et al*., 2005, Shinhara *et al*., 2011
OhsC (RyfC)	*shoB-acpS*	←→←	2698542	2698620	78		A, B, D, E, F	Kawano *et al*., 2005, Conway *et al*., 2014
RyfD	*clpB-yfiH*	←←←	2732170	2732316	146	*clpB* †(←)	A, B, F	Kawano *et al*., 2006, Conway *et al*., 2014
tmRNA (*ssrA*)	*smpB-intA*	→→→	2753607	2754060	453	tmRNA*	A, B, C, E, D, F	Raghavan *et al*., 2011, Conway *et al*., 2014
MicA (SraD, psrA10)	*luxS-gshA*	←→←	2812824	2812900	76		A, B, C, D, E, F	Argaman *et al*., 2001, Conway *et al*., 2014
IS141	*iap-cas2*	→←←	2875905	2876486	581	*IS141* *(←)		Raghavan *et al*., 2011

CsrB	*yqcC-syd*	←←←	2922178	2922547	369		A, B, C, D, E, F	Conway *et al*., 2014
GcvB (IS145)	*gcvA-ygdI*	←→←	2940718	2940925	207		A, B, C, D, E, F	Argaman *et al*., 2001, Conway *et al*., 2014
OmrA (psrA12, RygA, PAIR2a, t59, SraE)	*aas-* OmrB	←←←	2974123	2974213	90		A, B, C, F	Argaman *et al*., 2001, Conway *et al*., 2014
OmrB (PAIR2b, t59, RygB)	OmrA-*galR*	←←→	2974325	2974410	85		A, B, C, F	Vogel *et al*., 2003, Conway *et al*., 2014
SsrS (ssr)	*zapA-fau*	→→→	3053996	3054225	229		A, B, E, F	Wassarman *et al*., 2000, Conway *et al*., 2014
sibC	*fau-serA*	→←←	3054805	3055032	227	*sibC* *(→), *fau* †(→), *ibsC* *(←)	A, B, D, E, F	Fozo *et al*., 2008, Conway *et al*., 2014
SibC (QUAD1c, t27, RygC)	*fau-serA*	→→←	3054873	3055015	142	*ibsC* *(←)	A, B, D, E, F	Wassarman *et al*., 2001, Conway *et al*., 2014
ECS034	*zupT-ribB*	→←←	3181345	3181648	303	*ECS034* †(←)		Shinhara *et al*., 2011
SroG (HB_456)	*ribB-yqiC*	←←→	3182585	3182741	156		A, F	Vogel *et al*., 2002
SibD (tp8, C0730, IS156, QUAD1d, RygD)	*yqiK-sibE*	←←→	3192735	3192890	155	*ibsD* *(→)	A, B, D, E, F	Fozo *et al*., 2008, Conway *et al*., 2014
SibE (QUAD1e, rygE)	*ibsD-rfaE*	←←←	3193105	3193265	160	*ibsE* *(→)	A, B, D, E, F	Fozo *et al*., 2008
sraF	*ygjR-alx*	→→→	3236322	3236590	268	*sraF* *(→) phantom gene	A, C, D, E, F	Argaman et al., 2001, Conway *et al*., 2014
rnpB	*yhaC-garK*	→←←	3268237	3268616	379	RNaseP*	A, B, C, E, F	Raghavan *et al*., 2011, Conway *et al*., 2014
ArcZ (psrA16, SraH, RyhA)	*elbB-arcB*	←→←	3348599	3348721	122	*arcB* †(←)	A, B, C, D, E, F	Argaman *et al*., 2001, Conway *et al*., 2014
RyhB (psrA18, IS176, SraI)	*yhhX-yhhY*	←←→	3578940	3579042	102		A, B, C, D, E, F	Massé & Gotterman 2002
agrA	*dinQ-agrB*	←→→	3646085	3646172	87	*agrA* *	A, B, E, F	Weel-Sneve, R. *et al*. 2013, Shinhara *et al*., 2011, Raghavan *et al*., 2011
agrB ##	*agrA-arsR*	→→→	3646316	3646400	84	*agrB* *	A, B, E, F	Weel-Sneve, R. *et al*. 2013, Shinhara *et al*., 2011, Raghavan *et al*., 2011
GadY (IS183)	*gadW-gadX*	←→←	3662887	3662995	108		A, B, C, D, E, F	Chen *et al*., 2002, Conway *et al*., 2014
RdlD	*ldrD-yhjV*	←→→	3698159	3698270	111		A, B, D, E, F	Kawano *et al*., 2002
ecr3632 (u+)	*waaQ-waaA*	←←→	3806190	3806263	73			Conway *et al*., 2014
IstR-1	*ivbL-tisA*	←←→	3851130	3851216	86	*IstR-2* †(←)	A, C, E, F	Vogel *et al*., 2004
ECS050	*mnmE-tnaC*	→←→	3886215	3886316	101	*ECS050* *(←)		Shinhara *et al*., 2011
IS190	*bglG-phoU*	←←←	3904610	3904722	112			Raghavan *et al*., 2011

ecr3777 (d+) [##]	*ppiC-yifO*	←←←	3957925	3958004	79			Conway *et al*., 2014
GlmZ (SraJ, RyiA)	*aslA-hemY*	←→←	3984455	3984680	225		A, B, C, D, E, F	Argaman *et al*., 2001, Conway *et al*., 2014
Spot42 (spf)	*polA-yihA*	→→←	4047920	4048032	112		A, B, C, D, E	Joyce & Grindley 1982, Conway *et al*., 2014
CsrC (SraK, psrA21, RyiB, tpk2, IS198)	*yihA-yihI*	←→→	4049059	4049300	241		A, B, C, D, E, F	Weilbacher *et al*., 2003, Conway *et al*., 2014
ecr4484 (d+)	*cpxP-fieF*	→→→	4104351	4104410	59			Conway *et al*., 2014
OxyS	*argH-oxyR*	→←→	4156298	4156419	121		A, B, C, D, E, F	Altuvia *et al*., 1997
SroH (IS206)	*yjaZ-thiH*	→←←	4188340	4188512	172		A, C, D, E, F	Vogel *et al*., 2003, Conway *et al*., 2014
ECS163	*thiC-rsd*	←←←	4194200	4194325	125			Shinhara *et al*., 2011
ecr4037 (d+)	*malM-yjbL*	→→→	4248465	4248560	95	*malM* †(→)		Conway *et al*., 2014
RyjA (psrA24, SraL)	*soxR-yjcD*	→←→	4275945	4276090	145		A, B, C, D, E, F	Argaman *et al*., 2001, Conway *et al*., 2014
I014/ ECS109	*epmB -ecnA*	←←→	4373825	4374050	225	*ECS109* *(←), *I014* *(←), *efp* *(→)		Shinhara *et al*., 2011, Sætrom *et al*., 2005
mgtA	*treR-mgtA*	←→→	4465385	4465647	262	*mgtL* *(→), *mgtA* * (→)		Raghavan *et al*., 2011
IGRSTa	*yjgZ-insG*	→→←	4499861	4500185	324	Raghavan IGRST*, *insG* †(←)		Raghavan *et al*., 2011, Conway *et al*., 2014
ECS094	*insG-yjhB*	←←→	4501450	4501548	98	*insG* †(←), *ECS094* *(←)		Shinhara *et al*., 2011
sRNA associated with 5' of *yjhX*	*yjhX-yjhR*	←←→	4532010	4532248	238	*yjhX* †(←)		Raghavan *et al*., 2011
IGRSTc	*nanC-fimB*	←←→	4537775	4537952	177	Raghavan IGRST*		Raghavan *et al*., 2011
IGRSTb	*nanC-fimB*	←→→	4538709	4538880	171	Raghavan IGRST*		Raghavan *et al*., 2011
sRNA associated with 5' of *mdtM*	*mdtM-yjiP*	←←→	4566620	4566778	158	*mdtM* †		Raghavan *et al*., 2011
SymR (RyjC)	*symE-hsdS*	←→←	4577858	4577942	84	*symE* †(←)	A, B, C, D, E, F	Kawano *et al*., 2007, Conway *et al*., 2014
**REP elements detected (this study)**								
REP6 cluster	*araD-araA*	←←←	66575	66815	240	REP6 cluster		
REP6 cluster	*araD-araA*	←→←	66580	66815	235	REP6 cluster		
REP7a, REP7b	*yabI-thiQ*	→→←	72132	72222	90	REP7a, REP7b		
REP16a, REP16b	*nlpE-yaeF*	→←←	216048	216327	279	*yaeF* †(→), REP16a, REP16b		
REP16a, REP16b	*nlpE-yaeF*	→→←	216051	216145	94	REP16a, REP16b		
REP19 cluster	*rayT-lfhA*	→→←	248144	248340	196	REP19 cluster		
REP23 cluster	*yahG-yahI*	→→→	338972	339343	371	*yahH* * (→), REP23 cluster		
REP23 cluster	*yahG-yahI*	→←→	338972	339343	371	*yahH* *(→), REP23 cluster		

REP25 cluster	*prpB-prpC*	→→→	348851	349195	344	REP25 cluster
REP26 cluster	*prpE-codB*	→→→	353860	353995	135	REP26 cluster
REP31 cluster	*mhpE-mhpT*	→←→	374140	374595	455	REP31 cluster
REP32 cluster	*yaiL-frmB*	→←←	376550	376816	266	*frmB* †(←), REP32 cluster
REP35 cluster	*mak-araJ*	→←←	410297	410495	198	REP35 cluster
REP35 cluster	*mak-araJ*	→→←	410300	410525	225	*araJ* †(←), REP35 cluster
REP46a, REP46b	*gsk-ybaL*	→→←	500636	500760	124	*gsk* †(→), REP46a, REP46b
REP48 cluster	*ybaQ-copA*	→←←	507803	508044	241	REP48 cluster
REP48 cluster	*ybaQ-copA*	→→←	507804	508049	245	REP48 cluster
REPv50b	*glxK-allE*	→→←	542360	542450	90	REPv50b
REP52 cluster	*ybcF-purK*	→←←	550573	550737	164	REP52 cluster
REP52 cluster	*ybcF-purK*	→→←	550574	550740	166	REP52 cluster
REPv54a, REP54b	*entD-fepA*	←→←	609317	609465	148	REPv54a, REP54b
REP67 cluster	*sdhB-sucA*	→←→	757623	757752	129	*b0725* †(→) phantom gene, *sdhB* †(→), REP67 cluster
REPv75a, REP75b	*rhlE-ybiA*	→→←	831498	831615	117	REPv75a, REP75b
REPv79a, REP79b	*ybiT-ybiU*	→←←	856835	856941	106	REPv79a, REP79b
REP90	*appA-etk*	→←←	1041140	1041279	139	etk†(←), REP90
REP144 cluster	*wcak-wzxC*	←←←	2116485	2116680	195	REP144 cluster
REP144 cluster	*wcak-wzxC*	←→←	2116487	2116683	196	REP144 cluster
REP150	*yegS-gatR*	→←←	2167660	2167783	123	*gatR* †(←), REP150
REP161 cluster	*eco-mqo*	→←←	2302450	2303100	650	REP161 cluster
REP163a, REP163b	*ubiG-yfaL*	→→←	2338330	2338425	95	REP163a, REP163b
REP164 cluster	*nrdA-nrdB*	→←→	2345175	2345352	177	*nrdA* †(→), REP164 cluster
REPv188a, REP188b	*srmB-yfiE*	→←←	2712290	2712411	121	REPv188a, REP188b
REP197a	*ascB-hycl*	→→←	2840407	2840485	78	*ascB* †(→) , REP197a
REP221a, REP221b	*yghU-hybG*	→←←	3137643	3137731	88	REP221a, REP221b
REP225 cluster	*cca-bacA*	→←←	3201155	3201282	127	REP225 cluster
REP233a, REP233b	*higB-rlmG*	←←←	3232640	3232783	143	*rlmG* †(←), REP233a, REP233b
REP245 cluster	*tldD-yhdP*	←→←	3390096	3390395	299	*ECS187* †(←), REP245 cluster
REP254a, REP254b	*gntT-malQ*	→→←	3545759	3546005	246	*gntT* †(→), REP254a, REP254b
REP266a, REPv266b	*dppB-dppA*	←→←	3703983	3704085	102	REP266a, REPv266b

REPv269a, REP269b	*avtA-ysaA*	→→←	3738979	3739100	121	*avtA* †(→), REPv269a, REP269b	
REP287a, REP287b	*aslB-aslA*	→←←	3982235	3982352	117	REP287a, REP287b	
REP292	*rrfA-mobB*	→←←	4038825	4038915	90	REP292	
REP299 cluster	*rhaD-rhaA*	←→←	4092335	4092745	410	REP299 cluster	
REP299 cluster	*rhaD-rhaA*	←←←	4092335	4092743	408	REP299 cluster	
REP306	*metL-metF*	→→→	4130372	4130475	103	REP306	
REP309 cluster	*ppc-argE*	←→←	4151367	4151720	353	REP309 cluster	
REP311 cluster	*aceA-aceK*	→→→	4216456	4216630	174	*aceK* †(→), REP311 cluster	
REP311 cluster	*aceA-aceK*	→←→	4216455	4216630	175	*aceK* †(→), REP311 cluster	
REP317 cluster	*ubiA-plsB*	→→←	4251884	4252060	176	*ubiA* †(→), REP317 cluster	
REP320a, REP320b	*yjcH-acs*	←←←	4283318	4283405	87	REP320a, REP320b	
REP320a, REP320b	*yjcH-acs*	←→←	4283321	4283408	87	REP320a, REP320b	
REP321 cluster	*gltP-yjcO*	→→←	4293856	4294460	604	REP321 cluster	
REP323a, REP323b	*phnL-phnK*	←←←	4315935	4316023	88	REP323a, REP323b	
REP325 cluster	*yjdM-yjdN*	←→←	4323825	4324400	575	REP325 cluster	
REPv328a, REP328b	*yjeI-yjeJ*	→←←	4371215	4371320	105	REPv328a, REP328b	
REP331a, REPv331b	*rnr-rlmB*	→←→	4407160	4407260	100	REP331a, REPv331b	
REP342a, REP342b	*holC-pepA*	←→←	4482354	4482445	91	REP342a, REP342b	
REPt344	*yjhR-nanS*	→←←	4534405	4534508	103	REPt344	
REP346	*uxuB-uxuR*	→→→	4552503	4552582	79	REP346	
REP352 cluster	*yjjV-yjjW*	→→←	4612276	4612672	396	REP352 cluster, *ECS045* †(←)	
**Previously annotated sRNAs associated with REP elements**							
REPv75a, REP75b	*rhlE-ybiA*	→←←	831505	831647	142	REPv75a, REP75b	Conway *et al*., 2014
REP77b, REP77c	*ybiC-ybiJ*	→→←	836802	836885	83	REP77b, REP77c	Conway *et al*., 2014
REPv92a, REP92b	*ymdF-rutG*	→→←	1067564	1067730	166	REPv92a, REP92b	Conway *et al*., 2014
REPv160b, REP160c	*narP-ccmH*	→←←	2289215	2289327	112	REPv160b, REP160c	Conway *et al*., 2014
REP 171a, REP171b	*mnmC-yfcL*	→→←	2441802	2441900	98	REP 171a, REP171b	Conway *et al*., 2014
ECS048 (REP184 cluster)	*iscR-trmJ*	←→←	2660329	2660600	271	REP184 cluster, *ECS048* †(→)	Shinhara *et al*., 2011

ECS187 (REP245 cluster)	*tldD-yhdP*	←←←	3390096	3390444	348	ECS187†, REP245 cluster	Shinhara *et al*., 2011
IS174 (REP252)	*yhgF-feoA*	→←→	3537770	3537948	178	*IS174* †(←), REP252	Raghavan *et al*., 2011, Conway *et al*., 2014
REP294a, REP294b	*hemN-glnG*	→←←	4051440	4051800	360	*yshB* *(←), REP294a, REP294b	Conway *et al.*, 2014
REP295a, REP295b	*glnL-glnA*	←←←	4054447	4054664	217	*glnA* †(←), REP295a, REP295b	Conway *et al*., 2014
REP313a, REP313b	*pgi-yjbE*	→←→	4233438	4233524	86	REP313a, REP313b	Conway *et al*., 2014
REP337a, REP337b	*mpl-yjgA*	→→←	4455210	4455400	190	REP337a, REP337b	Conway *et al*., 2014
ECS045 (REP352 cluster)	*yjjV-yjjW*	→←←	4612276	4612672	396	REP352 cluster, *ECS045* †(←)	Shinhara *et al*., 2011

Table S5. Small RNAs in *E. coli*. As determined here from gRNA-seq data. IGRST is an abbreviation for Intergenic region with significant transcription. # sRNA probed by northern blotting. ## sRNA detected by northern blotting. Databases: A, Ecocyc; B, Ecogene; C, Rfam; D, BSRD; E, NCBIGene; F, RegulonDB. (Joyce & Grindley, 1982, Andersen et al., 1987, Sledjeski & Gottesman, 1995, Altuvia et al., 1997, Pedersen & Gerdes, 1999, Argaman et al., 2001, Rivas et al., 2001, Wassarman et al., 2001, Chen et al., 2002, Masse & Gottesman, 2002, Tjaden et al., 2002, Vogel et al., 2003, Weilbacher et al., 2003, Vanderpool & Gottesman, 2004, Kawano et al., 2005, Saetrom et al., 2005, Fozo et al., 2008, Shinhara et al., 2011, Gerstle et al., 2012, Raghavan et al., 2012, Burge et al., 2013, Keseler et al., 2013, Salgado et al., 2013, Weel-Sneve et al., 2013, Zhou & Rudd, 2013)

Name	Left	Right	Strand	Length (nt)	Annotation	Reference
**sRNAs previously undetected experimentally**						
	**Potential *cis* -encoded asRNAs**					
as0627	669652	669953	fwd	301	asRNA to 5' end of SCO0627	This study
as1602	1713303	1713628	fwd	325	sRNA; anti to 5' end of SCO1602	This study
as2632	2858915	2859169	rvs	254	sRNA; anti to 5' end of SCO2632	This study
as3470	3831524	3831586	fwd	62	sRNA; anti to 5' end of SCO3470	This study
asSER/ARG tRNA	4407607	4407796	rvs	189	asRNA to tRNA precursor	This study
as4126	4538660	4538796	rvs	136	sRNA, internal to SCO4126	This study
as5612	6111490	6111563	rvs	73	sRNA, anti to 5' end of SCO5612	This study
as5620	6118309	6118370	rvs	61	sRNA, anti to 5 end of SCO5620	This study
as5641	6140844	6140905	fwd	61	sRNA anti to 5' end of SCO5641	This study
as5842	6394437	6394796	rvs	359	sRNA; anti to 5' end of SCO5842	This study
as6405[#]	7069449	7069584	rvs	135	sRNA, anti to 5' end of SCO6405	This study
	**Homologues of known riboswitches and conserved elements**					
rs0985	1037861	1038075	fwd	214	cobalamin riboswitch of SCO0985	This study
rs1378	1457725	1457946	rvs	221	glycine riboswitch of SCO1378	This study
rs1443	1539048	1539415	rvs	367	riboflavin element of SCO1443	This study
RNase P RNA	2462885	2463314	fwd	429	RNA component of RNase P; M1 RNA	This study
rs2321	2491687	2491870	fwd	183	cobalamin riboswitch of SCO2321	This study
rs2347	2517342	2517637	rvs	295	Predicted SraF leader element of SCO2347	This study
rs3928	4321572	4321783	fwd	211	thi-box riboswitch of SCO3928	This study
rs4293	4708375	4708600	fwd	225	SAM riboswitch of SCO4293	This study
rs5472	5958850	5959117	rvs	267	glycine riboswitch of SCO5472	This study
	**Others**					
scr0471(d+)[#]	492858	493054	fwd	196	sRNA	This study
scr0747(d-)	789174	789322	fwd	148	sRNA	This study
scr1805(d+)	1933883	1933990	fwd	107	sRNA	This study
scr2092(u+)	2248676	2248850	rvs	174	sRNA	This study

scr2393(d-)	2565876	2565976	rvs	100	sRNA	This study
scr3201(d+)[#]	3509075	3509290	rvs	215	sRNA	This study
scr4197(d-)	4607118	4607264	rvs	146	sRNA	This study
scr4474(d-)[#]	4893640	4893835	rvs	195	sRNA	This study
scr4632(u+)	5055608	5055720	fwd	112	sRNA	This study
scr4825(d-)	5255368	5255433	rvs	65	sRNA	This study
scr4885(d+)	5319336	5319580	fwd	244	sRNA	This study
scr6405(u+)	7065757	7066083	fwd	326	sRNA	This study
**Predicted by bio-computational methods only**						
scr1906(d+)	2040550	2040933	rvs	383	new ORF?	Swiercz et al.
tmRNA[##]	3226540	3226999	rvs	459	tmRNA; 10Sa RNA; SsrA	Pánek et al.
scr3871(u-) [##]	4258729	4258917	rvs	188	sRNA	Swiercz et al.
scr3973(d-)	4375752	4375704	fwd	48	sRNA	Pánek et al. ; Swiercz et al.
SRP RNA[##]	4457035	4457120	fwd	85	4.5S RNA of signal recognition particle	Pánek et al.
**sRNAs previously detected experimentally**						
as0091	78047	78191	rvs	144	asRNA	Vockenhuber *et al* .
scr0991	1045891	1046270	fwd	379	cobalamin riboswitch of SCO991	Vockenhuber *et al* .
scr1104	1161587	1161700	rvs	113	sRNA	Vockenhuber *et al* . ; Moody *et al.*
scr1424(d+)	1519532	1519788	rvs	256	sRNA	Moody *et al.*
scr1594(d-)	1703981	1704250	fwd	269	sRNA	Swiercz *et al.* ; Moody *et al.*
scr1821	1950867	1950948	fwd	81	new ORF2	Vockenhuber et al.
scr2076	2226957	2227406	fwd	449	T-box leader of SCO2076	Vockenhuber *et al.* ; Swiercz *et al.*
scr2100(d-) [##]	2257985	2258213	rvs	228	sRNA	Swiercz *et al* . ; Moody *et al.*
rs2107	2264717	2264822	fwd	105	thi-box riboswitch of SCO2107	Moody *et al* .
scr2288(d-)	2458883	2458992	fwd	109	sRNA	Moody *et al* .
scr2445(d+)	2625429	2625648	fwd	219	sRNA	Moody *et al* .
scr2736-1	2982224	2982505	fwd	281	sRNA	Vockenhuber *et al* . ; Swiercz *et al* . ; Moody *et al* .
scr2736-2	2982570	2982745	fwd	175	sRNA	Vockenhuber *et al* . ; Swiercz *et al* .
scr2750(d-)	2996338	2996440	rvs	102	sRNA	Moody *et al* .
scr2822(d-) [##]	3082238	3082390	fwd	152	sRNA	Pánek *et al* . ; Swiercz *et al* . Moody *et al* .

scr2952	3208719	3208857	fwd	138	sRNA	Vockenhuber *et al*. ; Moody *et al.*
scr3202	3510095	3510186	rvs	91	sRNA	Vockenhuber *et al*. ; Moody *et al.*
as3252	3605514	3605596	fwd	82	sRNA, anti to 5 'end of SCO3252	Moody *et al.*
as3287	3636488	3636569	rvs	81	asRNA	Vockenhuber *et al*.
as3317	3669433	3669657	fwd	224	asRNA	Vockenhuber *et al*.
scr3436(d-)	3799059	3799372	rvs	313	sRNA	Moody *et al.*
as3496	3861776	3861898	fwd	122	asRNA	Vockenhuber *et al*.
scr3558	3933527	3933668	rvs	141	6C motif	Vockenhuber *et al*. ; Swiercz *et al*. ; Pánek *et al*. ; Moody *et al*.
6S RNA[#]	3934693	3934927	fwd	234	6S RNA; SsrS RNA	Vockenhuber *et al*. ; Pánek *et al*.
scr3920	4315358	4315484	fwd	126	sRNA	Vockenhuber *et al*. ; Swiercz *et al*. ; Moody *et al*.
scr3928	4323842	4324023	fwd	181	sRNA	Vockenhuber *et al*. ; Moody *et al.*
scr3931(u-)	4326707	4326773	rvs	66	sRNA	Moody *et al.*
as4108	4507126	4507328	fwd	202	ATP-sensing riboswitch of SCO4108	Moody *et al.*
scr4115	4515318	4515426	fwd	108	sRNA	Vockenhuber *et al*.
as4233[#]	4637278	4637493	rvs	215	sRNA, anti to 5' end of SCO4233?	Moody *et al.*
scr4389	4805579	4805789	rvs	210	sRNA	Vockenhuber *et al*. ; Swiercz *et al*. ; Moody *et al*.
as4394/5	4811714	4811828	rvs	114	asRNA to SCO4394/95	Moody *et al.*
as4566	4984053	4984400	rvs	347	asRNA	Vockenhuber *et al*.
as4618	5043145	5043221	fwd	76	sRNA, anti to 5' end of SCO4618	Moody *et al.*
scr4632	5055055	5055172	rvs	117	sRNA	Vockenhuber *et al*.
as4676	5108124	5108202	fwd	78	sRNA, anti to 5' end of SCO4676	Moody *et al.*
scr4800	5224234	5224319	rvs	85	alternative ORF8	Vockenhuber *et al*.
scr4827	5257254	5257339	fwd	85	sRNA	Vockenhuber *et al*.
scr5028(u+)	5462579	5462631	fwd	52	sRNA	Swiercz *et al*. ; Moody *et al*.
as5439	5912160	5912259	rvs	99	sRNA, anti to 5' end of SCO5439	Pánek *et al*. ; Moody *et al*.
scr5676	6176158	6176413	rvs	255	sRNA	Vockenhuber *et al*. ; Swiercz *et al*. ; Moody *et al*.
scr5822	6370296	6370789	rvs	493	new ORF5	Vockenhuber *et al*.
scr5917(u+)[#]	6484345	6484941	fwd	596	new ORF?	Moody *et al.*
scr6106	6706584	6706667	fwd	83	sRNA	Vockenhuber *et al*. ; Moody *et al.*
scr6925	7688336	7688466	fwd	130	sRNA	Vockenhuber *et al*. ; Moody *et al.*

scr7098(d-)	7888420	7888551	fwd	131	sRNA		Moody *et al.*

Table S6. Small RNAs in *S. coelicolor* . Labelling in parentheses indicates whether the sRNA is upstream (u) or downstream (d) of the nearest protein-coding gene and whether on the same (+) or opposite (-) strand. The prefix 'scr' is used for discrete RNAs of unknown function, 'as' is reserved for those that are antisense to genes, and 'rs' is served for riboswitches that appear to be active. # sRNA probed by northern blotting. ## sRNA detected by northern blotting.

Name	Left	Right	Strand	Length (nt)	Original annotation	Comment
as0091	78047	78191	rvs	145	asRNA	Confirmed
scr1104	1161587	1161700	rvs	114	sRNA	Confirmed
scr1821	1950867	1950948	fwd	82	new ORF2	Confirmed
scr2736-1	2982224	2982505	fwd	282	sRNA	Confirmed
scr2736-2	2982570	2982745	fwd	176	sRNA *	Confirmed
scr3202	3510095	3510186	rvs	92	sRNA *	Confirmed
as3287	3636488	3636569	rvs	82	asRNA	Confirmed
as3317	3669433	3669657	fwd	225	asRNA	Confirmed
as3496	3861776	3861898	fwd	123	asRNA	Confirmed
scr3558	3933527	3933668	rvs	142	6C motif	Confirmed
scr3559	3934693	3934927	fwd	235	6S RNA	Confirmed
scr3920	4315358	4315484	fwd	127	sRNA *	Confirmed
scr4115	4515318	4515426	fwd	109	sRNA *	Confirmed
scr4389	4805579	4805789	rvs	211	sRNA *	Confirmed
scr4632	5055055	5055172	rvs	118	sRNA *	Confirmed
scr4800	5224234	5224319	rvs	86	alternative ORF8	Confirmed
scr4827	5257254	5257339	fwd	86	sRNA	Confirmed
scr5822	6370296	6370789	rvs	494	new ORF5	Confirmed
scr6106	6706584	6706667	fwd	84	sRNA *	Confirmed
scr6925	7688336	7688466	fwd	131	sRNA *	Confirmed
scr0991	1045891	1046090	fwd	200	cis-encoded, cobRs	Confirmed, but extends to 1046270
scr2076	2226957	2227117	fwd	161	cis-encoded, t-box	Confirmed, but extends to 2227405
scr2952	3208719	3208810	fwd	92	sRNA *	Confirmed, but extends to 3208857
scr3928	4323842	4324023	fwd	182	sRNA	Confirmed, but extends to 432443, low expression
as4566	4984053	4984157	rvs	105	asRNA	Confirmed, but extends to 4984400
scr5676	6176284	6176413	rvs	130	sRNA *	Confirmed, but extends to 6176158
scr5529	6023430	6023545	fwd	116	sRNA	Confirmed; may extend to SCO5529
as0642	682646	682730	fwd	85	asRNA	Not verified
scr1980	2118877	2118959	rvs	83	alternative ORF6	Not verified
as2080	2233552	2233634	fwd	83	asRNA	Not verified
as2364	2533451	2533546	rvs	96	asRNA	Not verified
as2780	3034099	3034209	rvs	111	asRNA	Not verified
as3029	3312231	3312325	fwd	95	asRNA	Not verified

as3111	3412144	3412346	fwd	203	asRNA	Not verified
as3125	3425777	3425967	rvs	191	asRNA	Not verified
as3404	3770178	3770278	fwd	101	asRNA	Not verified
scr4132	4545730	4545843	fwd	114	sRNA	Not verified
as4672	5104194	5104588	fwd	395	asRNA	Not verified
as4675	5106760	5106941	fwd	182	asRNA	Not verified
as4692/3	5119179	5119518	rvs	340	asRNA	Not verified
as4699	5124039	5124366	rvs	328	asRNA	Not verified
as5028	5464169	5464418	rvs	250	asRNA	Not verified
as5721	6241794	6241937	rvs	144	asRNA	Not verified
scr6280	6937783	6937870	fwd	88	sRNA	Not verified
as6323	6983615	6983803	fwd	189	asRNA	Not verified
as6418	7087036	7087162	fwd	127	asRNA	Not verified
as6721	7476805	7476903	rvs	99	asRNA	Not verified
scr6908	7672451	7672670	fwd	220	sRNA	Not verified
as7201	8004633	8004746	rvs	114	asRNA	Not verified
scr7601	8427312	8427437	rvs	126	sRNA	Not verified
scr1601	1711967	1712074	rvs	108	sRNA *	Extends to SCO1600
scr3035	3321216	3321353	rvs	138	new ORF3	Extends to SCO3034
as3321	3673385	3673581	fwd	197	asRNA	Extends to SCO3322, low expression
scr3323	3675098	3675329	fwd	232	alternative ORF7	Extends to SCO3323
scr3580	3958028	3958181	rvs	154	sRNA	Extends to SCO3579
as3680	4064602	4064701	fwd	100	asRNA	Extends to SCO3681
scr4164	4580774	4580933	fwd	160	new ORF4	Extends to SCO4164
as4261	4674583	4674770	fwd	188	asRNA	Extends to SCO4262
scr4659	5088759	5088859	fwd	101	sRNA	Extends to SCO4659
scr4701	5127282	5127536	fwd	255	cis-encoded, S10	Extends to SCO4701
scr5856	6412335	6412512	rvs	178	alternative ORF9	Extends to SCO5855
as2247	2416288	2416397	fwd	110	asRNA	Low expression
as4567	4984635	4984793	rvs	159	asRNA	Segment of extended anti-sense transcription

Table S7. Comparison with prior RNA-seq analysis of *S. coelicolor.** expression verified by northern blotting (Vockenhuber *et al* .)

Position of TSS (this study)	TSS Class	Strand	Gene	Function	Annotated start of coding region	TSS relative to annotated start	Possible alternative start of coding sequence
1225868-1225868	I	rvs	SCO1166	integral membrane protein	1225983	115	1225833
1463578-1463580	I	rvs	SCO1385	hypothetical protein	1463926	346	1463461
1527054-1527054	I	rvs	SCO1431	membrane protein	1527104	50	1527053
2130679-2130680	III	fwd	SCO1994	integral membrane protein	2130596	84	2130695
3154574-3154576	I	rvs	SCO2900	membrane protein	3154582	8	3154516
3163178-3163178	I	rvs	SCO2912	hypothetical protein	3163225	47	3163177
3255126-3255126	III	fwd	SCO2985	integral membrane protein	3254989	137	3255132
3832248-3832248	I	rvs	SCO3471	extracellular agarase precursor	3832634	386	3832235
3836987-3836989	I	rvs	SCO3475	isomerase	3837003	14	3836952
4059496-4059496	I	fwd	SCO3676	integral membrane protein	4059415	81	4059496
4247966-4247967	I	rvs	SCO3862	hypothetical protein	4248081	114	4247928
5436351-5436351	I	rvs	SCO4997	hypothetical protein	5436359	8	5436272
5688404-5688405	I	rvs	SCO5227	redoxin	5688415	11	5688381
673581-673581	I	rvs	SCO0631	hypothetical protein	673633	52	673537
7174943-7174943	I	fwd	SCO6484	hypothetical protein	7174911	32	7174946

Table S8. Examples of *S. coelicolor* genes whose annotation merits review.

Name	Target	Sequence
**5' adaptor for RLM-PCR**		
S4	5'-monoP RNA	5'-ACAUGAGGAUUACCCAUGUCGAAGACAACAAAGAAGUUCAACUCUUUAUGUAUU
**Adaptor primers for RLM-PCR**		
RLM1	cDNA	5'- CGAAGACAACAAAGAAGTTCAACTC
RLM2	cDNA	5'- CATGAGGATTACCCATGTCG
**Transcript-specific primers for RLM-PCR**		
16S	16S rRNA	5'-GATCCAACCGCAGGTTCC
ftsT	*ftsT*	5'-GGCTTCTCAACAGGTGGTGT
pgpA	*pgpA*	5'-TGCCACGGATTACTCATCTTC
rhlB	*rhlB*	5'-TGGGCGTACAGTTATGAAACC
ymfK	*ymfK*	5'-GGCCTGTACCCATGATATGAC
**Primers for generation of riboprobe templates (*S. coelicolor*)**		
SRP_F (95)	SRP RNA	5'-CCGAAAAAAATAATCGCCAAC
SRP_Rt7	SRP RNA	5'-ATCC**TAATACGACTCACTATA**GGGACGCACCCGCCAGAG
tmRNA_F (301)	tmRNA	5'-CCGAAAAAAATAATCGCCAAC
tmRNA_Rt7	tmRNA	5'-ATCC**TAATACGACTCACTATA**GGGAATCAGGGCTTCTCCGTGT
LDS2_F (169)	scr3871(u-)	5'-ACACCAACAAGGCTGGACA
LDS2_Rt7	scr3871(u-)	5'-ATCC**TAATACGACTCACTATA**GGGGTGGGGCTAACAGGATGTGA
LDS3_F (114)	scr2822(d-)	5'-ATCACGGGGGCCAACT
LDS3_Rt7	scr2822(d-)	5'-ATCC**TAATACGACTCACTATA**GGGGCCTTGCACCTGCATTTC
LDS6_F (106)	scr2100(d-)	5'-GGCGGGAGCGAGACAC
LDS6_Rt7	scr2100(d-)	5'-ATCC**TAATACGACTCACTATA**GGGTCTCCTGCTCCCCCGTA
**Probes for northern blotting (*E. coli*)**		
ORL3	ecr0174(u+)	5'-CCACGGCATATCTGACCTTATAAAGCCAAC-3'
ORL34	ecr3777(d+)	5'-ACCTTTTCGGCTGTCTCTTCTCTCGTACTG-3'
ORL43	ecr2775(u+)	5'-CTCAAGGGGAGAAAACTTAGGGCCTCTATG-3'
ORL47	ecr4051(u+)	5'-CTGTATGTAGGGTACAGCACGATGAATCTG-3'
ORL30	ecr1743(d+)	5'-GTATTACCGTAGTAATGCAAGCGCGTCTCAG-3'
ORLagrB	AgrB	5'-ACTTTCCAGCCCTGAGTTGGTGGCTCTG-3'

Table S9. Oligonucleotides used in this study. The sizes of riboprobes are provided in parenthesis as part of the labelling of forward primers.The T7 promoter sequence is bold.

Figure S1

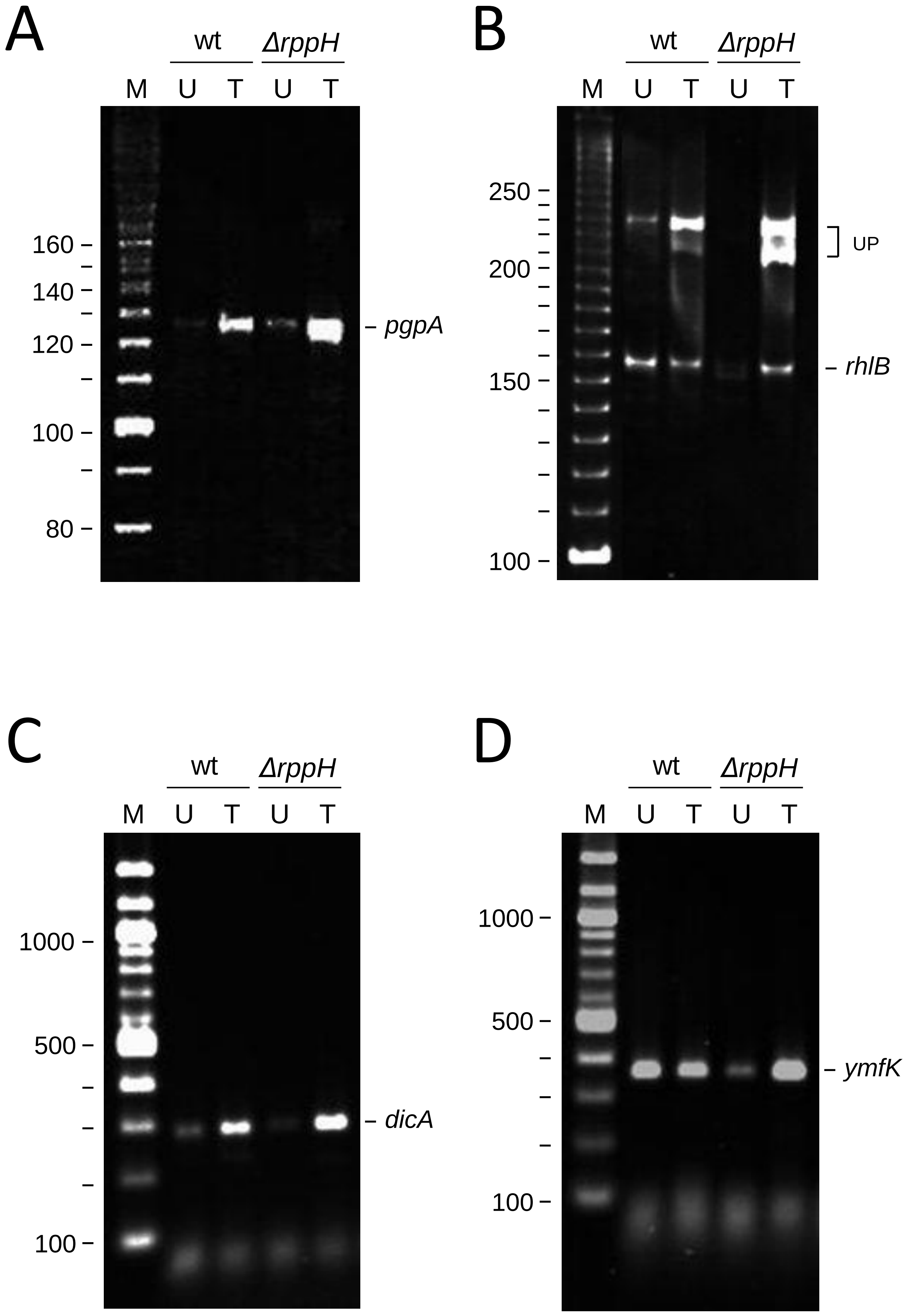
A
wt
ΔrppH
M
U
T
U
T
160
140
120
100
80
pgpA
B
wt
ΔrppH
M
U
T
U
T
250
200
150
100
UP
rhlB
C
wt
ΔrppH
M
U
T
U
T
1000
500
100
dicA
D
wt
ΔrppH
M
U
T
U
T
1000
500
100
ymfK

Fig. S1. RLM-RT-PCR analysis of *E. coli* leaderless mRNAs. **(A)**, **(B)**, **(C)** and **(D)** correspond to *pgpA*, *rhlB*, *dicA* and *ymfK*, respectively. RNA was analysed from strain BW25113 (labelled wt) and a congenic *ΔrppH* strain; both were growing exponentially. Prior to RLM-RT-PCR analysis, an aliquot of each sample was treated with TAP (labelled T). Aliquots of untreated samples (labelled U) were also analysed. Labelling on the left indicates the sizes of molecular markers from Invitrogen (labelled M). Amplicons of the size expected for the lmRNAs (see below) are indicated on the right of each panel. Amplicons corresponding to transcription upstream of the start codon (labelled UP) are also indicated for *rhlB*. The amplicons of *pgpA* and *rhlB* were separated using 10% polyacrylamide, while *dicA* and *ymfK* were separated using 2% agarose. No amplicons were produced without reverse transcription (data not shown).

Interpretation: For *pgpA*, *rhlB, dicA* and *ymfK* amplicons of the size expected for lmRNAs were produced; 125, 156, 300 and 335 bp, respectively. Amplicons of ~210 and ~225 bp were also produced for *rhlB* indicating the presence of leadered as well as leaderless mRNAs. An attempt to amplify the Rac-prophage transcript using our standard conditions was unsuccessful. For all four of the *E. coli* lmRNA that were amplifiable, the abundance of the amplicons increased substantially following TAP treatment of RNA from the *ΔrppH* mutant, which was consistent with the corresponding 5′ ends being the products of transcription initiation and not processing. Interestingly, amplicons were produced, albeit to a low level, before TAP treatment of RNA from the *ΔrppH* mutant. While this may suggest the presence of a second RNA pyrophosphohydrolase activity, it could be that some of the transcription is primed by nanoRNAs, a recently described class of sRNAs (Goldman et al., 2011, Nickels & Dove, 2011, Vvedenskaya et al., 2012), a proportion of which may have 5′-monophosphorylated ends. Similar results were obtained using RNA from the wild-type strain, with the exception that the abundance of the amplicon corresponding to the leaderless *rhlB* and *ymfK* transcripts was high without TAP treatment, which suggested that normally these transcripts are de-pyrophosphorylated by RppH (Deana et al., 2008). Cloning and sequencing of the *rhlB* amplicons produced from wild-type RNA following TAP treatment confirmed the presence of a TSS at precisely position +1 (relative to the start codon) and revealed another upstream at position -70 (data not shown). The latter corresponds to the largest amplicon of 225 bp. The TSS corresponding to the amplicon of ~210 bp remains unmapped. Notwithstanding the identification of transcription farther upstream than the start codon, the global RNA-seq data, which being derived from multiple fragments and should be reliably quantitative, indicates that the major form of *rhlB* mRNA is leaderless.

Figure S2

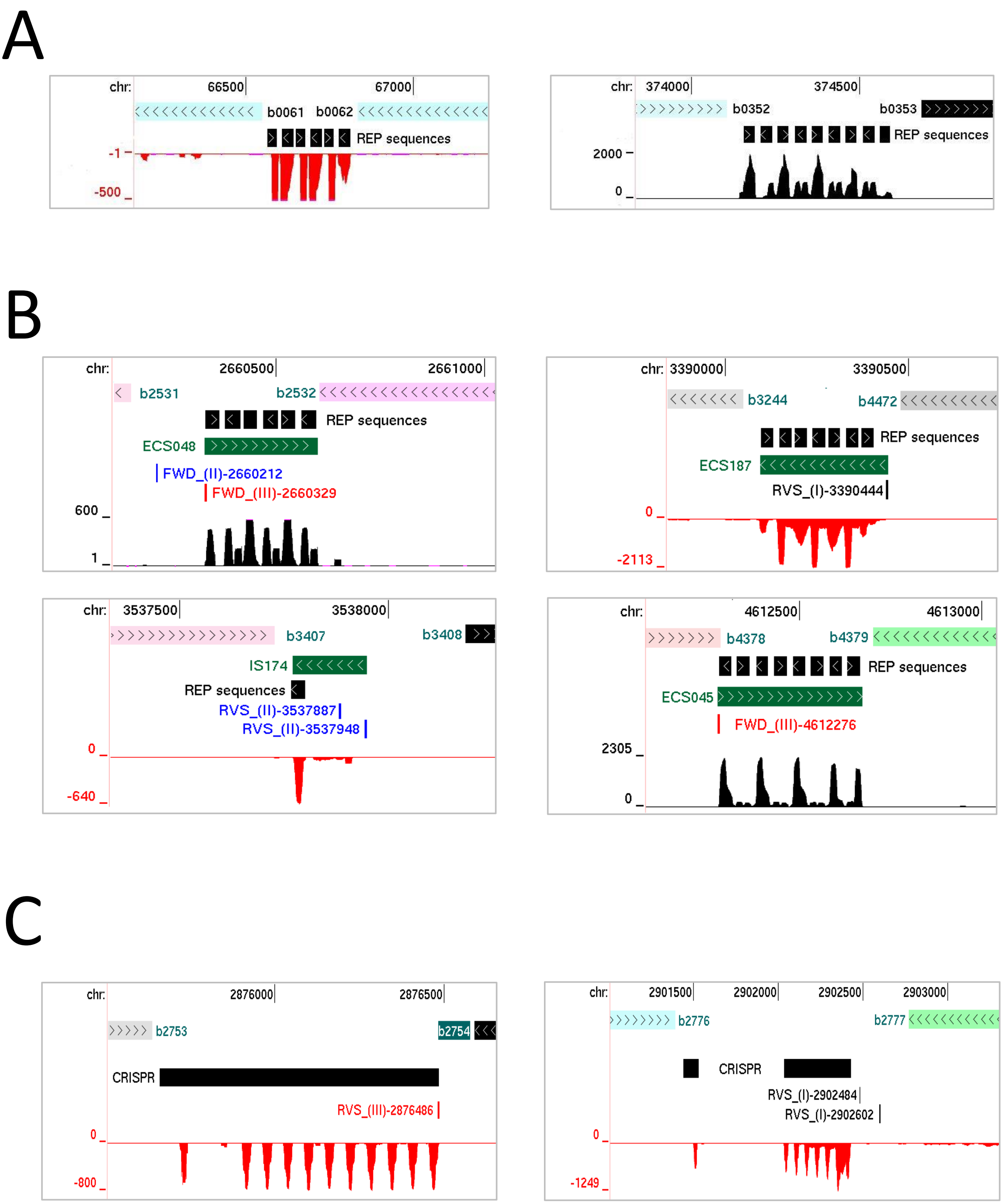

Fig. S2. sRNAs associated with repeat sequences in *E. coli.* **(A)** shows REP-associated RNAs from this study, **(B)** shows sRNAs previously identified by others and found to be associated here with REP elements, **(C)** shows the RNA profiles for the CRISPR loci of K12. The track depicting REP elements was generated using data derived from EcoCyc (Keseler et al., 2013). It should be noted when viewing the examples of REP elements that those with identical sequences cannot be distinguished when aligning short sequence reads to the genome.

A

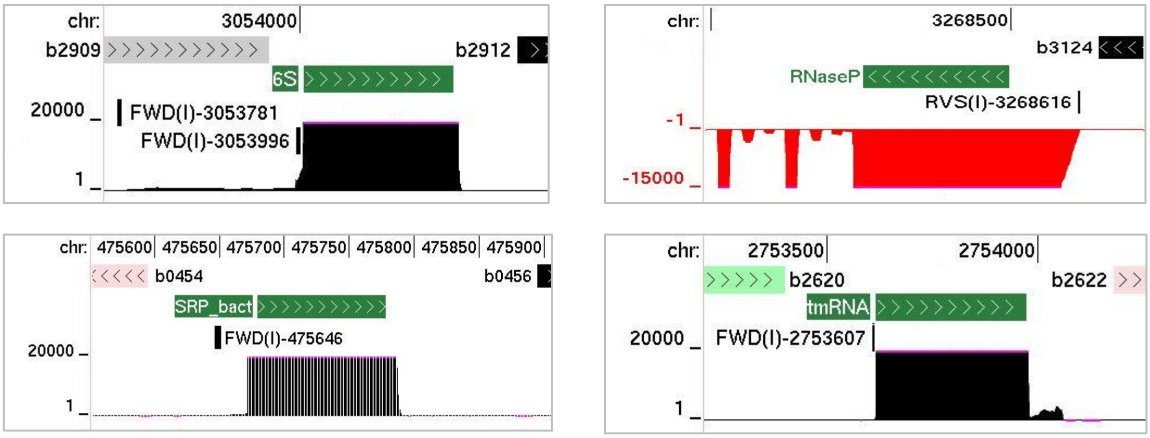

B

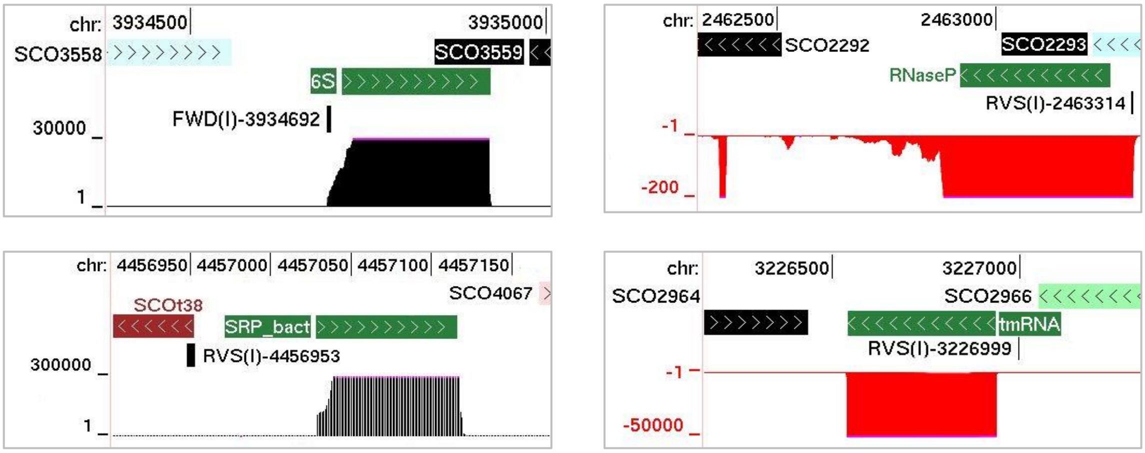

Fig. S3. Ubiquitous bacterial sRNAs. **(A)** and **(B)** show data for 6S RNA, the RNA components of RNase P and the signal recognition particle (SRP), and tmRNA for *E. coli* and *S. coelicolor*, respectively. The tracks from top to bottom show the genome positions, gene locations, TSSs and gRNA-seq reads. The *S. coelicolor* homologue of 6S RNA that is shown is the one known to interact with the housekeeping sigma factor (Panek et al., 2011). The other was poorly expressed in our sample.

Figure S4

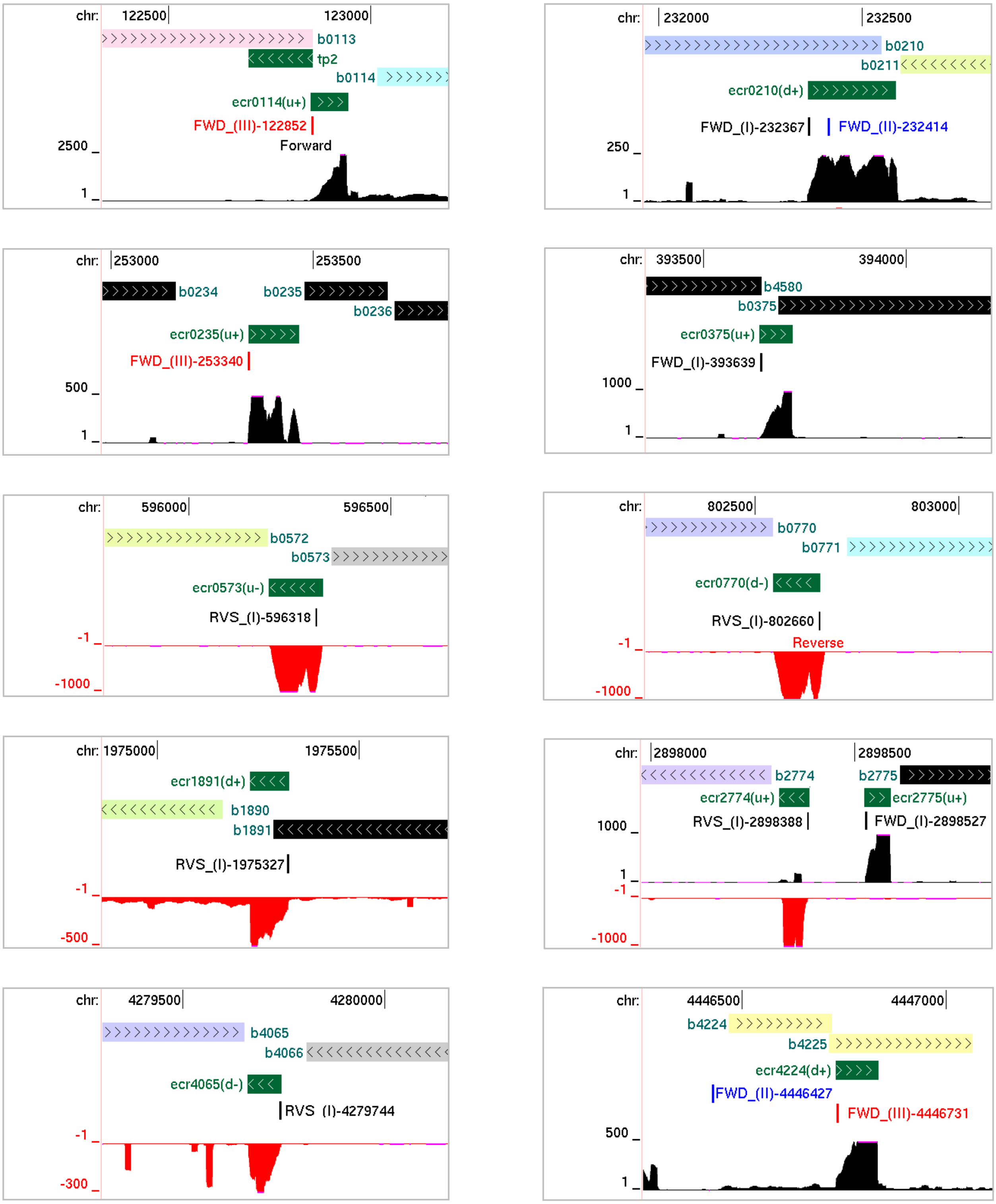

Fig. S4. Potentially novel small regulatory RNAs identified for *E. coli*. The tracks and labelling are as in Fig. S2. Labelling in parentheses indicates whether the sRNA is upstream (u) or downstream (d) of the nearest protein-coding gene and whether on the same (+) or opposite (-) strand. The prefix 'ecr' is used for discrete RNAs of unknown function. Five additional examples confirmed by northern blotting are shown in Fig. 8. The sRNA that was not confirmed by northern blotting is ecr0770(d-), which is in the middle row, middle column.

References

Altuvia, S., WeinsteinFischer, D., Zhang, A. X., Postow, L. & Storz, G. 1997. A small, stable RNA induced by oxidative stress: Role as a pleiotropic regulator and antimutator. Cell, 90**,** 43-53.

Andersen, J., Delihas, N., Ikenaka, K., Green, P. J., Pines, O., Ilercil, O. & Inouye, M. 1987. The isolation and characterization of RNA coded by the *micF* gene in *Escherichia coli*. Nucleic Acids Res., 15**,** 2089-101.

Argaman, L., Hershberg, R., Vogel, J., Bejerano, G., Wagner, E. G. H., Margalit, H. & Altuvia, S. 2001. Novel small RNA-encoding genes in the intergenic regions of *Escherichia coli*. Curr. Biol., 11**,** 941-950.

Burge, S. W., Daub, J., Eberhardt, R., Tate, J., Barquist, L., Nawrocki, E. P., Eddy, S. R., Gardner, P. P. & Bateman, A. 2013. Rfam 11.0: 10 years of RNA families. Nucleic Acids Res., 41**,** D226-32.

Chen, S., Lesnik, E. A., Hall, T. A., Sampath, R., Griffey, R. H., Ecker, D. J. & Blyn, L. B. 2002. A bioinformatics based approach to discover small RNA genes in the *Escherichia coli* genome. Biosystems, 65**,** 157-77.

Cipriano, M. J., Novichkov, P. N., Kazakov, A. E., Rodionov, D. A., Arkin, A. P., Gelfand, M. S. & Dubchak, I. 2013. RegTransBase - a database of regulatory sequences and interactions based on literature: a resource for investigating transcriptional regulation in prokaryotes. BMC Genomics, 14**,** 213.

Deana, A., H. Celesnik & J. G. Belasco, (2008) The bacterial enzyme RppH triggers messenger RNA degradation by 5' pyrophosphate removal. *Nature* **451**: 355-358.

Fozo, E. M., Kawano, M., Fontaine, F., Kaya, Y., Mendieta, K. S., Jones, K. L., Ocampo, A., Rudd, K. E. & Storz, G. 2008. Repression of small toxic protein synthesis by the Sib and OhsC small RNAs. Mol. Microbiol., 70**,** 1076-93.

Gerstle, K., Klatschke, K., Hahn, U. & Piganeau, N. 2012. The small RNA RybA regulates key-genes in the biosynthesis of aromatic amino acids under peroxide stress in *E. coli*. RNA Biology, 9**,** 458-68.

Goldman, S. R., J. S. Sharp, I. O. Vvedenskaya, J. Livny, S. L. Dove & B. E. Nickels, (2011) NanoRNAs prime transcription initiation *in vivo*. *Mol. Cell* **42**: 817-825.

Joyce, C. M. & Grindley, N. D. 1982. Identification of two genes immediately downstream from the *polA* gene of *Escherichia coli*. J. Bacteriol., 152**,** 1211-9.

Kawano, M., Reynolds, A. A., Miranda-Rios, J. & Storz, G. 2005. Detection of 5'- and 3'-UTR-derived small RNAs and *cis*-encoded antisense RNAs in Nucleic Acids Res., 33**,** 1040-1050.

Keseler, I. M., Mackie, A., Peralta-Gil, M., Santos-Zavaleta, A., Gama-Castro, S., Bonavides-Martinez, C., Fulcher, C., Huerta, A. M., Kothari, A., Krummenacker, M., Latendresse, M., Muniz-Rascado, L., Ong, Q., Paley, S., Schroder, I., Shearer, A. G., Subhraveti, P., Travers, M., Weerasinghe, D., Weiss, V., Collado-Vides, J., Gunsalus, R. P., Paulsen, I. & Karp, P. D. 2013. EcoCyc: fusing model organism databases with systems biology. Nucleic Acids Res., 41**,** D605-D612.

Masse, E. & Gottesman, S. 2002. A small RNA regulates the expression of genes involved in iron metabolism in *Escherichia coli*. Proc Natl Acad Sci U S A, 99**,** 4620-5.

Moody, M. J., Young, R. A., Jones, S. E. & Elliot, M. A. 2013. Comparative analysis of non-coding RNAs in the antibiotic-producing *Streptomyces* bacteria. BMC Genomics, 14**,** 558.

Nickels, B. E. & S. L. Dove, (2011) NanoRNAs: a class of small RNAs that can prime transcription initiation in bacteria. *J. Mol. Biol.* **412**: 772-781.

Panek, J., Bobek, J., Mikulik, K., Basler, M. & Vohradsky, J. 2008. Biocomputational prediction of small non-coding RNAs in *Streptomyces*. BMC Genomics, 9.

Panek, J., Krasny, L., Bobek, J., Jezkova, E., Korelusova, J. & Vohradsky, J. 2011. The suboptimal structures find the optimal RNAs: homology search for bacterial non-coding RNAs using suboptimal RNA structures. Nucleic Acids Res., 39**,** 3418-3426.

Pedersen, K. & Gerdes, K. 1999. Multiple *hok* genes on the chromosome of *Escherichia coli*. Mol. Microbiol., 32**,** 1090-102.

Raghavan, R., Sloan, D. B. & Ochman, H. 2012. Antisense transcription is pervasive but rarely conserved in enteric bacteria. MBio, 3.

Rivas, E., Klein, R. J., Jones, T. A. & Eddy, S. R. 2001. Computational identification of noncoding RNAs in *E. coli* by comparative genomics. Curr. Biol., 11**,** 1369-73.

Saetrom, P., Sneve, R., Kristiansen, K. I., Snove, O., Grunfeld, T., Rognes, T. & Seeberg, E. 2005. Predicting non-coding RNA genes in *Escherichia coli* with boosted genetic programming. Nucleic Acids Res., 33**,** 3263-3270.

Salgado, H., Gama-Castro, S., Peralta-Gil, M., Diaz-Peredo, E., Sanchez-Solano, F., Santos-Zavaleta, A., Martinez-Flores, I., Jimenez-Jacinto, V., Bonavides-Martinez, C., Segura-Salazar, J., Martinez-Antonio, A. & Collado-Vides, J. 2006. RegulonDB (version 5.0): *Escherichia coli* K-12 transcriptional regulatory network, operon organization, and growth conditions. Nucleic Acids Res., 34**,** D394-D397.

Salgado, H., Peralta-Gil, M., Gama-Castro, S., Santos-Zavaleta, A., Muniz-Rascado, L., Garcia-Sotelo, J. S., Weiss, V., Solano-Lira, H., Martinez-Flores, I., Medina-Rivera, A., Salgado-Osorio, G., Alquicira-Hernandez, S., Alquicira-Hernandez, K., Lopez-Fuentes, A., Porron-Sotelo, L., Huerta, A. M., Bonavides-Martinez, C., Balderas-Martinez, Y. I., Pannier, L., Olvera, M., Labastida, A., Jimenez-Jacinto, V., Vega-Alvarado, L., Del Moral-Chavez, V., Hernandez-Alvarez, A., Morett, E. & Collado-Vides, J. 2013. RegulonDB v8.0: omics data sets, evolutionary conservation, regulatory phrases, cross-validated gold standards and more. Nucleic Acids Res., 41**,** D203-13.

Shinhara, A., Matsui, M., Hiraoka, K., Nomura, W., Hirano, R., Nakahigashi, K., Tomita, M., Mori, H. & Kanai, A. 2011. Deep sequencing reveals as-yet-undiscovered small RNAs in *Escherichia coli*. BMC Genomics, 12**,** 428.

Sledjeski, D. & Gottesman, S. 1995. Small RNA acts as an antisilencer of the H-NS-silenced *rcsA* gene of *Escherichia coli*. Proc. Natl. Acad. Sci. USA, 92**,** 2003-2007.

Swiercz, J. P., Hindra, Bobek, J., Haiser, H. J., Di Berardo, C., Tjaden, B. & Elliot, M. A. 2008. Small non-coding RNAs in *Streptomyces coelicolor*. Nucleic Acids Res., 36**,** 7240-7251.

Tjaden, B., Saxena, R. M., Stolyar, S., Haynor, D. R., Kolker, E. & Rosenow, C. 2002. Transcriptome analysis of *Escherichia coli* using high-density oligonucleotide probe arrays. Nucleic Acids Res., 30**,** 3732-3738.

Vanderpool, C. K. & Gottesman, S. 2004. Involvement of a novel transcriptional activator and small RNA in post-transcriptional regulation of the glucose phosphoenolpyruvate phosphotransferase system. Mol. Microbiol., 54**,** 1076-1089.

Vockenhuber, M. P., Sharma, C. M., Statt, M. G., Schmidt, D., Xu, Z. J., Dietrich, S., Liesegang, H., Mathews, D. H. & Suess, B. 2011. Deep sequencing-based identification of small non-coding RNAs in *Streptomyces coelicolor*. RNA Biology, 8**,** 468-477.

Vogel, J., Bartels, V., Tang, T. H., Churakov, G., Slagter-Jager, J. G., Huttenhofer, A. & Wagner, E. G. 2003. RNomics in *Escherichia coli* detects new sRNA species and indicates parallel transcriptional output in bacteria. Nucleic Acids Res., 31**,** 6435-43.

Vvedenskaya, I. O., J. S. Sharp, S. R. Goldman, P. N. Kanabar, J. Livny, S. L. Dove & B. E. Nickels, (2012) Growth phase-dependent control of transcription start site selection and gene expression by nanoRNAs. *Genes Dev.* **26**: 1498-1507.

Wassarman, K. M., Repoila, F., Rosenow, C., Storz, G. & Gottesman, S. 2001. Identification of novel small RNAs using comparative genomics and microarrays. Genes Dev., 15**,** 1637-1651.

Weel-Sneve, R., Kristiansen, K. I., Odsbu, I., Dalhus, B., Booth, J., Rognes, T., Skarstad, K. & Bjoras, M. 2013. Single transmembrane peptide DinQ modulates membrane-dependent activities. PLoS Gen., 9.

Weilbacher, T., Suzuki, K., Dubey, A. K., Wang, X., Gudapaty, S., Morozov, I., Baker, C. S., Georgellis, D., Babitzke, P. & Romeo, T. 2003. A novel sRNA component of the carbon storage regulatory system of *Escherichia coli*. Mol. Microbiol., 48**,** 657-670.

Zhou, J. D. & Rudd, K. E. 2013. EcoGene 3.0. Nucleic Acids Res., 41**,** D613-D624.
